# Incentives for climate mitigation in the land use sector—the effects of payment for environmental services on environmental and socioeconomic outcomes in low‐ and middle‐income countries: A mixed‐methods systematic review

**DOI:** 10.1002/cl2.1045

**Published:** 2019-09-29

**Authors:** Birte Snilsveit, Jennifer Stevenson, Laurenz Langer, Natalie Tannous, Zafeer Ravat, Promise Nduku, Joshua Polanin, Ian Shemilt, John Eyers, Paul J. Ferraro

**Affiliations:** ^1^ International Initiative for Impact Evaluation London UK; ^2^ Africa Centre for Evidence (ACE) University of Johannesburg Johannesburg South Africa; ^3^ American Institutes of Research Washington District of Columbia; ^4^ Institute of Education University College London London UK; ^5^ Johns Hopkins University Baltimore Maryland

## Abstract

Unsustainable practices in the land use sector contribute to climate change through the release of greenhouse gases. Payment for environmental services (PESs) provide economic incentives to reduce the negative environmental impacts of land use and are a popular approach to mitigate climate change in low‐ and middle‐income countries. Some PES programmes also aim to improve socioeconomic outcomes and reduce poverty. This systematic review examines the effect of programmes on environmental and socioeconomic outcomes. We identified 44 quantitative impact evaluations and 60 qualitative studies of PES programmes for inclusion in the review, to assess both the effects of PES and identify context, design and implementation features that may influence PES effectiveness. The studies covered 18 programmes from 12 countries in Latin America and the Caribbean, East Asia and Pacific, South Asia and Sub‐Saharan Africa. The review finds that PES may increase household income, reduce deforestation and improve forest cover, but the findings are, however, based on low and very low quality evidence from a small number of programmes and should be treated with caution. Qualitative evidence indicates that several factors influence whether PES programmes are likely to be effective in different contexts and suggests that the inclusion of strong governance structures and the effective targeting of both locations and participants may improve intervention effectiveness. Funders, implementing agencies and researchers should collaborate to develop a coordinated programme of rigorous, mixed‐methods impact evaluation implemented across contexts. Until such evidence is available, PES programmes remain a high‐risk strategy for climate change mitigation.

## 1 PLAIN LANGUAGE SUMMARY

### 1.1 Payment for environmental services (PES) remains high‐risk strategy for climate change mitigation until rigorous impact evaluations can determine its effects

Programmes that provide economic incentives to reduce the negative environmental impact of land use are a popular means to reduce deforestation and degradation and mitigate climate change. In some cases they also aim to improve socioeconomic outcomes. The effects of PESs programmes on these outcomes, however, remain unclear due to the low quality of available evidence.

*Payment for environmental services (PES)programmes provide financial incentives for resource managers to adopt positive behaviours*.

### 1.2 What is the review about?

Greenhouse gas is released by unsustainable practices in the land use sector. PES programmes seek to create positive environmental outcomes by providing an economic incentive to the owners and managers of environmental services in low‐ and middle‐income countries to change their behaviour.

This review uses existing evidence to assess whether PES programmes have positive effects on environmental and socioeconomic outcomes. It also assesses how these effects vary across different contexts and implementation strategies.

### 1.3 What studies are included?

Studies were included that evaluated a PES programme in low‐ and middle‐income countries and targeted populations living in or near forests, agricultural land, wetlands, grasslands and mangroves.

Forty‐four impact evaluations and 60 qualitative studies were included. They covered 18 programmes from 12 countries in Latin America and the Caribbean, East Asia and Pacific, South Asia and Sub‐Saharan Africa.

Ten of the 18 programmes had as their objectives the improvement of both environmental and socioeconomic outcomes.

### 1.4 What are the main results of this review?

PES may produce reduced deforestation, improved forest cover and increased household income. These findings are, however, based on low and very low quality evidence from a small number of countries, and should be treated with caution.

Qualitative data indicates that the effects will vary, depending on where and to whom projects are targeted, the quality of implementation, presence of governance structures, contextual factors, and attitudes towards environmental protection and towards PES itself.

### 1.5 What do the findings of this review mean?

Until higher quality research is conducted, the large‐scale implementation of PES programmes should be considered a high‐risk strategy for mitigating climate change.

Based on the current evidence, strong conclusions about the impact of PES cannot be made, however effective targeting and including strong governance structures may improve project results.

To address the evidence gap, funders and implementing agencies should collaborate to develop rigorous methods for impact evaluation. They should also invest in the collection and analysis of qualitative data that examines diverse research participants and follows change over longer periods.

### 1.6 What is the aim of this review?

This Campbell systematic review examines the effects of PESs programmes on environmental and socioeconomic outcomes in low‐ and middle‐income countries. The review summarises findings from 44 quantitative and 60 qualitative studies from 12 countries.

### 1.7 How up‐to‐date is this review?

The review authors searched for studies in August and September 2017.

## 2 EXECUTIVE SUMMARY

### 2.1 Background

Around a quarter of all anthropogenic greenhouse gas (GHG) emissions originate from the agricultural, forest and other land use sectors (AFOLU), driven primarily by deforestation, forest degradation and emissions from unsustainable livestock, soil and nutrient management practices. At the same time, there is a large potential for climate change mitigation in the sector. Economic incentives‐based programmes, which aim to change behaviour around preserving or restoring ecosystems services, have grown in popularity in the last two decades. Initially such programmes were implemented for environmental conservation. But more recently they have been promoted as a climate change mitigation measure, and some programmes also aim to improve socioeconomic outcomes and alleviate poverty. PESs is one such approach where users of an environmental service pay the owners or managers of the service, conditional on changes in behaviours that are likely to affect the provision of environmental services. Despite their increasing popularity, key policy questions around the effectiveness of PES on both environmental and socioeconomic outcomes remain unanswered.

### 2.2 Objectives

To address the gaps in knowledge around effectiveness of PES, 3ie and the University of Johannesburg undertook a mixed methods systematic review, funded by the Children's Investment Fund Foundation (CIFF). The objective was to assess the effects of PES programmes on environmental and socioeconomic outcomes in low‐ and middle‐income countries (L&MICs). This assessment includes identifying and synthesising evidence on how PES programme effects vary by programme design, implementation, context and by subgroups of PES programme participants.

#### 2.2.1 Methods

##### 2.2.1.1 Search

We implemented a systematic and comprehensive search strategy, developed in consultation with an information specialist, following the Campbell Collaborations' guidelines to systematic searching. We searched a range of databases and websites, including general sources of social science literature as well as sources specific to climate change, forestry, agriculture and impact evaluation. We complemented this with citation tracking, checking reference list of included studies and existing reviews, and contacting experts. The searches were conducted in August–September 2017. At both the title and abstract and full‐text screening stages, all papers were double screened by two authors.

##### 2.2.1.2 Selection criteria

To address questions of intervention effects we included quantitative impact evaluations using experimental designs or quasiexperimental designs with nonrandom assignment that attempt to address confounding and selection bias in the analysis. To address questions related to intervention design, process and implementation we also included qualitative studies, project documents, process evaluations and cost data on the programmes examined. Studies had to evaluate a PES programme in countries classified by the World Bank as lower income, lower‐middle income, or upper‐middle income (L&MICs), targeted at populations living in or near to forests, agricultural land, wetlands, grasslands and mangroves.

##### 2.2.1.3 Data collection and analysis

We used a standardised data extraction form to extract data and critically appraise included papers, using a combination of Microsoft Excel and EPPI reviewer.^1^ We sused meta‐analysis to synthesise evidence on the effect of PESs when feasible, using an inverse‐variance weighted, random effects model. Where there were too few studies, or included studies were too heterogeneous in terms of interventions or outcomes, we report on the individual effect estimates only. For the qualitative synthesis, we conducted a thematic synthesis on intervention design, implementation and contexts that mitigate or reinforce intervention beffectiveness.

### 2.3 Results

#### 2.3.1 Characteristics of the evidence base

We identified 5,265 citations through the searching process, reduced to 4,742 papers when removing duplicates. After title/abstract and full‐text screening, we included 44 impact evaluation studies of 18 different PES programmes, and a further 60 studies for the qualitative thematic synthesis.

The 18 programmes took places in 12 countries covering Latin America and the Caribbean, East Asia and Pacific, South Asia and Sub‐Saharan Africa. Ten of the 18 programmes had as their objectives to improve both environmental and socioexonomic outcomes. Just over half of the evidence comes from three long‐standing PES programmes: the Payments for Hydrological Services Program (PSAH) in Mexico, the Programa de Pagos por Servicios Ambientales (PSA) in Costa Rica, and the Sloping Land Conversion Program (SLCP) in China.

Overall, the quality of the impact evaluation evidence in this area is low, with just over 50% of included impact evaluations rated as having a critical risk of bias. Being rated as having critical risk of bias means that studies fail to address all but one of do not adequately address more than one of the main methodological issues that may contribute to bias, namely intervention assignment mechanism, group equivalence and spill‐over effects. The results therefore need to be interpreted with caution.

The qualitative literature is limited in the type of evidence it provides. Only a small number of the included studies consist of rich qualitative studies that collect and analyse in‐depth qualitative data. The large majority of the included studies are of a descriptive nature and focus on factors affecting adoption of PES programmes. This dominance of descriptive designs limits the extent to which we are able to address the research questions related to programme design, process, implementation and contextual factors that may play a role in the effectiveness of PES programmes.

#### 2.3.2 Findings

##### 2.3.2.1 Effects of PES on socioeconomic and environmental outcomes

1. **The meta‐analyses suggest PES may increase household income, reduce deforestation and improve forest cover. However, the findings are based on evidence of low or very low quality and should therefore be interpreted with caution**. Moreover, the evidence comes from a small number of programmes, limiting the generalisability of the results.

###### 2.3.2.1.1 Effects on socioeconomic outcomes

The results from the meta‐analyses suggest a positive effect on overall household income (standardised mean difference [SMD] = 0.25, 95% confidence interval [CI] [0.09, 0.41]), household income from nonagricultural sources (0.05 SMD, 95% CI [−0.03, 0.13]) and a household income from agricultural sources (SMD = 0.11, 95% CI [−0.06, 0.29]). The meta‐analysis of three studies suggest no effect on household assets (SMD = 0.04, 95% CI [−0.12, 0.20]).

However, these results should be interpreted with caution for several reasons. First, most of the studies suffer from high or critical risk of bias, including all the studies of programmes in China. Second, the overall effects are largely driven by multiple studies drawing on independent samples to evaluate the effect of three large programmes in China. Third, the meta‐analysis based on a more diverse set of contexts of effects on household assets where no overall effect is observed include studies of PSA in Costa Rica, PES pilot in Malawi and PSAH in Costa Rica, with effects close to the line of no effects. Finally, the only low risk of bias study reporting effects on socioeconomic outcomes, while being underpowered, do not find a difference between the treatment and comparison groups in a PES pilot in Malawi (Jack and Santos, 2017).

Taken together, these limitations questions the generalisability of the results for socioeconomic outcomes. They may reflect the true effect of the PES programmes in this context, and considering the relatively large size of the payment, it is plausible they led to an increase in overall household income. But it is also possible the results in these studies are at least partially driven by bias.

###### 2.3.2.1.2 Effects on environmental outcomes

The results of the meta‐analyses suggest an improvement of forest cover (SMD = 0.32, 95% CI [0.10, 0.55]) and a reduction in deforestation (SMD = −0.12, 95% CI [−0.19, −0.05]). There is substantial heterogeneity attached to both estimates. For forest cover this is driven by the smaller effect observed for the PSAH in Mexico (Alix‐Garcia, Sims, & Yañez‐Pagans, 2015a), and removing this study from the analysis eliminates all heterogeneity and substantially increases the overall estimate to 0.43 (95% CI [0.25, 0.61]).

Overall the results suggest PES has improved environmental outcomes substantially in some contexts. As with the evidence on socioeconomic outcomes the results need to be interpreted with caution, although the average effects here are more precise and do not cross the line of no effect. Moreover, while issues with risk of bias remain overall, the evidence of beneficial effects is at least to some extent driven by studies with lower risk of bias, including the experimental study of PES in Uganda (Jayachandran, De Laat, Lambin, Stanton, Audy & Thomas, 2017). At the same time, the study by Alix‐Garcia et al. (2015a), which is among the more robust quasiexperimental studies we included, finds no substantive effect of PSAH on forest cover in Mexico.

In addition, the lack of measurement of environmental outcomes for seven of 18 programmes, despite conservation and climate change mitigation being a primary objective, suggests the overall effects may be influenced by outcome reporting bias in the literature.

##### 2.3.2.2 Context, design and implementation features that may influence PES effectiveness (research questions 2 and 3)

We identified a number of analytic themes from the qualitative data in terms of the role of design, implementation and context factors in influencing effectiveness of PES programmes. The main themes emerging from the qualitative synthesis are outlined below.

2. **Targeting (programme design): PES programmes need to be carefully targeted at the most relevant programme participants to support environmental and social outcomes**. Targeting is of particular relevance to support social outcomes such as poverty reduction and equity objectives.
  We find that the effects of PES are heterogeneous both across countries and within countries, highlighting the importance of PES targeting. The alignment of the programme targeting approach with the main objectives of the programme is central. If the programme targets a decrease in deforestation, participants and areas at the highest risk of deforestation need to be included. In programmes that also aim to address social objectives there is a need for deliberate efforts to also reach marginalised and vulnerable groups.
3. **Participation in the programme (implementation): Full participation in PES programmes presents a key factor for effective programme implementation**. The evidence suggests that participation has sometimes been hindered by a lack of beneficiary awareness and understanding of PES programmes.
  A lack of knowledge about the programme, perceived difficulties in completing programme enrolment and a lack of understanding of programme conditions and structures appear to have reduced programme take up among eligible participants. For some participants, even when they enrol in the PES programme, they do not fully understand its objective and conditionality.
4. **Programme governance and institutions building (design): PES programmes require strong governance structures within the communities in which they are implemented in order to monitor and ensure compliance and behaviour change**.
  Creating these governance structures presents a key mechanism through which programmes can achieve social objectives by supporting the building of local institutions and development structures.^2^
  The importance of strong programme governance structures emerged as a key theme in the thematic synthesis, both to monitor and support the compliance of participants with the PES conditionality as well as to build trust in the PES programme. The creation of local programme governance structures may also present a key mechanism through which programmes can achieve social objectives by supporting the building of local institutions and development structures.
5. **Factors that determine programme take up (context): A range of factors determine the uptake of PES programmes. The most common factors for adoption identified referred to existing levels of income, size of the land, availability of labour, the opportunity cost of participation, social norms and capital, and the state of the ecosystem service targeted**.
  The evidence suggests participants with a higher level of existing income, a more diversified income base and larger land are more likely to take up PES programmes. Similarly, landowners that depend to a larger extent on natural resources for their livelihoods and thus have a higher opportunity cost to join the programme, are less likely to enrol.
6. **Perception of nature (context/design): Perceptions of nature influence the design and relevance of PES programmes. Existing support for environmental protection supports programme implementation, but it is not clear if financial incentives undermine such existing, intrinsic motivation for environmental protection**.
  Existing support and adoption of practices related to conserving the environment emerge as a key facilitator for PES programmes. Somewhat unsurprisingly, where communities have already organised themselves to protect and conserve their natural resources or have positive attitudes towards environmental protection supports the implementation of PES programmes.
7. **Perceptions of PES (context): The majority of PES programmes was positively received by programme participants. However, a share of participants indicate they will revert to old practices in the absence of the PES programme**.

Across a range of contexts PES programmes were perceived positively by programme participants. But in three studies of large‐scale PES programmes, a substantive share of participants indicated that the adopted environmental practices (i.e., sloping land conversation, forest conservation, and silvopastoral practices) would not be sustained were the subsidies for them withdrawn.

##### 2.3.2.3 Cost‐effectiveness (research question 4)

8. **There is insufficient evidence to conclude whether PES provides a cost‐effective approach to support environmental and socioeconomic outcomes**.

The available evidence on cost‐effectiveness is limited and consists of different types of estimates, preventing any synthesis. The results available suggests a mixed picture, with authors finding PES to be cost‐effective in some contexts but not in others. Given the small sample of studies that this observational analysis is based on we are unable to conclude whether PES is a cost‐effective approach to support environmental and socioeconomic outcomes.

### 2.4 Authors' conclusions

There is nothing more disappointing for applied researchers than to conclude that more research is needed. But this is our main conclusion. Despite the hundreds of millions of dollars dedicated to PES programmes over the last decades, including by bilateral aid agencies, multilateral organisations and L&MIC governments, we are unable to determine with any certainty if these are worthwhile investments.

While the limited meta‐analyses which we are able to conduct in this review suggest that, in particular contexts, PES may have positive effects on selected environmental and socioeconomic outcomes, these findings cannot be generalised and remain highly programme‐specific. The evidence base is characterised by quasiexperimental impact evaluations with a high or critical risk of bias. There is also a lack of common outcome measures across studies, making it more challenging to draw lessons across contexts. Moreover, the majority of studies focus on three long‐standing programmes in Costa Rica, Mexico and China, although there is an absence of any evidence on the effect of PES programmes on environmental outcomes in China.

Given the findings of our review, the role of deforestation and land‐use change as a source of greenhouse gas emissions and the critical need to identify effective mitigation strategies, we conclude that the large‐scale implementation of PES is a high‐risk strategy. Our primary conclusion is therefore that there is an urgent need to integrate rigorous impact evaluation with the roll‐out of any new PES programme. This echoes repeated calls for rigorous evidence on the effects of PES over the least the last decade (Ferraro, 2011; Ferraro & Pattanayak, 2006; Samii, Lisiecki, Kulkarni, Paler, & Chavis, 2014).

### 2.5 Implications for practice and policy

With the above caveats in mind, we nevertheless identify a number of implications for decision‐makers working on the design and implementation of conservation and development programmes such as PES. These implications need to be adapted to specific contexts, including by drawing on additional local evidence and expert knowledge to be appropriately translated to recommendations for policy and programme design.

1. Whether to invest in PES programmes: The findings of our review suggest reasons to be cautious about investing in the implementation of PES programmes in LMICs. Given the current available evidence base, we do not know whether PES programmes do in fact achieve desired environmental and, in particular, social outcomes. Given the need for mitigation interventions with transformational effects in the forestry sector, we regard the large‐scale implementation of PES programmes as a high‐risk strategy.
2. Investing in PES programmes with built‐in piloting and evaluation: There is suggestive evidence that PES may deliver positive effects on both environmental and socioeconomic outcomes in some contexts. But because of the limitations of the existing evidence we suggest careful piloting and evaluation as a prerequisite when investing in the implementation of a PES programme in a new context.
3. Targeting of PES programmes: The heterogeneous effects of PES across and within countries highlight the importance of PES programmes being carefully targeted at the programme participants and contexts with the largest potential for environmental and socioeconomic benefits. Targeting criteria that the qualitative evidence suggests to enhance the relevance of PES programmes to environmental and social objectives include: targeting at areas with high‐risk of deforestation; targeting at the specific contexts of low‐income groups and targeting at characteristics of the locality (e.g., type of forests, sloping, proximity of existing infrastructure and industrial development).
4. PES governance structures as a win‐win strategy: Based on qualitative evidence, PES governance structures emerge as key design criterion that might be able to support PES as a win‐win strategy for environmental and social objectives. Governance structures are central in ensuring programme implementation and compliance, thereby supporting environmental outcomes. At the same time, creating strong local governance structures can also support PES's social objectives by ensuring programmes are accessed by all stakeholders and that benefits are shared equitably.

### 2.6 Implications for research and evaluation

Addressing the lack of available high quality research can be best addressed in the form of coordinated action by funders, implementing agencies and interdisciplinary research themes. There are two main avenues for improving the impact evaluation evidence base, and we suggest they are pursued in parallel.

- To develop a common framework for the design and implementation of theory based, mixed methods impact evaluations to be conducted in conjunction with the roll out of new programmes. Such studies should be conducted across multiple contexts to identify generalisable and context specific findings. They should assess effects on a common set of environmental and socioeconomic outcomes, including deforestation, GHG emissions, household income and food security. To identify and address potential unintended negative socioeconomic effects studies should draw on existing literature to anticipate and collect data on such outcomes for relevant populations in a particular context, including an integrated approach to assessing effects on gendered inequality.
- Exploit opportunities to draw on existing data to assess the effect of programmes that are already ongoing or completed. Several of the included studies combined different econometric techniques, such as propensity score matching (PSM) and fixed effects panel regressions to evaluate the effect of PES programmes using existing data sets. The University of Maryland hosts a freely available and regularly updated the time‐series Landsat data set which characterise forest extent, loss and gain globally from 2000–2017 which could be utilised for such studies. Combining panel data with an understanding of the factors that affected programme implementation (treatment assignment mechanism) can be a strong design for estimating PES impacts.

In terms of the available qualitative evidence base, we suggest to focus on a range of weaknesses in the existing evidence base. Future qualitative research should:

- More systematically invest in the collection and analysis of in‐depth qualitative data when planning and conducting impact evaluations. This is likely to increase the relevance of the evaluations and to facilitate a better understanding of programme mechanisms and design factors.
- Diversify the research participants to present a more reflective picture of all PES programme participants. This includes how different societal groups can access and experience PES programmes; and how equity objectives can be fully integrated within PES programme design and implementation.
- Invest in longitudinal, in‐depth qualitative data. The majority of the included qualitative studies are small‐scale (*n* < 30) and conducted over a short time frame (±6 months). To understand how programme implementation changes and affects participants over time, more longitudinal, in‐depth qualitative data is required.

## 3 BACKGROUND

### 3.1 The issue

Around a quarter of all anthropogenic GHG emissions originate from the AFOLU, driven primarily by deforestation, forest degradation and emissions from unsustainable livestock, soil and nutrient management practices (IPCC, 2014). But there is also a large potential for climate change mitigation in the sector, through removal of GHGs in the atmosphere (carbon sequestration) and reduction in emissions from reduced forest and vegetation removal and improved agricultural practices.

The AFOLU sector also provides a range of other ecosystem services in addition to climate regulation.^3^ Forests and lands provide clean water, regulate soil and provide food, fuel, fibre and fresh water (MEA, 2005). Agriculture provides directly and indirectly for the livelihoods of billions of people, in addition to providing food for all the world's population (FAO, 2016a). The sector also offers livelihoods for an estimated 750 million of the world's extreme poor (FAO, ibid). Finally, forests provide paid employment for at least 100 million people and support the livelihoods of many millions more (FAO, 2016b).

The United Nations Framework Convention for Climate Change (UNFCCC) has recognised the critical importance of reducing emissions from deforestation and degradation for climate mitigation (UNFCCC, 2010). In addition, the IPCC highlights the importance of preservation and restoration of other ecosystems such as peatlands and mangroves for maintaining carbon stocks and reducing emissions (FAO & IPCC, 2017; IPCC, 2014). Improved livestock and crop management also represent practices with mitigation potential (FAO & IPCC, ibid).

The links between climate change, agriculture, forests and human wellbeing are complex. The world's forest area declined from 4,128 million hectares of forest in 1,990–3,999 million hectares in 2015 (FAO, 2016c). Agriculture, both commercial and subsistence, was the main driver of this global deforestation, accounting for 73% of forest clearance worldwide (FAO, 2016b). This is partially driven by an increasing global demand for food from increasing incomes and growing populations, which is expected to rise 60% from 2006 levels by 2050 (FAO, 2016a). At the same time, climate change is expected to negatively affect all dimensions of food security, including agricultural production of food, quality, food access through the impacts on livelihoods and food price stability (IPCC, 2014).

These complex relationships make sustainable preservation and management of forests and land, while at the same time ensuring food and livelihoods for the world's population, one of the biggest policy challenges facing the world (FAO, 2016a, 2016b). Concerns that climate change mitigation programming may have negative knock‐on effects on human wellbeing and human rights, especially for the poor, remain (Larson et al., 2013; Lawlor, Madeira, Blockhus, & Ganz, 2013; Mutabazi, George, Dos Santos, & Felister, 2014; Stickler et al., 2009). It is therefore important to identify strategies that reduce trade‐offs between environmental protection and human wellbeing, and ideally programmes that offer win‐win solutions.

### 3.2 The intervention

Economic incentives‐based programmes, which aim to preserve or restore ecosystems services through financial incentives, have grown in popularity in the last two decades (Ezzine‐de‐Blas, Wunder, Ruiz‐Pérez, & Moreno‐Sanchez, 2016; GEF, 2014; Pirard, 2012). One such incentive‐based mechanism is PESs. PES is a market‐based approach, where users of an environmental service pay the owners or managers of the service, conditional on changes in behaviours that are likely to effect the provision of environmental services (Wunder, 2015). PES may be conditional on commitments to protect or restore forest areas or sustainable forest management, such as management of forest fires (Alix‐Garcia et al., 2014; Jayachandran, de Laat, Lambin, & Stanton, 2016). Payments may also be tied to agricultural practices associated with reduction in GHG emissions or increase of carbon stocks, including introduction of agroforestry, silvo‐pastoral or integrated crop systems, which combine crops, grazing lands and trees on agricultural land, improved tillage practices such as conservation agriculture, and reduced use of fire in rangeland management (Garbach, Lubell, & DeClerck, 2012; Hedge & Bull, 2011).

There is some debate on the definition of PES (Engel, Pagiola, & Wunder, 2008; Muradian, Corbera, Pascual, Kosoy, & May, 2010; Wunder, 2015). At the simplest level, PES is a voluntary transaction between service users and service providers, conditional on agreed rules for natural resource management that aims to generate environmental services or benefits that are felt off‐site, for example, carbon sequestration (Wunder, 2015). In practice, the service “user” is typically a government or NGO acting on behalf of beneficiaries of the environmental service and the service “providers” are individuals, households or community organisations that own or manage the land or forest areas in the programme.

There are a number of long‐standing PES programmes in existence around the world, for example, the Pago por Servicios Ambientales‐Hidrologico (PSAH) in Mexico and the SLCP in China. The PSAH in Mexico makes payments to landowners conditional on maintenance of certain level of forest cover, according to 5‐year contracts (Alix‐Garcia et al., 2014). If forestland is converted to another land use such as agriculture, the landowner is removed from the programme. The SLCP in China is a large‐scale programme that aims to incentivise the conversion of cropland back to forests or grassland through cash and in‐kind payments to participating households, to reverse or prevent soil erosion and desertification (Démurger & Wan, 2012). In addition to these long‐standing programmes, the number of new PES programmes has grown rapidly in the last decade (Börner et al., 2017). They increasingly also include goals around poverty alleviation. For example, while the original goal of the PSAH was to maintain the provision of hydrological services from Mexico's forested land, in 2006 the objectives were extended to alleviating poverty (Alix‐Garcia et al., ibid).

Because of the restrictions around land use from participating in the programme, implementers of PES programmes sometimes combine them with other activities to support behaviour change, such as awareness raising activities around environmental conservation or capacity building in sustainable resource use (Sharma & Pattanayak, 2015). In some cases, they are also combined with more extensive support for livelihoods development. For example, a REDD+ pilot programme in Nepal made incentive‐based payments to Community Forest User Groups (CFUGs). In addition to forest carbon monitoring, this programme included awareness raising and capacity building for improving local livelihoods and the use of alternative fuel and cooking technologies (Sharma & Pattanayak, ibid).

### 3.3 How the intervention might work

PESs are frequently framed as a response to “market failure” (Arriagada & Perrings, 2009). A market failure occurs when the market does not provide a socially optimum level of a service or good because of the presence of positive externalities for society from providing the service. Carbon sequestration is an example of a public good with positive externalities felt at the global level (Alix‐Garcia & Wolff, 2014). While households may get some individual benefits from environmental practices such as keeping trees on land, the larger benefits are felt externally but households are not compensated financially for these external benefits by market mechanisms. This leads to household or individual decisions that are suboptimal for society, like deforestation.

**Figure 1:**
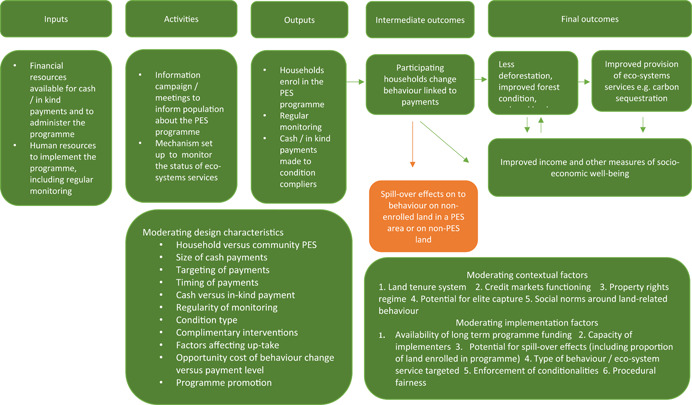
Proposed theory of change for payment for environmental services programmes [Color figure can be viewed at wileyonlinelibrary.com]

The overarching theory of how PES works is quite simple. It is designed to act as an incentive for a household or community to contribute to the provision of a socially optimal level of environmental services, thus correcting the market failure. Figure 1 presents a programme theory for how PES may influence environmental and socioeconomic outcomes. The outcomes presented in the model are not the only potential outcomes of PES programmes, however we have chosen to focus on those that are of direct interest in this review.

#### 3.3.1 How PES may influence environmental outcomes

The intervention aims to influence environmental outcomes primarily through provision of a positive financial incentive to change environment‐related behaviours (Pattanayak, Wunder, & Ferraro, 2010). Cash or in‐kind payments are typically made to participating individuals, households or communities on a regular basis, conditional on the environmental behaviour, for example, payments to landowners to avoid deforestation on their land. Payments may come from private actors that directly benefit from the environmental service, but more typically come from government or nongovernmental organisations acting on their behalf. If a participating household or community organisation fails to uphold the minimum environmental service provision, payments are suspended.

The theory underlying PES is that the financial incentives motivate participants to comply with the rules of the programme, resulting in improved land or forest management practices (Alix‐Garcia & Wolff, 2014). The theory is that the increase in take‐up of these improved practices will ultimately restore, maintain or enhance the provision of the environmental service that has wider benefits for society. The theory assumes that the conservation payments outweigh the benefits derived from business as usual, such as converting forests to agricultural uses, or harvesting wood for energy.

PES may have positive or negative spill‐over effects on land that is not enroled in the programme. If households or communities do not enrol all their land in a programme, resource exploitation pressures may simply move on to the nonenroled areas, known as leakage or substitution effects (Sills et al., 2008). Similarly, increased household income because of the PES programme may have implications for spending patterns and put increased pressure on local resources (Börner et al., 2017). Conversely, positive spill‐overs may occur due to increased forest monitoring resulting from the programme or changes in social norms relating to resource use. Such indirect effects can affect the magnitude or even the direction of the effect of a PES programme (Pattanayak et al., 2010).

#### 3.3.2 How PES may influence environmental and socioeconomic outcomes

While not originally intended as a tool for poverty alleviation, PES may increase income for complying individuals or households. To directly increase household income, the assumption is that the cash transfer is greater than lost rents previously generated from the enroled land. Alternatively, payments may also indirectly act as an incentive for households to diversify towards other livelihood activities that are less reliant on practices that reduce the provision of the ecosystems services. For example, participants may move away from agriculture that relies on regular forest clearing towards sustainable forest activities.

However, there are potential trade‐offs between poverty alleviation and environmental goals. The effectiveness of PES in improving environmental outcomes is theorised to depend on effective targeting towards those actors that are the biggest threat to the provision of the environmental service (Börner et al., 2017). If the biggest threat comes from larger, better off households or communities, the payment is best targeted towards them, but this will come at the cost of income transfers to poorer families that could support poverty alleviation (Alix‐Garcia & Wolff, 2014).

A range of programme design, implementation and contextual factors may influence the effectiveness of PES programmes. Below are some key design, implementation and contextual variables that are frequently theorised to moderate the effectiveness of PES schemes. In many cases, the theory is not conclusive on whether the impact on effectiveness would be positive or negative and thus on the direction of effects of PES schemes in general (Ferraro, 2017; Pattanayak et al., 2010). These factors are explored in the review in the analysis of heterogeneity.

##### 3.3.2.1 Targeting can influence whether PES programmes achieve their objectives

PES programmes are typically voluntary and there is a risk that households that already meet conditions will self‐select into the programme. Depending on the opportunity cost of participating in the programme, households may choose not to enrol or only enrol some of their land (Ferraro, 2017). Land enroled in PES programmes may therefore be land with the lowest value in terms of exploitation potential and thus the least likely to be exploited in the absence of PES. The result of this would be little or no added benefit of the programme in terms of environmental outcomes as land owners may have preserved resources even in the absence of payments.

The lack of additionality may therefore be more prevalent where preprogramme compliance with PES conditions is high (e.g., low levels of resource exploitation, as indicated by low baseline deforestation rates for example). Thus, programmes targeted to land that is at a high‐risk of exploitation may result in higher levels of resource protection. However, this involves predicting where landholders will exploit resources in the future, information that is generally hidden from the policymaker implementing the PES programmes (Alix‐Garcia & Wolff, 2014).

##### 3.3.2.2 The size of payments may influence take‐up and the extent to which programme participants change their behaviour

If the cost of lost rents from restrictions on land or resource use from participating in the programme are greater than the payments received, a land owner is unlikely to choose to enrol. This requires a payment that is large enough to overcome the opportunity costs for households to decide to participate in the programme and then to stick to conditions. However, because of missing markets the payment size that will induce people to participate in the programme cannot be directly observed (Börner et al., 2017).

##### 3.3.2.3 Timing of payments

The timing of payments may influence how programme participants respond to the financial incentive. There is some suggestion that payments made at the end of the contracted period are most effective at incentivising changes in environmental behaviours (Alix‐Garcia & Wolff, 2014). However, this is often not feasible, particularly in low‐income contexts, and often payments are made on a yearly basis.

##### 3.3.2.4 The characteristics of PES conditions

Even if an improvement in an ecosystem service is the goal of a programme, few PES programmes are conditional on the provision of the ecosystem service itself (such as demonstrated increases in carbon sequestration in forests). In practice, PES programme payments are frequently conditional on proxies or changes in behaviours that are likely to affect the provision of the ecosystem service (Wunder, 2015). For example, planting trees on agricultural land to improve carbon sequestration. While the use of proxies is typically easier to observe, there is no guarantee that changes in the behaviour will lead to improved ecosystems provision, particularly where the ecosystem service is heavily influenced by external factors to the programme (Börner et al., 2017; Pattanayak et al., 2010).

##### 3.3.2.5 The extent to which conditions are monitored and enforced

Monitoring and enforcement of conditions may moderate effects on environmental outcomes (Börner et al., 2017). Monitoring and enforcement may influence the extent to which programme participants change their behaviour and comply with conditions. A systematic review of the effects of cash transfer payments for building human capital found a larger effect on children's education outcomes when conditions were monitored and enforced (Baird, Ferreira, Özler, & Woolcock, 2013).

##### 3.3.2.6 Long run programme funding

Permanent benefits of PES schemes may depend on continuous programme funding, which may be particularly difficult in government run PES schemes (Engel et al., 2008). On the other hand, payments may act to incentivise people to incur the fixed costs of switching to a more environmentally friendly practice and to “learn by doing” (learn about benefits and learn to reduce variable costs). And, once a new practice is adopted, the marginal benefits may outweigh the marginal costs and the practice will persist even in the absence of payments.

##### 3.3.2.7 Property rights system

Weak property rights are a common driver of deforestation and lack of secure property rights may make PES implementation difficult (Alix‐Garcia & Wolff, 2014). Lack of secure property rights may reduce programme take‐up rates and compliance as participants are less willing to invest in the sustainable management of land when they are uncertain if they will be able to reap benefits from those investments in the future.

##### 3.3.2.8 Land tenure system

Incentives to change behaviour around land management practices may depend on whether the land is privately owned, collectively owned, state owned or restricted in some way by the state (Robinson et al., 2017). For example, PES payments may have weaker effects on conservation behaviour of users living in or near state owned lands than on private land or land held under collective title.

##### 3.3.2.9 Credit markets

The presence of credit constraints for poor families in LMICs may be a barrier for them to make investments in, or exploit land (Ferraro, 2017). There may be negative environmental consequences when payments to participating families allow them to overcome these constraints to make investments in unenrolled land, or enroled land once payments stop, that result in less environmentally favourable land uses.

### 3.4 Why it is important to do the review

#### 3.4.1 Review of existing literature

There is an emerging impact evaluation literature on PES programmes. A 3ie evidence gap map (EGM) published in 2016 identified 41^4^ experimental or quasiexperimental evaluations of PES programmes globally, with most taking place in L&MICs. We are only aware of one systematic review on the effectiveness of PES, published in 2014 (Samii et al., 2014). There have also been a large number of nonsystematic literature reviews, either presenting narrative discussions on the effectiveness of PES (Alix‐Garcia & Wolff, 2014; Börner et al., 2017; Pattanayak et al., 2010) or presenting a range of effect sizes for PES programmes (Ferraro, 2017).

There are several reasons that warrant an update and extension of the Samii et al. (2014) systematic review. First, the search for the review was completed in August 2013. 3ie's Evidence Gap Map of land use and forestry programmes (Snilstveit et al., 2016) identified at least six new evaluations of PES programmes that have been published since then, including studies from Uganda, Ecuador, Tanzania and new evaluations of long‐term programmes in China, Mexico and Costa Rica. Second, Samii et al. (2014) were unable to conduct a meta‐analysis for income and poverty related outcomes and for forest condition due to lack of data and heterogeneity between studies. Given the increase in the evaluation evidence base since then, we anticipated to be able to undertake additional meta‐analyses.

Third, Samii et al.'s (2014) review focused on PES for forest areas. We expand the scope of the review to include PES in other settings such as farmland, mangroves and grasslands. A number of PES programmes target other important environmental behaviours of relevance to climate change mitigation programming, for example, payments to incentivise farmers to take up agroforestry on their farmland (Hedge & Bull, 2011). This is the first review that we are aware of to systematically cover the literature on the effectiveness of PES in these areas.

Finally, this review answers new questions around design, implementation, context and costs of programmes, in addition to assessing programme effects. In doing so, we look at a broader range of literature, including process evaluations, programme documents and associated qualitative studies for the programmes evaluated in included impact evaluations.

#### 3.4.2 Relevance to policy and practice

It is estimated that additional global investments of US$35 billion in the agriculture sector and US$21 billion in the forestry sector will be needed by 2030 to mitigate the effects of climate change (UNFCCC, 2009). At the landmark United Nations Climate Change Conference (COP 21) in 2015, countries agreed to conserve and enhance sinks of GHGs, including forests (UNFCCC, 2015). To ensure resources are used effectively to achieve agreed mitigation objectives it is important to ensure that decision‐makers have access to a reliable and synthesised evidence base.

The United Nations Reducing Emissions from Deforestation and Forest Degradation mechanism (REDD+) is one of the main frameworks for making payments to L&MICs to preserve and sustainably manage forests. There are significant resources pledged to the REDD+ initiative. At the COP21, Germany, Norway and the UK announced that they would provide US$ 5 billion between 2015 and 2020 to forest countries if they could demonstrate verified emissions reductions (BMUB, 2015). The UN‐REDD Programme currently supports 64 countries across Africa, South and East Asia and Latin America and the Caribbean to enable their participation in REDD+, and 47 so far have qualified (UN‐REDD, 2016).

PES are promoted as an important tool by REDD+ and are supported by a range of actors, from national governments to multinational institutions such as IFAD, UNDP and the World Bank (GEF, 2014). The number of PES programmes operating in L&MICs has rapidly increased. A recent global review of PES identified hundreds of programmes mentioned in the literature, with 55 programmes currently in operation around the world that clearly fit the classic definition of PES (Ezzine‐de‐Blas et al., 2016). The Global Environmental Facility (GEF) alone has supported 57 projects containing elements of PES since its inception, totalling investments of over $225 million, in addition to $1.59 billion leveraged from cofinancing (GEF, 2014).

Despite their popularity, key policy questions around the effectiveness of PES remain unanswered (Ferraro, 2017; Le Velly & Dutilly, 2016; Samii et al., 2014). One of these questions is the extent to which the environmental and poverty reduction goals of such a programme conflict or present strategies that can generate both environmental and poverty reduction benefits. A second, and equally important question is if PES generate environmental benefits that are additional to “business as usual”. To meet UNFCCC emissions targets, governments implement PES programmes on the assumption that by compensating some groups to reduce their emissions, emissions in other sectors are offset (Nhantumbo & Camargo, 2015).

Evaluations of PES programmes finding small effects have led some to dismiss it as an important mechanism. Indeed, a recent FAO‐IPCC (2017) report on climate change and land use following the Paris Agreements stated that “[PES] effectiveness, however, is limited and they are more readily applied in some sectors (*e.g.,* forest management) than in other emerging concerns (land restoration, soil health and soil carbon)” (FAO‐IPCC, 2017, p. 28). The report concludes that for PES programmes to be effective, they must be better designed and informed by meta‐analysis of the effects of previous programmes.

A range of policy alternatives to PES exist, including private sector zero‐deforestation commitments (Climate Focus, 2015) and community forestry initiatives (Agrawal & Angelsen, 2009; Angelsen, 2009). Though, the effectiveness of many of these approaches is also contested and should be subject of future reviews. While PES may be one of the most popular policy tools in the sector, it is important to assess the relative costs and effectiveness of the approach, facilitating comparison with other options in the future.

Given the resources dedicated to PES and the global importance of effective climate change mitigation activities, it is essential that rigorous and comprehensive evidence is available to policymakers and implementers. To help inform decisions about how to use available resources most effectively we provide a comprehensive review and synthesis of the evidence on the effects of PES, including an assessment of how intervention design, implementation and contextual factors moderate outcomes and cost‐effectiveness.

## 4 OBJECTIVES

The objective of this review is to assess the effects of PES programmes on environmental and socioeconomic outcomes in L&MICs. This includes identifying and synthesising evidence on how PES programme effects vary by programme design, implementation, context and by subgroups of PES programme participants. We also attempt to assess the cost‐effectiveness of PES programmes.

To address these objectives, we aimed to answer the following questions:

1)
  a) What is the effectiveness of PES programmes on intermediate, environmental and socioeconomic outcomes in L&MICs?
  b) Do PES programs simultaneously deliver positive environmental and socioeconomic effects?
  c) Do effects vary by subgroups of people participating in PES programmes, including low‐income groups, women and indigenous people?
2) Do effects vary by type of environmental services targeted?
3) To what extent do design and implementation features moderate the effectiveness of PES programmes?
4) In which contexts are PES programmes effective (or ineffective)? What are the contextual barriers to, and facilitators of, programme effectiveness?
5) What is the cost‐effectiveness of PES programmes?

## 5 METHODOLOGY

The review followed the Campbell and Cochrane Collaborations' guidelines to systematic reviewing (Hammerstrøm, Wade, & Jørgensen, 2010; Higgins & Green, 2011; Shadish & Myers, 2004; The Steering Group of the Campbell Collaboration, 2016). It also drew on the concepts of theory‐based impact evaluation (White, 2009) and theory‐based systematic reviews (Snilstveit, 2012; Waddington et al., 2012) to provide a mixed‐methods systematic review and analysis along the causal chain, to also address questions related to intervention design, implementation and context. We conducted the review following the methods outlined in a published protocol (Snilstveit et al., 2018), and also described in this section.

We included studies in two phases. To address questions 1a, b and c, we included studies meeting the impact evaluations study design criteria, presented below. To address questions 2, 3 and 4, studies that meet these criteria were used as the basis for a second, targeted search to identify and include qualitative studies, project documents, process evaluations and cost data on the programmes examined.

### 5.1 Criteria for considering studies for the review

#### 5.1.1 Type of population

We included studies of programmes in countries classified by the World Bank as lower income, lower‐middle income, or upper‐middle income. We use the classification of the country in the year of the initiation of the programme under study. There are several reasons why we decided to focus on L&MICs only. Some scoping of the literature suggests that the impact evaluation literature on PES from high‐income countries (HICs) is significantly smaller and does not typically use methods that would be included in the review (Schomers & Matzdorf, 2013; Snilstveit et al., 2016). It does not typically self‐identify as PES (Ezzine‐de‐Blas et al., 2016; Schomers & Matzdorf, 2013) and would likely result in a need to search a separate literature. This would have likely to added a significant amount of work to the searching and screening with only a potentially very small number of included studies. In addition, L&MICs contain most of the world's tropical forests, which offer the greatest potential for climate change mitigation in the AFOLU sector, such as climate regulation, watershed protection and carbon sequestration (Pattanayak et al., 2010). Similarly, the findings from the HIC literature would be less relevant for mechanisms such as REDD+. Finally, given that one of our main objectives was understanding the potential for PES to offer “win‐win” environmental and poverty alleviation solutions, L&MIC contexts offer a more likely setting for answering this. Studies of programmes in HICs were therefore excluded.

We included studies targeted at populations living in or near to forests, agricultural land, wetlands, grasslands and mangroves. Forests are defined as an area over 0.5 hectares with trees higher than 5 m and canopy cover more than 10% (FAO, 2012), including mangrove forest areas. Grasslands are areas with tree or shrub canopy cover below 10% but with herbaceous plant cover (FAO, 2005).

#### 5.1.2 Type of interventions

We included studies of PES programmes, defined as those providing payments to owners or managers of land, conditional on some minimum environmental/ecosystems service provision. Payments could either be cash or in‐kind material transfers, such as seedlings, apiculture and fencing. Ecosystems services are defined as the benefits that humans get from ecosystems (MEA, 2005). In ideal type PES programmes, payments are conditional on the provision of the ecosystem service itself, for example, payments for increased carbon sequestration in forests (Le Velly & Dutilly, 2016). However, in practice most PES programme payments are conditional on changes in behaviours that are likely to affect the provision of the ecosystem service, for example, reducing deforestation or planting trees on agricultural land. We included payments tied either to the provision of an ecosystem service or to any of the following practices related to climate‐regulating ecosystems services: forest protection or regeneration, sustainable forest management practices, sustainable watershed management, sustainable agricultural practices and sustainable livestock management.

The payments could be made to an individual, household, community or organisation and can either be conditional on a specified environmental commitment, for example, on the fulfilment of an obligation to maintain a certain forest cover on land or paid in advance of the PES programme. We did not limit inclusion of these programmes by the funder/implementer (e.g., private vs. public) or status of land (private land or state‐owned/protected land). Finally, we included programmes that study PES alone or in combination with other intervention activities, for example, interventions supporting alternative livelihoods.

#### 5.1.3 Type of outcomes

We included studies that assess the impact of PES on either environmental, socioeconomic or intermediate outcomes, as defined below. PES programmes often have multiple objectives, related to both the preservation or restoration of environmental services and human welfare. There is a considerable literature on the potential trade‐offs or complementarities between these objectives. By looking at both sets of outcomes, we aimed to inform this debate.

We also included studies that assess intermediate outcomes such as changes in agricultural, forest or land management practices. This allowed us to report on effects at earlier stages of the PES causal chain.

##### 5.1.3.1 Intermediate outcomes

We included studies that assess changes in land or forest management practices, defined as measures of the type, frequency, intensity or adoption of such practices at the household or community level. We also included studies that assess the adoption of sustainable agricultural practices or technologies, for example, incorporating trees into agricultural or grazing lands. We also assessed measures of forest dependence, for example, resource extraction.

##### 5.1.3.2 Environmental outcomes

We included environmental outcomes that are related to GHG emissions or carbon storage/sequestration. This covered both direct measures of emissions (CO_2_, CH_4_, N_2_0) or carbon storage/sequestration and proxies for such outcomes. Based on previous mapping work in this area, we know that there are few evaluations that measure provision of environmental services such as carbon sequestration (Snilstveit et al., 2016). Proxy outcomes include deforestation rate, forest cover, forest condition/degradation, forest fires, soil quality, and so on. We accepted whichever measure was used by the study authors.

We also included outcomes related to the spillover effects of PES programmes on to land or forests not enroled in PES programmes.

##### 5.1.3.3 Socioeconomic outcomes

We included any measures of socioeconomic outcomes, including income, consumption, well‐being, livelihood security and assets of communities/households/individuals participating in PES programmes. We also included measures of food security across the four dimensions of food availability, access, utilisation and stability included in the Declaration on Food Security (FAO, 2009). These include food consumption, food expenditure, prevalence of undernourishment and nutritional status (FAO, 2013). We accepted whichever socioeconomic measure was used by the study authors.

#### 5.1.4 Types of study designs

We included studies in two stages, in a similar approach to Snilstveit et al. (2015). In the first stage, we included studies that assessed the effects of interventions using experimental designs or quasiexperimental designs with nonrandom assignment that allow for causal inference (to address primary research question 1). Specifically, we included the following:

▪ Studies where participants are randomly assigned to treatment and comparison group (experimental study designs).
▪ Studies where assignment to treatment and comparison group is based on other known allocation rules, including a threshold on a continuous variable (regression discontinuity designs) or exogenous geographical variation in the treatment allocation (natural experiments), where the assignment variable is not true random allocation (e.g., as determined by a random number table) by researchers involved in the study or intervention.
▪ Studies with nonrandom assignment to treatment and comparison group that include pre‐ and posttest measures of the outcome variables of interest to ensure equity between groups on the baseline measure, and that use appropriate methods to control for selection bias and confounding. Such methods include statistical matching (e.g., PSM, or covariate matching), regression adjustment (e.g., difference‐in‐differences (DIDs), fixed effects regression, single difference regression analysis, instrumental variables and “Heckman” selection models).
▪ Studies with nonrandom assignment to treatment and comparison group that include posttest measures of the outcome variables of interest only and attempt to use methods to control for selection bias and confounding, as above. This includes pipeline and cohort studies.

Ferraro and Miranda (2014, 2017) argue that combining panel data with baseline observations and statistical matching is the most effective quasiexperimental method at reducing bias when evaluating conservation sector programmes. However, given the expected small size of the evidence base, we included studies with postintervention outcome data only as long as they use some method to control for selection bias and confounding. To account for the differences in the quality of study designs and analysis methods, we appraised the risk of bias in all included studies.

Before‐after studies and observational studies without control for selection bias and confounding were excluded. Additionally, modelling based studies, commentaries and literature reviews were excluded.

To address questions 2 and 3 on programme design, implementation and context, we extracted descriptive and qualitative data from the included experimental and quasiexperimental studies. In addition, we conducted a targeted search for additional papers on the programmes covered by the included impact evaluations to provide additional detail on these areas. In order to be included, the papers had to be related to the programmes in the included impact evaluations and also be one or more of the following types of studies^5^:

▪ A qualitative study collecting primary data using qualitative or quantitative methods of data collection and analysis, and reporting some information on all of the following: the research question, procedures for collecting data, sampling and recruitment and at least two sample characteristics.
▪ A descriptive quantitative study collecting primary data using quantitative methods of data collection and descriptive quantitative analysis and report some information on all of the following: the research question, procedures for collecting data, sampling and recruitment, and at least two sample characteristics.
▪ A process evaluation assessing whether a programme is being implemented as intended and what is felt to be working more or less well, and why (HM Treasury, 2011). Process evaluations may include the collection of qualitative and quantitative data from different stakeholders to cover subjective issues, such as perceptions of intervention success or more objective issues, such as how an intervention was operationalised. They might also be used to collect organisational information.
▪ A project document providing information about planned, ongoing or completed programmes. They may describe the background and design of an intervention, or the resources available for a project for instance. As such, these documents do not typically include much analysis of primary evidence, but they provide factual information about interventions. The purpose of including them in our review is to ensure we had sufficient information about the context and interventions in included studies.

To address question 4 on cost‐effectiveness we included economic evaluations. We also used any economic evaluation or cost data provided in any of the studies included under the criteria above.

#### 5.1.5 Type of comparison

We included studies with a comparison group that received no intervention (including wait‐list comparisons), business as usual, or a different environmental intervention. Studies that only included a temporal (before‐after) comparison were excluded.

#### 5.1.6 Other criteria for including and excluding studies

We did not impose any restriction on inclusion of studies by language of publication or publication status. However, we undertook searches in English. We searched the literature back to 1990, excluding any studies published before this date. This date‐cut off is justified by both previous reviews of the literature, as well as the implementation of PES as a policy instrument for reducing deforestation. An evidence gap map covering PES interventions that searched back to 1990 did not identify any studies published before 2000 (Puri, Nath, Bhatia, & Glew, 2016). Moreover, PES was pioneered by Costa Rica as an approach to reducing deforestation in the late 1990s and REDD was first discussed at the UNFCCC conference of the parties in 2005 (UNFCCC, 2005). Thus, implementation and study of PES is unlikely to have taken place before 1990.

An overview of the inclusion criteria is provided in Table 1.

**Table 1:** Summary of inclusion criteria

Characteristics	Inclusion criteria
Population	Populations living in or near forests, wetlands, grasslands, mangroves and farmland areas in countries classified by the World Bank as low‐or‐middle income
Interventions	Payments for environmental services programmes
Comparisons	Comparison group that receives no intervention (including wait‐list comparisons), business as usual or a different environmental intervention
Outcomes	Intermediate, environmental and socioeconomic outcomes
Study design	To answer question 1, experimental and quasiexperimental studies
	To answer questions 2 and 3, qualitative studies, descriptive quantitative studies, process evaluations, project documents
Other	No inclusion restrictions by publication status or language

### 5.2 Search strategy: Studies to address review question 1

We implemented a systematic and comprehensive search strategy, developed in consultation with an information specialist, as outlined below.

#### 5.2.1 Electronic searches

We searched a range of databases and websites, including general sources of social science literature as well as sources specific to climate change, forestry, agriculture and impact evaluation. To reduce the potential for publication bias, this included both academic databases as well a range of specialist organisational websites and repositories of impact evaluations in international development. The sources covered by the search are listed below and a full record of the applied search terms is provided in Appendix 1. All searches were conducted in August–September 2017, as detailed in Appendix 4.

Bibliographic databases:

▪ CAB Abstracts: http://www.cabi.org/publishing‐products/online‐information‐resources/cab‐abstracts/
▪ Web of Science: http://wok.mimas.ac.uk/
▪ Greenfile (EBSCO): https://www.ebscohost.com/academic/greenfile
▪ Econlit: https://www.aeaweb.org/econlit/
▪ IDEAS/RePeC (EBSCO Discovery): https://www.ebscohost.com/discovery
▪ Agris (EBSCO Discovery): https://www.ebscohost.com/discovery

Specialist organisational databases:

▪ Centre for International Forestry Research (CIFOR): http://www.cifor.org/library/
▪ International Food Policy Research Institute Library (IFPRI): http://library.ifpri.info/discover/collections/
▪ International Institute for Environment and Development (IIED): http://pubs.iied.org/about/
▪ ATAI Research: https://www.atai‐research.org/emerging‐insights/?
▪ Global Environment Facility Evaluation Office: http://www.gefieo.org/evaluations/all?f[0]=field_ieo_grouping%3A312
▪ Conservation Evidence: http://www.conservationevidence.com/
▪ Climate Change Agriculture and Food Security (CCAFS) publications: https://ccafs.cgiar.org/publications
▪ Conservation International publications: http://www.conservation.org/publications/Pages/default.aspx
▪ IUCN Library: https://portals.iucn.org/library/dir/publications‐list
▪ Biodiversity International: http://www.bioversityinternational.org/e‐library/publications/
▪ AgEcon: https://ageconsearch.tind.io/?ln=en

Bilateral and multilateral agencies and general repositories of impact evaluations in international development:

- World Bank Open Knowledge Repository: https://openknowledge.worldbank.org/
- DFID Research for Development (R4D): http://r4d.dfid.gov.uk/
- Inter‐American Development Bank Publications: https://publications.iadb.org/facet‐view?locale‐attribute=en&field=type_view
- African Development Bank (AfDB): https://www.afdb.org/en/documents/publications/
- Asian Development Bank (ADB) https://www.adb.org/publications
- United Nations Development Programme (UNDP): http://www.undp.org/content/undp/en/home/library.html
- United National Environmental Programme: http://www.unep.org/publications/
- International Fund for Agricultural Development (IFAD): https://www.ifad.org/pub/overview
- Food and Agriculture Organisation of the United Nations (FAO): http://www.fao.org/publications/en/
- 3ie Repository of Impact Evaluations http://www.3ieimpact.org/en/evidence/impact‐evaluations/
- 3ie RIDIE (Registry for International Development Impact Evaluations): http://ridie.3ieimpact.org/
- Innovations for Poverty Action (IPA): http://www.poverty‐action.org/projectevaluations
- J‐Poverty Action Lab: https://www.povertyactionlab.org/evaluations

#### 5.2.2 Other searches

We screened the bibliography of existing systematic reviews, literature reviews and evidence gap maps for eligible studies, including the systematic review that this review will update and extend (Samii et al., 2014), and recent evidence gap maps (Puri et al., 2016; Snilstveit et al., 2016). We also screened the reference lists of included studies and undertook forward citation‐tracking for those studies using Google Scholar.

We contacted authors to identify additional studies.

### 5.3 Targeted search: Studies to address review questions 2, 3 and 4

After identifying our set of included impact evaluations, we undertook targeted searching for qualitative studies, process evaluation, project documents and economic evaluations for those interventions evaluated in the included studies. We conducted citation tracking of included studies to identify relevant sister papers and conduct internet and database searches using the names of programs from included studies. To identify project documents and process evaluations, we conducted targeted searches of databases of project documents and websites of implementing agencies. We also contacted authors and implementing agencies to request available project documentation.

### 5.4 Screening

We imported all search results into EPPI‐Reviewer 4 (version 4.7.1.0). Once duplicates were removed we screened citations against review inclusion criteria at title/abstract and full‐text. At the title/abstract screening stage, we used innovative text mining technologies to speed up the initial screening workload and test the potential for reductions in screening workload (O'Mara‐Eves, Thomas, McNaught, Miwa, & Ananiadou, 2015; Shemilt, Khan, Park, & Thomas, 2016). We used two functions in EPPI Reviewer to do this: the priority‐screening function and inclusion/exclusion classifier. We relied on the first option in the list below to include studies in the review, but compared the results of 2 and 3 retrospectively to assess reliability (results of this testing are report in full in Snilstveit et al., 2018):

1) Full independent double screening using the priority screening function to order results by probability of inclusion, based on a training set of screening.
2) Single screening using the priority screening function with a “safety first” approach (an option to mark unclear studies for review by a second screener) (Shemilt et al., 2016).
3) Single screening using the priority screening function combined with the use of the classifier function to auto‐exclude studies with a very low probability of inclusion.

The priority screening function can be used at the title/abstract screening stage to prioritise the items most likely to be “includes” based on previously included documents. This involved screening a random test set of at 700 citations to train the priority screening function, which then learned to identify relevant records based on keywords in the title and abstract of the included and excluded studies. Using priority screening in this way allows for the identification of includable records at an earlier stage in the review process so that work can begin earlier on full‐text screening and data extraction.

Independent double screening is typically considered the most reliable approach to screening in systematic reviews. However, this approach is also very resource intensive. In the “single screening with text mining” approach the machine effectively plays the role of the second screener. Moreover, before applying text mining all authors were allocated the same set of 100 randomly selected records for independent screening to establish interrater reliability, followed by a meeting to discuss any disagreements.

At the full‐text screening stage, all papers were double screened by two authors.

### 5.5 Data mapping

After completing the search and screening stage, we realised that a considerable number of the papers we identified evaluated the same programmes and outcomes, and that there appeared to be a number of cases where the same study was reported in multiple papers. We therefore undertook an additional stage to map the included papers by authors, programme, region of the evaluation and outcome before extracting data. This allowed us to get an overview of the scope and overlap of the evaluation work done to inform data extraction and the analysis.

### 5.6 Data extraction and coding procedures

We used a standardised data extraction form to extract data from included papers (the full data extraction form is included in Appendix 2). One person undertook the descriptive and effect size data extraction and it was checked by a senior a. We used a combination of Microsoft Excel and EPPI reviewer and extracted data on the following categories of information:

▪ Descriptive data on study design, intervention and context for purposes of descriptive analysis of the body of research.
▪ Data on the population, context, study design, intervention design, process and implementation and cost for purposes of moderator analysis and qualitative synthesis addressing questions 2 and 3.
▪ Data on the outcomes of interest and sample size for purposes of effect size calculation.

All data extraction for the qualitative synthesis was undertaken in EPPI reviewer.

### 5.7 Critical appraisal^6^

#### 5.7.1 Assessment of risk of bias in experimental and quasiexperimental studies

We undertook risk of bias assessments of each of the included impact evaluations using criteria as suggested by an adapted version of the Cochrane Risk of Bias Tool (Hombrados & Waddington, 2012). We assessed the risk of bias based on the following criteria, coding each paper as “Yes”, “No” and “Unclear” according to how well they address each domain:

1. Mechanism of assignment: Was the allocation or identification mechanism able to control for selection bias?
2. Group equivalence: Was the method of analysis executed adequately to ensure comparability of groups throughout the study and prevent confounding?
3. Performance bias: Was the process of being observed free from motivation bias?
4. Spill‐overs, cross‐overs and contamination: was the study adequately protected against spill‐overs, cross‐overs and contamination?^7^
5. Selective outcome reporting: Was the study free from selective outcome reporting?
6. Selective analysis reporting: Was the study free from selective analysis reporting?
7. Other risks of bias: Is the study free from other sources of bias?

Two authors undertook the risk of bias assessment independently for a sample of 20% of the studies, with disagreements resolved by a third author. The remaining 80% were assessed by one author but checked by a second author. We attempted to explore in the meta‐analysis if there are systematic differences between primary studies with different risk of bias but did not identify a sufficient number of studies for this analysis.

We used the results of the risk of bias assssments to produce an overall rating for each study as low, medium, high or critical risk of bias. We used the following decision rules to come to this decision. As selection bias is the most serious methodological issue affecting impact studies, and especially so in the field of PES where self‐selection is the norm, we give a greater weight to methodological weaknesses is this area, as well as group equivalence and spillovers.

- If all questions are answered “yes”, studies are assigned a low risk of bias rating.
- If studies score “yes” for selection, group equivalence and spillovers, but “no” or “unclear” for other domains studies are assigned a medium risk of bias rating. If they score “yes” for two out of three of the categories selection, group equivalence and spillovers, and unclear for another, we assign a medium risk of bias rating.
- If studies score “no” for any one of the following: selection, group equivalence or spillovers they are assigned a high‐risk of bias rating. For studies unclear on two or more of the three key categories (selection, group equivalence or spillovers) but that attempted matching/matching w. regression, we give a high‐risk of bias rating.
- If studies score “no” for more than one of the selection, group equivalence or spillover questions the study is assigned a critical risk of bias rating.
- Otherwise, we take an unclear rating as “no”.

#### 5.7.2 Assessment of trustworthiness in descriptive quantitative studies, qualitative studies and process evaluations

We assessed the trustworthiness of included qualitative studies, process evaluations and descriptive quantitative studies using an adapted version of the Critical Appraisal Skills Programme checklist (CASP, 2006) and Pluye et al. (2011) mixed‐methods appraisal tool. The developed tool makes judgements on the adequacy of reporting, data collection, presentation, analysis and conclusions drawn. The appraisal assessed the trustworthiness of the included qualitative studies and descriptive quantitative studies using six appraisal domains:

1. The defensibility of the applied research design to answer the research question under investigation.
2. The defensibility of the selected research sample and the process of selecting research participants.
3. The rigour of the technical research conduct, including the transparency of reporting.
4. The rigour of the applied analysis and credibility of study's claims given the nature of the presented data.
5. The consideration of the study's context (for qualitative studies only).
6. The reflexivity of the reported research (for qualitative studies only).

Each appraisal domain was assessed from a scale of low trustworthiness to medium, high and critical trustworthiness. An overall appraisal judgement per study was allocated using a numerical threshold of the appraised quality domains.

We did not undertake a critical appraisal of included project documents. They typically provide information about planned, ongoing or completed programmes, providing information about the design or resources available for a project for instance. As such these documents do not typically include much analysis of primary evidence, but they provide factual information about interventions. The purpose of including them in our review is to ensure we have sufficient information about the context and interventions included in our review. We therefore focused the appraisal on assessing the relevance of the documents against the interventions assessed in our review. Before extracting any data, we ensured that the name of the intervention, the implementing agency, context and timeline of the intervention described in the project document corresponds to the intervention assessed in the impact evaluation included in our review. Finally, collecting data from a range of sources, especially if used for triangulation, can enhance confidence in the trustworthiness of the information included (Montgomery et al., forthcoming). If several sources were available, we extracted data from all sources for purposes of triangulation. However, we took a saturation approach for the larger programmes such as Costa Rica where are larger number of qualitative documents were available.

### 5.8 Effect size calculation

Where possible we extracted the necessary data to calculate standardised effect sizes. For continuous outcomes, we calculated the Hedges' *g* sample‐size corrected SMDs, its variance and standard error using the following formula (Ellis, 2010):

$$g≅d\left(1-\frac{3}{4\left(n_{1}+n_{2}\right)-9}\right).$$

The decision as to which formula to use to calculate effect sizes was made taking into account what was reported in the majority of the studies sharing common outcomes. We used the most appropriate formulae for calculating effect sizes, considering the types of study designs we identify and the data they report. All but two of the studies were quasiexperimental designs with outcome measures reported either as regression coefficients (partial (adjusted) estimates) or mean differences following matching, with standard errors or *t* statistics and sample sizes. Typically, the studies did not report standard deviations.

We therefore used the following formulae below (Lipsey & Wilson, 2001).

For studies reporting regression coefficients and different sample sizes in treatment and control:

$$d=t\sqrt{\frac{1}{n_{t}}+\frac{1}{n_{c}}},$$

where *t* denotes the *t* statistic, either taken directly from the paper or calculated by dividing the regression coefficient by the standard error, *n* denotes the sample size of treatment group (*t*) and control (*c*).

For studies reporting regression coefficients and equal sample sizes in treatment and control (or where samples sizes for treatment and control were not presented separately):

$$\begin{array}{cc} d=\frac{2t}{\sqrt{n_{t}+n_{c}}} & Var_{d}=\frac{\;n_{T}+n_{C}}{n_{T}n_{C}}+\frac{d^{2}}{2(n_{T}+n_{C})} \end{array}.$$

We calculated the *t* statistic (*t*) by dividing the coefficient by the standard error. If the study did not report the standard error, but reported the *t* statistics, we extracted this and used as reported by the authors.

For studies reporting mean differences ($∆\overset{®}{X})$ between treatment (*T*) and control (*C*) and standard deviation (*SD*) at follow up (*p +* 1), we used the following:

$$d=\frac{{∆\bar{X}}_{p+1}}{{\mathrm{SD}}_{p+1}}=\frac{{\bar{X}}_{\mathrm{Tp}+1}-{\bar{X}}_{\mathrm{Cp}+1}}{{\mathrm{SD}}_{p+1}}.$$

Studies reporting mean differences between treatment and control, standard error (*SE*) and sample size (*n*):

$$d=\frac{{∆\bar{X}}_{p+1}}{\text{SE}\sqrt{n}}.$$

Studies reporting means and standard deviations for treatment and control groups at baseline (*p*) and follow up:

$$d=\frac{{∆\overset{®}{X}}_{p}-{∆\overset{®}{X}}_{p+1}}{{\mathrm{SD}}_{p+1}},\text{where}$$

$${\mathrm{SD}}_{p+1}=\sqrt{\frac{\left(n_{\mathrm{Tp}+1}-1\right){\mathrm{SD}}_{\mathrm{Tp}+1}^{2}+\left(n_{\mathrm{Cp}+1}-1\right){\mathrm{SD}}_{\mathrm{Cp}+1}^{2}}{n_{\mathrm{Tp}+1}+n_{\mathrm{Cp}+1}-2}}.$$

In cases in which significance levels were reported rather than the *t* statistics or standard errors (*b*), then we imputed *t* using the following in order to be able to make use of the most data possible:

$$\begin{array}{c} \text{Prob}\;>0.1:t=0.5\\ 0.1\geq \text{Prob}>\;0.05:t=\;1.8\\ 0.05\geq \text{Prob}>0.01:t=2.4\\ 0.01\geq \text{Prob}:t=2.8 \end{array}$$

Dependent effect sizes can arise when one study provides multiple results for the same outcome of interest or multiple studies use the same dataset and report on the same outcome. Dependent effect sizes are problematic because the traditional estimation of a mean effect size relied on the statistical assumption of independence of each included estimation of effect (Gleser & Olkin, 2007). We identified a large number of PES evaluations that reported multiple, dependent effect sizes and therefore this was an important issue to address. We used the rules laid out below for deciding on inclusion in meta‐analysis.

We only included one effect estimate per sample in a single meta‐analysis. We intended to use robust variance estimation (Hedges, Tipton, & Johnson, 2010; Tanner‐Smith & Tipton, 2014) in cases where we identified 10 or more effect sizes for the same meta‐analysis; however, we did not come across any of these cases.

When we identified several papers that reported on the same study, we used effect sizes from the most recent publication. Where several studies existed using the same data set or where multiple outcomes are reported from alternate specifications within the same study, we selected the study or specification which was most similar to other estimates for the same outcome type to enhance the potential for meta‐analysis. This discussed further in the results. Where different studies reported on the same programme but used different samples (e.g., from different regions) we included both estimates, treating them as independent samples.

Several studies provided estimates at several different time points. In such cases we identified the most common follow‐up period and included the follow up measures that matched this most closely in the meta‐analysis. Nevertheless, we extracted data and calculated effect sizes for all time points and report these in the review.

#### 5.8.1 Unit of analysis

We assessed if studies account for unit of analysis errors as part of risk of bias assessment, where the unit of the treatment is different to the unit of analysis (The Campbell Collaboration, 2014). There were a small number of cases where the the unit of analysis was at a lower level than the assignment unit. We noted these cases in our risk of bias assessment and while we aimed to correct them using standard formula, the information was not available to correct the issue.

#### 5.8.2 Missing or incomplete data

Several of the included studies did not provide sufficient data to calculate effect sizes. We contacted study authors when there was missing or incomplete data for calculating effect sizes, however in most cases we did not receive the missing data.^8^ In these cases, we report on the descriptive characteristics of the study but state that it was excluded from the meta‐analysis or reporting of effect sizes due to missing data. We were unable to use data from two studies (Hedge & Bull, 2011; Robalino et al., 2014).

### 5.9 Calculating cost estimates

We planned to calculate incremental costs by building a profile of inputs, resource use and costs for each included intervention, drawing on the Ingredients Method (Dhaliwal, Duflo, Glennerster, & Tulloch, 2012; McEwan, 2012) and the resource‐use data‐coding tool proposed by Shemilt et al. (2012). We extracted data on costs from the included impact evaluations and a range of additional sources including sister papers, as well process evaluations, economic evaluations and programme documents identified through the targeted searches.

Because of the limited availability of cost data, and the heterogeneity of estimates provided, we were unable to implement our planned strategy as described in detail in the protocol (Snilstveit et al., 2018). Instead we simply report the findings provided by the study authors in a table and discuss them in brief.

### 5.10 Methods of synthesis

#### 5.10.1 Review questions 1, 2 and 3: Statistical meta‐analysis and meta‐regression

We synthesised evidence on the effectiveness of PES programmes using meta‐analysis where possible. We used inverse‐variance weighted, random effects model due to heterogeneity in the included studies (Higgins & Green, 2011). Where there were too few studies, or included studies were too heterogeneous in terms of interventions or outcomes, we report on the individual effect estimates only. We decided to combine studies using meta‐analysis when we identified three or more effect sizes using a similar outcome construct and where the comparison group state was judged to be similar across the two, similar to the approach taken by Wilson, Weisburd, and McClure (2011). We will use the metafor package in R software to conduct the meta‐analysis (R Development Core Team, 2008; Viechtbauer, 2010). The information used to decide on the meta‐analysis was collected during the mapping process discussed in the previous section.

##### 5.10.1.1 Assessment of heterogeneity

We assessed the heterogeneity of effect sizes graphically using forest plots. We also assessed heterogeneity formally by calculating the *Q*‐statistic, *I* ^2^ and *τ* ^2^ to provide an overall estimate of the amount of variability in the distribution of the true effect sizes (Borenstein, Hedges, Higgins, & Rothstein, 2009).

##### 5.10.1.2 Moderator analyses

We aimed to conduct moderator analysis to explore heterogeneity in the included studies, using subgroup analysis to explore heterogeneity by different treatment subgroups. However, due the limited number of studies this was not feasible. Instead we conducted sensitivity analysis and explored reasons for heterogeneity in the qualitative synthesis, paying attention to the following potential moderators:

▪ Methodology: study design, risk of bias status
▪ Substantive variables: Intervention characteristics (length of programme exposure, size of transfer, type of condition, including whether the PES targets conservation, restoration of an environment or change to a different, more environmentally favourable land use, whether the PES scheme is government, NGO, multilateral/bilateral institution or user financed and whether it is a national level, regional or local programme),
▪ Context (region, country income level, tenure security),
▪ Participant characteristics (gender, socioeconomic status).

##### 5.10.1.3 Sensitivity analysis

We conducted sensitivity analysis to assess whether the results of the meta‐analysis were sensitive to the removal of any single study. We did this by removing studies from the meta‐analysis one‐by‐one and assessing for changes in results.

##### 5.10.1.4 Publication bias

We attempted to reduce publication bias by searching for and including unpublished studies in the review. We also tested for suggestion of publication bias by using funnel plots and Egger et al. (1997) test. Given the inherent subjectivity in assessing funnel plot asymmetry, we also assessed the sensitivity of meta‐analyses using “trim and fill” (Duval & Tweedie, 2000).

#### 5.10.2 Review questions 2 and 3: Qualitative synthesis

To address questions 2 and 3 we aimed to undertake a statistical meta‐regression to complement the qualitative synthesis, as discussed above (Rubenstein, Williams, Danz, & Shekelle, 2009). As discussed above, due to limitations in the number of studies included for each outcome we were only able to undertake meta‐regression at the review level for region and income level.

For the qualitative synthesis, we conducted a thematic synthesis on intervention design, implementation and contexts that mitigate or reinforce intervention effects (Thomas & Harden, 2008). The findings of qualitative research studies were synthesised in form of analytical themes configured around programme mechanisms, design, implementation and contexts in relation to research questions 2 and 3. We followed Thomas and Harden's (2008) suggested three‐stage approach to thematic synthesis of qualitative data.

In **stage one**, the reported research findings of the included qualitative studies were subject to inductive line‐by‐line coding. Research findings would ideally have referred to the primary data reported in each included study (e.g., interview excerpts), but due to limited reporting of this information, authors' analyses and conclusions represented study findings and the unit of analysis in the thematic synthesis. The line‐by‐line coding feature in EPPI‐reviewer was applied to guide and manage the inductive coding of the reported analyses and conclusions. Guidelines for thematic analysis, as applied in qualitative primary research, informed this process of generating inductive codes from the included studies.

In **stage two**, the identified inductive codes were then grouped into descriptive themes. In addition to the inductive creation of descriptive themes from studies' codes, a number of predefined (deductive) descriptive themes were introduced in the synthesis and controlled for during line‐by‐line coding. These deductive themes relate to areas of interest that are potentially under‐reported in the literature, for example, gendered effects. Only by introducing these deductive descriptive themes can we identify a possible absence of evidence on these themes, which would have not emerged in a purely inductive thematic synthesis. We used EPPI‐Reviewer's coding software to illustrate the link between the inductive codes in the primary studies and the identified descriptive themes.

In **stage three** of the thematic synthesis, we translated the descriptive themes into analytical themes. This translation is the key process in generating new data in the thematic synthesis. In the context of the review questions, analytical themes were formulated exclusively around mechanisms, design, implementation and contexts that can configure the effects of PES programmes in LMICs. We used EPPI‐Reviewer's coding software to illustrate the link between the descriptive themes and identified analytical themes.

#### 5.10.3 Question 4: Cost analysis

Costs and resource use are key considerations in the resource allocation choices of policymakers and practitioners. Cost analysis and economic evaluation can help inform decisions about the relative efficiency of environmental programmes (Shemilt et al., 2008; Shemilt, Valentine, Pössel, Mugford, & Wooldridge, 2012). There was insufficient data available to assess costs and resource use, and conduct cost‐effectiveness analysis. We therefore present the available cost data descriptively.

#### 5.10.4 Integrated synthesis

The overarching goal for the review was to provide an integrated synthesis of the findings from synthesis of review questions 1, 2, 3 and 4 in a narrative synthesis. We envisaged to use the programme theory provided above to present the findings from the different syntheses with the aim of providing an integrated narrative synthesis addressing the objectives of the review. However, because of the overall high‐risk of bias and lack of evidence we did not conduct such analysis. However, we summarise the findings and the strength of the underlying evidence base followed the GRADE approach (Schünemann et al., 2011) to facilitate the transparent and systematic presentation of our findings.

## 6 RESULTS

### 6.1 Description of studies

**Figure 2:**
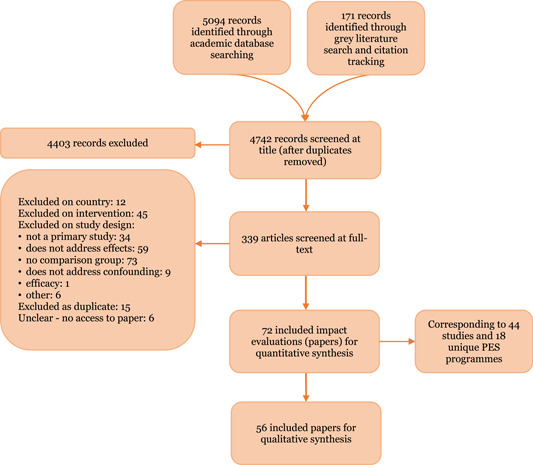
PRISMA diagram [Color figure can be viewed at wileyonlinelibrary.com]

Figure 2 presents the PRISMA diagram which describes the process of identifying studies for the review.^9^ We identified a total of 5,265 studies through the searching process. After removal of duplicates, we were left with 4,742 papers to screen at title and abstract. We discarded 4,303 records at this stage as they clearly did not relate to PES, they studied a HIC or the abstract clearly referred to the use of an ineligible study design. This left 339 studies to screen at full‐text.

At full‐text, the most common reason for exclusion from the review was that the study did not use a comparison group (*n* = 73), followed by the study not addressing effectiveness (*n* = 59) or not evaluating a PES intervention (*n* = 45). We excluded 12 papers for not looking at an LMIC, 34 papers for not being a primary study and nine for not addressing confounding factors in their analysis. We excluded 15 papers for being earlier versions of included papers but not presenting any new analysis. Finally, we were unable to get hold of full‐texts of six papers.

The final number of papers included for the quantitative synthesis was 72. These papers corresponded to 44 unique studies, covering 18 PES programmes. The full list of included papers is provided in Appendix 5. There is a significantly larger number of papers than studies as many studies are published in multiple papers, for example, as a journal article and as multiple earlier working papers that present other outcomes or more programme detail. In addition, the number of studies is much higher than programmes as we identified multiple studies that evaluated the same programme. This is discussed in more detail below under characteristics of programmes and studies.

After identifying the 18 included PES programmes, we undertook a targeted search for qualitative, descriptive quantitative, process evaluations and projects documents associated with those programmes in order to undertake a qualitative synthesis. We included 60 additional qualitative documents not counting the included impact evaluations themselves, which occasionally featured qualitative data and analysis too.

We first present the characteristics of the 18 PES programmes evaluated in the 44 studies, for example, setting, intervention design characteristics and objectives. This is followed by a description of the characteristics of the individual studies that evaluated these programmes, including the outcomes assessed and study design and analysis methods.

### 6.2 Characteristics of included programmes

#### 6.2.1 Setting

Table 2 presents the full table of characteristics of the included PES programmes. The 18 programmes took places in 12 countries covering several regions. Eight of the programmes took place in Latin America and the Caribbean. We identified evaluations of three different PES programmes from Mexico: the PSAH, the Monarch Butterfly Conservation Fund scheme and the Special Program for the Lacandon rainforest (Programa Especial de la Selva Lacandona [PESL]). We also identified evaluations of two PES programmes that had been evaluated in Costa Rica, the PSA and the Regional Integrated Silvopastoral Approaches to Ecosystem Management Project (RISEMP). In addition, we identified evaluations of PES programmes from Brazil (a REDD+ Pilot, se llama Projeto Assentamentos Sustentáveis Amazônia [PAS]), Columbia (also RISEMP) and Ecuador (Programa Socio Bosque).

**Table 2:** Table of characteristics—PES programmes

Programme	Country	Programme description	Ecosystem targeted	Programme objectives	Scale	Targeting approach	Start and end date	Impact evaluations
Projeto Assentamentos Sustentáveis na Amazônia	Brazil	Local‐level programme that targets conversation of forest land as well as the adoption of more sustainable land use techniques. Payments are conditional on the conservation of at least 50% of land as legal reserve, another 30% of the payment is conditional on the conservation of 15‐m wide forest riparian zones and the remaining 40% is conditional on the adoption of an environmentally sustainable production system. In addition, the programme is offering free administrative and technical support	Forests	Conservation; environmentally beneficial/preferable to BAU land‐use; socioeconomic (livelihoods, poverty reduction, etc.); other (support farmers to comply with law)	Local	Intervention target groups not clear	2012–2017	Simonet et al. (2017)
Bird Nest protection programme	Cambodia	A local‐level PES programme that rewards community members for the monitoring and protection of nests of specific endangered bird species. Payments are conditional on nest protection and chick survival	Forests	Conservation; restoration	Local	Not clear	2003 and ongoing	Beauchamp (2018) (associated papers: Clements (2012)—thesis and Clements et al. (2015)
Conservation Agreement	Cambodia	Local‐level programme that serves as an additional conservation incentives to an established protected area. Payments are conditional on a range of land‐use changes and conversation practices such as preventing slash‐and‐burn practices in pristine forest, as well as monitoring poaching and prohibiting logging for commercial purpose, but also participating in community patrolling. CI also required setting up committees at commune level, which are in charge of organising the distribution of incentives and patrolling	Forests	Conservation; other (community building and collective action)	Local	Priority target communes are selected following three sets of criteria relevant to local characteristics: the importance in terms of biological diversity, causes/intensity of deforestation threats and credibility of resources users as an conservation partner	2005–2012	Chervier (2017a) and (2017b)—paper on same intervention but with different data set on a different outcome
Sloping Land Conversion Program/Grain for Green Program	China	The regional‐level PES programme is one of the largest PES experiments in the world in terms of scale, payment and duration. Initiated in 1999, the programme aimed to increase vegetative cover over 32 million hectares by 2010, of which 14.7 million hectares would be converted from cropland on steep slopes back to forest and grassland. The programme is primary targeted at the reforestation of previously converted, mainly sloping land via compensation for changes in land‐use practices. Poverty alleviation objectives were referenced as secondary objectives at a later stage	Forests; grassland	Restoration; environmentally beneficial/preferable to BAU land‐use; socioeconomic (livelihoods, poverty reduction, etc.)	Regional	Slope is one of the main criteria by which land is selected. In practice, the central and the local governments bargain over the land conversion quota	1999 and ongoing	Duan (2015); Groom (2010); Liang (2012); Lin (2014); Lui (2013); Liu (2014); Liu (2018); Liu (2015); Uchida (2009); Xu (2010); Yao (2010)
Paddy Land‐to‐Dry Land program	China	A regional‐level land use conversion programme that aims to protect water quality and quantity. Payment is conditional on a conversion from rice to dryland cultivation essentially compensating upstream communities for providing ecosystem services valuable to downstream areas. Poverty alleviation objectives were referenced as secondary objectives at a later stage	Farmland	Conservation only; socioeconomic (livelihoods, poverty reduction, etc.)	Regional	Eligible areas decided by government. Eligibility criteria include areas with land‐use practices targeted for conservation	2006 and ongoing	Zheng et al. (2013)
Desertification Combating Program around Beijing and Tianjin	China	A local‐level PES programme that targets cropland conversion to reduce desertification and associated sandstorms. Payment is conditional on farmers planting trees on barren forestland of at least the area of their converted cropland. Other elements of the programme include irrigation projects; resettlement of rural households away from fragile ecological areas; and changing herding and animal husbandry practices to control overgrazing and rehabilitate degraded grassland	Forests; farmlands	Restoration; environmentally beneficial/preferable to BAU land‐use	Local	Eligible areas decided by government with household then being able to opt into the programme. Eligibility criteria include areas with land‐use practices targeted to change in order to avoid desertification	2001 and ongoing	Liu, Mullan, et al. (2014) (Associated papers Liu et al. (2013); Liu et al. (2018); Zhang and Liu (2005)
Regional Integrated Silvopastoral Ecosystem Management (RISEMP)	Colombia	Local‐level programme that targets the adoption of silvopastoral practices in degraded pastures, so as to generate increased biodiversity conservation and carbon sequestration. Payment is conditional on the adoption of a suite of more sustainable silvopastoral practices. Additional technical assistance to support the uptake of practices is provided	Farmland	Environmentally beneficial/preferable to BAU land‐use	Local	Both intervention target groups and targeting methods are unclear	2003–2007	Pagiola et al. (2016) (associated paper Pagiola et al., 2013)
Regional Integrated Silvopastoral Approaches to Ecosystem Management Project (RISEMP)	Costa Rica	A regional PES programme that was implemented in a total of three countries (Columbia, Nicaragua and Costa Rica) aiming to change silvopastoral practices in degraded systems. Payments were conditional on the adoption of a suite of more sustainable silvopastoral practices. Additional technical assistance to support the uptake of practices is provided	Farmland	Environmentally beneficial/preferable to BAU land‐use	Regional	Both intervention target groups and targeting methods are unclear	2002–2008	Garbach et al. (2012)
Programa de Pagos por Servicios Ambientales (PSA)	Costa Rica	The PSA offers different contracts to landholders for forest conservation, reforestation and/or sustainable forest management. Government makes direct payments to those landholders that comply with the contracts. Farmers are paid for the area (per hectare) of forest on land enroled in the programme (rather than directly for ecosystems services). Those with a contract for forest conservation need to fence off their land and post signs, prevent forest fires and hunting and not engage in agricultural activities or cutting down of trees for timber. Pre 2000, enrolment in the programme required landowners to have an official cadastral map of their land from the national registry, proof of ownership and an agreed forest management plan. In some areas, local NGOs faciliated the application process for signing up to PSA. Landholders may also receive technical assistance from local NGOs in implementation of forest management	Forests	Conservation; socioeconomic (livelihoods, poverty reduction, etc.)	National	Voluntary programme, first come, first served. Contracts could be established on properties of up to 300 hectares. In some areas, the local NGO implementers gave priority to areas based on areas that they percevied to be a higher risk of deforestation	Contracts signed between 1998 and 2004‐ongoing	Arriagada (2012); Arriagada et al. (2011, 2015); Robalino (2013, 2014, 2015); Sierra and Russman (2006)
Programa Socio Bosque	Ecuador	National‐level programme that targets the prevention of destruction and degradation of native ecosystems, as well as the increase of income and human capital in the poorest communities of Ecuador. The programme specifically targets ecosystems that are threatened, provide valuable environmental services such as regulation of hydrological systems, carbon storage and biodiversity; and are located in the poorest regions. Payment is conditional on a range of conversation related‐practices	Forests; other ecosystems	Conservation only socioeconomic (livelihoods, poverty reduction, etc.)	National	Eligible areas decided by government with communities being able to opt in. Eligibility criteria include (a) deforestation threat, (b) type of environmental services including: carbon storage, water cycle regulation, habitat for biodiversity and (c) poverty levels	2008 (general programme); 2009 specific area, ongoing	Jones et al. (2017); Hayes et al. (2017); Mohebalian (2016, 2018)
ICRAF PES experiment	Malawi	Local‐level pilot programme that targets afforestation of degraded areas. Payment is conditional on the number of surviving trees and additional technical assistance on forest management is provided	Forests	Restoration; environmentally beneficial/preferable to BAU land‐use; Socioeconomic (livelihoods, poverty reduction etc.)	Local	Landholders were identified in census with >1 ha of land and with clear land rights. Households reporting >1 acre of private land in the baseline survey were ineligible for contracting and were excluded from the randomisation	2008–2011	Jack and Santos (2017)
Mexico's PSAH	Mexico	National‐level PES programme that targets the conversation of forest cover. Poverty alleviation objectives were added at a later stage of the programme. Payments are conditional on the maintenance of forest functions as measured by forest cover. The programme grants 5‐year renewable contracts to both individual and communal landowners. Landowners may enrol a portion of their property and must maintain existing forest cover within the enroled parcel, but can make changes to land cover in other parts of their property. Verification of forest cover is made by satellite image analysis or ground visits. Landowners are removed from the programme if CONAFOR finds deforestation due to conversion to agriculture or pasture within the enroled area. Payments are reduced if forest is lost due to natural causes such as fire or pests	Forests	Conservation only; socioeconomic (livelihoods, poverty reduction, etc.)	National	Eligible areas decided by government with communities having to apply for inclusion. Eligibility criteria include areas targeted for conversation and with sufficient forest cover. Socioeconomic criteria (e.g., degree of marginalisation, female applicant, existing forest management plan) were adopted at a later stage	2003 and ongoing	Alix‐Garcia et al. (2015a; 2015); Arriagada et al. (2018); Le Velly et al. (2017); Scullion et al. (2011); Sims et al. (2017)
The Monarch Butterfly Conservation Fund	Mexico	A regional PES programme that combined designation of protected areas with PES to conserve over wintering habitat for the monarch butterfly. Payments are an incentive to abstain from felling timber and conditional on observed forest cover status	Forests	Conservation only	Regional	Groups needed to fall in area where butterfly takes habitat in winter, but no further information provided	2000 and ongoing	Honey‐Roses et al. (2011)
PESL (and PSAH)	Mexico	This local‐level programme includes a PES mechanism specifically designed to address local drivers of deforestation and forest degradation, among other incentives for sustainable use and rainforest conservation. Payment is conditional on the conversation of standing rainforest to ensure the provision of hydrological and biodiversity services	Forests	Conservation only (biodiversity conservation forest and hydrologic services)	Local	Eligible areas decided by government with communities required to apply for inclusion in the programme. Eligibility criteria include sufficient among of forest and clear property rights, among other	2005; 2008‐ongoing	Costedoat et al. (2015)
Nhambita PES‐project	Mozambique	A local‐level PES programme that targets reforestation and poverty alleviation. Payments are conditional on the planting and management of tress. Additional community development and capacity‐building initiatives are provided to strengthen the developmental objectives of the PES	Farmland	Restoration; socioeconomic (livelihoods, poverty reduction, etc.)	Local	Not clear why specific area for programme was chosen	2002 and ongoing	Hedge et al. (2011); Jindal et al. (2012)
Reducing Emissions from Deforestation and Forest Degradation (REDD+) Pilot	Nepal	A local‐level pilot PES programme that targets sustainable forest managment and poverty alleviation. The programme attempted to test the feasibility of the design of a PES programme that builds on existing community‐based forest management practices in Nepal including a strong equity focus and livelihood development objective. Instead of being conditional purely on forest carbon increments, pilot payments were based on weights assigned to the baseline carbon stock, annual carbon growth and social safeguard components. Additional capacity‐building and livelihood support activities were conducted	Forests	Environmentally beneficial/preferable to BAU land‐use; socioeconomic (livelihoods, poverty reduction, etc.)	Local	Not clear why specific area for programme was chosen	2011–2013	Sharma et al. (2015)
EPWS	Tanzania	A local‐level PES programme that incentives farmers to change current land use practices by planting trees and conservation farming so as to reduce forest products harvesting and reducing soil erosion so as to protect the flow and depth of water in the Mfizigo sub catchments. The programme explicityly combines conversation and poverty alleviation objectives to nuture sustainable natural resource management and improved livelihood security for the communities adjacent to the forest. It also includes a specific focus on equity in programme desing and ojectives	Forests; farmland	Conservation only; socioeconomic (livelihoods, poverty reduction, etc.)	Local	The programme was voluntary. A prerequisite for site selection was the livelihood status of farming communities, as they had to be at, or below, the poverty line	2006; 2008–2012	Kwayu (2017); Lokina (2016) (associated paper: John (2012)
PES experiment	Uganda	Regional‐level PES programme that targets the conversation of forestland. Payment is conditional on no not clearing tress with an additional option to participate in reforestation activities. Their first step when entering a community was to hold a parish‐level meeting for eligible PFOs to advertise and explain the programme. They then worked with interested PFOs to verify their forest land, measure its area and determine their eligibility. For those who signed up, an organisation monitored their land via spot checks and made annual payments to those who complied with the contract. The monitoring occurred through in‐person spot checks once every one or two months, during which the organisation employees checked for fresh tree stumps or other signs of cleared forest. PES enrollees also had the option to reforest up to two hectares of land. They were provided seedlings, and the PFO received 70,000 UGX per hectare per year if the seedlings survived	Forests	Conservation; restoration	Regional	Not clear why specific area for programme was chosen	2011–2013	Jayachandran et al. (2017) (associated paper: Jayachandran et al. (2011)

Abbreviations: BAU, business as usual; PES, payment for environmental service; PESL, Programa Especial de la Selva Lacandona; PFO, private forest owner; PSAH, Payments for Hydrological Services Program.

In addition, five of the programmes took place in the East Asia and Pacific region. We identified evaluations of three different PES programmes from China; the SLCP, also known as the Grain for Green Program (GFG), the Paddy Land‐to‐Dry Land (PLDL) program and the Desertification Combating Program around Beijing and Tianjin (DCBT). We also identified evaluations of two programmes from Cambodia, the Bird Nest protection programme and an intervention known only as the Conservation Agreement. We only identified one programme from South Asia, a REDD+ pilot that took place in Nepal. Finally, we identified four programmes from Sub‐Saharan Africa, which took place in Malawi (an experiment implemented by ICRAF), Mozambique (the Nhambita Community Carbon programme), Tanzania (Equitable Payment for Watershed Services [EPWS]) and Uganda (a PES experiment). We did not identify any evaluations of PES programmes from North Africa and the Middle East.

**Figure 3:**
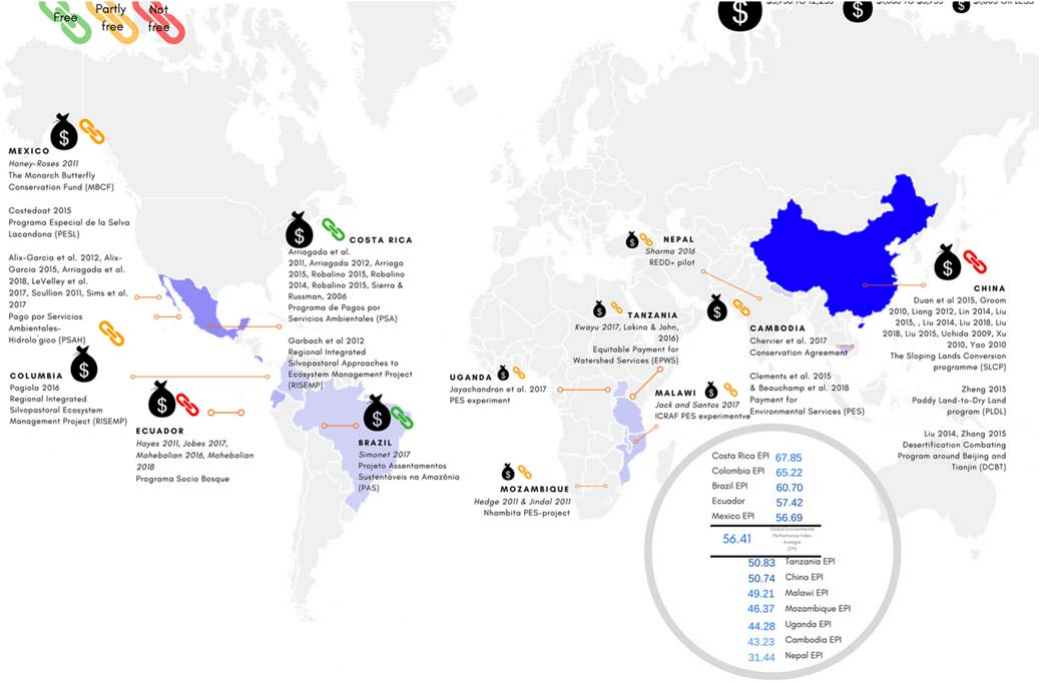
Programme settings [Color figure can be viewed at wileyonlinelibrary.com]

Figure 3 provides an overview of the setting in which the PES programmes were conducted. In terms of socioeconomic indicators, we applied the World Bank classification of economies^10^ to group programmes. The majority of our included programmes were conducted in countries classified as upper‐middle‐income countries (*n* = 11). In total five programmes were implemented in countries that were classified as low‐income countries. Just two programmes came from a lower‐middle income country. These geographical patterns were particularly driven by only six UMICs, which were responsible for 11 of 18 programmes alone, namely: China, Costa Rica, Mexico, Ecuador, Brazil and Colombia.

In terms of political indicators, we used the Freedom House Index to indicate the type of political regime contextualising the programmes reported in the included studies. The Freedom House Index was chosen as it is the most widely cited index assessing the condition of political rights and civil liberties around the world; the index has been calculated and reported consistently for over 40 years. The majority of programmes (*n* = 11) fell in the “partly free” country category having some restrictions on political freedoms. Four of the programmes were conducted in two countries rated as “not free”: Cambodia and China. Just three programmes, the large minority, were conducted in countries classified as “free”.

In order to zoom in to the environmental context in which the programmes were implemented, we used the Environmental Performance Index (EPI). Its global average is 56.41. We chose the EPI as an economic indicator as it is a comprehensive index that covers 180 countries ranking them according to 24 performance categories. The measures comprised within the index provide a national scale overview of the proximity between countries and achieving documented environmental policy goals. Regarding the EPI 10 programmes were conducted in countries below the average EPI, indicating that these programmes were applied in contexts with more acute environmental degradation. On the other hand, eight programmes were conducted in settings with above average EPI.

#### 6.2.2 PES programme design characteristics

We categorised the 18 programmes by a range of design characteristics, including the type of ecosystems targeted, the scale of the programme, the stated objectives and targeting approach. All of the included programmes met the basic criteria of a PES programme, meaning they provided payments to owners or managers of land, either households or communities, conditional on some minimum environmental/ecosystems service provision.

##### 6.2.2.1 Eco‐systems services targeted

The programmes targeted the restoration, conservation or improved management of several types of ecosystems with the payments. Ten of the programmes targeted forests only, specifically the REDD+ project in Brazil, the three programmes in Mexico, the REDD+ pilot in Nepal, the PES experiment in Uganda, the PSA programme in Costa Rica, the two programmes in Cambodia and the programmes in Malawi. The DCBT programme in China and the EPWS programme in Tanzania both targeted payments towards both forests and farmland. The Socio Bosque programme targeted payments towards forest and other native ecosystems. The SLCP programme in China targeted the restoration of both forests and grasslands. Three programmes targeted the improved management of farmland, specifically the PLDL programme in China, the RISEMP programme in Costa Rica and the Nhambita community carbon project in Mozambique. Finally, the RISEMP programme in Columbia targeted both farmland and grasslands.

##### 6.2.2.2 Scale

Ten of the PES projects worked at a local scale only, implemented in a small area of the country only: in Brazil, the two programmes in Cambodia, the DCBT in China, the RISEMP in Colombia, the ICRAF in Malawi, the PESL in Mexico, the Nhambita in Mozambique, the REDD+ pilot in Nepal and the EPWS in Tanzania. Five programmes worked at a regional level, covering whole regions of the country; specifically, the PES experiment in Uganda, the SLCP and PLDL programmes in China, the Monarch Butterfly Conservation Fund in Mexico and RISEMP in Costa Rica. The other three PES programmes in Costa Rica (PSA), Mexico (PSAH) and Ecuador (Socio Bosque) had national coverage.

##### 6.2.2.3 Programme objectives

As expected, all 18 of the programmes had at least one type of environmental objective. Eleven of the programmes targeted conservation, that is, the maintenance of existing forest cover (in Brazil, Ecuador, Tanzania, Uganda, the three Mexican programmes, the two Cambodian programmes, the PLDL in China, the PSA in Costa Rica). In addition, five targeted the restoration of lost forest or grassland (in Mozambique, Malawi, the SLCP and DCBT programmes in China and the Bird Nest Protection programme in Cambodia). Finally, seven also targeted change in land use to one more environmentally beneficial, but not necessarily the restoration of the former land use. This includes in Brazil, Colombia, Malawi, Nepal, RISEMP in Costa Rica and the SLCP and DCBT in China.

In addition, 10 of the identified programmes had an explicit objective of improving socioeconomic outcomes, for example, reducing poverty and supporting local livelihoods: in Brazil, Nepal, Mozambique, Tanzania, the Conservation Agreement in Cambodia, the SLCP and PLDL in China, the PSA in Costa Rica, Programa Socio Bosque in Ecuador, the ICRAF PES experiment in Malawi and the PSAH in Mexico. The Conservation Agreement in Cambodia was the only one that explicitly targeted community building and collective action in addition to environmental objectives.

##### 6.2.2.4 Complementary activities

The PES programmes provided varying amounts of support for the households or communities to meet the programme requirements. In the ICRAF experiment in Malawi (Jack & Santos, 2017) and the PES experiment in Uganda (Jayachandran et al., 2017), both of which targeted forest restoration, tree seedlings were provided at the beginning of the programme. In addition, the implementing organisation in Malawi provided trainings on tree planting and care to participants. In Uganda, parish‐level meetings were held for eligible forestry groups to advertise and explain the programme, which was followed by support to verify forest land, measure its area and determine eligibility. The REDD+ pilot in Brazil (Simonet et al., 2017) provided awareness meetings to support a better understanding of the Brazilian Forest Code and administrative support for the regularisation of land tenure through land registration and other administrative support for signing up to the PES programme. In some parts of Costa Rica, the PSA programme allowed local NGOs to facilitate the application process for signing up to PSA, for example, to provide the required official cadastral map of their land from the national registry, proof of ownership and a forest management plan (Arriagada et al., 2008, 2012). In the PSAH and PESL programmes in Mexico, participants can hire technical service providers to develop their application, as well as to design a forest management plan (Costedoat et al., 2015). In Mozambique in the Nhambita Community Carbon, farmers could participate in a training on the project requirements and the links between carbon storage and planting of trees (Jindal et al., 2012). Finally, the REDD+ Pilot in Nepal provided included activities such as forest carbon monitoring, awareness raising and capacity building for community forest management committees (Sharma et al., 2015).

Some of the programme combined payments with technical assistance for alternative or more sustainable livelihoods development.^11^ In Brazil, the REDD+ pilot provided technical support alongside payments for farmers to adopt environmentally sustainable production systems, for example, agroforestry and fish farming (Simonet et al., 2017). The DCBT programme in China provided support for changing herding and animal husbandry practices to control overgrazing and rehabilitate degraded grasslands (Liu et al., 2014). The evaluation also states that there was some resettlement of rural households away from fragile ecological areas (Liu et al., 2018). The RISEMP programmes in Costa Rica and Colombia included extension activities for farmers around silvopastoral practices, including education, outreach and demonstrations of how to best use plant materials (Garbach, 2012; Pagiola et al., 2016). The Nhambita project in Mozambique included fairly extensive alternative livelihoods alongside payments. It provided a range of forest related activities associated with community development including a carpentry unit, a bee keeping unit, a plant nursery and a demonstration garden, providing employment for 100 people (Jindal et al., 2012; Hedge & Bull, 2011). In Nepal, the REDD+ pilot provided capacity building activities to improve local livelihoods and to guide the participants to the use of alternative fuel and cooking technologies (Sharma et al., 2015). Finally, the Conservation Agreement programme in Cambodia (Chervier et al., 2017a) provided in‐kind support to communities involved in the programme such as salary for contractual teachers working in local schools or financial support for infrastructure and equipment in the community.

##### 6.2.2.5 Time period

Of the 18 included programmes, 10 are still in operation. These are the Bird Nest protection programme in Cambodia, the SLCP, DCBT and PLDL programmes in China, the PSA programme in Costa Rica, the Programa Socio Bosque in Ecuador, the PSAH, PESL and Monarch Butterfly Conservation Fund in Mexico, and the Nhambita PES‐project in Mozambique. The oldest programme in operation is the PSA in Costa Rica, which began to sign contracts with landowners in 1998. The SLCP in China began to work with landowner in some parts of the country in 1999. The rest of the programmes and pilots identified by the review have now finished, operating for between 2 years for the PES experiment in Uganda and 7 years the Conservation agreement in Cambodia.

### 6.3 Characteristics of included studies

An overview of the characteristics of the included studies is provided in Table 3 below.

**Table 3:** Table of characteristics—included studies

Included study	Country	Programme name	Included outcomes	Definitions of primary outcomes	Subgroups	Study design	Study analysis method	Sample Size
Hedge et al. (2011)	Mozambique	Nhambita PES‐project	Income/consumption/expenditure; intermediate outcomes	Expenditure per capita (MTS); cash income per capita (MTS); crop value (MTS); forest products (value‐MTS)	Woman headed households and poor households	CBA; method of analysis PSM		290
Jindal et al. (2011)	Mozambique	Nhambita PES‐project	Other socioeconomic outcome	Number of literates per household; number of m'shambas (plots) per household; household's annual cash income (MTN); households with access to wage labour in the village (%); household with at least one permanent job or a small business (%); asset ownership per household (number)	No	CBA; method of analysis DID	DID (simple t‐test)	334
Garbach et al. (2012)	Costa Rica	Regional Integrated Silvopastoral Approaches to Ecosystem Management Project (RISEMP)	Intermediate outcomes	Total number of silvopastoral practices adopted	No	RCT (random assignment to households/individuals)	OLS regression	124
Honey‐Roses (2011)	Mexico	The Monarch Butterfly Conservation Fund	Forest cover/deforestation	Avoided disturbance: percent conserved forest (>70% canopy cover) and hectares of forest cover; avoided deforestation: percent forest cover and hectares of forest cover	No	Spatial panel data with matched controls; method of analysis PSM		4,203 polygons
Beauchamp (2018) (associated papers: Clements (2015)	Cambodia	Bird Nest protection programme	Food security; other socioeconomic outcome	Rice surplus (kg) ; rice harvest (kg); education (whether a child is attending high school)	No	CBA	Matching with DID—post matching regression	596 247
Sharma et al. (2015)	Nepal	Reducing Emissions from Deforestation and Forest Degradation (REDD+) Pilot	Forest condition; carbon stocks; income/consumption/expenditure; other socioeconomic outcome	Observed in the sampled forest plots: forest fire signs; tree crown cover; shrub cover; grass cover; signs of wildlife; encroachment signs; timber extraction signs; firewood collection signs; open grazing signs; fodder collection signs; total forest carbon; gross income from CFUGs; household income from CFUG; backloads of total firewood collected by household annually; household with improved cooking stove installed for household cooking (have ICS) ; household with improved cooking stove installed for household cooking (have biogas); percentage share of firewood in household cooking; backloads of leaf‐litter collected by household annually; backloads of total fodder grass collected by household annually	No	CBA	PSM and DID	630; 277
Arriagada et al. (2011)	Costa Rica	Programa de Pagos por Servicios Ambientales	Forest cover/deforestation	Forest gain 1997–2005; forest loss 1997–2005; net deforestation 1997–2005	No	CBA	Various types of PSM matching	8188
Arriagada (2012) (associated papers: Arriagada (2008a)	Costa Rica	Programa de Pagos por Servicios Ambientales	Forest cover/deforestation	Change in forest cover on the farm between 1992 and 2005; self‐reported native forest cover change (ha); spillover effects—change in Self‐Reported Mature Native Forest Cover 1996–2005	No	CBA	Various types of PSM matching combined with DID/regression	202 197
Arriagada (2015)	Costa Rica	Programa de Pagos por Servicios Ambientales	Other socioeconomic outcome	Changes in cattle herd owned by the farmer; changes in hired labour; change in absentee status since 1996; Household Change in Asset Index; household change in asset count; family's quality of life	No	CBA	PSMatching + OLS regression	80
Robalino (2013)	Costa Rica	Programa de Pagos por Servicios Ambientales	Forest cover/deforestation	Deforestation (1997–2000)	No	CBA	Various types of PSM matching	10,108
Robalino (2014)	Costa Rica	Programa de Pagos por Servicios Ambientales	Other socioeconomic outcome	Poverty and extreme poverty	Type of slope; gender age, 35 or less, older than 35; distance to national roads	Panel data but no baseline OLS	Various types of PSM matching	18,425
Robalino (2015)	Costa Rica	Programa de Pagos por Servicios Ambientales	Forest cover/deforestation	Deforestation (2000–2005)—5 year effect (%)	No	Spatial panel data with matched controls; method of analysis PSM	Various types of PSM matching	10,944
Sierra and Russman (2006)	Costa Rica	Programa de Pagos por Servicios Ambientales	Forest cover/deforestation	Land use	No	Panel data but no baseline OLS	OLS regression	60
Alix‐Garcia et al. (2012)	Mexico	Pago por Servicios Ambientales‐Hidrolo´gico or PSAH	Forest cover/deforestation	The classification of deforestation in the Monitoreo is based on changes in NDVI values across years	No	RDD PSM with subsequent fixed effects regression		814
Alix‐Garcia et al. (2015a) (associated papers: Alix‐Garcia et al. (2015b)	Mexico	Pago por Servicios Ambientales‐Hidrolo´gico or PSAH	Forest cover/deforestation; other socioeconomic outcome; intermediate outcomes	NDVI; percent forest cover change (locality data); Poverty Index ; education investment; Food index; Durables index; Housing index; number of cattle; number of small animals; livestock infrastructure; agricultural inputs; agricultural equipment; quantity firewood collected; has large or small grazers; # Large grazers (such as cattle); participates livestock activity; quantity staples cultivated; produces staples	No	RDD PSM with subsequent fixed effects regression	Weighted, fixed effects regression	1,210; 21,769; 1,162; 1,401; 1,464
Arriagada et al. (2018)	Mexico	Pago por Servicios Ambientales‐Hidrolo´gico or PSAH	Income/consumption/expenditure; other socioeconomic outcome; intermediate outcomes	Proportion of households that earned more than the minimum wage from nonagricultural activities from 2007 to 2013; proportion of households that earned more than the minimum wage from agricultural activities from 2007 to 2013; difference in the proportion of households that processed goods from 2007 to 2013; difference in the number of household assets from 2007 to 2013; difference in household's asset index from 2007 to 2013; difference between ha of managed land in 2007 and 2013; difference in the proportion of households that owned livestock from 2007 to 2013; Cultural Services Number of Cultural Services mentioned by respondent; difference between ha of managed land for agriculture in 2007 and 2013.; Ecosystem Services Total Number of ES mentioned by respondent; Provisioning Services Number of Provisioning Services mentioned by respondent; Regulating Services Number of Regulating Services mentioned by respondent		CBA (comparison group with baseline and endline data collection)	Genetic matching + DID (OLS regression)	1,102;1,198; 1,190; 2,424
Le Velley et al. (2017)	Mexico	Pago por Servicios Ambientales‐Hidrologico or PSAH	Forest cover/deforestation	Forest loss within a polygon—2005–2012	No	CBA (comparison group with baseline and endline data collection)	PSM + OLS regression (and also weighted regression)	10,352
Scullion (2011)	Mexico	Pago por Servicios Ambientales‐Hidrologico or PSAH	Forest cover/deforestation	The outcome variable measured was the change in hectares of forest cover between time periods	No	Spatial panel data with matched controls Method of analysis PSM & DID		Not sure about the sample
Sims et al. (2017)	Mexico	Pago por Servicios Ambientales‐Hidrologico or PSAH	Forest cover/deforestation; other socioeconomic outcome	Net change in forest cover from 2000–2012; population growth; poverty alleviation; % without electricity; % without piped water; % without refrigerator; % with dirty floor; localities with a >5% share in PES; population growth Full Index, % population illiterate; % without primary school localities with a >5% share in PES	No	Panel data but no baseline Method of analysis Other regression		59,535
Duan et al. (2015)	China	SLCP	Income/consumption/expenditure	Family total income.; nonfarm employment income‐nonfarm employment; crop production income; forest income.	Income quantile 20%, 80%	Panel data but no baseline Method of analysis Quantile regression model, Tobit regression model and weighted least square model		375
Groom (2010)	China	SLCP	Other socioeconomic outcome	Househld off‐farm labour supply (194 days per household per annum)	No	CBA (comparison group with baseline and endline data collection) Method of analysis DID and switching regression	Switching regression + DID	286
Liang (2012) (associated papers: Li, 2011)	China	SLCP	Income/consumption/expenditure	Local wage‐income; migrating wage‐income; on‐farm income; total income	Income quantile 10%, 25%, 50%, 75%, and 90%	Panel data but no baseline Method of analysis Regression	DID OLS regression/Tobit regression multivariate linear regression + quantile regression	366 1,078
Lin (2014)	China	SLCP	Income/consumption/expenditure	Household income	No	Panel data Method of analysis maximum likelihood method	MLM regression	189; 200; 236; 269
Liu (2013)	China	SLCP	Income/consumption/expenditure	Average Quintile Immobility Rate; Average Quintile Move Rate	No	Panel data Method of analysis regression		3,375
Liu (2014)	China	SLCP	Income/consumption/expenditure	Land‐based income (RL); off‐farm income (RO); total income (R)	Stage of implementation	Panel data Method of analysis regression		3,375
Liu (2018)	China	SLCP	Intermediate outcomes	Tenure security; land reallocation	No	Panel data Method of analysis regression		300; 1,310
Liu (2018)	China	SLCP	Other socioeconomic outcome	Off‐farm labour time inputs (person‐days)	No	Panel data, but no baseline Method of analysis: PSM		1,158
Liu (2015)	China	SLCP	Income/consumption/expenditure	Household income diversity index	High medium‐ and low‐income	Panel data Method of analysis regression		1,458
Uchida (2009) (associated papers: Uchida, 2007)	China	SLCP	Income/consumption/expenditure; other socioeconomic outcome; intermediate outcomes	Off‐farm labour status change income per capita (yuan); crop income per capita (yuan); other agricultural income per capita (yuan); nonagricultural income per capita (yuan); value of house (yuan); fixed productive assets (yuan); livestock inventories (yuan); off‐farm work (number of adults with off‐farm work in household); migration status (number of adult migrants in household)	Income quantile	CBA (comparison group with baseline and endline data collection)	Matching + DID OLS regression	270 339
Xu (2010)	China	SLCP	Income/consumption/expenditure	Cropping before subsidy; other income; noncropping income; off‐farm income; total agricultural with subsidy; husbandry income	No	Panel data, but no baseline Method of analysis regression	Fixed effects regression for quantiles	360
Yao (2010)	China	SLCP	Income/consumption/expenditure; other socioeconomic outcome	Other income; total income; off‐farm income; animal husbandry income; crop production income; off‐farm employment	No	CBA (comparison group with baseline and endline data collection)	DID OLS regression	600
Kwayu (2017)	Tanzania	EPWS	Food security; other socioeconomic outcome	Food security; livestock ownership; ownership of consumer durables	No	Comparison group with endline data only PSM	PSM (nearest neighbour with replacement) +*t* tests to compare means	233
Lokina and John (2016) (associated paper: John, 2012)	Tanzania	EPWS	Other socioeconomic outcome; intermediate outcome	Perception of household on there welfare before and after 2008; perception of forest size	No	Comparison group with endline data only PSM	PSM with probit regression	200 189
Hayes (2011)	Ecuador	Programa Socio Bosque	Forest cover/deforestation	Household decision to stop grazing animals (cows and sheep) in the collective páramo	No	CBA (comparison group with baseline and endline data collection)	DID	399
Jones (2017)	Ecuador	Programa Socio Bosque	Forest cover/deforestation	Household level deforestation—change in deforestation rates	No	Spatial panel data with matched controls Method of analysis PSM	PSM (caliper matching with replacement) + fixed effects panel regression	513
Mohebalian (2016)	Ecuador	Programa Socio Bosque	Forest cover/deforestation	Deforestation between 2008 and 2014	No	Spatial panel data with matched controls Method of analysis PSM	PSM (one‐to‐one nearest neighbour match, without replacement) + comparison of means with *t* test	1,772
Mohebalian (2018)	Ecuador	Programa Socio Bosque	Forest cover/deforestation	Net effect on avoided deforestation (percent); avoided deforestation controlling for slippage; tree species richness (frequency); trees species at risk of extinction (frequency); tree species with commercial timber value (frequency)	No	Spatial panel data with matched controls Method of analysis PSM	PSM with *t* test of means	38; 536
Jayachandran et al. (2017) (associated document: Jayachandran et al., 2011)	Uganda	PES experiment	Forest cover/deforestation; food security; intermediate outcomes; other socioeconomic outcome;	Cut any trees in the past year; PFO‐level land circles: change in tree cover (ha); village boundaries: change in tree cover (ha); IHS of nonfood expend in past 30 days; IHS of food expend in past 30 days; allow others to gather firewood from own forest; increased patrolling of the forest in last 2 years; has any fence around land with natural forest. Programme impacts on tree‐planting: total trees survived; programme impacts on tree‐planting: total trees planted; programme impacts on tree‐planting: reforestation area; programme impacts on tree‐planting: took up reforestation option; tree cover‐spillovers/anticipation effects; child was sick with diarrhoea in last 30 days (age 0–5); child was sick with malaria in last 30 days (age 0–15); Has outstanding loan or repaid a loan in past year; nine‐step income ladder; IHS of alcohol/tobacco expend; claim to ownership of forest became stronger in last 2 years; have planted trees in the past year; had dispute with neighbours in last 2 years; decreased access to others who take trees from forest in last 2 years; any revenue from cut trees in the last year; IHS of total revenue from cut trees; total revenue from cut trees; cut trees for timber products; cut trees for emergency/lumpy expenses; cut trees to clear land for cultivation	No	RCT (random assignment to Households/individuals)		1,099
Pagiola (2016) (associated papers: Pagiola et al., 2013)	Columbia	Regional Integrated Silvopastoral Ecosystem Management Project	Forest cover/deforestation; intermediate outcomes	Change in ESI; proportion of farm changed % ESI per ha 2011‐follow up data from the above, post‐PES implementation (2007–2011)	No	CBA (comparison group with baseline and endline data collection)	DID + regression OLS regression	101 99
Chervier et al. (2017)	Cambodia	Conservation Agreement	Forest cover/deforestation; intermediate outcomes	The average yearly forest cover loss in ha in each grid square; perceived monetary‐related values from conserving the forest	No	CBA (comparison group with baseline and endline data collection)	PSM	325; 921; 841; 1,078
Zheng (2013)	China	Paddy Land‐to‐Dry Land programme	Income/consumption/expenditure; intermediate Outcome	Washing machine, refrigerator; television; motorcycle; liquefied petroleum gas; coal; wood; education; SLCP income; migrant income; nonfarm income; agricultural income; all income; seed expenditures; fertiliser expenditures; pesticide expenditures; P application; N application; estimated P export; estimated N export; agricultural intensification	No	CBA (comparison group with baseline and endline data collection)	DID with PSM	723
Jack and Santos (2017)	Malawi	ICRAF PES experiment	Income/consumption/expenditure; food security; other socioeconomic outcome	Total income from crop sales; per capita spending on food; casual labour income; months of food shortage; asset index; stated labour constraint; casual labour is a coping strategy; has acquired new land since 2008; total trees across all plots; No. of plots planted with trees; total plots cleared in last 3 years; has acquired new land since 2008	Lottery Auction	RCT (random assignment to households/individuals)	DID	319
Simonet et al. (2017)	Brazil	Projeto Assentamentos Sustentáveis na Amazônia (PAS)	Forest cover/deforestation; income/consumption/expenditure; other socioeconomic outcome	Forest cover as a share of total land area (hectares); wage salary; cattle ranching; total land as a share of total land area; cropland as a share of total land area; pastures as a share of total land area	No	CBA (comparison group with baseline and endline data collection)	DID with psmatching (nearest neighbour)	181
Liu (2014) (associated papers: Liu et al., 2018)	China	DCBT	Income/consumption/expenditure; other socioeconomic outcome	Land‐based income (RL); Off‐farm income (RO); total income R off‐farm labour time inputs (person‐days)	Stage of implementation	Panel data Method of analysis Fixed effects regression		3,375 1,158
Zhang (2015)	China	DCBT	Income/consumption/expenditure	Household per capital income	No	Panel data but no baseline Method of analysis Regression		188
Costedoat (2015)	Mexico	Unclear: seems like 2 programmes: PESL and the hydroligc federal one which is PASH	Forest cover/deforestation	Total forest cover in 2007 and 2013	No	CBA (comparison group with baseline and endline data collection)	Covariate matching DID	2,174

Abbreviations: CBA, comparison group with baseline and endline data collection; CFUG, Community Forest User Group; DCBT, Desertification Combating Program around Beijing and Tianjin; DID, difference‐in‐difference; EPWS, Equitable Payment for Watershed Services; ESI, environmental services index; MLM, multilevel modeling; NDVI, normalised difference vegetation index; OLS, ordinary least squares; RCT, randomised controlled trial; PES, payment for environmental service; PESL, Programa Especial de la Selva Lacandona; PFO, private forest owner; PSM, Propensity Score Matching; PSAH, Payments for Hydrological Services Program; SLCP, Sloping Land Conversion Program.

#### 6.3.1 Outcomes

We captured primary outcomes according to eight different categories namely: (a) forest cover/deforestation, (b) forest condition, (c) carbon stocks, (d) GHG emissions, (e) income/consumption/expenditure, (f) food security, (g) other socioeconomic outcome and (h) intermediate outcomes.

Of these eight outcomes, only six were reported in the included studies. Two outcomes—green gas emissions and forest condition—were not reported at all. The most frequently reported primary outcomes were “forest cover/deforestation” (*n* = 20), “other socioeconomic outcomes” (*n* = 18) and “income/consumption/expenditure” (*n* = 17). Food security was measured in four studies and, only a single study reported on carbon stocks.

In terms of outcomes measures, forest cover had been assessed using forest cover change. Similarly, deforestation had been measured as the change in deforestation rates. Other socioeconomic outcomes were measured quite heterogeneously with employment (*n* = 9) and assets (*n* = 8) being the most commonly reported socioeconomic outcomes. Intermediate outcomes have been reported in 19 of the included studies with agricultural behaviour dominating the outcome measures (*n* = 11). Table 4 below provides an overview of the outcomes assessed in the included studies.

**Table 4:** Overview of outcomes assessed

Outcomes assessed	# studies
1. Forest cover/deforestation	20
2. Other socioeconomic outcomes	18
3. Income/consumption/expenditure	17
4. Food security	4
5. Carbon stocks	1
6. Forest condition	0
7. Greenhouse gas emissions	0
8. Intermediate outcomes	19

##### 6.3.1.1 Subgroup outcomes

There were few studies that reported on the results of outcomes per subgroups. Of the 46 studies, only nine have conducted some form of subgroup analysis. Income related subgroups have been reported in five of the studies with gender and the stage of implementation each reported in two studies respectively. The remaining study looked at a subgroup focussed on the selection process for enrolment into the intervention.

#### 6.3.2 Study design and analysis methods

In terms of the study design, the most common type of studies followed a panel data design (*n* = 20). Of these, eight studies used panel data with no baseline. The remaining 12 studies using panel data could be grouped into two categories with six studies each: (a) spatial panel data with matched controls and (b) standard panel data. The second most frequent type of studies referred to comparison group with baseline and endline data collection (CBA) studies, a design used in 19 studies. Randomised controlled trials (RCTs) were conducted in three studies only. Comparison group with endline data only and regression discontinuity design were each utilised in two studies respectively. There was a large degree of heterogeneity in the conducted analysis methods. A range of different analysis methods were applied and often combined with each other. The most common analysis methods employed were PSM (*n* = 21), DID (*n* = 16) and ordinary least squares regression (*n* = 9).

### 6.4 Risk of bias

**Figure 4:**
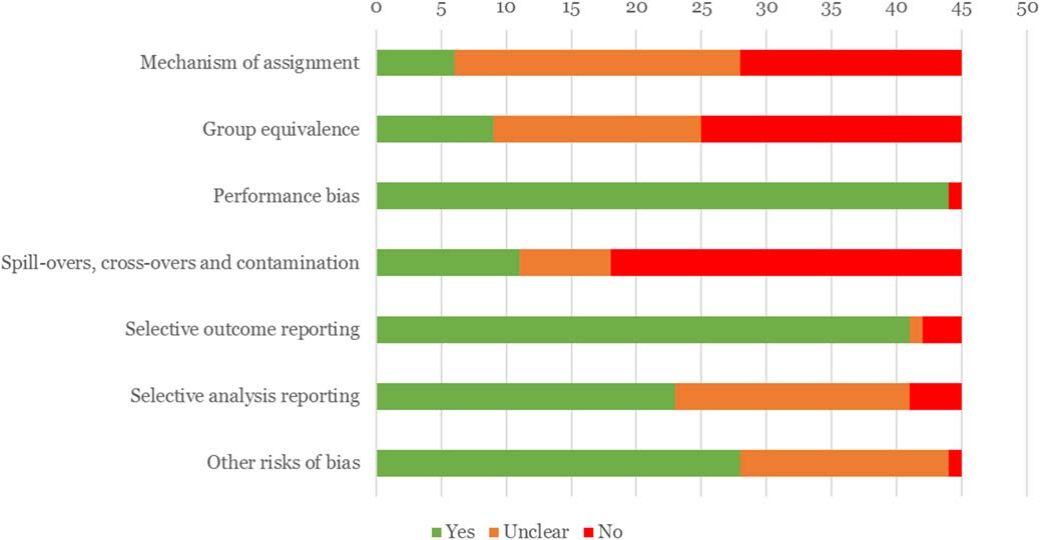
Summary of risk of bias across impact evaluations [Color figure can be viewed at wileyonlinelibrary.com]

Figure 4 presents a summary of the risk of bias assessments across the included impact evaluations. The full risk of bias assessments for each study can be found in Appendix 6.

Overall, the quality of the impact evaluation evidence base for PES is low. As described earlier, we assigned selection bias through the mechanism of assignment, group equivalence and spill overs, cross‐overs and contamination as the three most serious categories of bias for studies of PES in terms of their potential importance for influencing bias. Most PES programmes are voluntary and therefore there is self‐selection built in to the programme; however, most studies were unable to sufficiently address this in their design and analysis methods. Only 13% of the included studies sufficiently addressed selection bias, corresponding to two RCTs and four quasiexperimental studies. In these quasiexperimental studies, the authors had clearly investigated the process of selection into the programme and convincingly demonstrated how they could account for all relevant characteristics explaining participation and outcomes. We gave an unclear rating for selection bias to almost 50% of the studies. The rest of the studies clearly did not address selection bias. In addition, only 20% of studies adequately ensured their method lead to comparability of groups throughout the study and prevented confounding (group equivalence). In 36% of the cases, it was unclear if groups were comparable, and in almost 45% of studies they clearly did not ensure comparability of groups to overcome confounding.

A large majority of studies did not clearly address the potential for spillovers or contamination in PES programmes. This is despite the fact that spatial spillovers are likely to occur within PES programmes (Le Velley & Dutilly, 2016), including through within‐farm or land activity shifting resulting from only partially enroled land, spillovers on to nearby land or general equilibrium effects, for example, though a greater number off farm labourers in a local labour market. Therefore comparison groups and the unit of analysis needs to be chosen carefully or authors should demonstrate that they have investigated spillovers and concluded they were not an issue in their context. Only 25% of the studies clearly addressed spillovers, cross‐overs and contamination, with 15% unclear and 60% rated as not sufficiently addressing spillovers.

Almost all of the studies addressed performance bias or were not at risk of performance bias (*n* = 98%), that is, were able to create a process of being observed that was free from motivation bias, either from the use of administrative data or by taking steps in the collection of data to make it unlikely that being monitored could affect the performance of participants in treatment and comparison groups in different ways. We identified only one study, Garbach et al. (2012), that did not clearly address performance bias.

The vast majority of studies did not have selective outcome reporting within the paper (*n* = 91%), although this can be difficult to assess comprehensively without preanalysis plans. Over 50% of studies were free of selective analysis reporting. In 40%, the issue was unclear, while 9% were rated as selectively reporting analysis. These unclear and no ratings occurred mainly as the authors did not present any robustness tests to different specifications in their effects estimations or do not appear to use the most robust methods available to them.

In 62% of studies, no other risks of bias were identified, while in 35% it was unclear. Most of these cases were rated as unclear due to potential outcome measurement bias, including courtesy bias in reporting of changes in outcomes that were clearly linked to the programme.

Finally, 14 of the included studies used recall data to create baseline outcome and/or covariate data. While not necessarily a bias issue as we would not expect recall to be systematically different between the treatment and comparison groups, it may have increased error of the estimates when participants do not remember previous experiences or status accurately or neglect important details in their recall of an event. In most of the cases where recall data was used, the researchers asked the participants to recall information such as household income or agricultural behaviour over extended periods of time, in some cases more than 10 years.

**Figure 5:**
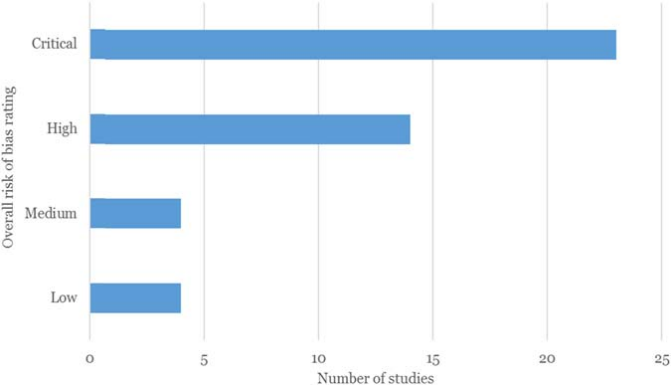
Summary of overall risk of bias ratings across impact evaluations [Color figure can be viewed at wileyonlinelibrary.com]

Figure 5 presents a summary of the overall risk of bias rating across the included impact evaluations, ranging from a low risk of bias rating up to a critical risk of bias rating. Fifty‐one percent of the included studies had sufficient methodological issues to be rated as suffering from a critical risk of bias. We rated 31% of the studies as high‐risk of bias and 9% as medium risk of bias. We rated just 9% of the studies as having a low risk of bias.

### 6.5 Data and analysis

The results of our synthesis are presented in three sections. We first present the results of the quantitative analysis, including meta‐analysis, relating to the effects of payment for environmental services on intermediate, socioeconomic economics and environmental outcomes. These are presented along the programme theory of change as presented in Section 1.2.1. All effect sizes are expressed as SMDs. To explain the findings of programmes with particularly large or negative results, we integrate some results of the descriptive and qualitative analysis in this section. In the following section, we present the results of the qualitative synthesis.

### 6.6 Quantitative synthesis

#### 6.6.1 Meta‐analysis decisions

We only included papers within the same meta‐analysis if they evaluated a similar outcome construct and the population samples did not, or where unlikely to, overlap. However, we identified many papers that evaluated the same programmes and, in some cases, also looked at the same outcome. In addition, there were many papers that presented various effect sizes for the same, or similar, outcomes. For these cases, we used the following rules to decide on inclusion in the meta‐analysis:

▪ If two or more papers evaluating the same programme assessed effects on the same or similar outcomes, we compared the regional coverage of the evaluation to determine depedence. If the papers evaluated the same programme in different regions, we included them in the meta‐analysis. However, if they evaluated the same outcome in the same region, we included the paper with the larger sample size. This mainly applied to the evaluations of the SLCP programme in China.
▪ If one paper presented multiple follow up periods for the same outcome, we chose the follow up period most similar to the other papers to be included in the meta‐analysis. In one case, the authors presented multiple effect sizes using different baseline points in the calculation of the effect size (Jones et al., 2017). As there was no most similar follow up point in this case, we chose the most conservative estimate of effects to include in the meta‐analysis.
▪ If one paper presented effect sizes for multiple similar outcome constructs, we chose the effect size most similar to the outcome constructs in the other papers to be included in the meta‐analysis.
▪ If one paper presented results for different variations of PES interventions, we chose the effect size for the intervention that was most similar to the interventions in the other papers to be included in the meta‐analysis.
▪ Several papers presented results for multiple mathching methods. In these cases, we extracted data and calculated effects for the nearest neighbour matching method, as this was the most commonly used matching method across the body of studies.
▪ In several papers, authors presented effect sizes for the same outcome using observed data and imputed data where data was missing. In those cases, we chose the effect size calculated using imputed data.
▪ For papers or data not included in the meta‐analysis due to dependency or outcome construct, we still calculated effects where possible. These are presented in the results alongside the meta‐analysis and in the appendices: Appendix 7 presents the full detail on all calculated meta‐analysis and sensitivity analyses (which is largely additional statistical information), while Appendix 8 presents an exhaustive list of all effect sizes not included in any of the meta‐analyses.

#### 6.6.2 Intermediate outcomes

We have results of the effects of PES programmes on intermediate outcomes for 15 of the 18 included programmes. This corresponds to 19 studies out of a total 44. Intermediate outcomes refer to outcomes that measures changes in agricultural or forest management behaviour and practices at the household or community level, including the adoption of sustainable agricultural practices or technologies. After mapping all the included studies, we grouped the intermediate outcomes into three groups of similar outcomes: (a) agricultural behaviour, (b) forest behaviour and (c) other intermediate outcomes. Unfortunately, we were unable to undertake meta‐analysis as the outcomes measured in the included studies were too diverse. This is despite being able to calculate 63 different effect sizes. The full tables of effect sizes for the intermediate outcome effects are reported in Appendix 8. We summarise the results narratively below.

##### 6.6.2.1 Intermediate outcomes (1): Effects of PES on agricultural behaviour

We identified nine studies that assessed the impact on PES on a measure of agricultural behaviour, from which we were able to calculate 30 effect sizes. These measures were too heterogeneous for meta‐analysis and therefore we report them narratively, grouped by similar outcomes. These studies came from China (Zheng et al., 2013), Nepal (Sharma et al., 2015), Brazil (Simonet et al., 2017), Costa Rica (Arriagada et al., 2015, Sierra & Russman, 2006), Mexico (Alix‐Garcia et al., 2015b), Colombia (Pagiola et al., 2013), Ecuador (Hayes et al., 2017) and Malawi (Jack & Santos, 2017).

###### 6.6.2.1.1 Agricultural inputs

Several studies assess the effect of PES on investment or use of agricultural inputs. Zheng et al. (2013) report the effects of the PLDL programme in China on three measures of agricultural input behaviour. They find a positive effect of the programme on phosphorus application (kg/mu) of 0.16 SMD (95% CI [0.01, 0.31]) and a fairly large negative effect on agricultural intensification (person‐days/mu) of 0.50 SMD (95% CI [−0.65, −0.35]). They find a statistically insignificant effect on nitrogen application, kg/mu (SMD = 0.08, 95% CI [−0.06, 0.23]). Alix‐Garcia et al. (2015b) report the effects of the PSAH in Mexico in agricultural inputs and agricultural equipment, broken down by PES contracts under private property and common property. For agricultural inputs in private property, they find a statistically insignificant effect of 0.20 SMD (95% CI [−0.06, 0.46]), and for agricultural equipment, a statistically insignificant effect of 0.09 SMD (95% CI [−0.17, 0.35]). For common property PES, they find no effect on agricultural inputs (SMD = −0.01, 95% CI [−0.10, 0.08]) and a statistically insignificant effect on agricultural equipment (SMD = −0.04, 95% CI [−0.13, 0.05]).

###### 6.6.2.1.2 Livestock ownership and investment

Several studies assess the effects of PES on the ownership or investment in livestock. Sharma et al. (2015) report on the effects of the REDD+ Pilot in Nepal on open grazing signs in forest plots, finding an insignificant effect (SMD = 0.07, 95% CI [−0.10, 0.23]). Alix‐Garcia et al. (2015b) report on the effects of the PSAH in Mexico on several livestock outcomes, finding positive effects on households that own small or large grazers (SMD = 0.08, 95% CI [−0.02, 0.18]), the number of large grazers (cattle) owned (SMD = 0.11, 95% CI [0.01, 0.21]) and whether a household participates in livestock activities (SMD = 0.10, 95% CI [−0.01, 0.20]). Alix‐Garcia et al. (2015b) also break the results down by PES contracts for private property and common property. For private properties, they find insignificant results of the PSAH on number of cattle (SMD = 0.08, 95% CI [−0.18, 0.34] and no impact on number of small animals (SMD = 0.01, 95% CI [−0.25, 0.27]). In contrast for common property, they find a positive effect on the number of cattle (SMD = 0.11, 95% CI [0.02, 0.21]) and a negative effect on the number of small animals (SMD = −0.32, 95% CI [−0.41, −0.23]). Finally, they report an insignificant effect of PSAH on livestock infrastructure in private properties (SMD = 0.17, 95% CI [−0.09, 0.42]) and an insignificant effect in common properties (SMD = 0.05, CI 95% [−0.04, 0.14]). In Ecuador, Hayes et al. (2017) find a negative effect of the Socio Bosque PES programme on household decision to graze animals (cows and sheep) in the collective areas of −0.17 SMD, (95% CI [−0.31, −0.03]). In Brazil, Simonet et al. (2017) report on the effect of the PAS programme on cattle ranching, as measured by the ratio of the value of total livestock owned to pasture in 2014, expressed in Reais per hectare, finding an insignificant effect of 0.14 SMD (95% CI [−0.16, 0.43]). In Costa Rica, Arriagada et al. (2015) find a large negative effect on the number of cattle owned between 1996 and 2005 of −0.96 SMD (95% CI [−1.42, −0.50]).

###### 6.6.2.1.3 Land use

Several studies report the effects of PES on indicators of the use of land for agriculture. In Colombia, Pagiola et al. (2013) find that the Regional Integrated Silvopastoral Ecosystem Management (RISEMP) programme had a positive effect on the proportion of farm changed to another land use of 0.52 SMD (95% CI [0.08, 0.96]) and area of farm land changed to another land use of 0.42 SMD (95% CI [−0.02, 0.85]). In Costa Rica, Sierra and Russman (2006) find that the PSA programme had a large positive effect on the area under scrubland (charral) of 0.73SMD (95% CI [0.21, 1.26]), but a negative although statistically insignificant effect on area under agriculture of −0.39 (95% CI [−0.90, 0.12]). In Brazil, Simonet et al. (2017) find an insignificant effect of the PAS on crop land of −0.02 SMD (95% CI [−0.27, 0.32]). Alix‐Garcia et al. (2015a) find a negative effect of the PSAH in Mexico on both quantity of staples cultivated including beans and maize (SMD = −0.13, 95% CI [−0.24, −0.03]) and households that cultivate staples (SMD = −0.15, 95% CI [−0.25, −0.04]).

###### 6.6.2.1.4 Land ownership

Simonet et al. (2017) report on effects of the PAS in Brazil on the total land of farmers, finding no effect (SMD = −0.01, 95% CI [−0.30, 0.29]). Jack and Santos (2017) present results for two intervention groups in the Malawi PES experiment, a group that received the PES programme after participating in a lottery and a group that participated in an auction, on new land acquired since 2008. For both groups, they find a statistically insignificant negative effect on new land acquired (for the lottery group, SMD = −0.12, 95% CI [−0.35, 0.11], and for the auction group, SMD = −0.19, 95% CI [−0.41, 0.04]).

##### 6.6.2.2 Intermediate outcomes (2): Effects of PES on Forest Behaviour

We identified four studies that assessed the impact on PES on a measure of forest behaviour, from which we were able to calculate 27 effect sizes. These measures were too heterogeneous for meta‐analysis and therefore we report them narratively, grouped by similar outcomes. These studies come from Uganda (Jayachandran et al., 2016, 2017), Mexico (Alix‐Garcia et al., 2015b), Nepal (Sharma et al., 2015) and Malawi (Jack & Santos, 2017).

###### 6.6.2.2.1 Forest clearing behaviour

Several papers report on household collection of firewood following PES. In Nepal, Sharma et al. (2015) report on the effects of the REDD+ pilot, finding insignificant positive effects on firewood collection signs observed in the sampled forest plots (SMD = 0.15 95% CI [−0.01, 0.32]) and fodder collection signs observed in the sampled forest plots (SMD = 0.09, 95% CI [−0.08, 0.25]). In Uganda, Jayachandran et al. (2017) report the effects of the PES experiment on whether households allowed others to gather firewood from their own forest, finding a negative effect of −0.36 (95% CI [−0.49, −0.23]). They find an insignificant effect on decreasing access to others who take trees from forest in last 2 years (SMD = 0.08, 95% CI [−0.04, 0.21]). Finally, in Mexico, Alix‐Garcia et al. (2015b) find a positive effect of the PSAH on firewood collection (SMD = 0.13, 95% CI [0.02, 0.25]).

Sharma et al. (2015) also report on the effects of the REDD+ pilot in Nepal on timber extraction signs observed in the sampled forest plots, finding a negative effect of –0.17 SMD (95% CI [−0.34, −0.01]). In Uganda, Jayachandran et al. (2016, 2017) also report on the effects of the PES experiment on various forest extraction measures. They find a negative effect on cutting of trees in the past year of −0.30 SMD (95% CI [−0.43, −0.18]) and a negative effect of cutting trees for timber products of −0.23 SMD (95% CI [−0.35, −0.10]). They also find a negative effect on cutting of trees for emergencies of −0.15 SMD (95% CI [−0.28, −0.03]). However; they find an increase in cutting of trees to clear land for cultivation of 0.14 SMD (95% CI [0.02, 0.27]). In Malawi, Jack and Santos (2017) present results for two intervention groups in the PES experiment, a group that received the PES programme after participating in a lottery and a group that participated in an auction, on clearing of land in the last 3 years and total plots cleared in the last 3 years. For the lottery group, they find a positive effect of PES on land clearing of 0.28 SMD (95% CI [0.05, 0.51]) and a positive effect on total plots cleared of 0.26 SMD (95% CI [0.03, 0.49]). For the auction group, they find similar positive effects on land clearing of 0.29 SMD (95% CI [0.06, 0.52]) and total plots cleared of 0.24 SMD (95% CI [0.01, 0.0.47]).

###### 6.6.2.2.2 Reforestation behaviour

In Uganda, Jayachandran et al. (2016) report the effects of the PES experiment on whether households took up reforestation option and number of trees planted, finding a fairly large positive effect on both (respectively, SMD = 0.50, 95% CI [0.38, 0.62] and SMD = 0.53, 95% CI [0.41, 0.65]). They also find a positive effect on planting trees in the past 12 months of 0.25 SMD (95% CI [0.16, 0.34]).

In Malawi, Jack and Santos (2017) present results for two intervention groups in the PES experiment, a group that received the PES programme after participating in a lottery and a group that participated in an auction, on the number of plots planted with trees and the total number of trees across plots. For the lottery group, they find a positive effect of 0.23 SMD (95% CI [0.00, 0.46]) on the number of plots planted with trees and a statistically insignificant positive effect on total number of trees of 0.15 SMD (95% CI [−0.08, 0.38]). For the auction group, they find statistically insignificant effects on the two outcomes (respectively, SMD = 0.07, 95% CI [−0.16, 0.30] and SMD = −0.05, 95% CI [−0.28, 0.18]).

###### 6.6.2.2.3 Forest protection behaviour

Sharma et al. (2015) assess the effects of the REDD+ pilot in Nepal on two other behavioural measures around forest protection. They find a negative effect of –0.21 SMD (95% CI [−0.38, −0.05]) on encroachment signs observed in the sampled forest plots and a negative effect of –0.21 SMD (95% CI [−0.38, −0.05]) on forest fire signs observed in the sampled forest plots. In Uganda, Jayachandran et al. (2017) find that the PES experiment increased patrolling of the forest in last 2 years by 0.15 SMD (95% CI [0.03, 0.28]). They find no effect on fences around land with natural forest (SMD = 0.01, 95% CI [−0.11, 0.14]).

###### 6.6.2.2.4 Property rights

Just one study looked the effect of PES on property rights, the PES experiments in Uganda (Jayachandran et al., 2016). They find a positive effect on claims to ownership of forest becoming stronger in the last 2 years of 0.09 SMD (95% CI [−0.03, 0.22]). They find an insignificant effect on disputes with neighbours regarding land in the last 2 years (SMD = −0.06, 95% CI [−0.18, 0.07]).

##### 6.6.2.3 Intermediate outcomes (3): Effects of PES on other intermediate outcomes

We identified three studies that assess the effects of PES participation on a measure of migration, from which we were able to calculate six effect sizes. These studies came from China (Demurger et al., 2012; Uchida et al., 2007) and Costa Rica (Arriagada et al., 2015). In China, Demurger et al., 2012) assess the effects of the SLCP on decisions around rural labour migration, finding a positive effect on migration of 0.34 SMD (95% CI [0.28, 0.40]). Uchida et al. (2007) find an insignificant effect on the number of migrants in a household of 0.07 SMD (95% CI [−0.17, 0.32]). In Costa Rica, Arriagada et al. (2015) report on four measures of changes in migration status, although these are all statistically insignificant. For change in absentee status since 1996 from living off‐farm for work to on‐farm, they find a negative effect of −0.26 SMD (95% CI [−0.70, 0.18]).

#### 6.6.3 Socioeconomic outcomes

We have results of the effects of PES programme on socioeconomic outcomes for 12 of the 18 included programmes, corresponding to 28 out of a total 44 studies. The large number of studies in comparison to programmes reflects the large number of studies that evaluate the impact of the SLCP programme in China on socioeconomic outcomes. We began by undertaking a meta‐analysis across household socioeconomic outcomes to get an initial idea of the effect of PES programmes on this set of outcomes. This includes household income, assets, expenditure and other measures of household economic status where available. Given that we would expect different effects for nonagricultural and agricultural income measures, we decided not to include these measures in this analysis; instead, we include measure of total household income. However, as this meta‐analysis combines a diverse set of outcome variables that may not be comparable and we therefore also undertake meta‐analysis for four more homogeneous sets of socioeconomic outcomes: (a) total household income, (b) household income from agricultural sources, (c) household income from nonagricultural sources and (d) household assets. We also calculated effect sizes for a number of other socioeconomic outcomes but were unable to undertake meta‐analysis due to the diversity of the types of outcomes measured. This includes results for employment, education, food security, poverty and perceived welfare. These findings are presented narratively.

##### 6.6.3.1 Socioeconomic outcomes (1): Effects of PES on household socioeconomic outcomes

Fourteen studies provided outcome data for the initial meta‐analysis on household socioeconomic outcomes, corresponding to 10 different PES programmes. Seven of these studies covered the three programmes in China, while the others covered the PSA in Costa Rica, the PSAH in Mexico, the ICRAF trial in Malawi, the Bird Nest Protection programme in Cambodia and the EPWS in Tanzania, the N'Hambita community carbon project in Mozambique and the PES RCT in Uganda.

**Figure 6:**
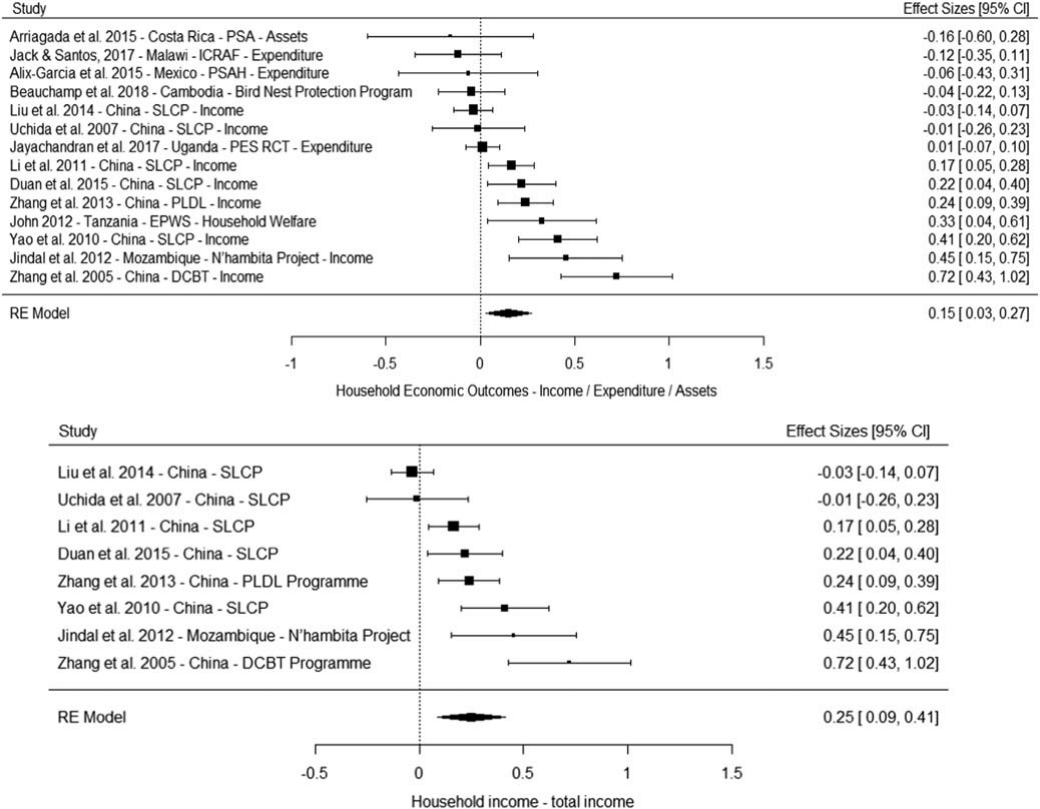
Effects of payment for environmental service on household socioeconomic outcomes

The average effect of these programmes on household socioeconomic outcomes is 0.15 SMD, 95% CI [0.03, 0.27]), calculated under a random effect model (Figure 6). The forest plot in Figure 6 suggests a substantial amount of variability between studies, and this is also suggested by the statistical heterogeneity tests (*I* ^2^ = 84.02%, 0.0406, *Q*(df = 13) = 58.8360, *p* < .0001). The effects range from −0.16 SMD (95% CI [−0.60, 0.28]) for the effect of the PSA in Costa Rica on household assets to 0.72 SMD (95% CI [0.43, 1.02]) for the effect of the DCBT in China on total household income.

$$(I^{2}=84.02\%,\;\tau^{2}=0.0406,\;Q(df=13)=58.8360,\;p\;<\;.0001).$$

##### 6.6.3.2 Socioeconomic outcomes (2): Effects of PES on total household income

Eight studies provided outcome data on overall household income for inclusion in the meta‐analysis, with six of these studies covering programmes in China. Five different studies evaluated the SLCP in China, covering different geographical locations. In addition, there were one study each of the PLDL and DCBT programmes respectively. Finally, one study assessed the effect of the N'hambita community carbon project in Mozambique.

**Figure 7:**
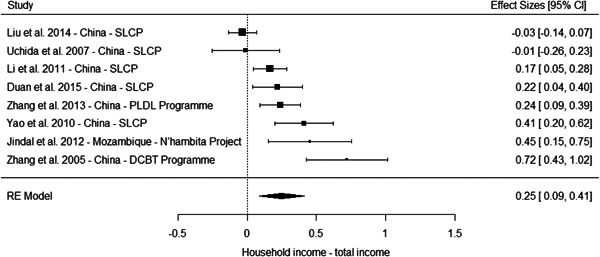
Effects of payment for environmental service on total household income

The average effect of these programmes on household income is 0.25 SMD, 95% CI [0.09, 0.41], calculated under a random‐effect model (Figure 7). The assessment of homogeneity suggest there is a large amount of variability between the studies (*I* ^2^ = 85.51%, *τ* ^2^ = 0.0439, *Q*(df = 7) = 40.366, *p* ≤ .0001). This is also evident when inspecting the forest plot in Figure 7, highlighting the wide range in effects, from SMD −0.03 [−0.14, 0.07] to 0.72 [0.43, 1.02]. The size and precision of the average effect is particularly sensitive to the removal of the Zheng et al. (2005), which reduces the average effect to 0.18 SMD [0.06, 0.32], although the CIs still do not cross the line of no effect. In addition, the removal of Liu et al. (2014) causes the average effect to increase to 0.29 SMD [0.14 0.45].

 

$\left(I^{2}=85.51\%,\;\tau^{2}=0.0439,\;Q(df=7)=40.366,\;p\;<\;.0001\right)$

We were able to calculate eight additional effect sizes from China for total household income that we were unable to include in the meta‐analysis due to dependencies with the other included studies from China.^12^ Liu et al. (2014) report an additional effect size for the effect of the SLCP programme combined with a non‐PES conservation programme to prevent logging and other harmful activities, the Natural Forest Protection Program (NFPP). They find an effect of 0.04 SMD (95% CI [−0.03, 0.11]). They also report results for the DCBT programme in China, finding a negative effect on total household income of −0.16 SMD (95% CI [−0.23, −0.08]). This finding is in contrast to Zhang et al. (2005), included in the meta‐analysis, who find a large impact of the DCBT on total household income. In addition, Liu et al. (2013) assess the effect of the SLCP on the average quintile move rate, that is, the average proportion of rural households that have the same income at *t* period after the initial income and the weighted average of transition probability, where the weight is the shift between different groups. They find a decrease in the proportion of rural households that have the same income after the initial period of −0.48 SMD (95% CI [−0.68, −0.27]) and an increase in households transitioning between different income groups of 0.43 SMD (95% CI [0.23, 0.63]), that is, more income mobility.

Liang et al. (2012) report the effects of the SLCP in China on local wage income for households with adults and the elderly, households with only adults, households with only adults and children and households with all three. They find effects of 0.09 SMD (95% CI [−0.26, 0.44]), 0.05 SMD (95% CI [−0.14, 0.23]), 0.25 SMD (95% CI [0.04, 0.46]) and 0.09 SMD (95% CI [−0.25, 0.43]).

**In summary**, our meta‐analysis on PES's effects on total household income suggests an overall positive effect with an increase in total household income of 0.25 SMD, 95% CI [0.09, 0.41]. This result, however, is subject to large heterogeneity across the included studies, which are further subject to a very serious risk of bias. In addition, while comprising eight studies, the meta‐analysis only synthesised evidence of the effects of four PES programmes. Using the GRADE scale to assess the strengths of the evidence in this meta‐analysis, we rate the meta‐analysis' results to be based on low quality of evidence (Table 7).

##### 6.6.3.3 Socioeconomic outcomes (3): Effects of PES on nonagricultural income

Nine studies provided outcome data on nonagricultural income for inclusion in the meta‐analysis. Seven of these studies are the same studies from China included above, with the other studies being of the PAS programme in Brazil and the PSAH in Mexico.

The average effect of these programmes on nonagricultural income is 0.05 SMD, 95% CI [−0.03, 0.13], calculated under a random‐effect model. This overall effect has a moderate amount of variability between the studies (*I* ^2^ = 43.35%, *τ* ^2^ = 0.0058, *Q*(df = 8) = 12.6829, *p* = .1232). There is a wide range in effects, from a negative effect reported for one of the China studies (SMD = −0.07 [−0.18, −0.03]) to a positive effect reported for Duan et al.'s (2015) evaluation of the SLCP in China of 0.26SMD (95% CI [0.03,0.50]).

$I^{2}=54.69\%,\;\tau^{2}=0.0095,\;Q(df=6)=14.7323,\;p=.02.$

$$I^{2}=43.35\%,\;\tau^{2}=0.0058,\;Q(df=8)=12.6829,\;p=.1232.$$

 

**Figure 8:**
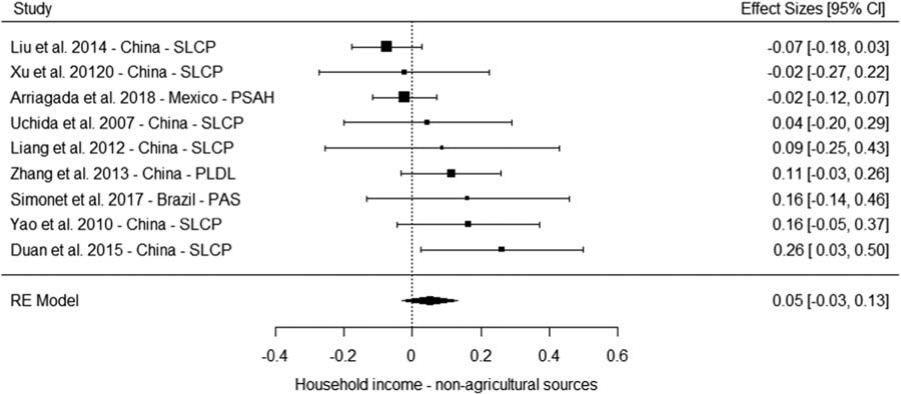
Effects of payment for environmental service on household income from nonagricultural sources

We were also able to calculate an additional 12 effect sizes for nonagricultural sources of household income, which we were unable to include in the meta‐analysis due to dependencies or different outcome constructs. These come from seven studies from China (Liang et al., 2012; Liu et al., 2014; Xu et al., 2010; Yao et al., 2010; Zheng et al., 2013), Malawi (Jack & Santos, 2017) and Nepal (Sharma et al., 2015). (Figure 8)

Liu et al. (2014) report the effects of the SLCP in China combined with a non‐PES conservation programme to prevent logging and other harmful activities, the NFPP on nonfarm income, finding no effect (SMD = 0.05, 95% CI [−0.02, 0.12]). Xu et al. (2010) also report the effects of the SLCP programme on other sources of income, including aquaculture, rental and interest income, gifts, pension income and government subsidies. They find no effect of the SLCP on this outcome (SMD = −0.02, 95% CI [−0.27, 0.23]). Yao et al. (2010) also look at the impact of the SLCP on a measure of other sources of income, including family properties and government subsidies, also finding no effect (SMD = 0.01, 95% CI [−0.19, 0.22]).

Liang et al. (2012) report the effects of the SLCP in China on local wage income for households with adults and the elderly, households with only adults and households with only adults and children and households. They find effects of 0.09 SMD (95% CI [−0.25, 0.44]), 0.05 SMD (95% CI [−0.14, 0.23]) and 0.29 SMD (95% CI [0.09, 0.50]).

Liu et al. (2014) also report an additional effect size for the effect of the DCBT programme in China on household nonfarm income, again finding no effect (SMD = 0.01, 95% CI [−0.09, 0.12]). Finally, Zheng et al. (2013) look at the effect of the PLDL programme in China on income from migration. They find a positive impact of 0.22 SMD (95% CI [0.08, 0.37]).

Jack and Santos (2017) present results for two intervention groups in the Malawi PES experiment, a group that received the PES programme after participating in a lottery and a group that participated in an auction, on whether or not households report income from casual labour. For the lottery group, they find a positive effect of 0.24 SMD (95% CI [0.14, 0.47]) and for the auction group, a nonsignificant effect of 0.15 SMD (95% CI [−0.08, 0.38]). Finally, Sharma et al. (2015) report results of the PES REDD+ pilot in Nepal on Household income from CFUGs activities and gross income from CFUGs, finding no effect (respectively, 0.01 SMD, 95% CI [−0.14, 0.17], 0.03 SMD, 95% CI [−0.12, 0.19]).

**In summary**, our meta‐analysis on PES's effects on household income from nonagricultural sources finds an overall positive effect (0.05 SMD, 95% CI [−0.03, 0.13]). The result is further subject to moderate heterogeneity across the included studies, and the underlying studies suffer from very serious risk of bias. In addition, while comprising nine studies, the meta‐analysis only synthesised evidence of the effects of four PES programmes. Using the GRADE scale to assess the strength of the evidence in this meta‐analysis, we rate the meta‐analysis' results to be based on a very low quality of evidence (Table 7). The cautious results of the meta‐analysis are largely supported by the effect sizes not included in the meta‐analysis due to dependencies and heterogeneous outcome constructs, of which a large majority of studies do not identify any substantively significant effects.

##### 6.6.3.4 Socioeconomic outcomes (4): Effects of PES on agricultural income

Nine studies provided outcome data on agricultural income for inclusion in the meta‐analysis. Seven of these studies are the same studies from China included above, with the other two studies being of an ICRAF programme in Malawi (Jack & Santos, 2017) and the P‐SAH in Mexico (Arriagada et al., 2018).^13^

**Figure 9:**
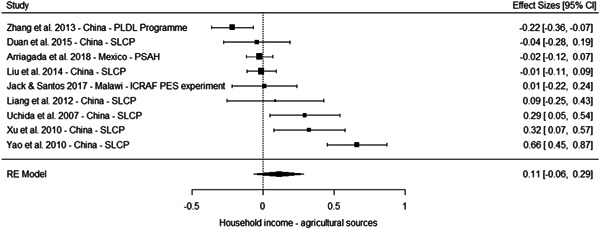
Effects of payment for environmental service on household income from agricultural sources

As expected the average effect of these programmes on agricultural income is smaller than the effect on overall income and nonagricultural income, but just as with the latter it remains imprecise with the CI crossing the line of no effect (SMD = 0.11, 95% CI [−0.06, 0.29], calculated under a random‐effect model). Inspecting the forest plot in Figure 9 suggests substantial variability between studies, and this is also suggested by the statistical tests (*I* ^2^ = 89.15%, 0.0605 (*SE* = 0.0359), *Q* (df = 8) = 57.1129, *p* < .0001). While sensitivity analysis suggests removing Yao et al.'s (2010) evaluation of the SLCP from China result in a reduction in the overall average effect size to 0.03 SMD (95% CI [−0.09, 0.15]), the estimate remains statistically insignificant.

$$I^{2}=89.15\%,\;\tau^{2}=0.0605(SE=0.0359),\;Q(df=8)=57.1129,\;p<.0001.$$

 

We were able to calculate 11 additional effect sizes for the effects of PES on agricultural sources of household income, which we were unable to include in the meta‐analysis due to dependencies or different outcome constructs. Thirteen of these effects are from programmes in China (Duan et al., 2015; Liang et al., 2012; Liu et al., 2014; Xu et al., 2010; Yao et al., 2010; Zheng et al., 2013) while one reports on a different trial arm of an RCT in Malawi (Jack & Santos, 2017).

Xu et al. (2010) also report the effects of the SLCP in China on total agricultural income with subsidy, as opposed to total agricultural income without the PES subsidy included in the meta‐analysis. They find a positive effect of 0.33 SMD (95% CI [0.08, 0.58]). They also report several additional agricultural income outcomes, finding a positive effect on husbandry income including both sales income and own consumption (SMD = 0.29, 95% CI [0.04, 0.54]) and cropping income with and without subsidy (respectively, SMD = 0.66, 95% CI [0.40, 0.91] and SMD = 0.66, 95% CI [0.41, 0.92]). This suggests that the overall increase in agricultural income from the SLCP evaluated in Xu et al. (2010), shown in Figure 9, is driven by the increase in crop income. Liu et al. (2014) report the combined effects of the NFFP and SLCP in China as well as the effect of the DCPT programme on land based income, finding effects of −0.02 SMD (95% CI [−0.12, 0.08]) and −0.04 SMD (95% CI [−0.15, 0.06]) respectively.

Yao et al. (2010) also report the effect of the SLCP on animal husbandry, finding a negative effect of the SLCP in China of −0.29 SMD, 95% CI (−0.49, −0.07). Uchida et al. (2009) report the effects of the SLCP on other agricultural income per capita (as opposed to income from cropping included in the meta‐analysis), finding a positive effect of 0.41 SMD (95% CI [0.17, 0.66]). Duan et al. (2015) report the effect of the SLCP on household income from forests, finding an insignificant effect of 0.07 (95% CI [−0.17, 0.30]). Zhang et al., 2013 report the effects of the PLDL programme in China on the % of income from agricultural sources, finding a negative effect of −0.47 SMD, (95% CI [−0.62, −0.32]).

Finally, Jack and Santos (2017) present results for two intervention groups in the Malawi PES experiment, a group that received the PES programme after participating in a lottery and a group that participated in an auction. The lottery group is included in the meta‐analysis. They also report the effects on income from crop sales of the auction allocation trial arm, finding an effect of 0.21 SMD (95% CI [−0.02, 0.44]).

**In summary**, the meta‐analysis of the effect of PES on agricultural income suggests a large amount of heterogeneity between studies, and the overall estimate is imprecise (SMD = 0.11, 95% CI [−0.06, 0.29]). The studies contributing data to the analysis are subject to a very serious risk of bias, and includes evidence from only four PES programmes. Using the GRADE scale to assess the strength of the evidence in this meta‐analysis suggest the findings are based on a very low quality of evidence (Table 7). The effect sizes not included in the meta‐analysis due to dependencies and heterogeneous outcome constructs also provide mixed results.

##### 6.6.3.5 Socioeconomic outcomes (5): Effects of PES on household assets

Three studies, from Costa Rica, Malawi and Mexico, provided outcome data on the effects of PES on an asset index at the household level. The meta‐analysis suggests that the average effect of PES on assets is close to zero (SMD = 0.04, 95% CI [−0.12, 0.20]), calculated under a random‐effect model). The effect is fairly consistent across studies, as is evident from both the overlapping CIs in the forest plot and heterogeneity tests (*I* ^2^ = 0.00%, *τ* ^2^ = 0.0, *Q*(df = 2) = 0.3748, *p* = .8291), although the CIs are wide.

**Figure 10:**
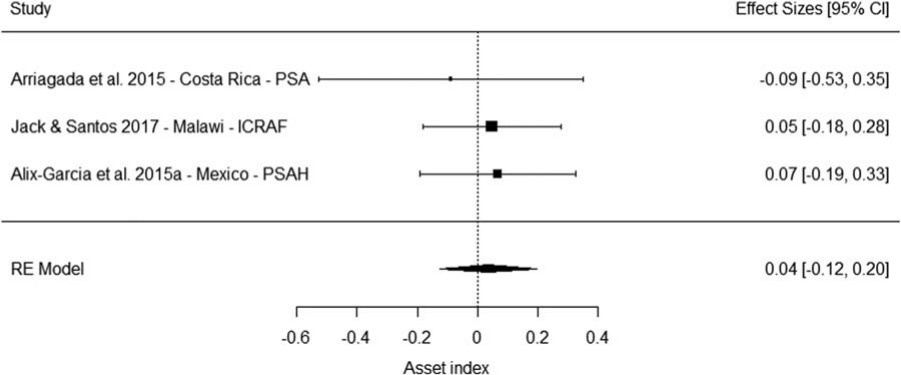
Effects of payment for environmental service on household asset index

Jack and Santos (2017) present results for two intervention groups in the Malawi PES experiment, a group that received the PES programme after participating in a lottery and a group that participated in an auction. The results in the meta‐analysis are the group that participated in the lottery as this method of allocation was more similar in terms of intervention to the other programmes in the meta‐analysis. However, the impact on the household asset indexes for the auction group was higher than the average effect size (SMD = 0.10, 95% CI [−0.12, 0.33]), although the effect is still small and imprecise. In addition, Alix‐Garcia et al. (2015) present results by PES contracts allocated to private property and those allocated to common property. The results in the meta‐analysis are the private property group as these are more similar to the other programmes in the meta‐analysis. For the common property group, they find a similarly small effect of 0.06 SMD (95% CI [0.00, 0.12]). (Figure 10)

$$I^{2}=0.00\%,\;\tau^{2}=0.0,(SE=0.0204),\;Q(df=2)=0.3748,p=.8291.$$

We also identified three studies that provided outcome data on the number of household assets as a count or value measure rather than as an index, however these were too diverse to combine in a meta‐analysis. These were studies from Mozambique (Jindal et al., 2012), Costa Rica (Arriagada et al., 2015) and China (Uchida et al., 2009). In Mozambique, Jindal et al. (2012) find a statistically insignificant effect of 0.09 SMD (95% CI [0.21, 0.39]) on asset ownership per household. In Costa Rica, Arriagada et al. (2015) find a decrease in the number of household assets of −0.16 SMD between 1996 and 2005 (95% CI [−0.60, 0.28]), although this is statistically insignificant. In China, Uchida et al. (2009) find a positive impact of the SLCP on the value of houses (yuan) (SMD = 0.31, 95% CI [0.07, 0.56]), fixed productive assets (yuan) (SMD = 0.10, 95% CI [−0.34, 0.15]) and livestock inventories (SMD = 0.34, 95% CI [0.10, 0.59]).

**In summary**, the meta‐analysis on PES's effects on households' assets suggests no change in asset outcomes (SMD = 0.04, 95% CI [−0.12, 0.20]). This result is fairly consistent across the studies, although the underlying evidence base is limited to three studies, all subject to serious risk of bias. Using the GRADE scale to assess the strength of the evidence in this meta‐analysis, we rate the meta‐analysis' results to be based on a very low quality of evidence (Table 7). The effect sizes not included in the meta‐analysis due to dependencies and heterogeneous outcome constructs suggest mixed results.

##### 6.6.3.6 Socioeconomic outcomes (other): Narrative overview of effects

###### 6.6.3.6.1 Effects of PES on employment

We identified five studies that assessed the impact of PES on a measure of employment, from which we were able to calculate 22 effect sizes. However, these were too diverse to combine in a meta‐analysis and we therefore report the results narratively. The studies are from Mozambique (Jindal et al., 2010) and China (Groom et al., 2010; Liu et al., 2015, 2018; Uchida et al., 2009).

Three of the studies from China report on measures of household changes in off and on farm labour supply. Groom et al. (2010) assess the impact of the SLCP programme in China on household off‐farm labour supply, finding an overall small and imprecise effect of 0.04 SMD (95% CI [−0.30, 0.38]). However, they also break down the results by whether the household faces constraints on off‐farm work or not. For the constrained households, they find a fairly large effect of 0.64 SMD (95% CI [0.29, 0.98]) on off farm labour supply, whereas for the unconstrained households they find a negative but imprecise effect of −0.13 SMD (95% CI [−0.47, 0.21]). Uchida et al. (2009) also look at the impact of the SLCP programme in China on various indicators of off and on farm labour status. For change in off farm labour status, they find a positive impact of 0.25 SMD (95% CI [0.07, 0.43]). For change in on farm labour status, they also find a positive impact of 0.21 SMD (95% CI [0.03, 0.39]). For the effect of the SLCP on the number of adults with off‐farm work in the household, they find a positive but less precise impact of 0.20 (95% CI [−0.04, 0.45]). Liu et al. (2018) report on the effects of the SLCP, the DCBT and the SLCP combined with another non‐PES programme, the NFPP in China on off‐farm labour time inputs in terms of person‐days. For the SLCP, they find a positive effect of 0.16 SMD (95% CI [0.04, 0.27]) on off‐farm labour time. Conversely, for households that received the SLCP combined with the NFPP, they find a negative effect of −0.22 SMD (95% CI [−0.33, −0.10]). Finally, they find an effect of 0.13 SMD (95% CI [0.01, 0.24]) for the DCBT programme.

Finally, Liu et al. (2015) report 12 effects of the SLCP on an index of Household income diversity (HDI), by year of implementation of the programme, from 1999 to 2010. In the first 3 years 1999, 2000 and 2001, the effect on household income diversification are 0.1 SMD or less (respectively, SMD = 0.07, 95% CI [−0.04, 0.18], SMD = 0.10, 95% CI [−0.02, 0.21], SMD = 0.03, 95% CI [−0.08, 0.14]). From 2002, the effect on the HDI is slightly bigger, with the largest impact of 0.20 SMD (95% CI [0.09, 0.31]) on household income diversification in 2008 after 9 years of implementation of the SLCP.

###### 6.6.3.6.2 Effects of PES on food security

We identified three studies that assessed the impact of PES on a measure of food security, from which we were able to calculate seven effect sizes. These were too diverse to combine in a meta‐analysis and we therefore report the results narratively. The studies are from Mexico (Alix‐Garcia et al., 2015a), Malawi (Jack & Santos, 2017) and Uganda (Jayachandran et al., 2017).

Alix‐Garcia et al. (2015a) present results by PES contracts allocated to private property and those allocated to common property, on an index of food consumption, using prices reported by households and whether or not they purchased a particular food item in the past month. For households living in areas under common property contracts, the effect of the food index is 0.09 SMD (95% CI [−0.03, 0.21]) but statistically insignificant, while the effect on households in private property is −0.06 SMD (95% CI [−0.43, 0.31]), again statistically insignificant. In addition, Jack and Santos (2017) present results for two intervention groups in the Malawi PES experiment, a group that received the PES programme after participating in a lottery and a group that participated in an auction. They report effects on per capita spending on food, finding a statistically insignificant effect of −0.12 SMD (95% CI [−0.35, 0.11]) for the lottery group and a statistically insignificant effect of 0.17 SMD (95% CI [−0.06, 0.40]) for the auction group. Both effect sizes are imprecise. In addition, they report effects of the experiment on months of food shortages, finding an effect of −0.04 SMD for the lottery group (95% CI [−0.27, 0.19]) and an effect of 0.11 SMD for the auction group (95% CI [−0.12, 0.34]). Again, both effect sizes are imprecise. Finally, Jayachandran et al. (2017) report the effects of the PES RCT in Uganda on food expenditure in the past 30 days. They find an imprecise effect of −0.03 SMD (95% CI [−0.15, 0.10]).

###### 6.6.3.6.3 Effects of PES on education

We identified three studies that assessed the impact of PES on a measure of education, from which we were able to calculate six effect sizes. These were too diverse to combine in a meta‐analysis and we therefore report the results narratively. The studies are from Mozambique (Jindal et al., 2012), China (Zheng et al., 2013) and Mexico (Alix‐Garcia et al., 2015a).

Jindal et al. (2012) find an insignificant effect of 0.08 SMD (95% CI [−0.21, 0.38]) on the number of literate people per household. Zheng et al. (2013) find a statistically insignificant effect of 0.13 SMD (95% CI [−0.01, 0.28]) of the PLDL program on household spending on education in yuan. Alix‐Garcia et al. (2015a) report the effects of the PSA‐H programme in Mexico on four education investment outcomes, divided by the age group of the people receiving the investment and whether the PES contracts were allocated to private property or common property. They find an insignificant effect of 0.11 SMD (95% CI [−0.16, 0.38]) on household education investment for young people aged 12–22 in private property. For education investment for young people aged 12–14 in common property, they find an insignificant effect of 0.07 SMD (95% CI [−0.09, 0.23]) and for education investment for young people aged 15–17 in common property, they find an effect of 0.13 SMD (95% CI [−0.02, 0.28]). Finally, for education investment for young people aged 18–22 in common property, they find an effect of 0.05 SMD (95% CI [−0.84, 0.17]).

###### 6.6.3.6.4 Effects of PES on Poverty Indicators

We identified four studies that assessed the impact of PES on an indicator of poverty status. However, one the studies did not provide sufficient data to calculate effect sizes (Robalino et al., 2014). For the remaining three, were able to calculate three effect sizes. The studies are from Tanzania (John, 2012), Camobdia (Beauchamp et al., 2018) and Mexico (Sims & Alix‐Garcia, 2017). John (2012) presents the results of the EPWS programme in Tanzania on welfare, finding an effect size of 0.32 SMD (95% CI [0.03, 0.61]). Sims and Alix‐Garcia (2017) present the effect of the PSAH in Mexico on a weighted average of indicators including rates of literacy, primary schooling, availability of potable water, sanitation and electricity, and housing characteristics. They present the results for share of the locality engaged in the PES programme, finding an effect of only 0.03 SMD [0.01, 0.04]). Beauchamp et al. (2018) present the results of the Bird Nest Protection Program in Cambodia on economic status, calculated using the Basic Necessities Survey. They found an effect of 0.04 SMD (95% CI [−0.13, 0.22]).

###### 6.6.3.6.5 Effects of PES on other socioeconomic outcomes

We identified three studies that assessed the impact of PES on another socioeconomic outcome that did not fit into the other categories, from which we were able to calculate seven effect sizes. We were unable to undertake meta‐analysis due to too few studies or heterogeneous outcome constructs. The studies are from Mozambique (Jindal et al., 2012), Uganda (Jayachandran et al., 2016, 2017) and Mexico (Sims & Alix‐Garcia, 2017). Jindal et al. (2012) report the effect on the number of m'shambas (farmer fields) per household, finding an effect of 0.22 SMD (95% CI [−0.08, 0.52]).

Jayachandran et al. (2016, 2017) assess the effect of the PES experiment in Uganda on various socioeconomic outcomes. They find an insignificant effect of 0.05 SMD (95% CI [−0.07, 0.18]) on nonfood expenditure in the past 30 days and an insignificant effect on alcohol and tobacco expenditure in the last 30 days of −0.08 SMD (95% CI [−0.20, 0.05]). In addition, they find that the PES experiment reduced the number of households that had outstanding loan or repaid a loan in past year by −0.13 SMD (95% CI [−0.26, −0.01]). They also find that PES reduced the number of households with a child that was sick with malaria in last 30 days (age, 0–15) by −0.16 SMD (95% CI [−0.24, −0.07]) and the number of households with a child sick with diarrhoea in last 30 days (age, 0–5) by −0.33 SMD (95% CI [−0.51, −0.15]). Finally, Sims and Alix‐Garcia (2017) assess the effect of the PSAH programme in Mexico on population growth, in terms of hundreds of people per square km. They present the results for share of the locality engaged in the PES programme, finding an effect of −0.02 SMD (95% CI [−0.03, 0.00]).

##### 6.6.3.7 Summary of PES's effects on socioeconomic outcomes

We are able to provide synthesised evidence on the effects of PES programmes on four socioeconomic outcomes: total household income, household income from nonagricultural sources, on agricultural income and on asset indexes. These meta‐analyses cover 8 of the 18 individual PES programmes, are subject to a high degree of heterogeneity, and are based on a body of research that suffers from a very serious risk of bias. Using the GRADE scale to assess the strength of the evidence in the meta‐analyses, we rate three meta‐analyses to be based on *very low quality of evidence* and one meta‐analysis as *low quality of evidence* (Table 7).

Keeping the above caveats in mind, the results of the meta‐analyses overall suggest that PES programmes have, at best, *mixed effects on socioeconomic outcomes*. Of four meta‐analysis conducted to assess different socioeconomic outcomes, we find a positive effect on measures of total household income. In contrast, PES had no clear effect on household income from nonagricultural sources,^14^ on agricultural income and on asset indexes.

#### 6.6.4 Environmental outcomes

Despite PES having environmental protection as a primary objective, of the 18 included programmes we only have results for 11 programmes in terms of their effects on environmental outcomes. This corresponds to 19 studies out of a total 44. There were also some major programmes for which we identified no evaluations of environmental outcomes, notably the SLCP programme in China. We began by undertaking a meta‐analysis across environmental outcomes to get an initial idea of the effect of PES programmes on this set of outcomes. This includes deforestation, forest cover and other measures of tree or vegetation cover. However, this meta‐analysis combines a diverse set of outcome variables that may not be comparable and we therefore also undertake meta‐analysis for two more homogeneous sets of environmental outcomes: (a) forest cover and (b) deforestation^15^ and present results narratively for (3) other environmental outcomes. The outcome forest cover allows for a positive outcome in the expansion of forested land resulting from the programme, while deforestation includes only the impact on the rates of forest loss. We were only able to undertake a meta‐analysis for forest cover and deforestation. For the other forest outcomes, including forest condition, we report effect sizes narratively only in Appendix 8.

##### 6.6.4.1 Environmental outcomes (1): Overall effects of PES on environmental outcomes

Eleven studies provided data on environmental outcomes for inclusion in the meta‐analysis. This included PES programmes in Colombia, Uganda, two programmes from Mexico, Costa Rica, Ecuador, Brazil, Cambodia and Nepal. Our meta‐analysis of the average effect aross these studies suggest an improvement in environmental outcomes of 0.21 SMD (95% CI [0.09, 0.33]), calculated under a random effects model. There is a high amount of heterogeneity attached to this set of results (*I* ^2^ = 88.16%, *τ* ^2^ = 0.0272, Q(df = 10) = 116.9430, *p* < .0001), which can also be seen in the forest plot. Results vary from a insignificant negative effect of the silvopastoral project in Colombia on an environmental services index (ESI) (−0.10 SMD, 95% CI [−0.52, 0.33]) up to an increase in forest cover as a result of the PSA programme in Costa Rica of 0.60 SMD, 95% CI [0.22, 0.98]). The results are sensitive to the removal of Arriagada et al.'s (2012) study in Costa Rica, the average effect goes down to 0.14 SMD (95% CI [0.07, 0.23]) and there is a more moderate amount of heterogeneity (*I* ^2^ = 67.21, *τ* ^2^ = 0.0090).

##### 6.6.4.2 Environmental outcomes (2): Effects of PES on forest cover

Five studies provided data on forest cover for inclusion in meta‐analysis, including studies of two different programmes in Mexico, one study in Brazil, Costa Rica and Uganda. For the Alix‐Garcia et al. (2015) study from Mexico, we include their outcome dry season normalised difference vegetation index (NDVI) in this meta‐analysis. Our meta‐analysis of the average effect across these studies suggest an improvement in forest cover (SMD = 0.32, 95% CI [0.10, 0.55], calculated under a random effect model).

**Figure 11:**
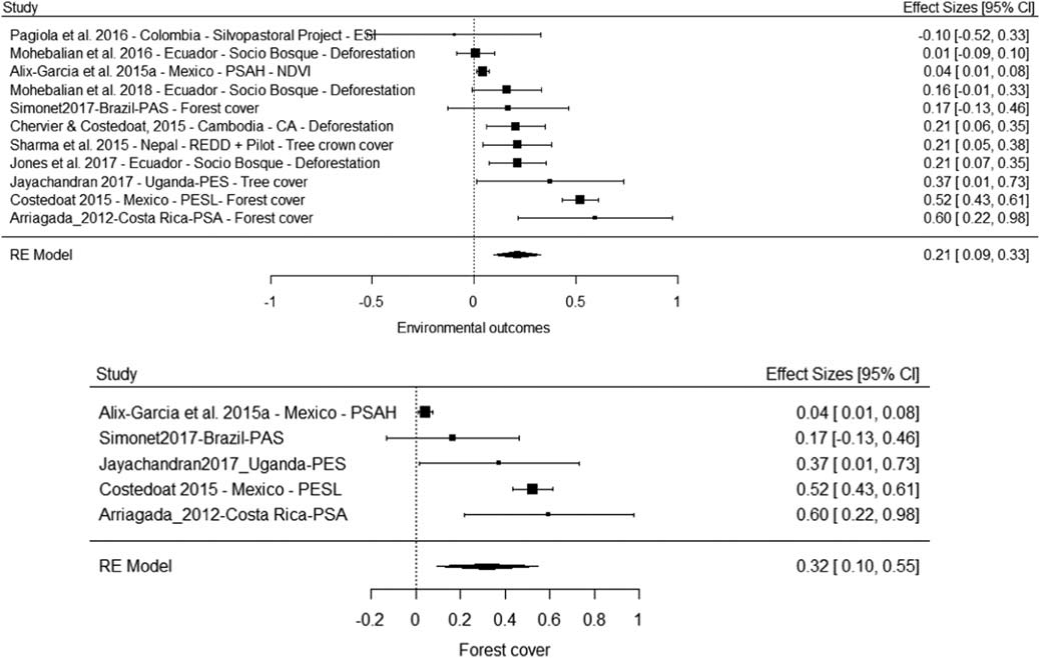
Effects of payment for environmental service on environmental outcomes

There is a high degree of heterogeneity attached to this estimate (*I* ^2^ = 92.74%, *τ* ^2^ = 0.0500, *Q*(df = 4) = 105.6837, *p* ≤ .0001). This can be seen visually in the forest plot in Figure 11, where effects range from 0.04 SMD (95% CI [0.01, 0.08]) in Mexico up to 0.60 SMD (95% CI [0.22, 0.98]) in Costa Rica. Removing the study from Mexico from the analysis eliminates most heterogeneity and increases the overall estimate (SMD = 0.43, 95% CI [0.25, 0.61]).

$$I^{2}=95.98\%,\;\tau^{2}=0.0639,\;Q(df=3)=135.3948,p=<.0001.$$

**Figure 12:**
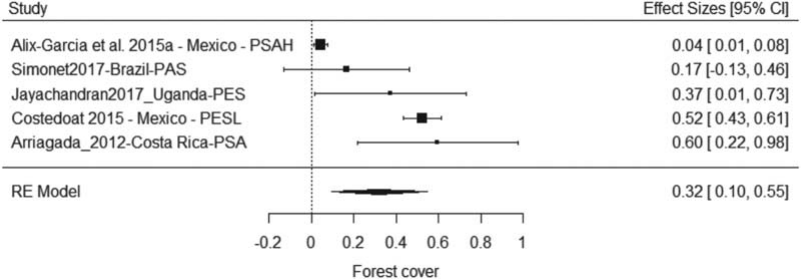
Effects of payment for environmental service on forest cover

We were able to calculate an additional 11 effect sizes for indicators of forest cover that could not be included in the meta‐analysis due to dependencies or different outcome constructs. These came from Costa Rica (Arriagada et al., 2012, 2008; Sierra & Russman, 2006), Uganda (Jayachandran et al., 2016, 2017), Mexico (Alix‐Garcia et al., 2015a; Sims & Alix‐Garcia, 2017) and Tanzania (Lokina & John, 2016) (Figure 12)

In Costa Rica, Arriagada et al. (2012) assess the impact of the PSA on change in forest cover on the farm between 1992 and 2005, using imputed data for missing results (as compared with the results included in the meta‐analysis which did not use imputed data), finding a smaller effect size of 0.49 SMD (95% CI [0.17, 0.82]). In Mexico, Sims and Alix‐Garcia (2017) assess the effect of the PSAH on the net change in forest cover from 2000 to 2012, finding a very small negative effect of −0.02 SMD (95% CI [−0.03, −0.01]). Ali‐xGarcia et al. (2015a) estimate the effect of the PSAH on locality level forest cover, finding an effect of 0.04 SMD (95% CI [0.02, 0.05]).

In an earlier paper on the PSA in Costa Rica, Arriagada et al. (2008) assess the effects on self‐reported native forest cover change in hectares, again with an estimation using only observed data and with an estimation using imputed data for missing results. Using only observed data, they find a statistically insignificant effect on forest cover of 0.11 SMD (95% CI [−0.18, 0.41]). Using imputed data, they find a smaller, statistically insignificant effect on self‐reported forest cover of 0.05 SMD (95% CI [−0.23, 0.32]). Lokina and John (2016) assess the impact of the EPWS in Tanzania on perception of forest size, finding a statistically insignificant effect of 0.11 SMD (95% CI [−0.17, 0.39]).

Sierra and Russman (2006) estimate the effect of the PSA programme on the percent of land under intervened forest cover and percent of land under primary forest, finding a positive effect for intervened forest cover of 0.40 SMD (95% CI [−0.12, 0.90]) but a fairly large decrease in land under primary forest of −0.48 SMD (95% CI [−0.99, 0.03]).

Jayachandran et al. (2016, 2017) report a number of measures of forest cover that we could not include in the meta‐analysis due to dependencies. For the outcome change in tree cover in hectares, measured as a circle around the private forest owner home, they find a positive effect of 0.16 SMD (95% CI [0.03, 0.28]). This is smaller than the effect included in the meta‐analysis, where they measure effects at the village boundary level. They find a fairly large effect on reforestation area of 0.38 SMD (95% CI [0.26, 0.50]) and the total number of trees that survived 0.38 SMD (95% CI [0.26, 0.50]).

**In summary**, the meta‐analysis suggests PES results in an overall improvement in forest cover (SMD = 0.32, 95% CI [0.10, 0.55]). There is a large amount of heterogeneity, but this is driven by a smaller effect of the PSAH programme in Mexico, and removing this study from the analysis result in a larger overall estimate. The studies have a comparatively low risk of bias, but the small number of studies suggest caution in generalising the finding to other contexts without further research. Using the GRADE scale to assess the strength of the evidence in this meta‐analysis, we rate the meta‐analysis' results to be based on a low quality of evidence (Table 7). The effect sizes not included in the meta‐analysis due to dependencies and heterogeneous outcome constructs suggest mixed results.

###### 6.6.4.2.1 Effects of PES on forest cover spill overs

We only identified one paper that tested for spill‐over effects of PES programmes onto nonenroled forest areas, Jayachandran et al.'s (2016) evaluation of a PES experiment in Uganda. We were able to estimate two effect sizes from this paper. They do not find evidence of spill overs of the PES programme onto forest reserves not in the programme, as assessed by interacting the treatment variable with distance to forest reserves (SMD = 0.02, 95% CI [−0.07, 0.10]) or PES contract areas being contiguous to forest covers (SMD = −0.06, 95 CI [−0.15, 0.02]).

##### 6.6.4.3 Environmental outcomes (3): Effects of PES on deforestation

Six studies provided data on deforestation rates for inclusion in meta‐analysis, including studies of a programme in Mexico, one study in Costa Rica, one study in Cambodia and three studies of the Socio Bosque programme in Ecuador looking at the effect of the programme of different parts of the country. A negative effect size for deforestation indicates a desirable outcome, as it indicates a reduction in the rate of deforestation. Our meta‐analysis of the average effect across these studies suggest an improvement in deforestation (SMD = −0.12, 95% CI [−0.19, −0.05], calculated under a random effect model).

**Figure 13:**
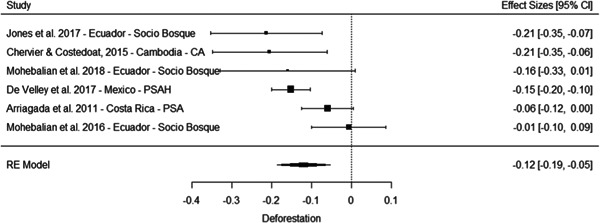
Effects of payment for environmental service on deforestation

There is a moderate degree of heterogeneity attached to this estimate (*I* ^2^ = 65.95%, *τ* ^2^ = 0.0040, *Q*(df = 5) = 13.8505, *p* = .0166). This can be observed in the forest plot in Figure 13. This heterogeneity applies both across programmes and within programmes; Jones et al. (2017) find a positive effect of the Socio Bosque programme on deforestation in Ecuador (SMD = −0.21, 95% CI [−0.35, −0.07]), that is, a reduction in deforestation, while Moheabalian et al. (2016) find no effect of the programme on deforestation (SMD = −0.01, 95% CI [−0.10, 0.09]).

 

$$I^{2}=65.95\%,\;\tau^{2}=0.0040,\;Q(df=5)=13.8505,\;p=.0166.$$

We were also able to calculate an additional seven effect sizes for deforestation from three studies, which were too heterogeneous to be included in the meta‐analysis or had dependencies with included effect sizes. These came from Costa Rica (Robalino et al., 2015; 2008; Robalino et al., 2013) and Mexico (De Velley et al., 2017).

Robalino et al. (2008) assess the impact of the PSA programme in Costa Rica on the 5‐year effect on deforestation in percent and the result is not substantially different from zero (SMD = −0.02 SMD, 95% CI [−0.08, 0.05]). In a later update of the paper (Robalino et al., 2015), the authors also assess the effect of the PSA in a national park compared with households without PES and not in a national park, on deforestation between 2000 and 2005, finding a small reduction in deforestation, however the CIs cross the line of no effect (SMD = −0.08, 95% CI [−0.19, 0.04]).^16^ Assessing the effect of PES on deforestation in a buffer zone around a national park versus in buffer zones without PES, suggests a reduction in rates of deforestation (SMD = −0.13, 95% CI [−0.22, −0.04]). Finally, Robalino et al. (2013) assess the effect of the PSA programme on deforestation in the first 3 years of implementation from 1997 to 2000, finding a small effect of −0.06 SMD (95% CI [−0.09, −0.01]).

De Velley et al. (2017) assess the impact of the PSAH programme in Mexico on forest loss in three types of land; land (analysed at the grid level) newly enroled into the programme, land under renewed contracts and land that had not had its PES contract renewed. For newly enroled land, they find the programme reduced forest loss by −0.10 SMD (95% CI [−0.15, −0.05]). They find a slightly larger effect on forest loss on renewed land and no effect on land without a renewed contract (SMD = −0.13 (95% CI [−0.17, −0.08] and SMD = 0.01, 95% CI [−0.04, 0.06]).

**In summary**, the meta‐analysis suggests a reduction is deforestation as a result of PES (SMD = −0.12, 95% CI [−0.19, −0.05]). However, on the result is based on studies with a very serious risk of bias and a small underlying evidence (five studies of three programmes). Using the GRADE scale to assess the strength of the evidence in this meta‐analysis, we rate the meta‐analysis' results to be based on a low quality of evidence (Table 7). The effect sizes not included in the meta‐analysis due to dependencies and heterogeneous outcome constructs support the findings of the meta‐analysis similarly pointing towards a reduction in deforestation rates following the introduction of PES programmes.

##### 6.6.4.4 Environmental outcomes (4): Effects of PES on other environmental outcomes

We identified four studies that assessed the effects of PES on an environmental outcome other than forest cover or deforestation, from which we were able to calculate 22 effects sizes. We were unable to undertake meta‐analysis as a result of too few studies or heterogeneous outcome constructs. The results are from studies from Nepal (Sharma et al., 2015), Colombia (Pagiola et al., 2016, 2013), Mexico (Alix‐garcia et al., 2015a) and Ecuador (Mohebalian & Aguilar, 2018)

Two studies assessed indicators of forest condition. Sharma et al. (2015) assess the effects of REDD+ Pilot in Nepal on six outcomes. They find an insignificant effect on total forest carbon (SMD = 0.09, 95% CI [−0.08, 0.26]) and an insignificant effect on signs of soil erosion (SMD = −0.15, 95% CI [−0.31, 0.02]). They also find an insignificant effect on shrub cover observed in the sampled forest plots (SMD = 0.06, 95% CI [−0.22, 0.11]). They find a positive effect of the pilot on tree crown cover observed in the sampled forest plots (SMD = 0.21, 95% CI [0.05, 0.38]) and a positive effect on grass cover observed in the sampled forest plots (SMD = 0.20, 95% CI [0.03, 0.37]). Finally, they find a positive effect on signs of wildlife observed in the sampled forest plots (SMD = 0.19, 95% CI [0.02, 0.35]). Mohebalian and Aguilar (2018) assess the effect of the Socio Bosque on three forest condition outcomes. They find a large positive effect on tree species richness (frequency) of 1.05 SMD (95% CI [0.37, 1.73]) and for tree species with commercial timber value (frequency) of 0.50 SMD (95% CI [−0.15, 1.14]). They find an insignificant effect on trees species at risk of extinction (frequency) of 0.19 SMD (95% CI [−0.44, 0.82]).

Pagiola et al. (2016, 2013) assess the effect of the RISEMP in Colombia on the ESI at various follow up periods during the programme and after it had stopped. This programme had several treatment groups, one with PES combined with technical assistance around silvopastoral practices and one PES group without. In addition, two of the groups received the programme for 4 years while one received for just 2 years. All the results the authors found are statistically insignificant. For the group receiving just PES for 4 years in the post‐PES implementation period of 2007–2011, they find a statistically insignificant effect of −0.10 SMD on the ESI (95% CI [−0.52, 0.33]). For the group receiving PES and technical assistance for 4 years in the post‐PES implementation period of 2007–2011, they find a statistically insignificant effect of 0.09 SMD on the ESI (95% CI [−0.34, 0.51]). For the group that received PES and technical assistance for 2 years, in the post‐PES implementation period of 2007–2011 they find a statistically insignificant effect of 0.18 SMD on the ESI (95% CI [−0.25, 0.61]). Pagiola et al. (2013) look at the effects in an early period during the programme. For the group receiving PES and technical assistance, they find an insignificant effect of 0.17 SMD (95% CI [−0.26, 0.60]) on ESI per hectare and an effect of 0.36 SMD (95% CI [−0.08, 0.79]) on ESI overall. Finally, for the group receiving just PES, they find an effect of 0.18 SMD (95% CI [−0.25, 0.61]) on ESI per hectare and an effect of −0.14 SMD (95% CI [−0.57, 0.29]) for ESI overall.

##### 6.6.4.5 Summary of PES's effects on environmental outcomes

In total, we are able to provide synthesised evidence on the effects of PES programmes on two environmental outcomes: forest cover and deforestation. These meta‐analyses cover only five of the 18 individual PES programmes, are subject to a high degree of heterogeneity, and are based on a body of research that is characterised by a very serious risk of bias. Using the GRADE scale to assess the strength of the evidence in the meta‐analyses, we rate both meta‐analyses to be based on *low quality evidence* (Table 7).

Keeping the above caveats in mind, the results of the meta‐analysis overall suggest that PES programmes can have *positive effects on environmental outcomes in some contexts*. The two meta‐analyses identify an improvement in deforestation rates and forest cover respectively.

#### 6.6.5 Moderator analysis—how do results vary by region and income level

We attempted to conduct a moderator analysis to assess to what extent the results of the meta‐analyses vary by underlying factors related to the programme context and design, such as do effects of PES programmes vary significantly depending on the region in which they are implemented. We specified potential moderating variables for investigationt in the protocol and section 3.10.1. However, we did not identify a sufficient number of studies and variety of contexts to conduct such analyses. Our largest meta‐analysis comprises eight studies, covering four PES programmes from two different countries. As a result, we cannot formally test the effects of different moderating variables on programme outcomes. However, we explore some potential moderating factors in the qualitative synthesis below.

### 6.7 Qualitative synthesis

#### 6.7.1 Included qualitative evidence base

We included a total of 56 studies in the thematic synthesis (Appendix 5). These studies cover all but one of the 18 PES programmes. However, the amount of qualitative evidence varies per study. For programmes such as Malawi's ICRAF experiment and China's DCBT, we only included a single study in the thematic synthesis while other PES programmes, in particular China's SLPC and Costa Rica's PSA, feature 10 studies. Table 5 below illustrates the spread of studies included in the qualitative synthesis per PES programme. The results of the thematic synthesis presented here therefore reflect a configuration of data across different programmes, each of which contributes a different amount of evidence. Reported results are therefore not necessarily applicable to each individual programme.

**Table 5:** Spread of studies included in the qualitative synthesis per PES programme

PES programme		# of qualitative studies
China	SLCP	10
	PLDL	1
	DCBT	1
Costa Rica	PSA	10
	RISEMP	1
Mexico	PSAH	4
	MBCF	2
	PESL	1
Ecuador	Socio Bosque	6
Columbia	Silvopastoral Project	4
Mozambique	Nhambita PES project	4
Cambodia	PES	3
Nepal	REDD	3
Tanzania	EPWS	2
Uganda	PES	1
Cambodia	Conservation agreement	1
Malawi	ICRAF	1
Brazil	PAS	1

Abbreviations: DCBT, Desertification Combating Program around Beijing and Tianjin; EPWS, Equitable Payment for Watershed Services; MBCF, Monarch Butterﬂy Conservation Fund; PES, payment for environmental service; PESL, Programa Especial de la Selva Lacandona; PLDL, Paddy Land‐to‐Dry Land; PSA, Programa de Pagos por Servicios Ambientales; PSAH, Payments for Hydrological Services Program; REDD, Reducing Emissions from Deforestation and Forest Degradation; RISEMP, Regional Integrated Silvopastoral Approaches to Ecosystem Management Project; SLCP, Sloping Land Conversion Program.

The 56 included studies span a range of study designs and are dominated by descriptive studies, with only 16 studies conducting in‐depth qualitative data collection and analysis. The descriptive studies are made up of 22 process evaluations of PES programmes and 16 descriptive quantitative study designs. Two included studies applied explicit mixed‐methods research designs.

The included process evaluations combined quantitative and qualitative data to investigate the implementation of the programmes. They thereby conducted observational analyses to describe the status of a programme and whether it encountered implementations challenges and successes. The descriptive quantitative study designs applied survey methodologies and regression analyses to provide correlational data on programme uptake and design. These studies focussed heavily on investing factors correlated with the uptake of PES programmes and beneficiaries' continued participation. The qualitative study designs can be grouped into studies self‐identifying as qualitative case studies and studies conducting in‐depth interviews of PES participants. The case studies focussed their analysis on the institutional and organisational settings and arrangement of PES programmes and how these affected governance and management issues. The studies conducting in‐depth interviews largely were concerned with investigating PES participants' perceptions of the programmes. In addition, we also used qualitative data reported in the included impact evaluations in the meta‐analysis where this information was available. All of the included studies were subject to inductive coding on EPPI‐Reviewer 4. For two programmes, China's SLPC and Costa Rica's PSA we reached data saturation in coding after completing 10 studies each.

#### 6.7.2 Critical appraisal of studies included in the qualitative synthesis

**Figure 14:**
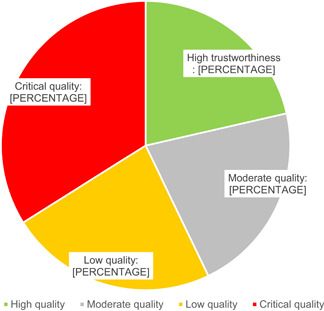
Summary of overall critical appraisal ratings across studies included in the qualitative synthesis [Color figure can be viewed at wileyonlinelibrary.com]

**Figure 15:**
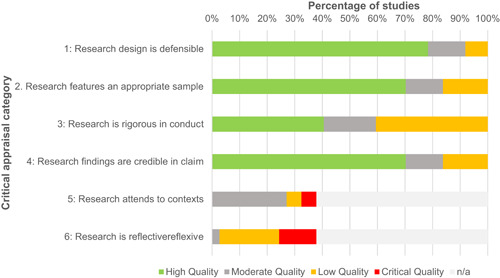
Critical appraisal category ratings across studies included in the qualitative synthesis (excludes studies of critical quality) [Color figure can be viewed at wileyonlinelibrary.com]

All studies included in the qualitative synthesis were critically appraised for the trustworthiness of their contribution to the thematic synthesis. We rated studies on a scale from high quality, to moderate, low and critical trustworthiness using a predefined critical appraisal tool for qualitative studies, descriptive quantitative studies and process evaluations (see Section 3.7 and Appendix 3). Figure 14 provides the results of the critical appraisal on aggregate while Figure 15 presents the breakdown of appraisal ratings per appraisal category. Last, Appendix 6 provides the detailed critical appraisal ratings per study.

Overall, the trustworthiness of the studies included in the qualitative synthesis is low. Of 56 included studies, more than half (57%) are of either critical (34%) or of low trustworthiness (23%). Only 22% of studies were rated of high trustworthiness with the remaining 21% being assessed as of moderate trustworthiness. While these results are more encouraging than the risk of bias results for the impact evaluations reported in Section 4.4, it still leaves the majority of the included evidence base as of low trustworthiness—a finding which needs to be remembered when interpreting the results of the qualitative synthesis.

The drivers of this poor quality of the included evidence base stem from 19 studies that were rated as of critical trustworthiness and 13 studies rated of low trustworthiness. Eight‐four percent of studies (*n* = 16) rated as critical trustworthiness either did not report the collected primary data, did not link primary data to studies' findings, or did not apply a research design that fit the research question and objective. The remaining three studies were rated as critical due to an absence of information on the conduct of the empirical research.

For the 13 studies rated of low trustworthiness, all but two (*n* = 11) only provided most basic information about the research conduct, for example, not illustrating the applied research instruments. In addition, almost half of the studies (*n* = 6) did not illustrate how the identified sample of participants was relevant to collect rich and detailed data on the investigated research question. Studies rated of a low trustworthiness further were subject to methodological concerns of varying degree on the link between the reported data and stated research findings and conclusions (*n* = 7) and the fitness of the applied study design to answer all of the specified research objectives and questions (*n* = 4).

Studies rated of moderate trustworthiness overall only had minor quality concerns with the patterns of quality similar to the low trustworthiness studies above. Of all appraisal categories all but two moderate rated studies (*n* = 10) were subject to some reservations regarding the rigour of the conduct of the research as well as the chose sample of participants. Last, for the 12 studies rated of high trustworthiness, all but one received a high trust rating in each appraisal domain.

Figure 15 below reiterates the above overall critical appraisal ratings for the included qualitative studies. It excludes the 19 studies that were rated of critical trustworthiness. Investigating only the body of evidence for which all appraisal categories could be completed, Figure 15 indicates that 41% of included studies still scored poorly in terms of the rigour of the research conduct. Further, and particular concerning for qualitaitive research, none of the studies was rated of high trust for either “attention to context” or “deep reflection”. In contrast, the remaining studies show trustworthy critical appraisal ratings in relation to the defensibility of the research design (78% of studies), the appropriateness of the included sample (70% of studies) and the credibility of the studies findings (70% of studies).

#### 6.7.3 Results of the qualitative synthesis

Coding the 56 included studies for data related to mechanisms, design, implementation and contexts factors influencing the effects of PES programmes, we identify a total of 107 inductive codes. These codes were then organised and configured into 21 descriptive themes. These descriptive themes on average comprise five inductive codes.^17^ Following the identification of the 21 descriptive themes, these were then further organised and configured into six analytical themes. These analytical themes related to mechanisms, design, implementation and contexts factors influencing the effects of PES programmes and present the unit of analysis in this thematic synthesis. These are discussed in more detail below. Table 6 provides an overview of the generation of analytical themes and descriptive themes.

**Table 6:** Overview of the generation of analytical and descriptive themes

Descriptive themes based on the inductive coding of primary studies' findings	Analytical themes derived from the configuration of descriptive themes
▪ Targeting at areas with high‐risk of deforestation ▪ Targeting at low‐income groups ▪ Targeting at locality (e.g., access to roads, slope, type of forest)	**Analytical theme 1: Targeting (design)** PES programmes need to be carefully targeted at the most relevant programme participants to maximise environmental and social outcomes. Targeting is of particular importance to support social outcomes such as poverty reduction and equity objectives.
▪ Awareness of the programme ▪ Design of informational materials and campaigns ▪ Ease of access/signing up the programme ▪ Structure of the programme/contract ▪ Technical assistance	**Analytical theme 2: Participation in the programme (implementation)** Participation in PES programmes presents a key barrier to effective programme implementation. Participation is hindered by a lack of awareness and understanding of PES programmes with technical assistance and more relevant information campaigns presenting possible remedies.
▪ Governance structures and ownership ▪ Institution building as a programme mechanism ▪ Trust as a facilitator of programme success	**Analytical theme 3: Programme governance and institutions building (design)** PES programmes require strong governance structures within the communities in which they are implemented in order to monitor and ensure compliance and behaviour change. What is more, creating these governance structures presents a key mechanism through which programmes can achieve social objectives by supporting the building of local institutions and development structures^a^
Factors of adoption: ▪ Existing levels of income ▪ Size of the land ▪ Availability of labour ▪ The opportunity cost of participation ▪ Social norms ▪ State and impact of environmental degradation	**Analytical theme 4**: **Factors to determine programme take up (context)** A range of factors determine the uptake of PES programmes. The most common factors for adoption identified referred to: existing levels of income, size of the land, availability of labour, the opportunity cost of participation, social norms and capital, and the state of the ecosystem service targeted
▪ Existing perceptions of nature and conversation ▪ Changing perceptions of nature and conversation ▪ State and impact of environmental degradation	**Analytical theme 5: Perception of nature (context/design)** Perceptions of nature influence the design and relevance of PES programmes. While existing support for environmental protection supports programme implementation, there is little empirical evidence that financial incentives lead to a monetisation of environmental behaviour

Abbreviations: PES, payment for environmental service.

^a^ This mechansism is largely identified in community‐level PES programmes rather than individual‐level programmes.

##### 6.7.3.1 Analytical theme 1: Targeting (design)

**PES programmes need to be carefully targeted at the most relevant programme participants to support environmental and social outcomes**. Targeting is of particular relevance to support social outcomes such as poverty reduction and equity objectives.

The effective and relevant targeting of programme participants emerged as a key design criterion of PES programmes in the thematic synthesis. For example, qualitative research on Mexico's PSAH (Alix‐Garcia et al., 2009), Ecuador's Socio Bosque (Murtinho & Hayes, 2017) and Tanzania's EPWS programme (Branca, 2011) suggests that programme effects were supported by the design of effective targeting criteria to identify programme participants. In the case of PSAH, participant targeting emerged from a simple location‐based criterion to a point‐based system weighted per applicant assessing social, economic and environmental factors in much detail, which led to a more accurate programme targeting.

The thematic synthesis suggests that the alignment of the programme targeting approach with the main objectives of the programme is central. If the programme targets a decrease in deforestation, participants and areas at the highest risk of deforestation need to be included. Research on the Mexico's PELS (Costedoat, 2015) and Costa Rica's PSA (Arriagada, 2012), for example, indicates that programmes can struggle to cover areas at the highest risk of deforestation. This risks creating a situation in which payments are made for the conversation of forests that were at a low risk of deforestation in the first place, potentially challenging the additionality of the PES programme.

Targeting of programme participants is particular important when the PES design attempts to combine environmental and social objectives. In order to ensure the inclusion of the most marginalised and vulnerable groups, who could benefit most from the social objective of the PES programmes, deliberate efforts and design considerations have to be included in the programme. Qualitative research on Nepal's REDD+, Tanzania's EPWS, Mexico's PSAH and Ecuador's Socio Bosque, underlines that without direct targeting participation of low‐income and marginalised groups in the PES programmes remained low.

Other targeting criteria frequently reported in the evidence‐base refer to criteria related to the accessibility of the programme area (e.g., access to roads), the geography of the programme area (e.g., sloping land, type of forest), and the use of auctions as a promising mechanism to identify relevant programme participants and their revealed willingness to pay for environmental services (Alix‐Garcia et al., 2009; Jack et al., 2016).

##### 6.7.3.2 Analytical themes 2: Participation in the programme (implementation)

**Full participation in PES programmes presents a key factor in effective programme implementation. Participation is hindered by a lack of awareness and understanding of PES programmes with technical assistance and more relevant and extensive information campaigns presenting possible remedies**.

The thematic synthesis identified a range of themes highlighting barriers to participants taking part in the PES programmes. These barriers relate in particular to a lack of awareness and effective information sharing about the programme and difficulties in signing up to the programme and understanding its conditions and structures. For example, in Costa Rica's PSA programme, a key reason for nonparticipation of landholders was a lack of information about the programmes leading to participants not being aware about their eligibility (Bossel, 2013; Schoffelen, 2013). The same finding emerged in Uganda where two‐thirds of eligible participants who did not enrol were unaware of the programme or did not know what it was about (Jayachandran et al., 2016).

Moreover, throughout the synthesis, there was a common theme that, even when participants enrol in the PES programme, they do not fully understand its objective and conditionality. Qualitative data from participants' interviews across a range of contexts—Costa Rica, Mexico, Uganda, Ecuador, China, Cambodia—indicate that a large number of participant cannot fully explain what the PES programme is for, and why and how payments are made. This risks undermining the ownership and sustainability of programmes something discussed in more detail in theme 6.

Combining both themes above, that is, a lack of awareness of PES programmes as well as a lack of understanding the nature and design of programmes can allow more advantaged groups to have preferential access to the programmes. The qualitative synthesis indicates that groups with higher social capital and education seem to be in a better position to participate in PES programmes; though, there is insufficient data on how this affects programme outcomes and important exceptions to this observation apply (e.g., Pagiola et al., 2010).

Throughout the synthesis, two main facilitators for more equitable and increased access to programmes were identified: a redesign of information campaigns that better target groups with lower levels of education and limited social networks (e.g., Chandra, 2015; Jayachandran et al., 2016); and technical assistance and capacity‐building to support participants in understanding the structure of the PES programme and to implement its objective and conditionality (e.g., Garbach et al., 2012; Hayes, 2012).

##### 6.7.3.3 Analytical theme 3: Programme governance and institutions building (design)

**PES programmes require strong governance structures within the communities in which they are implemented in order to monitor and ensure compliance and behaviour change**. What is more, creating these governance structures presents a key mechanism through which programmes can achieve social objectives by supporting the building of local institutions and development structures.^18^

Strong programme governance structures emerged as a key theme in the thematic synthesis. Programme governance is required to monitor and support the compliance of participants with the PES conditionality as well as to build trust in the PES programme. Hedge et al. (2015) and Sims et al. (2014), for example, show how a single missed or inaccurate payment can drastically undermine support for PES programme. Likewise, qualitative research frequently indicates that a large number of eligible programme participants do not sign up for the programme immediately and rather observes for some duration of the programme whether implementers and funders are trustworthy (Calle, 2009; Mudaca, 2015). Building trust between programme implementers and participants presented a reoccurring subtheme within the qualitative evidence on PES governance structures; and transparent management, reliable implementation and constant stakeholder engagement were identified as contributing practices in this regard.

In order to support strong and acceptable governance structures, a range of programmes in, for example, Tanzania, Nepal and Uganda, rely on existing community‐based organisations. This practice is reported to support local ownership of and participation in the programme. It also can serve as a more relevant conflict resolution mechanism, but is unlikely to eliminate conflict over the PES resources altogether, which should be expected in the implementation in any PES programme—a finding consistent throughout the qualitative synthesis.

However, in addition to governance structures serving as a facilitator of PES programmes, the creation of local programme governance structures presents a key mechanism through which programmes can achieve social objectives by supporting the building of local institutions and development structures. In a range of different PES programmes across contexts—Columbia, Ecuador, Nepal, Mozambique and Uganda—the introduction of programme governance structures either strengthened or built new local governance structures. This change supported local institutions which then were used as a foundation for additional development projects, as in the case of REDD+ in Nepal and the Nhambita PES project in Mozambique; used to strengthen property rights in the Ugandan PES project; and used to support community activism and cohesion more broadly as observed in the Ecuadorian Socio Bosque programme and the Silvopastoral Project in Columbia.

##### 6.7.3.4 Analytical theme 4: Factors to determine programme take up (context)

**A range of factors determine the uptake of PES programmes. The most common factors for adoption identified referred to existing levels of income, size of the land, availability of labour, the opportunity cost of participation, social norms and capital, and the state of the ecosystem service targeted**.

Our thematic synthesis identified a large range of factors determining programme take up reported in the qualitative evidence (33 in total). Configuring the data across these factors, we identify six factors with the richest evidence base. First, the existing level of income is a key determinant of programme participation across contexts. PES programmes in Mozambique, Costa Rica, Ecuador, Uganda, Columbia, China, Brazil, Tanzania and Cambodia each report this factor. There is convergence of data that participants with a higher level of existing income and a more diversified income base are more likely to take up PES programmes (e.g., Beauchamp, 2015; Hedge, 2012; Yuan, 2017).

This theme overlaps with a number of related themes. For example, a range of studies investigate participants' opportunity costs and dependence on environmental resources rather than level of income. Again, the qualitative data indicates that participants that are less well‐off, that is, depend to a larger extent on natural resources for their livelihoods and thus have a higher opportunity cost to joining the programme, are less likely to take up the programme (e.g., Hedge, 2015; Jones, 2017; Jayachandran et al., 2016). Likewise, the size of the existing land and the availability of household labour are positively related to the uptake of PES programmes across contexts: households with larger amounts of land are more likely to participate in PES programmes, arguably given their lower opportunity cost (e.g., Arriagada, 2015; Schoffelen, 2013); and households with more additional labour also are more likely to take up PES programmes, in particular where there are opportunities to engage in wage labour actives as households shift to nonagricultural income‐generating activities (e.g., Garbach, 2012; Yao, 2010).

A different factor of adoption identified in the thematic synthesis referred to existing social norms and capital. The qualitative evidence indicates that the uptake of new agricultural practices and environmental behaviours is highly receptive to social influence and learning. For example, PES programmes across contexts from Mozambique, to Columbia, and China observed the role of social influence in farmers' adoption of land‐use change techniques required by PES programmes. PES participants observed how trusted social sources fared with signing up to the programme and only after the programme and its associated practices had been validated as trustworthy did participants fully engage (e.g., Calle, 2009; Mudaca, 2015). Peer‐ and social‐learning activities such as community workshops, participatory rural appraisal, and ongoing field and mentoring visited were also reported as effective means to increase programme take‐up through establishing social norms and capital of prospective and current participants.

Lastly, the existing environmental situation and how it affects social and economic activity also served as a strong factor of adaption, which is discussed more in the next theme.

##### 6.7.3.5 Analytical theme 5: Perception of nature (context/design)

**Perceptions of nature influence the design and relevance of PES programmes. While existing support for environmental protection supports programme implementation, there is little empirical evidence that financial incentives lead to a monetisation of environmental behaviour**.

Existing support for and practices related to conserving the environment emerged as a key facilitator for PES programmes in the synthesis. Somewhat unsurprisingly, where communities have already organised themselves to protect and conserve their natural resources, this supports the implementation of PES programmes (e.g., Jones, 2017; Krause, 2013). The same holds true where prospective participants have positive attitudes towards environmental protection (Arriagada, 2008b; Chandara, 2012). However, the motivations behind these attitudes and behaviours differ broadly across intrinsic and extrinsic factors. In some contexts, for example, Ecuador and Nepal, intrinsic motivation is reported as the main reason behind positive attitudes towards conversation. In other contexts, for example, Columbia, China, Uganda, extrinsic motivations are identified. Such extrinsic motivations are reported where the state of the environmental degradation is so advanced that it negatively affects participants' social and economic livelihoods. Here, support for conversation activities is not so much driven by an altruist motive but rather by self‐interest in the conservation of one's own livelihood.

In addition, the thematic synthesis also investigated whether the introduction of PES might lead to a monetisation of environmental behaviour. That is, by providing financial incentives to nurture environmental behaviours, such behaviours become dependent on financial resources in the long‐run. Such dependence can undermine more intrinsic motivation for environmental behaviours and thus pose a challenge to conversation activities in the long‐run. In our review of the qualitative literature, we only identified a single study providing empirical data on the question of monetisation (Chervier, 2017b). While this study does indeed provide evidence to substantiate this risk, the overall empirical evidence base is too small to comment on this issue. There is currently an absence of evidence to answer this question.

##### 6.7.3.6 Analytical theme 6: Perceptions of PES (context)

**The majority of PES programmes was positively received by programme participants. However, a share of participants indicates to revert to old practices in the absence of the PES programme**.

The thematic synthesis included a range of themes based on qualitative evaluations of PES programme perceptions and acceptability. The large majority of qualitative evaluations found PES programmes to be perceived positively by programme participants. This includes PES programmes in Mozambique (Spiric, 2009), Costa Rica (World Bank, 2008), Mexico (Alix‐Garcia et al., 2015b), China (Uchida, 2009; Zheng, 2013), Columbia (Hayes, 2012), Cambodia (Clements, 2015) and Tanzania (Lopa, 2012). While these qualitative evaluation designs have to be treated with caution, the available data broadly lends support to the acceptability of PES as a mechanism for environmental protection in LMICs. All in all, participants seem to be satisfied with programme design, implementation and benefits received.

While the above finding could lend support to the long‐term effectiveness of PES programmes, a linked theme mitigates this somewhat. In three studies of large‐scale PES programmes, a substantive share of participants indicated that the adopted environmental practices (i.e., sloping land conversation, forest conversation and silvopastoral practices) would not be sustained were the subsidies for them withdrawn. In Columbia, less than half of the participants (41%) stated that they were likely to continue the silvopastoral practices (Hayes, 2012), while in Ecuador the majority of participants (57%) saw no benefits in programme participation and the continued enrolment of their forest (Krause, 2013). A similar finding was identified in China, where only 30% of participants indicated to be willing to continue converting farm land into forests were payments discontinued. It should be cautioned, however, that these findings are based on participants' perceptions and cannot be regarded as longitudinal evidence of programme effects.

**Non‐themes**:

The below variables were targeted as deductive themes in the qualitative synthesis, but we did not identify sufficient empirical research results to include them in the synthesis:

▪ Equity related themes
▪ Gendered effects of PES programmes and designs
▪ PES contract structure
▪ Type of participation (e.g., voluntary/top‐down).

Comments on the importance of these themes are therefore, currently, not based on a systematic and synthesised evidence base and any recommendation regarding their implications is speculative.

### 6.8 Integrated synthesis

In the integrated synthesis, we envisaged to bring the results from the meta‐analyes and the qualitative synthesis together in order to unpack the impact (or lack therefore) of PES programmes along the causal chain provided in Figure 1. This configuration of the two types of syntheses could have supported us in unpacking and explicating the results of the meta‐analysis and to investigate how and why PES programmes might work or fail to work. Unfortunately, the results of the meta‐analyses are inconclusive due to the poor quality of the available evidence. At this stage, we simply cannot assess whether PES are an effective conservation, climate change mitigation and poverty reduction approach or not. Due to the lack of tangible empirical review findings on the overall impact of PES, it is not possible to integrate the results of both types of syntheses.

### 6.9 Cost analysis

We systematically extracted data on programme cost and cost‐effectiveness from all included studies. This refers to cost data on total programme cost and information on the size of PES payments and total amount distributed. Of all 18 programmes, we identified data on total programme cost and/or cost‐effectiveness for seven PES programmes (reported in 10 studies). The cost data and analysis available are highly heterogenous, with the most common form of analysis being a simple cost benefit ratio using indicators of programme costs against the social cost of carbon for estimated or measured conservation outcomes (*n* = 4). We therefore only provide a narrative overview of reported cost information here and Table 8 below provides an overview of the extracted cost data and the studies' conclusions regarding cost‐effectiveness.

**Table 7:** GRADE Evidence profile

	Quality assessment					GRADE result	
Effects of PES on	No. of studies (design)	Limitations	Inconsistency	Indirectness	Imprecision	Pooled effect	Quality
*Socioeconomic outcomes*							
Socioeconomic outcomes combined	14 (2 RCTs)	Very serious risk of bias	Serious inconsistency	Very serious indirectness	Serious imprecision	0.15 [0.03, 0.27]	⊕○○○ Very low
Total household income	8 (0 RCTs)	Very serious risk of bias	No serious inconsistency	Very serious indirectness	No serious imprecision	SMD 0.25 [0.09, 0.41]	⊕⊕○○ Low
Household income from nonagricultural sources	7 (0 RCTs)	Very serious risk of bias	Serious inconsistency	Serious indirectness	Serious imprecision	SMD 0.05 [−0.03, 0.13]	⊕○○○ Very low
Agricultural income	7 (1 RCTs)	Serious risk of bias	Very serious inconsistency	Very serious indirectness	Serious imprecision	SMD 0.11 [−0.06, 0.29]	⊕○○○ Very low
Asset indexes	3 (1 RCTs)	No serious risk of bias	No serious inconsistency	No serious indirectness	Very serious imprecision	SMD 0.02 [−0.13, 0.17]	⊕○○○ Very low
*Environmental outcomes*							
Environmental outcomes combined	11 (1 RCT)	Very serious risk of bias	No serious inconsistency	No serious indirectness	Serious imprecision	SMD 0.21 [0.09, 0,33]	⊕⊕○○ Low
Forest cover	5 (1 RCTs)	No serious risk of bias	Serious inconsistency	No serious indirectness	Serious imprecision	SMD 0.32 [0.10, 0.55]	⊕⊕○○ Low
Deforestation	6 (0 RCTs)	Very serious risk of bias	No serious inconsistency	No serious indirectness	Serious imprecision	SMD −0.12 [−0.19, −0.05]	⊕⊕○○ Low

Abbreviations: PES, payment for environmental service; SMD, standardised mean difference.

**Table 8:** Overview of cost data extracted from studies

PES programme/study	Cost information	Formal cost‐effectiveness analysis conducted?	Authors' comment on cost‐effectiveness
PAS Costa Rica Arriagada (2012)	‐ Administrative cost data ‐ Value of land protected due to PES ‐ PES funds distributed	**No**, only total cost of programme provided to obtain additional forest cover. Calculation based on U.S. dollars per hectare gained per year over the study period	**Unclear**: Estimation of cost between $255 and $382 per year per hectare of additional forest
PSAH Mexico Alix‐Garcia et al (2012)	‐ Participation costs for applicants ‐ Implementation cost are provided pesos per year based on survey data but	**No**, assesses *participation* costs for applicants on nonfinancial specifics such as days required to apply for participation **Yes**, assesses the *implementation* costs on a suite of indicators for labour costs to PES payment	**Not cost‐effective**: In summary, by most of the possible measures, the available surplus of the programme beyond covering costs is quite small
PSAH Mexico Sims (2017)	‐ Budget for PES and protected area ‐ Mean predicted locality production revenues for each policy	**No**, relies on comparison of budgetary data **Yes**, conducts formal regression analysis on mean predicted locality production revenues for each policy	**Not cost‐effective**: PES was likely significantly more expensive to implement per hectare than a protected area. PES is not necessarily more cost‐effective simply because it is an incentive‐based rather than command and control conservation mechanism
EPWS Tanzania John (2012); Kwayu (2017); Lokina (2016)	‐ Administrative data of PES programme	**No**, only provides an overview of the total cost of the programme	**Unclear**: Following the initial feasibility assessment phase, which required an investment amounting to US$220,000 (CARE & WWF, 2007c) cited in John (2012), project implementation costs from 2008 are estimated at US$1.2 million covering negotiation, training and payments to farmers
PES Uganda Jayachandran (2017)	‐ Administrative data of programme cost ‐ PES funds distributed ‐ current market price of carbon	**Yes**, back of the envelop assessment of cost‐effectiveness in terms of averted carbon dioxide (CO_2_) emissions	**Cost‐effective**: We estimate that for each $0.25 in payments, or $0.57 in total programme costs, a metric ton (hereafter, ton) of CO_2_ emissions due to deforestation was delayed. The social benefit of the delayed CO_2_ emissions is then $1.11 per ton, or roughly two times the $0.57 programme cost
SLCP China Zheng (2013)	‐ Implementation costs ‐ Projected revenue	**Yes**, simple cost ratio between programme's benefits (the value of increased water yield and improved water quality) and programme's costs (the opportunity costs of the upstream farmers plus transaction cost)	**Cost‐effective**: Our analysis suggests that overall benefits of the PLDL programme exceed the costs of programme implementation. Overall, the benefit–cost ratio of the programme is 1.5
ICRAF Malawi Jack (2017)	‐ Total cost per PES contract ‐ Current market price of carbon	**Yes**, simple cost ratio between per contract costs and programme benefits measured in social cost of carbon	**Not cost‐effective**: Using a social cost of carbon of US$21, this implies sequestration benefits of US$0.26 per tree at the end of the contract. If carbon sequestration is the only social benefit generated by the programme, then there are more cost effective ways to sequester carbon
PAS Brazil Simonet (2017)	‐ Estimate of the number of tons of CO_2_ emissions that have been averted ‐ Estimate to calculate the project costs per ton of averted CO_2_ emissions	**Yes**, simple cost ratio between programme costs and programme benefits measured in social cost of carbon	**Unclear**: Assuming unchanged deforestation rates until the end of the project (2017), the total discounted project costs over the 2012–2017 period are 2,021,859 USD (5,777 USD per participant) while the total avoided emissions reach 3,628,166 tCO_2_ (10,366 tCO_2_ per participant). Over the 5 years of the project, the total cost of the project is thus 0.56 USD per ton of CO_2_

Overall, the reported cost‐effectiveness of the included programmes is mixed and appear context specific. The cost‐effectiveness analysis of both the ICRAF programme in Malawi, which measured against its impact on carbon sequestration, and the PSAH conclude the PES programmes are not cost‐effective. In addition, Sims and Alix‐Garcia (2017) compare the effects of the PSAH PES scheme against a different environmental intervention (a protected area) and find the PES programme to be comparatively less cost‐effective. This is the only reported case in our review where the impacts of a PES programme are compared against a different environmental intervention.

In three programmes the reported cost data does not allow for conclusions regarding programme cost‐effectiveness. The PAS programme in Costa Rica is estimated to spend between $255 and $382 per year per hectare of additional forest, while for the EPWS programme in Tanzania only the total cost of the programme is reported. Similarly, for the PAS PES scheme in Brazil the calculation provided establishes the total cost of the programme, estimated at 0.56 USD per ton of CO_2_, without assessing cost‐effectiveness.

In studies from China and Uganda PES programmes are found to be cost‐effective. Using a simple cost‐benefit ratio, the SLPC programme in China is found to have a positive ratio of programme benefits exceeding programme cost by a factor of 1.5, although this applies to socioeconomic outcomes only. The Ugandan PES scheme also is evaluated using a simple cost‐benefit ratio. Here, the authors estimate that the social benefit of the delayed CO_2_ emissions due to the programme amounts to $1.11 per ton, or roughly two times the $0.57 programme cost.

**In summary**, the evidence on cost‐effectiveness is rather limited and consists of different types of estimates. The results available suggests a mixed picture, with authors finding PES to be cost effective in some contexts but not in others. Given the small sample of studies that this observational analysis is based on we therefore cannot conclude whether PES is a cost‐effective approach to support environmental and socioeconomic outcomes or not.

## 7 DISCUSSION

The findings presented in this report summarise the evidence on the effects of Payment for Environmental Services on environmental and socioeconomic outcomes in L&MICs. We identified 44 experimental and quasiexperimental studies evaluating the effect of 18 unique programmes. We also included an additional 56 documents with qualitative studies, process evaluations and project descriptions associated with the 18 PES programmes covered in the impact evaluations. The 18 programmes took place in 12 different countries across regions. Eight programmes took place in the Latin America and the Caribbean region (Brazil, Colombia, Costa Rica, Ecuador, Mexico), five in East Asia and Pacific (China, Cambodia), four in Sub‐Saharan Africa (Malawi, Mozambique, Tanzania, Uganda) and one in South Asia (Nepal). This chapter provides a summary and discussion of the findings of the review, and the average estimates and overall quality of evidence is reported for all included primary outcomes in Table 8 below.

### 7.1 Summary of findings

#### 7.1.1 Socioeconomic outcomes

In total, we are able to provide synthesised evidence on the effects of PES programmes on four socioeconomic outcomes: total household income, household income from nonagricultural sources, on agricultural income and on asset indexes. These meta‐analyses cover eight of the 18 individual PES programmes, are subject to a high degree of heterogeneity, and are based on a body of research that suffers from a very serious risk of bias.

Keeping the above caveats in mind, the results of the meta‐analysis overall suggest that PES programmes have, at best, *mixed effects on socioeconomic outcomes*. Of four meta‐analysis conducted to assess different socioeconomic outcomes, PES programme were only found to have a clear positive effect on measures of total household income. In contrast, PES had no clear impact on household income from nonagricultural sources, on agricultural income, and on asset indexes.

In detail, we identified the following impacts of PES on socioeconomic outcomes:

##### 7.1.1.1 Effects of PES on total household income

Synthesising the effects of four PES programmes evaluated in eight studies, we identified an ***increase in total household income*** of 0.25 SMD (95% CI [0.09, 0.41]), which indicates an increase in income for households taking part in PES programmes when compared to a control group who were not receiving the PES programme. Overall, this finding is based on *low quality of evidence*.

##### 7.1.1.2 Effects of PES on household income from nonagricultural sources

Synthesising the effects of three PES programmes evaluated in seven studies, *we can detect no overall increase in household income from nonagricultural sources* (0.05 SMD, 95% CI [−0.03, 0.13]). Overall, this finding is based on *very low quality evidence*.

##### 7.1.1.3 Effects of PES on agricultural income

Synthesising the effects of three PES programmes evaluated in seven studies, *we can detect no overall impact on agricultural income* (SMD = 0.11, 95% CI [−0.06, 0.29]). Overall, this finding is based on *very low quality evidence*.

##### 7.1.1.4 Effects of PES on asset indexes

Synthesising the effects of three PES programmes evaluated in three studies, *we can detect no overall impact on asset indexes* (SMD = 0.04, 95% CI [−0.12, 0.20]). Overall, this finding is based on *very low‐quality evidence*.

##### 7.1.1.5 Strength of evidence

All of the above review findings are based on an evidence base that is rated as being of very low or low quality, according to GRADE criteria (Table 7). Therefore the review findings and their applicability should be interpreted with caution. Four key issues are compromising the evidence in particular:

- First, the CIs are wide and cross the line of no effect for all outcomes apart from overall household income.
- Second, the effects are largely driven by multiple studies drawing on independent samples to evaluate the effect of three large programmes in China. In all meta‐analyses apart from the one assessing effects on household assets there is only one estimate in each which are from a different context.
- Third, most of the studies suffer from high or critical risk of bias, including all the studies of programmes in China. The one exception to this is the meta‐analysis of household income from agricultural sources which includes a low risk of bias experimental study of a PES pilot in Malawi (Jack and Santos, 2017). This study finds no difference between treatment and comparison groups.
- Fourth, the effects on the different measures of income suffer from serious indirectness. The underlying income data used many of the studies comes from self‐reported and recalled (up to 10 years) income estimates by PES participants. This type of income data is highly unreliable and cannot be regarded as a reliable proxy for actual household income.

#### 7.1.2 Environmental outcomes

We synthesised evidence on the effects of PES programmes on forest cover (expansion of forested land) and deforestation (forest loss). These meta‐analyses include data from five of the 18 individual PES programmes, are subject to a high degree of heterogeneity, and are based on a body of research that suffers from serious risk of bias.

Keeping the above caveats in mind, the results of the meta‐analysis overall suggest that PES programmes may have *positive effects on environmental outcomes in some contexts*. The meta‐analyses identify an improvement in deforestation rates and a moderate improvement in forest cover.

In detail, we identified the following impacts of PES on environmental outcomes:

##### 7.1.2.1 Effects of PES on forest cover

Synthesising the effects of five PES programmes evaluated in five studies, we identified an *increase in forest cover* of SMD = 0.35 (95% CI [0.10, 0.55]), which translates into a greater expected forest cover in areas subject to a PES programme when compared to a control area which was not receiving the PES programme. Overall, this finding is based on *low quality of evidence*.

##### 7.1.2.2 Effects of PES on deforestation

Synthesising the effects of four PES programmes evaluated in six studies, we identified an *improvement in deforestation rates* of SMD = −0.12 (95% CI [−0.19, −0.05]), which translates into a decrease in deforestation in areas subject to a PES programmes when compared with a control area which was not receiving the PES programme. Overall, this finding is based on *low quality evidence*.

##### 7.1.2.3 Strength of the evidence

All of the above review findings are based on an evidence base that is rated as of very low or low quality (Table 7), again suggesting caution when interpreting the review findings and their applicability. There are two key issues in particular that are compromising the quality of the evidence:

- First, as with the evidence on socioeconomic outcomes, the results of the meta‐analyses suffer from imprecision, although the average effects are more precise and do not cross the line of no effect.
- Second, most of the studies suffer from high or critical risk of bias. However, while issues with risk of bias remain overall, the evidence of beneficial effects is at least to some extent driven by studies with lower risk of bias, including the experimental study of PES in Uganda (Jayachandran et al., 2017). But at the same time Alix‐Garcia et al. (2015a), which is among the more robust quasiexperimental studies we included, find a smaller although positive effect of PSAH on forest cover in Mexico.

#### 7.1.3 Design, implementation and context of PES programmes: Results from the qualitative synthesis

We identified six analytic themes from the qualitative data in terms of the importance of design, implementation and context factors influencing effectiveness of PES programmes. As in the meta‐analysis, the included evidence base is of low quality with more than half of all studies (57%) rates as of either critical (34%) or of low quality (23).

In terms of PES programme design and implementation, the thematic synthesis found the following:

- PES programmes need to be *carefully targeted* at the most relevant programme participants to maximise environmental and social outcomes. Targeting is of particular importance to support social outcomes such as poverty reduction and equity objectives.
- PES programmes *require strong governance structures* within the communities in which they are implemented in order to monitor and ensure compliance and behaviour change. What is more, creating these governance structures presents a key mechanism through which programmes can achieve social objectives by supporting the building of local institutions and development structures.^19^
- *Participation in PES programmes* presents a key factor to support effective programme implementation. Participation is hindered by a lack of awareness and understanding of PES programmes with technical assistance and more relevant information campaigns presenting possible remedies.

In terms of contextual factors affecting PES programme in their performance, the thematic synthesis found the following:

- A range of *factors determine the uptake of PES programmes*. The most common factors for adoption referred to: existing levels of income, size of the land, availability of labour, the opportunity cost of participation, social norms and capital, and the state of the ecosystem service targeted.
  a. These same factors are likely to affect environmental and social outcomes, and thus studies seeking to estimate PES impacts must find ways to control for them.
- *Perceptions of nature* influence the design and relevance of PES programmes. While preprogramme support for environmental protection supports programme implementation, there is little empirical evidence that financial incentives lead to a monetisation of environmental behaviour.^20^

Last, we also attempted to investigate a number of predefined themes in the qualitative synthesis but do not find any systematic evidence in the review. These include qualitative data on gendered effects of PES programmes; relevance and acceptability of different PES contract structures; systematic insights on how different types of participants are affected by PES programmes; and whether the type of participation (e.g., voluntary vs. top‐down) has systematic differences in the relevance and acceptability of PES programmes.

### 7.2 Overall completeness and applicability of evidence

The clearest finding of this review is that the evidence base is too limited in both quantity and quality to be able to confidently establish the effectiveness of PES programmes on environmental and human welfare outcomes. An inability to establish overall effectiveness also means that it is challenging to identify programme design and implementation features that moderate effects. While we do find a number of studies in the qualitative synthesis providing insights into PES design and implementation issues, we cannot formally test the impact of different design and implementation features on programme effectiveness.

The evidence that does exist is focused on a limited set of programmes and therefore limits the generalisability and applicability of the evidence. Taken together, our various meta‐analyses of environmental and socioeconomic outcomes cover nine of 18 and 10 of 18 PES programmes respectively. This leaves us unable to comment on the overall effectiveness of different PES programmes across contexts. The meta‐analyses that we undertake on socioeconomic outcomes are heavily influenced by programme evaluations of the Chinese PES programmes, which are limited in their generalisability to other contexts due to the largely semivoluntary uptake of the programme and relatively large size of the payment.

In addition, the evidence base is often characterised by small studies, without baseline data, that fail to use rigorous methods of analysis. Moreover, the risk of spill‐overs in the form of negative effects on vulnerable populations and displacement of deforestation within land owned by PES participants and to land owned by nonparticipants is well known, but few studies address these spillovers convincingly.

Lastly, the evidence base suffers from a surprising outcome reporting pattern. Despite environmental protection being the primary objective of PES programmes, only 11 of the included 18 programmes (corresponding to 19 out of 44 included studies) measure how the PES programmes affect environmental outcomes. For seven PES programmes, including the large‐scale Chinese PES programmes, there is no attempt to measure their impact on environmental changes or whether conservation objectives have been achieved. As no included study reports the use of a preanalysis plan, it is difficult to establish with certainty whether this is a deliberate attempt to not report particular types of results. But, the availability of panel data sets on forest cover based on satellite data, which was used in many of the most rigorous quasiexperimental studies we reviewed, raise questions as to why this was not used in more studies.

### 7.3 Quality of the evidence

There are serious limitations with the quality of the evidence on PES programmes. Using the GRADE scale to assess the strength of the evidence in this meta‐analysis, we rate the meta‐analyses' results to be based on low to very low quality evidence. Table 7 below provides an overview of the results of the GRADE assessment. Eighty‐two percent of studies suffer from critical (51%) or high (31%) risk of bias. In particular, many studies are limited by small sample size and a lack of baseline data and lack of control for covariates which have been theoretically and empirically shown to be associated with both land use outcomes and PES participation.

Moreover, few studies address spill‐over effects. For an intervention like PES where the risk of spill‐overs are particularly high this is a significant limitation. In addition, there are issues with the quality of reporting and a lack of studies that measure a range of outcomes, including intermediate outcomes, and assessment of implementation. Finally, the usefulness of the existing evidence is compromised by extreme fragmentation of the evidence base. While we extracted data and calculated a large number of effect sizes, relatively few of these could be included in a meta‐analysis because they use such a broad range of different outcome measures.

### 7.4 Limitations and potential biases in the review process

We took a number of steps to limit the potential for bias in the review process, including double screening of studies for inclusion and independent assessment of risk of bias. We did not however have resources for independent data extraction. Instead all data was checked by a second, more senior author. There were a number of included studies that did not contain the necessary data for us to calculate effect sizes and so were not included in our meta‐ analysis. We tried to obtain this information by contacting the author team but in several cases we did not receive a response. Due to a lack of sufficient studies we were also not able to conduct meta‐regressions to explore reasons for heterogeneity or to assess cost‐effectiveness.

### 7.5 Agreements and disagreements with other studies or reviews

Our review is a partial update of Samii et al. (2014). We identify more studies, many of them published the last couple of years. Our findings are similar however, in that issues with quality and quantity of evidence remains a major challenge for the field. Our conclusions are substantively similar, although suggest a slightly larger overall beneficial effect on environmental outcomes.

### 7.6 Deviation from the protocol

There are a few deviations from the protocol of this review (Snilstveit et al., 2018). First, we did not exclude qualitative studies judged at a critical risk of bias from the qualitative synthesis as initially planned. This decision was taken in order to align the use of the critical appraisal ratings with the quantitative risk of bias assessment in which critical studies were not excluded from the meta‐analysis. Second, we had scheduled to conduct a range of moderator analyses as well as potential meta‐regression. The protocol prespecified the variables we intended to use for these analyses. Due to small number of included programmes and contexts in the meta‐analyses, we were not able to conduct these analyses. Third, it was not feasible to construct an integrated syntheses of the meta‐analyses and the qualitative synthesis. As the evidence base is so poor in quality and does not allow us to arrive at reliable conclusions regarding the effectiveness of PES programmes in the meta‐analysis, we are unable to use the results from the qualitative synthesis to unpack and explicate the meta‐analysis results.

## 8 AUTHORS' CONCLUSIONS

This review set out to assess the effect of PES on socioeconomic and environmental outcomes in LMICs. Systematically reviewing over 40 impact evaluation of 18 PES programme and synthesising effect sizes for 11 of these programmes, we cannot establish whether PES are an effective approach to achieve environmental protection and human welfare objectives. In short, the available evidence base does not allow for conclusions on whether PES work or not. Despite the hundreds of millions of dollars dedicated to PES programmes over the last decades, including by bilateral aid agencies, multilateral organisations and LMIC governments, we are currently unable to determine if these are worthwhile investments.

While the limited meta‐analyses which we are able to conduct in this review suggest that, in particular contexts, PES may have small to moderate effects on selected environmental and monetary outcomes, these findings cannot be generalised and remain highly programme‐specific. The evidence base is characterised by quasiexperimental impact evaluations with a high or critical risk of bias. It is fractured, with a lack of common outcome measurement, making it more challenging to draw lessons across contexts. The majority of the evidence base is looking at just three long‐standing programmes in Costa Rica, Mexico and China. We also find that the evidence is skewed towards certain outcomes for certain programmes, with none of the studies from China reporting on effects on environmental outcomes.

Given the findings of our review, the role of deforestation and land‐use change as a source of greenhouse gas emissions and the urgent need to identify effective mitigation strategies, we conclude that the large‐scale implementation of PES is a high‐risk strategy. Our primary conclusion is therefore that there is an urgent need to integrate rigorous impact evaluation with the roll‐out of any new PES programme.

### 8.1 Implications for practice and policy

Our systematic review has a number of general implications for decision‐makers working on the design and implementation of conservation and development programmes such as PES. However, these implications need to be adapted to specific contexts, including by drawing on additional local evidence and expert knowledge to be appropriately translated to recommendations for policy and programme design.

1. *Whether to invest in PES programmes*: The findings of our review suggest reasons to be cautious about investing in the implementation of PES programmes in LMICs. Given the current available evidence base, we do not know whether PES programmes do in fact achieve desired environmental and, in particular, social outcomes. Given the current lack of knowledge on programme effects, and the need for mitigation interventions with transformational effects in the forestry sector, we regard the large‐scale implementation of PES programmes as a high‐risk strategy. That said, our review does not identify evidence of harmful of effects of PES either, which have been reported in a range of other, involuntary, conservation programmes.
2. *Investing in PES programmes with built‐in piloting and evaluation*: There is suggestive evidence that PES may deliver positive effects on both environmental and socioeconomic outcomes in some contexts. But because of the limitations of the existing evidence we suggest careful piloting and evaluation should become a prerequisite when investing in the implementation of a PES programme in a new context. Our review provides evidence that such built‐in of evaluations in the PES programme design is feasible. Specifically, we identified two recent experimental studies, highlighting that randomised programme roll‐out for PES is feasible at least in some contexts.
3. *Targeting PES programmes*: The heterogeneous effects of PES across and within countries highlight the importance of PES programmes being carefully targeted at the programme participants and contexts with the largest potential for environmental and socioeconomic benefits. This targeting design becomes particular important where PES programmes assume socioeconomic objectives such as poverty alleviation. The qualitative synthesis indicates that social objectives of PES programmes are likely to be missed if they are not deliberately designed for. Targeting criteria that the qualitative evidence suggests to enhance the relevance of PES programmes to environmental and social objectives include: targeting at areas with high‐risk of deforestation; targeting at the specific contexts of low‐income groups (e.g., taking the social opportunity cost of programmes into consideration; providing technical assistance; applying point‐based eligibility criteria); and targeting at characteristics of the locality (e.g., type of forests, sloping, proximity of existing infrastructure and industrial development).
4. *PES governance structures as a win‐win strategy*: Based on qualitative evidence, PES governance structures emerge as key design criterion that might be able to support PES as a win‐win strategy for environmental and social objectives. Governance structures are central in ensuring programme implementation and compliance, thereby supporting environmental outcomes; but, at the same time, creating strong local governance structures can also support PES's social objectives by ensuring programmes are accessed by all stakeholders and that benefits are shared equitably.

### 8.2 Implications for research

Addressing the lack of available high quality research can be best addressed in the form of coordinated action by funders, implementing agencies and interdisciplinary research themes. There are two main avenues for improving the impact evaluation evidence base, and we suggest they are pursued in parallel.

1. To develop a common framework for the design and implementation of theory based, mixed methods impact evaluations (White et al., 2009) to be conducted in conjunction with the roll out of new programmes. Such studies should be conducted across multiple contexts to identify generalisable and context specific findings. They should assess effects on a common set of environmental and socioeconomic outcomes, including deforestation, GHG emissions, household income and food security. A common issue with the existing literature is the lack of attention to potential negative spill‐over effects in the form of displacement of deforestation within land owned by PES participants and to land owned by nonparticipants and future studies will need to explicitly address this in their design and implementation to be able to establish with confidence whether programmes have reduced deforestation, for example, or simply relocated it to land not included in the programme. To identify and address potential unintended negative socioeconomic effects studies should draw on existing literature to anticipate and collect data on such outcomes for relevant populations in a particular context, including an integrated approach to assessing effects on gendered inequality (Morgan et al., 2016; Welch et al., 2017). Finally, studies should address a broader range of research questions of importance for policy and practice, including those related to effects on different subpopulations, programme design features, implementation consideration and costs.
2. In addition to an effort to produce ex‐ante impact evaluations in a coordinated manner, there are also opportunities to draw on existing data to assess the effect of programmes that are already ongoing or completed. Several of the included studies combined different econometric techniques, such as PSM and fixed effects panel regressions to evaluate the effect of PES programmes using existing data sets (Alix‐Garcia et al., 2015a; Jones et al., 2017). The University of Maryland hosts a freely available and regularly updated the time‐series Landsat data set which characterise forest extent, loss and gain globally from 2000 to 2017 (Hansen et al., 2013) which could be utilised for such studies. In doing so we suggest researchers consider working in interdisciplinary teams and use the most rigorous analytical techniques available to them (see e.g., Ferraro & Miranda, 2017).

In terms of the available qualitative evidence base, we suggest to focus on a range of weaknesses in the existing evidence base. Future qualitative research should:

1. More systematically invest in the collection and analysis of in‐depth qualitative data when planning and conducting impact evaluations. This is likely to increase the relevance of the evaluations and to facilitate a better understanding of programme mechanisms and design factors. While we identified a relatively large number of process evaluations, these did rarely collect in‐depth qualitative data and were usually conducted after the programme and its evaluation had been designed already.
2. Diversify the research participants to present a more reflective picture of all PES programme participants. There is a lack of qualitative research on the gendered effects of PES programmes; how different societal groups can access and experience PES programmes; and how equity objectives can be fully integrated within PES programme design and implementation.
3. Invest in longitudinal, in‐depth qualitative data. The majority of the included qualitative studies are small‐scale (*n* < 30) and conducted over a short time frame (±6 months). To understand how programme implementation changes and affects participants over time, more longitudinal, in‐depth qualitative data is required.

## ROLES AND RESPONSIBILITIES

Content: Professor Paul Ferraro is a leading expert on environmental economics, including PES. Birte Snilstveit, Laurenz Langer and Jennifer Stevenson have previously worked on evidence gap maps including PES.

Systematic review methods: Birte Snilstveit, Laurenz Langer, Jennifer Stevenson, Josh Polanin and Ian Shemilt have substantial expertise in systematic review methods, including mixed methods systematic reviews. They have all been co‐authors of systematic reviews published in the Cochrane and Campbell libraries.

Statistical analysis: Birte Snilstveit, Laurenz Langer, Jennifer Stevenson, Josh Polanin and Ian Shemilt all have experience of using meta‐analysis in systematic reviews. Dr. Josh Polanin is a research methodologist with significant expertise in statistical analysis, frequently providing expert advice on statistical analysis. Professor Paul Ferraro is an environmental economics with significant expertise in econometrics, including applying econometrics in impact evaluations.

Information retrieval: John Eyers is an information specialist with experience developing and peer reviewing search strategies for over hundred systematic reviews. He is the information specialist for the Campbell International Development group.

## SOURCES OF SUPPORT

The Children's Investment Fund Foundation provided the funding for this review.

## DECLARATIONS OF INTEREST

Paul Ferraro was involved in several of the studies included in the review and has written extensively on Payments for Environmental Services. However; he has no financial and professional interest in the outcomes of the review. The remaining study authors have no conflict of interests todeclare.

## PLANS FOR UPDATING THE REVIEW

The review will be updated when there are a sufficient number of new studies available, provided the authors are able to attract the necessary resource for doing so.

## Footnotes

1 A web‐based software programme for managing and analysing data in systematic reviews.

2 This mechansism is largely identified in community‐level PES programmes rather than individual‐level programmes.

3 The value of ecosystems services to humans was concretised in the Millennium Ecosystems Assessment report published in 2005 (MEA, 2005). They define ecosystems services as the benefits that humans get from ecosystems.

4 This number is quite high as it is inclusive of a broad range of study designs, including cross‐sectional studies with identification strategies considered to be at a very high‐risk of bias.

5 These criteria draw heavily on Snilstveit et al. (2015).

6 The critical appraisal tools used to assess the trustworthiness of the studies included in this review are presented in Appendix 3.

7 A crossover occurs where a treatment unit moves from treatment to comparison group (crossover) and contamination occurs where a comparison unit moves to the treatment group. Spillover effects refer to indirect effects of intervention in control caused by interactions (dependence) between treatment and control groups.

8 We are grateful to Phillip Mohebalian who provided additional data to calculate effect sizes for Mohebalian and Aguilar (2016, 2018).

9 Appendix 4 provides an overview of the search results per database.

10 World Bank Classifications can be found at: https://datahelpdesk.worldbank.org/knowledgebase/articles/906519‐world‐bank‐country‐and‐lending‐groups.

11 This information is based on what was available in the impact evaluations or associated qualitative and project documents.

12 For all meta‐analysis and results reported throughout Section 4, additional information on the robustness of the meta‐analysis can be found in Appendix 7, while additional information on all calculated effect sizes can be found in Appendix 8.

13 While we suspect there be a unit of analysis error for Arriagada et al. (2018), we were unable to recalculate due to missing number of clusters.

14 The identified pooled effect is too small and crossing the line of no effects in order to be regarded as convincing evidence of PES's positive effects.

15 We reversed the sign of the included studies that looked at deforestation for this meta‐analysis.

16 A reduction in deforestation, that is a negative effect size, is desirable for this outcome.

17 Inductive codes can be associated with more than one descriptive theme.

18 This mechansism is largely identified in community‐level PES programmes rather than individual‐level programmes.

19 This mechansism is largely identified in community‐level PES programmes rather than individual‐level programmes.

20 A possible monetisation of environmental behaviour refers to environmental practices such as conversation becoming dependent on the provision of a financial incentive.

## APPENDIX 1 SEARCH STRATEGIES

**CAB Abstracts (Ovid) <1990 to 2017 Week 33>Searched 25th August 2017**

1 (REDD+ or REDD or “Reduced Emissions from Deforestation and Degradation”).ti,ab. (1847)

2 ((pay* or reward* or incentiv* or compensat*) adj10 (agricultur* or livestock or farmland* or farm‐land* or “forest management” or “land management” or technology or conservation or “watershed management” or forest* or deforest* or eco or ecol* or ecos* or environment* or conservation or afforest* or reforest* or restor* or “natural regenerat*” or rainforest* or rain‐forest* or agroforest* or agro‐forest* or “natural resource*” or silvopastor* or “land use*” or “land cover” or “land‐cover” or “land‐use*” or peatland* or peat‐land* or mangrove* or grassland* or grass‐land* or wetland* or wet‐land*)).ti,ab. (15283)

3 (PES or Grain‐for‐green or “Grain for green” or “Sloping Land Conversion Program*” or “Priority Forestry Program*” or “Pago de Servicios Ambientales” or PSA or “Pago por Servicios Ambientales‐Hidrológico” or PSAH).ti,ab. (4576)

4 (sustainability or ecosystem services or carbon sequestration or environmental protection or ecosystem management or biodiversity).sh. and (pay* or reward* or incentiv* or compensat*).ti,ab. (8644)

5 or/1–4 (24461)

6 (Afghanistan or Albania or Algeria or Angola or Antigua or Barbuda or Argentina or Armenia or Armenian or Aruba or Azerbaijan or Bahrain or Bangladesh or Barbados or Benin or Byelarus or Byelorussian or Belarus or Belorussian or Belorussia or Belize or Bhutan or Bolivia or Bosnia or Herzegovina or Hercegovina or Botswana or Brasil or Brazil or Bulgaria or “Burkina Faso” or “Burkina Fasso” or “Upper Volta” or Burundi or Urundi or Cambodia or “Khmer Republic” or Kampuchea or Cameroon or Cameroons or Cameron or Camerons or “Cape Verde” or “Central African Republic” or Chad or Chile or China or Colombia or Comoros or “Comoro Islands” or Comores or Mayotte or Congo or Zaire or “Costa Rica*” or “Cote d'Ivoire” or “Ivory Coast” or Croatia or Cuba or Czechoslovakia or “Czech Republic” or Slovakia or “Slovak Republic” or Djibouti or “French Somaliland” or Dominica or “Dominican Republic” or “East Timor” or “East Timur” or “Timor Leste” or Ecuador or Egypt or “United Arab Republic” or “El Salvador” or Eritrea or Estonia or Ethiopia or Fiji or Gabon or “Gabonese Republic” or Gambia or Gaza or “Georgia Republic” or “Georgian Republic” or Ghana or “Gold Coast” or Greece or Grenada or Guatemala or Guinea or Guam or Guiana or Guyana or Haiti or Honduras or India or Maldives or Indonesia or Iran or Iraq or Jamaica or Jordan or Kazakhstan or Kazakh or Kenya or Kiribati or Korea or Kosovo or Kyrgyzstan or Kirghizia or “Kyrgyz Republic” or Kirghiz or Kirgizstan or “Lao PDR” or Laos or Latvia or Lebanon or Lesotho or Basutoland or Liberia or Libya or Lithuania or Macedonia or Madagascar or “Malagasy Republic” or Malaysia or Malaya or Malay or Sabah or Sarawak or Malawi or Nyasaland or Mali or Malta or “Marshall Islands” or Mauritania or Mauritius or “Agalega Islands” or Mexico or Micronesia or “Middle East” or Moldova or Moldovia or Moldovian or Mongolia or Montenegro or Morocco or Ifni or Mozambique or Myanmar or Myanma or Burma or Namibia or Nepal or “Netherlands Antilles” or “New Caledonia” or Nicaragua or Niger or Nigeria or “Northern Mariana Islands” or Oman or Muscat or Pakistan or Palau or Palestine or Panama or Paraguay or Peru or Philippines or Philipines or Phillipines or Phillippines or “Puerto Ric*” or Romania or Rumania or Roumania or Russia or Russian or Rwanda or Ruanda or “Saint Kitts” or “St Kitts” or “Nevis” or “Saint Lucia” or “St Lucia” or “Saint Vincent” or “St Vincent” or Grenadines or Samoa or “Samoan Islands” or “Navigator Island” or “Navigator Islands” or “Sao Tome” or “Saudi Arabia” or Senegal or Serbia or Montenegro or Seychelles or “Sierra Leone” or Slovenia or “Sri Lanka” or Ceylon or “Solomon Islands” or Somalia or “South Africa” or Sudan or Suriname or Surinam or Swaziland or Syria or Tajikistan or Tadzhikistan or Tadjikistan or Tadzhik or Tanzania or Thailand or Togo or Togolese Republic or Tonga or Trinidad or Tobago or Tunisia or Turkey or Turkmenistan or Turkmen or Uganda or Ukraine or Uruguay or USSR or “Soviet Union” or “Union of Soviet Socialist Republics” or Uzbekistan or Uzbek or Vanuatu or “New Hebrides” or Venezuela or Vietnam or “Viet Nam” or “West Bank” or Yemen or Yugoslavia or Zambia or Zimbabwe or Rhodesia).mp. not (“African American*” or “African‐American*” or “Mexican American*” or “American Indian*” or “Asian American*” or “native american*”).ti,ab,sh. [mp=abstract, title, original title, broad terms, heading words, identifiers, cabicodes] (1890298)

7 ((developing or “less* developed” or “under developed” or underdeveloped or “under developed” or “middle income” or “low* income”) adj3 (countr* or nation*)).ti,ab. (47918)

8 ((developing or “less* developed” or “under developed” or underdeveloped or “middle income” or “low* income”) adj3 (countr* or nation*)).ti,ab. (47918)

9 ((low adj3 middle adj3 countr*) or Africa or Asia or Caribbean or “West Indies” or “South America” or “Latin America” or “Central America”).ti,ab,sh. (167043)

10 (lmic or lmics or “third world” or “lami countr*” or “transitional countr*”).ti,ab. (2682)

11 or/6–10 (1960497)

12 (“random* control* trial*” or “random* trial*” or RCT or “propensity score matching” or PSM or “regression discontinuity design” or RDD or “difference in difference*” or matching or (random* adj3 allocat*) or “instrumental variable*” or IV or evaluation or assessment or “comparison group” or counterfactual or “counter factual” or counter‐factual or quasi‐experimental or quasiexperimental or ((quantitative or experiment*) adj3 (design or study or analysis)) or QED).ti,ab,sh. (702156)

13 5 and 11 and 12 (1649)

**Web of Science**—**Searched 29th August 2017**

# 132,222

#12 AND #11 AND #5

Indexes=SCI‐EXPANDED, SSCI, A&HCI, CPCI‐S, CPCI‐SSH, ESCI Timespan=All years

# 124,807,089

TS=(“random* control* trial*” or “random* trial*” or RCT or “propensity score matching” or PSM or “regression discontinuity design” or RDD or “difference in difference*” or matching or (random* adj3 allocat*) or “instrumental variable*” or IV or evaluation or assessment or “comparison group” or counterfactual or “counter factual” or counter‐factual or quasi‐experimental or quasiexperimental or ((quantitative or experiment*) NEAR/3 (design or study or analysis)) or QED)

# 112,923,401

#10 OR #9 OR #8 OR #7 OR #6

# 1012,272

TS=(lmic or lmics or “third world” or “lami countr*” or “transitional countr*”)

# 9487,643

TS=((low NEAR/3 middle NEAR/3 countr*) or Africa or Asia or Caribbean or “West Indies” or “South America” or “Latin America” or “Central America”)

# 8159,540

TS=((developing or “less* developed” or “under developed” or underdeveloped or “middle income” or “low* income”) NEAR/3 (countr* or nation*))

# 7159,540

TS=((developing or “less* developed” or “under developed” or underdeveloped or “under developed” or “middle income” or “low* income”) NEAR/3 (countr* or nation*))

# 62,627,340

TS=((Afghanistan or Albania or Algeria or Angola or Antigua or Barbuda or Argentina or Armenia or Armenian or Aruba or Azerbaijan or Bahrain or Bangladesh or Barbados or Benin or Byelarus or Byelorussian or Belarus or Belorussian or Belorussia or Belize or Bhutan or Bolivia or Bosnia or Herzegovina or Hercegovina or Botswana or Brasil or Brazil or Bulgaria or “Burkina Faso” or “Burkina Fasso” or “Upper Volta” or Burundi or Urundi or Cambodia or “Khmer Republic” or Kampuchea or Cameroon or Cameroons or Cameron or Camerons or “Cape Verde” or “Central African Republic” or Chad or Chile or China or Colombia or Comoros or “Comoro Islands” or Comores or Mayotte or Congo or Zaire or “Costa Rica*” or “Cote d'Ivoire” or “Ivory Coast” or Croatia or Cuba or Czechoslovakia or “Czech Republic” or Slovakia or “Slovak Republic” or Djibouti or “French Somaliland” or Dominica or “Dominican Republic” or “East Timor” or “East Timur” or “Timor Leste” or Ecuador or Egypt or “United Arab Republic” or “El Salvador” or Eritrea or Estonia or Ethiopia or Fiji or Gabon or “Gabonese Republic” or Gambia or Gaza or “Georgia Republic” or “Georgian Republic” or Ghana or “Gold Coast” or Greece or Grenada or Guatemala or Guinea or Guam or Guiana or Guyana or Haiti or Honduras or India or Maldives or Indonesia or Iran or Iraq or Jamaica or Jordan or Kazakhstan or Kazakh or Kenya or Kiribati or Korea or Kosovo or Kyrgyzstan or Kirghizia or “Kyrgyz Republic” or Kirghiz or Kirgizstan or “Lao PDR” or Laos or Latvia or Lebanon or Lesotho or Basutoland or Liberia or Libya or Lithuania or Macedonia or Madagascar or “Malagasy Republic” or Malaysia or Malaya or Malay or Sabah or Sarawak or Malawi or Nyasaland or Mali or Malta or “Marshall Islands” or Mauritania or Mauritius or “Agalega Islands” or Mexico or Micronesia or “Middle East” or Moldova or Moldovia or Moldovian or Mongolia or Montenegro or Morocco or Ifni or Mozambique or Myanmar or Myanma or Burma or Namibia or Nepal or “Netherlands Antilles” or “New Caledonia” or Nicaragua or Niger or Nigeria or “Northern Mariana Islands” or Oman or Muscat or Pakistan or Palau or Palestine or Panama or Paraguay or Peru or Philippines or Philipines or Phillipines or Phillippines or “Puerto Ric*” or Romania or Rumania or Roumania or Russia or Russian or Rwanda or Ruanda or “Saint Kitts” or “St Kitts” or “Nevis” or “Saint Lucia” or “St Lucia” or “Saint Vincent” or “St Vincent” or Grenadines or Samoa or “Samoan Islands” or “Navigator Island” or “Navigator Islands” or “Sao Tome” or “Saudi Arabia” or Senegal or Serbia or Montenegro or Seychelles or “Sierra Leone” or Slovenia or “Sri Lanka” or Ceylon or “Solomon Islands” or Somalia or “South Africa” or Sudan or Suriname or Surinam or Swaziland or Syria or Tajikistan or Tadzhikistan or Tadjikistan or Tadzhik or Tanzania or Thailand or Togo or Togolese Republic or Tonga or Trinidad or Tobago or Tunisia or Turkey or Turkmenistan or Turkmen or Uganda or Ukraine or Uruguay or USSR or “Soviet Union” or “Union of Soviet Socialist Republics” or Uzbekistan or Uzbek or Vanuatu or “New Hebrides” or Venezuela or Vietnam or “Viet Nam” or “West Bank” or Yemen or Yugoslavia or Zambia or Zimbabwe or Rhodesia) NOT (“AfricanAmerican*” or “African‐American*” or “Mexican American*” or “American Indian*” or “Asian American*” or “native american*”))

# 589,570

#4 OR #3 OR #2 OR #1

# 411,393

TS=((sustainability or “ecosystem services” or “carbon sequestration” or “environmental protection” or “ecosystem management” or biodiversity) AND (pay* or reward* or incentiv* or compensat*))

# 352,400

TS=(PES or Grain‐for‐green or “Grain for green” or “Sloping Land Conversion Program*” or “Priority Forestry Program*” or “Pago de Servicios Ambientales” or PSA or “Pago por Servicios Ambientales‐Hidrológico” or PSAH)

# 229,051

TS=((pay* or reward* or incentiv* or compensat*) NEAR/10 (agricultur* or livestock or farmland* or farm‐land* or “forest management” or “land management” or technology or conservation or “watershed management” or forest* or deforest* or eco or ecol* or ecos* or environment* or conservation or afforest* or reforest* or restor* or “natural regenerat*” or rainforest* or rain‐forest* or agroforest* or agro‐forest* or “natural resource*” or silvopastor* or “land use*” or “land cover” or “land‐cover” or “land‐use*” or peatland* or peat‐land* or mangrove* or grassland* or grass‐land* or wetland* or wet‐land*))

# 12,350

TS=(REDD+ or REDD or “Reduced Emissions from Deforestation and Degradation”)

**Ebsco Discovery**‐**Agris, Econlit & RePeC**‐**Searched 30th August 2017**

**Greenfile (Ebsco)**‐**Searched 30th August 2017**

S12 S5 AND S10 AND S119,445

(**Agris**‐**815; Econlit**‐**230; RePeC**‐**412; Greenfile**‐**295**)

S11 TI ((“random* control* trial*” or “random* trial*” or RCT or “propensity score matching” or PSM or “regression discontinuity design” or RDD or “difference in difference*” or matching or (random* N3 allocat*) or “instrumental variable*” or IV or evaluation or assessment or “comparison group” or counterfactual or “counter factual” or counter‐factual or quasi‐experimental or quasiexperimental or ((quantitative or experiment*) N3 (design or study or analysis)) or QED)) OR AB ((“random* control* trial*” or “random* trial*” or RCT or “propensity score matching” or PSM or “regression discontinuity design” or RDD or “difference in difference*” or matching or (random* N3 allocat*) or “instrumental variable*” or IV or evaluation or assessment or “comparison group” or counterfactual or “counter factual” or counter‐factual or quasi‐experimental or quasiexperimental or ((quantitative or experiment*) N3 (design or study or analysis)) or QED)) OR SU ((“random* control* trial*” or “random* trial*” or RCT or “propensity score matching” or PSM or “regression discontinuity design” or RDD or “difference in difference*” or matching or (random* N3 allocat*) or “instrumental variable*” or IV or evaluation or assessment or “comparison group” or counterfactual or “counter factual” or counter‐factual or quasi‐experimental or quasiexperimental or ((quantitative or experiment*) N3 (design or study or analysis)) or QED))

18,629,561

S10 S6 OR S7 OR S8 OR S9 25,507,282

S9 TI ((lmic or lmics or “third world” or “lami countr*” or “transitional countr*”)) OR AB ((lmic or lmics or “third world” or “lami countr*” or “transitional countr*”)) OR SU ((lmic or lmics or “third world” or “lami countr*” or “transitional countr*”)) 117,221

S8 TI (((low N3 middle N3 countr*) or Africa or Asia or Caribbean or “West Indies” or “South America” or “Latin America” or “Central America”)) OR AB (((low N3 middle N3 countr*) or Africa or Asia or Caribbean or “West Indies” or “South America” or “Latin America” or “Central America”)) OR SU (((low N3 middle N3 countr*) or Africa or Asia or Caribbean or “West Indies” or “South America” or “Latin America” or “Central America”))6,962,117

S7 TI (((developing or “less* developed” or “under developed” or underdeveloped or “middle income” or “low* income”) N3 (countr* or nation*))) OR AB (((developing or “less* developed” or “under developed” or underdeveloped or “middle income” or “low* income”) N3 (countr* or nation*))) OR SU (((developing or “less* developed” or “under developed” or underdeveloped or “middle income” or “low* income”) N3 (countr* or nation*)))1,738,088

S6 TI ((Afghanistan or Albania or Algeria or Angola or Antigua or Barbuda or Argentina or Armenia or Armenian or Aruba or Azerbaijan or Bahrain or Bangladesh or Barbados or Benin or Byelarus or Byelorussian or Belarus or Belorussian or Belorussia or Belize or Bhutan or Bolivia or Bosnia or Herzegovina or Hercegovina or Botswana or Brasil or Brazil or Bulgaria or “Burkina Faso” or “Burkina Fasso” or “Upper Volta” or Burundi or Urundi or Cambodia or “Khmer Republic” or Kampuchea or Cameroon or Cameroons or Cameron or Camerons or “Cape Verde” or “Central African Republic” or Chad or Chile or China or Colombia or Comoros or “Comoro Islands” or Comores or Mayotte or Congo or Zaire or “Costa Rica*” or “Cote d'Ivoire” or “Ivory Coast” or Croatia or Cuba or Czechoslovakia or “Czech Republic” or Slovakia or “Slovak Republic” or Djibouti or “French Somaliland” or Dominica or “Dominican Republic” or “East Timor” or “East Timur” or “Timor Leste” or Ecuador or Egypt or “United Arab Republic” or “El Salvador” or Eritrea or Estonia or Ethiopia or Fiji or Gabon or “Gabonese Republic” or Gambia or Gaza or “Georgia Republic” or “Georgian Republic” or Ghana or “Gold Coast” or Greece or Grenada or Guatemala or Guinea or Guam or Guiana or Guyana or Haiti or Honduras or India or Maldives or Indonesia or Iran or Iraq or Jamaica or Jordan or Kazakhstan or Kazakh or Kenya or Kiribati or Korea or Kosovo or Kyrgyzstan or Kirghizia or “Kyrgyz Republic” or Kirghiz or Kirgizstan or “Lao PDR” or Laos or Latvia or Lebanon or Lesotho or Basutoland or Liberia or Libya or Lithuania or Macedonia or Madagascar or “Malagasy Republic” or Malaysia or Malaya or Malay or Sabah or Sarawak or Malawi or Nyasaland or Mali or Malta or “Marshall Islands” or Mauritania or Mauritius or “Agalega Islands” or Mexico or Micronesia or “Middle East” or Moldova or Moldovia or Moldovian or Mongolia or Montenegro or Morocco or Ifni or Mozambique or Myanmar or Myanma or Burma or Namibia or Nepal or “Netherlands Antilles” or “New Caledonia” or Nicaragua or Niger or Nigeria or “Northern Mariana Islands” or Oman or Muscat or Pakistan or Palau or Palestine or Panama or Paraguay or Peru or Philippines or Philipines or Phillipines or Phillippines or “Puerto Ric*” or Romania or Rumania or Roumania or Russia or Russian or Rwanda or Ruanda or “Saint Kitts” or “St Kitts” or “Nevis” or “Saint Lucia” or “St Lucia” or “Saint Vincent” or “St Vincent” or Grenadines or Samoa or “Samoan Islands” or “Navigator Island” or “Navigator Islands” or “Sao Tome” or “Saudi Arabia” or Senegal or Serbia or Montenegro or Seychelles or “Sierra Leone” or Slovenia or “Sri Lanka” or Ceylon or “Solomon Islands” or Somalia or “South Africa” or Sudan or Suriname or Surinam or Swaziland or Syria or Tajikistan or Tadzhikistan or Tadjikistan or Tadzhik or Tanzania or Thailand or Togo or “Togolese Republic” or Tonga or Trinidad or Tobago or Tunisia or Turkey or Turkmenistan or Turkmen or Uganda or Ukraine or Uruguay or USSR or “Soviet Union” or “Union of Soviet Socialist Republics” or Uzbekistan or Uzbek or Vanuatu or “New Hebrides” or Venezuela or Vietnam or “Viet Nam” or “West Bank” or Yemen or Yugoslavia or Zambia or Zimbabwe or Rhodesia) NOT (“African American*” or “African‐American*” or “Mexican American*” or “American Indian*” or “Asian American*” or “native american*”)) OR AB ((Afghanistan or Albania or Algeria or Angola or Antigua or Barbuda or Argentina or Armenia or Armenian or Aruba or Azerbaijan or Bahrain or Bangladesh or Barbados or Benin or Byelarus or Byelorussian or Belarus or Belorussian or Belorussia or Belize or Bhutan or Bolivia or Bosnia or Herzegovina or Hercegovina or Botswana or Brasil or Brazil or Bulgaria or “Burkina Faso” or “Burkina Fasso” or “Upper Volta” or Burundi or Urundi or Cambodia or “Khmer Republic” or Kampuchea or Cameroon or Cameroons or Cameron or Camerons or “Cape Verde” or “Central African Republic” or Chad or Chile or China or Colombia or Comoros or “Comoro Islands” or Comores or Mayotte or Congo or Zaire or “Costa Rica*” or “Cote d'Ivoire” or “Ivory Coast” or Croatia or Cuba or Czechoslovakia or “Czech Republic” or Slovakia or “Slovak Republic” or Djibouti or “French Somaliland” or Dominica or “Dominican Republic” or “East Timor” or “East Timur” or “Timor Leste” or Ecuador or Egypt or “United Arab Republic” or “El Salvador” or Eritrea or Estonia or Ethiopia or Fiji or Gabon or “Gabonese Republic” or Gambia or Gaza or “Georgia Republic” or “Georgian Republic” or Ghana or “Gold Coast” or Greece or Grenada or Guatemala or Guinea or Guam or Guiana or Guyana or Haiti or Honduras or India or Maldives or Indonesia or Iran or Iraq or Jamaica or Jordan or Kazakhstan or Kazakh or Kenya or Kiribati or Korea or Kosovo or Kyrgyzstan or Kirghizia or “Kyrgyz Republic” or Kirghiz or Kirgizstan or “Lao PDR” or Laos or Latvia or Lebanon or Lesotho or Basutoland or Liberia or Libya or Lithuania or Macedonia or Madagascar or “Malagasy Republic” or Malaysia or Malaya or Malay or Sabah or Sarawak or Malawi or Nyasaland or Mali or Malta or “Marshall Islands” or Mauritania or Mauritius or “Agalega Islands” or Mexico or Micronesia or “Middle East” or Moldova or Moldovia or Moldovian or Mongolia or Montenegro or Morocco or Ifni or Mozambique or Myanmar or Myanma or Burma or Namibia or Nepal or “Netherlands Antilles” or “New Caledonia” or Nicaragua or Niger or Nigeria or “Northern Mariana Islands” or Oman or Muscat or Pakistan or Palau or Palestine or Panama or Paraguay or Peru or Philippines or Philipines or Phillipines or Phillippines or “Puerto Ric*” or Romania or Rumania or Roumania or Russia or Russian or Rwanda or Ruanda or “Saint Kitts” or “St Kitts” or “Nevis” or “Saint Lucia” or “St Lucia” or “Saint Vincent” or “St Vincent” or Grenadines or Samoa or “Samoan Islands” or “Navigator Island” or “Navigator Islands” or “Sao Tome” or “Saudi Arabia” or Senegal or Serbia or Montenegro or Seychelles or “Sierra Leone” or Slovenia or “Sri Lanka” or Ceylon or “Solomon Islands” or Somalia or “South Africa” or Sudan or Suriname or Surinam or Swaziland or Syria or Tajikistan or Tadzhikistan or Tadjikistan or Tadzhik or Tanzania or Thailand or Togo or “Togolese Republic” or Tonga or Trinidad or Tobago or Tunisia or Turkey or Turkmenistan or Turkmen or Uganda or Ukraine or Uruguay or USSR or “Soviet Union” or “Union of Soviet Socialist Republics” or Uzbekistan or Uzbek or Vanuatu or “New Hebrides” or Venezuela or Vietnam or “Viet Nam” or “West Bank” or Yemen or Yugoslavia or Zambia or Zimbabwe or Rhodesia) NOT (“African American*” or “African‐American*” or “Mexican American*” or “American Indian*” or “Asian American*” or “native american*”)) OR SU ((Afghanistan or Albania or Algeria or Angola or Antigua or Barbuda or Argentina or Armenia or Armenian or Aruba or Azerbaijan or Bahrain or Bangladesh or Barbados or Benin or Byelarus or Byelorussian or Belarus or Belorussian or Belorussia or Belize or Bhutan or Bolivia or Bosnia or Herzegovina or Hercegovina or Botswana or Brasil or Brazil or Bulgaria or “Burkina Faso” or “Burkina Fasso” or “Upper Volta” or Burundi or Urundi or Cambodia or “Khmer Republic” or Kampuchea or Cameroon or Cameroons or Cameron or Camerons or “Cape Verde” or “Central African Republic” or Chad or Chile or China or Colombia or Comoros or “Comoro Islands” or Comores or Mayotte or Congo or Zaire or “Costa Rica*” or “Cote d'Ivoire” or “Ivory Coast” or Croatia or Cuba or Czechoslovakia or “Czech Republic” or Slovakia or “Slovak Republic” or Djibouti or “French Somaliland” or Dominica or “Dominican Republic” or “East Timor” or “East Timur” or “Timor Leste” or Ecuador or Egypt or “United Arab Republic” or “El Salvador” or Eritrea or Estonia or Ethiopia or Fiji or Gabon or “Gabonese Republic” or Gambia or Gaza or “Georgia Republic” or “Georgian Republic” or Ghana or “Gold Coast” or Greece or Grenada or Guatemala or Guinea or Guam or Guiana or Guyana or Haiti or Honduras or India or Maldives or Indonesia or Iran or Iraq or Jamaica or Jordan or Kazakhstan or Kazakh or Kenya or Kiribati or Korea or Kosovo or Kyrgyzstan or Kirghizia or “Kyrgyz Republic” or Kirghiz or Kirgizstan or “Lao PDR” or Laos or Latvia or Lebanon or Lesotho or Basutoland or Liberia or Libya or Lithuania or Macedonia or Madagascar or “Malagasy Republic” or Malaysia or Malaya or Malay or Sabah or Sarawak or Malawi or Nyasaland or Mali or Malta or “Marshall Islands” or Mauritania or Mauritius or “Agalega Islands” or Mexico or Micronesia or “Middle East” or Moldova or Moldovia or Moldovian or Mongolia or Montenegro or Morocco or Ifni or Mozambique or Myanmar or Myanma or Burma or Namibia or Nepal or “Netherlands Antilles” or “New Caledonia” or Nicaragua or Niger or Nigeria or “Northern Mariana Islands” or Oman or Muscat or Pakistan or Palau or Palestine or Panama or Paraguay or Peru or Philippines or Philipines or Phillipines or Phillippines or “Puerto Ric*” or Romania or Rumania or Roumania or Russia or Russian or Rwanda or Ruanda or “Saint Kitts” or “St Kitts” or “Nevis” or “Saint Lucia” or “St Lucia” or “Saint Vincent” or “St Vincent” or Grenadines or Samoa or “Samoan Islands” or “Navigator Island” or “Navigator Islands” or “Sao Tome” or “Saudi Arabia” or Senegal or Serbia or Montenegro or Seychelles or “Sierra Leone” or Slovenia or “Sri Lanka” or Ceylon or “Solomon Islands” or Somalia or “South Africa” or Sudan or Suriname or Surinam or Swaziland or Syria or Tajikistan or Tadzhikistan or Tadjikistan or Tadzhik or Tanzania or Thailand or Togo or “Togolese Republic” or Tonga or Trinidad or Tobago or Tunisia or Turkey or Turkmenistan or Turkmen or Uganda or Ukraine or Uruguay or USSR or “Soviet Union” or “Union of Soviet Socialist Republics” or Uzbekistan or Uzbek or Vanuatu or “New Hebrides” or Venezuela or Vietnam or “Viet Nam” or “West Bank” or Yemen or Yugoslavia or Zambia or Zimbabwe or Rhodesia) NOT (“African American*” or “African‐American*” or “Mexican American*” or “American Indian*” or “Asian American*” or “native american*”)) 23,130,617

S5 S1 OR S2 OR S3 OR S4 435,257

S4 TI ((sustainability or “ecosystem services” or “carbon sequestration” or “environmental protection” or “ecosystem management” or biodiversity) N5 (pay* or reward* or incentiv* or compensat*)) OR AB ((sustainability or “ecosystem services” or “carbon sequestration” or “environmental protection” or “ecosystem management” or biodiversity) N5 (pay* or reward* or incentiv* or compensat*)) OR SU ((sustainability or “ecosystem services” or “carbon sequestration” or “environmental protection” or “ecosystem management” or biodiversity) N5 (pay* or reward* or incentiv* or compensat*)) 12,309

S3 TI (PES or Grain‐for‐green or “Grain for green” or “Sloping Land Conversion Program*” or “Priority Forestry Program*” or “Pago de Servicios Ambientales” or PSA or “Pago por Servicios Ambientales‐Hidrológico” or PSAH) OR AB (PES or Grain‐for‐green or “Grain for green” or “Sloping Land Conversion Program*” or “Priority Forestry Program*” or “Pago de Servicios Ambientales” or PSA or “Pago por Servicios Ambientales‐Hidrológico” or PSAH) OR SU (PES or Grain‐for‐green or “Grain for green” or “Sloping Land Conversion Program*” or “Priority Forestry Program*” or “Pago de Servicios Ambientales” or PSA or “Pago por Servicios Ambientales‐Hidrológico” or PSAH) 157,030

S2 TI (((pay* or reward* or incentiv* or compensat*) N10 (agricultur* or livestock or farmland* or farm‐land* or “forest management” or “land management” or technology or conservation or “watershed management” or forest* or deforest* or eco or ecol* or ecos* or environment* or conservation or afforest* or reforest* or restor* or “natural regenerat*” or rainforest* or rain‐forest* or agroforest* or agro‐forest* or “natural resource*” or silvopastor* or “land use*” or “land cover” or “land‐cover” or “land‐use*” or peatland* or peat‐land* or mangrove* or grassland* or grass‐land* or wetland* or wet‐land*))) OR AB (((pay* or reward* or incentiv* or compensat*) N10 (agricultur* or livestock or farmland* or farm‐land* or “forest management” or “land management” or technology or conservation or “watershed management” or forest* or deforest* or eco or ecol* or ecos* or environment* or conservation or afforest* or reforest* or restor* or “natural regenerat*” or rainforest* or rain‐forest* or agroforest* or agro‐forest* or “natural resource*” or silvopastor* or “land use*” or “land cover” or “land‐cover” or “land‐use*” or peatland* or peat‐land* or mangrove* or grassland* or grass‐land* or wetland* or wet‐land*))) OR SU (((pay* or reward* or incentiv* or compensat*) N10 (agricultur* or livestock or farmland* or farm‐land* or “forest management” or “land management” or technology or conservation or “watershed management” or forest* or deforest* or eco or ecol* or ecos* or environment* or conservation or afforest* or reforest* or restor* or “natural regenerat*” or rainforest* or rain‐forest* or agroforest* or agro‐forest* or “natural resource*” or silvopastor* or “land use*” or “land cover” or “land‐cover” or “land‐use*” or peatland* or peat‐land* or mangrove* or grassland* or grass‐land* or wetland* or wet‐land*))) 243,445

S1 TI (REDD+ or REDD or “Reduced Emissions from Deforestation and Degradation”) OR AB (REDD+ or REDD or “Reduced Emissions from Deforestation and Degradation”) OR SU (REDD+ or REDD or “Reduced Emissions from Deforestation and Degradation”) 16,106

**AgEcon**—**Searched 30th August 2017**

((pay* OR reward* OR incentiv* OR compensat*) AND (agricultur* OR livestock OR farmland* OR farm‐land* OR “forest management” OR “land management” OR technology OR conservation OR “watershed management” OR forest* OR deforest* OR eco OR ecol* OR ecos* OR environment* OR conservation OR afforest* OR reforest* OR restor* OR “natural regenerat*” OR rainforest* OR rain‐forest* OR agroforest* OR agro‐forest* OR “natural resource*” OR silvopastor* OR “land use*” OR “land cover” OR “land‐cover” OR “land‐use*” OR peatland* OR peat‐land* OR mangrove* OR grassland* OR grass‐land* OR wetland* OR wet‐land*))

## APPENDIX 2 DATA EXTRACTION

**Intervention and study description, process, implementation, qualitative and cost data**

**Table:**

	Description	Question	Coding
**Report identification**	Unique study identification #		For example, PES001
	First author—impact evaluation	Surname	Surname
	Other papers used for coding	First author surname and type of paper of any qualitative, descriptive quantitative, process evaluations or project documents used for coding	
	General comments	**(1) General comments**: any general comments on study not coded elsewhere **(2) Issues of comparability**: please report any potential issues of comparability between different documents (e.g., different documents assess a programme/intervention at different scales [geographic/time scale]). If the issue of comparability related only to a certain secion of a document (e.g., cost data), please put in brackets in relevant cell	Open answer
	Publication date	Year (letter)	XXXX (a)
	Publication type	What is the impact evaluation publication type?	1 = Peer‐reviewed journal 2 = Book chapter/book 3 = Conference paper 4 = Organisation report 5 = Working paper 6 = Implementation document 7 = Other grey 8 = PhD thesis/dissertation
	Funding agency	Who is funding the evaluation/study?	1 = Public institution (e.g., govt, NGO, university, research institute) 2 = Private institution (e.g., private company) 3 = Multilateral Organisation (World Bank, UN) 4 = Foundations 8 = Not clear 9 = Not applicable (Non‐funded)
	Name of funding agency	Please add name of the agency funding the evaluation	Open answer
	Independence of evaluation	What level of independence is there between the impleenting agency and study team?	1 = Funding and author team independent of implementers/funders of programme 2 = Funding independent of implementers/funders of programme, but includes authors from funder/implementer 3 = Evaluation funded and undetaken by funders/implementers 8 = Unclear
	Independent data collection	Has the data been collected by an independent party?	1 = Yes, 2 = No, 8 = Not clear
	Conflict of interest	Is there a potential conflict of interest associated with study which could influence results collected/reported? (e.g., is there a declaration of conflict of interest? Is any of the authors related in any way to the funding or implenting institution?)	1 = Yes, 2 = No, 8 = Not clear
	Comments on conflict of interest	Please add reason for your answer to whether there is a conflict of interest	Open answer
	Language of publication	Language of publication of the impact evaluation, for example, Spanish, English and for forth	Open answer
	Other methods	If the impact evaluation addresses other questions than effectiveness note questions and methods used here	Open answer (this will include, e.g., mixed‐methods to assess implementation, adherence, participant views etc.)
**Intervention descriptives**	Programme or project name	State the programme or project name. If no name, then list the location (e.g., Town, village etc.)	Open answer
	Intervention type	Indicate type of intervention	1 = PES alone 2 = PES + other intervention
	Type of ecosystem targeted	Indicate the type of ecosystem targeted	1 = Forests 2 = Farmland 3 = Grassland 4 = Mangroves 5 = Wetlands
	Intervention description	Provide descriptive details about the intervention. *Include detail on any other intervention provided alongside the PES, including alternative livelihoods strategies, awareness raising activities, increased forest monitoring etc*.	Open answer
	Objectives of intervention	Type of objective(s) of intervention	1 = Conservation only 2 = Restoration 3 = Environmentally beneficial/preferable to BAU land‐use 4 = Socioeconomic (livelihoods, poverty reduction etc.) 5 = Other (add description in comments)
	Objectives of intervention	State any objectives stated in study or project document, including whether the study targets both environmental and poverty objectives	
	Size of payment	Indicate the size of the regular payment	Open answer, $
	Frequency of payment	Indicate how frequently the payment is made (annual, monthly, etc.)	Open answer
	Method of payment	Indicate how payment made to participants	Open answer
	Conditionality	Indicate the stated conditions of the PES programme	Open answer
	Intervention scale	What is the scale of the intervention?	1 = Local 2 = Regional 2 = National
	Intervention implementing agency	Who is implementing the intervention? State the name (and department) of the implementing agency	Open answer
	Intervention funding agency	Type of funder	1 = Government 2 = User financed (companies using env service) 3 = NGO 4 = Multilateral/bilater organisation 5 = Carbon offset mechanism 6 = Other
	Intervention funding agency	Name of intervention funding agency	Open answer
	Intervention target group	What were the characteristics of beneficiaries used to target the intervention?	Open answer
	Targeting methods	How were beneficiaries targeted for the programme (e.g., how was the targeting implemented)?	Open answer
	Intervention start	Start date (if not stated, state study date) of intervention	XX/XXXX
	Intervention end	State end date (if ongoing state ongoing)	XX/XXXX
	Follow up	How long after the last payment was outcome data collected?	indicate number of months (numerical only). If not clear state so
	Program theory	Do the authors make explicit reference to program theory, theory of change or similar?	1 = Yes, 2 = No, 8 = Not clear
	Program theory	Report any description/statement of program theory as stated by author(s).	Open answer
**Context**	Country	List countries the study was conducted in	Country 1, Country 2, and so forth.
	Detailed location	If provided, give detailed information on where the study took place within a country, for example regions/districts covered	Open answer
	World Bank Region	Select region(s) the study was conducted in according to World Bank. For more info on region classification see http://data.worldbank.org/country	1 = East Asia & Pacific 2 = Europe & Central Asia 3 = Latin America & Caribbean 4 = Middle East & North Africa 5 = South Asia 6 = Sub‐Saharan Africa
	WB Income category	Select the World Bank income classification of the country at the time of the study	1 = Low income country 2 = Lower‐middle income country 3 = Upper‐middle income country
	REDD+ status	Is the country where the evaluation took place a REDD+ country?	1 = Yes, 2 = No, 3 = Unclear
	Environmental performance index	How does the country rank on the Environmental Perfomance Index: http://epi.yale.edu/?	Open answer—to be filled in after coding complete
	Baseline deforestation rates	Report any data/description on deforestation rates in programme/comparison area	Open answer
	Baseline socioeconomic status of participants	Report any data/description on baseline socio‐economic status of participants	Open answer
	Property right regime	Report any description in the primary evaluation or qualitative documents of the existing property rights regime	Open answer
**Process and implementation**	Information about program take‐up/adherence (among beneficiaries)	Is there any information about program take‐up/adherence (among beneficiaries)? Commentary by authors should be used when information on program adherence and so forth. is not backed up by some sort of research/when the authors do not report that/how they collected data to assess these areas	1 = Yes, commentary from author; 2 = No; 4 = Yes, formally assessed
	Methods of assessing take‐up/adherence	Which methods are used to assess program take‐up/adherence?	1 = Observation by intervention staff 2 = Reporting by participants 3 = Other 4 = Commentary from author 9 = Not measured
	Results of the assessment of take‐up/adherence	What is the result/information provided of the assessment of program take‐up/adherence?	Open answer
	Information about implementation fidelity/service delivery quality	Is there any information on implementation fidelity/service delivery quality? Commentary by authors should be used when information on program adherence and so forth. is not backed up by some sort of research/when the authors do not report that/how they collected data to assess these areas	1 = Yes, commentary from author; 2 = No; 4 = Yes, formally assessed
	Methods of assessing intervention fidelity	Which methods are used to assess implementation fidelity/service delivery quality	1 = Observation by intervention staff 2 = Reporting by participants 3 = Other 4 = Commentary from author 9 = Not measured
	Results of the assessment of intervention fidelity	What is the result/information provided of the assessment of implementation fidelity/service delivery quality	Open answer
	Other description of process factors	Any other description of process factors not covered above	Open answer
	Barriers and facilitators	Do the study identify any barriers and facilitators not included above?	Open answer
**Cost**	Cost	Are any unit cost data/cost‐effectiveness estimates provided?	1 = Yes. 2 = No
	Cost details	If yes, report any details of unit cost and/or total cost. Please also report year and currency	Open answer
**External Validity**	Length of study	Length of study in months (where study length not reported, code as length of intervention, noting that in brackets)	# months, if not reported N/A
	Efficacy or effectiveness trial	Was the intervention implemented under "real world" conditions? By real world we mean a programme implemented independently of the evaluation, either by government, NGO or international agency. For example, the programme is not designed and implemented for the purpose of research	1 = Yes, 2 = No, 9 = N/A
	Personell implementing the programme	Who was in charge of implementing the program?	1 = PI/researchers (study authors); 2 = implementing agency staff, 3 = external agency (eg: survey firm); 4 = others; 8 = not clear
	Sampling frame for the study	State the sampling frame (list of all those within a population who can be sampled, i.e., households, communities) for selection of study participants (i.e., census, etc.).	Open answer
	Author discussion of external validity	Do the authors discuss or explicitly address generalisability/applicability?	Open answer
	Theory	Is there any reference to theory of change underlying intervention?	1 = Yes, 2 = No, 9 = N/A
	Theory based evaluation	Is the study using theory to inform the evaluation design and analysis?	Open answer—describe if and how the authors use theory in the evaluation. Do they for example use it to inform data collection? Do they do any causal chain analysis?
**Equity**	Consideration of equity	Does the study consider equity?	1 = Yes, 2 = No
	Equity methods	How does the study consider equity?	1 = intervention target a disadvantaged group 2 = study measures inequality 3 = subgroup analysis by dimension of inequity
	Equity dimension	What dimension(s) of equity does the study consider?	1 = gender 2 = socioeconomic status 3 = place of recidence 4 = land ownership 5 = landsize

**Effect size data**

**Table:**

	Description	Question	Coding
ID	Unique study identification #		For example, PES001
	First author—impact evaluation	Surname	Open answer
Outcome for effect size (answer for all studies)	Primary outcome	Which primary outcomes is being coded?	1 = Forest cover/deforestation 2 = Forest condition 3 = Carbon stocks 4 = Greenhouse gas emissions 5 = Income/consumption/expenditure 6 = Food security 7 = Other socioeconomic outcome 8 = Intermediate outcomes
	Subgroup analysis	Is this effect size data for a subgroup?	1 = No 2 = Yes
	Subgroup analysis decription	If yes to question 2, which type of subgroup?	Open answer—this can include separate samples for gender, income, place of residence, land size, head of household (e.g., female or male headed)
	Definition of outcome	Please provide the authors definition of the outcome (including description of the subgroup if relevant)	Open answer
	Effect size location	Which page(s) contain the effect size data?	Open answer
	Data to be extracted	Which type of data to be extracted?	1 = Continuous—means and SDs 2 = Continuous—mean difference and SD 2 = Dichotomous outcome—proportions 3 = Regression data
Effect size data (answer for all studies)	Sample size metric	Sample size unit of analysis	1 = Individual 2 = Household 3 = Group (e.g., community organisation) 4 = Plot 5 = Village 6 = Not clear
	Treatment effect estimated	What treatment effect is estimated?	1 = ITT 2 = ATET 3 = ATE 4 = LATE
	Sample size (treatment)	Initial sample size treatment group	#
	Sample size (control)	Initial sample size control group	#
	Sample size (total)	Initial sample size total	#
	Observations (treatment)	Number of treatment observations after attrition (individuals)	#
	Observations (control)	Number of control observations after attrition (individuals)	#
	Observations (total)	Total number of control observations after attrition (individuals)	#
Outcome data—if continuous (means and SDs)	Baseline outcome treatement	State result of baseline outcome for treatment group	#
	SD baseline outcome treatement	State SD of baseline outcome measure for treatment group	#
	Sample size baseline treatment	State sample size at baseline	#
	Baseline outcome control	State result of baseline outcome for control group	#
	SD baseline outcome control	State SD of baseline outcome measure for contol group	#
	Sample size baseline control	State sample size at baseline	#
	Outcome in treatment post intervention	State result of post intervention outcome for treatment group	#
	SD outcome in treatment post intervention	State SD of post intervention outcome measure for treatment group	#
	Number with outcome in treatment post intervention	State sample size post intervention	#
	Outcome in control post intervention	State result of post intervention outcome for control group	#
	SD outcome in control post intervention	State SD of post intervention outcome measure for control group	#
	Number with outcome in contol post intervention	State sample size post intervention	#
	Outcome in treatment first follow up	State result of first follow up outcome measure for treatment group	#
	SD outcome in treatment first follow up	State SD first follow up outcome measure for treatment group	#
	Number with outcome in treatment first follow up	State sample size first follow up	#
	Outcome in control first follow up	State result of first follow up outcome measure for treatment group	#
	SD outcome in control first follow up	State SD first follow up outcome measure for treatment group	#
	Number with outcome in control first follow up	State sample size first follow up	#
Outcome data—If continuous (mean difference and SD at follow up)	Mean difference at follow up	State mean difference	#
	SD at follow up	State SD at follow up	#
Outcomes data—if dichotomous (Proportions r)	Baseline number with outcome in treatement	State result of baseline outcome for treatment group	#
	Sample size baseline treatment	State sample size at baseline	#
	Proportion with outcome at baseline in treatment	State proportion with outcome at baseline in treatment	#
	Baseline number with outcome in control	State result of baseline outcome for treatment group	#
	Sample size baseline control	State sample size at baseline	#
	Proportion with outcome at baseline in control	State proportion with outcome at baseline in contol	#
	Number with outcome in treatment post intervention	State number with outcome post intervention for treatment group	#
	Sample size post intervention treatment	State sample size for treatment group post intervention	#
	Proportion with outcome in treatment group post intervention	State proportion with outcome post intervention in control group	#
	Number with outcome in control post intervention	State number with outcome post interventionfor control group	#
	Sample size post intervention control	State sample size for control group post intervention	#
	Proportion with outcome in control group post intervention	State proportion with outcome post intervention in control group	#
	Number with outcome in treatment first follow up	State number with outcome at first follow up for treatment group	#
	Sample size first follow up treatment	State sample size at first follow up for treatment group	#
	Proportion with outcome in treatment group first follow up	State proportion with outcome at first follow up in treatment group	#
	Number with outcome in contro first follow up	State number with outcomeat first follow up for control group	#
	Sample size first follow up control	State sample size at for control group at first follow up	#
	Proportion with outcome in contol group first follow up	State proportion with outcome at first follow up in control group	#
Regression data	OLS	OLS used?	1 = Yes, 2 = No
	Logistic	Logistic used?	1 = Yes, 2 = No
	Type of logistic	What type of logistic regression?	1 = binomial, 2 = multinomial
	GLS/WLS	GLS or WLS used?	1 = Yes, 2 = No
	Poisson	Poisson regression used?	1 = Yes, 2 = No
	Other regression types	Other regression type used? Specify	open answer
	Multilevel models	Is this a multilevel model?	1 = Yes, 2 = No
	Continous outcome	Is the outcome continous?	1 = Yes, 2 = No
	Dichotomus outcome	Is the outcome dichotomus?	1 = Yes, 2 = No
	Multiple outcome categories	Does the outcome have more than two categories?	1 = Yes, 2 = No, 3 = Continous
	Type of coefficient	What is the coefficient type?	1 = raw, 2 = standardized, 3 = other
	Coefficient	What is the coefficient estimate?	#
	Standard error	What is the standard error of the coefficient estimate?	#
	*t* test	What is the *t* statistic associated with the focal predictor?	#

## APPENDIX 3 STUDY DESIGN DETAILS AND RISK OF BIAS TOOLS

**Table:**

Risk of bias tool for impact evaluations			
	Description	Question	Coding
ID		Study	
	Unique study identification #Paper	Surname/year of first author of paper for effect size data extraction	For example, PES001Open answer
Research methods—study design and risk of bias	Design type	What type of study design is used?	1 = Randomised controlled trial (RCT) (random assignment to households/individuals) 2 = Cluster‐RCT 3 = RDD (quasiexperiment with discontinuity assignment) 4 = CBA (comparison group with baseline and endline data collection) 5 = Panel data, but no baseline 6 = Comparison group with endline data only 7 = Natural experiment 8 = Other
	Methods used for analysis	Which methods are used to control for selection bias and confounding?	1 = PSM 2 = Covariate matching 3 = DID 4 = IV‐regression 5 = Heckman selection model 6 = Fixed effects regression 7 = Other regression 8 = Randomised study
	Design and analysis method description	Briefly describe the study design and analysis method undertaken by the authors	Open answer
	Mechanism of assignment	1: Mechanism of assignment: was the allocation or identification mechanism able to control for selection bias?	1 = Yes, 2 = No, 8 = Unclear
	Mechanism of assignment	Justification for coding decision (include a brief summary of justification for rating, mentioning your response to all sub questions, cite relevant pages)	Open answer
	Group equivalence	2: Group equivalence: was the method of analysis executed adequately to ensure comparability of groups throughout the study and prevent confounding?	1 = Yes, 2 = No, 8 = Unclear
	Group equivalence	Justification for coding decision (include a brief summary of justification for rating, mentioning your response to all sub questions, cite relevant pages)	Open answer
	Spill‐overs, cross‐overs and contamination	3: Spill‐overs, cross‐overs and contamination: was the study adequately protected against spill‐overs, cross‐overs and contamination?	1 = Yes, 2 = No, 8 = Unclear
	Spill‐overs, cross‐overs and contamination	Justification for coding decision (include a brief summary of justification for rating, mentioning your response to all sub questions, cite relevant pages)	Open answer
	Outcome reporting	4: Outcome reporting: was the study free from selective outcome reporting?	1 = Yes, 2 = No, 8 = Unclear
	Outcome reporting	Justification for coding decision (include a brief summary of justification for rating, mentioning your response to all sub questions, cite relevant pages)	Open answer
	Analysis reporting	5: Analysis reporting: was the study free from selective analysis reporting?	1 = Yes, 2 = No, 8 = Unclear
	Analysis reporting	Justification for coding decision (include a brief summary of justification for rating, mentioning your response to all sub questions, cite relevant pages)	Open answer
	Performance bias	6: Performance bias: was the process of being observed free from motivation bias?	1 = Yes, 2 = No, 8 = Unclear
	Performance bias	Justification for coding decision (include a brief summary of justification for rating, mentioning your response to all sub questions, cite relevant pages)	Open answer
	Other bias	7: Other risks of bias: Is the study free from other sources of bias?	1 = Yes, 2 = No, 8 = Unclear
	Other bias	Justification for coding decision (include a brief summary of justification for rating, mentioning your response to all sub questions, cite relevant pages)	Open answer
	Type of comparison group		1 = No intervention (business as usual) 2 = Other intervention 3 = Placebo control 4 = Pipeline (wait‐list) control
	Other intervention differentially received by comparison group	Describe any nonenvironmental comparison group intervention received which treatment group does not?	Open answer
	Unit of analysis	Are there any unit of analysis errors? (e.g., the unit of analysis is different from the unit of treatement allocation and authors do not correct for these unit of analysis differences)?	1 = Yes, 2 = No, 8 = Not clear, 9 = N/A
	Blinded participants	Blinding of participants?	1 = Yes, 2 = No, 9 = N/A
	Blinded observers	Blinding of outcome assessors?	1 = Yes, 2 = No, 9 = N/A
	Blinded analysts	Blinding of data analysts	1 = Yes, 2 = No, 9 = N/A
	Method used to blind	Describe method(s) used to blind	Open answer (including describe method of placebo control)

## APPENDIX 4 DATABASE SEARCH RESULTS

**Databases for academic literature searches**

**Table:**

Database/website	Date and time	Search strategy	Number of hits
CAB Abstracts	25 August 2017	See Appendix 1	1,649
Web of Science	29 August 2017	See Appendix 1	2,222
Ebsco Discovery	30 August 2017	See Appendix 1	9,445

**Databases for grey literature searches**

**Table:**

Database/website	Date and time	Search strategy	Number of hits
African Development Bank (AfDB): https://www.afdb.org/en/documents/publications/	17 August 2017	Search for all countries in 11 indexed sectors related to the environment	4
Asian Development Bank (ADB): https://www.adb.org/publications	17 August 2017	Four free text searches using filters for evaluation, publications, papers and reports.	52
ATAI Research: https://www.atai‐research.org/emerging‐insights/?	18 August 2017	Need to manually screen all hits	42
Centre for International Forestry Research (CIFOR): http://www.cifor.org/library/	18 August 2017	Two free text searches, one filter linked to publication type applied	31
DFID Research for Development (R4D): http://r4d.dfid.gov.uk/	18 August 2017	Four free text searches, one of which used a filter for topic	485
Inter‐American Development Bank Publications: https://publications.iadb.org/facet‐view?locale‐attribute=en&field=type_view	17 August 2017	Five free text searches using no filters	8
International Food Policy Research Institute Library (IFPRI): http://library.ifpri.info/discover/collections/	18 August 2017	Three free text searches, no filters applied	62
International Institute for Environment and Development (IIED): http://pubs.iied.org/about/	18 August 2017	Four free text searches, no filters available	385
United Nations Development Programme (UNDP): http://www.undp.org/content/undp/en/home/library.html	17 August 2017	Five free text searches using filters for three sectors	55
United National Environmental Programme: http://www.unep.org/publications/	17 August 2017	Four free text searches using no filters	19
World Bank Open Knowledge Repository: https://openknowledge.worldbank.org/	18 August 2017	Four free text searches, one of which used a filter for topic	589
International Fund for Agricultural Development (IFAD): https://www.ifad.org/pub/overview	28 August 2017	Go through publication series. From thematic series: agriculture, climate change, community driven development, nutrition. Also go through IFAD occasional papers and IFAD research series	86
Food and Agriculture Organisation of the United Nations (FAO): http://www.fao.org/publications/en/	31 August 2017	Go through the following publication series: climate change, climate smart agriculture, livestock and environment, REDD+	292
3ie Repository of Impact Evaluations: http://www.3ieimpact.org/en/evidence/impact‐evaluations/	28 August 2017	Environment filter, keyword: payment for ecosystem services, keywords: payment for environmental services	173
3ie RIDIE (Registry for International Development Impact Evaluations): http://ridie.3ieimpact.org/	28 August 2017	Environment filter, keyword: payment for ecosystem services, keywords: payment for environmental services	120
Innovations for Poverty Action (IPA): http://www.poverty‐action.org/projectevaluations	28 August 2017	Environment filter, keyword: payment for ecosystem services, keywords: payment for environmental services	11
J‐Poverty Action Lab: https://www.povertyactionlab.org/evaluations	28 August 2017	Environment and energy filter	39
Conservation Evidence: http://www.conservationevidence.com/	28 August 2017	Forest conservation filter, keyword: payment for environmental services, keyword: payment for ecosystem services	122
Climate Change Agriculture and Food Security (CCAFS) publications: https://ccafs.cgiar.org/publications	28 August 2017	Keyword: payment for environmental services, payment for ecosystem services	9
Conservation International publications: http://www.conservation.org/publications/Pages/default.aspx	28 August 2017	Go through all publications	90
IUCN Library: https://portals.iucn.org/library/dir/publications‐list	28 August 2017	keywords: community management, payment for environmental services, payment for ecosystem services, payment	12
Biodiversity International: http://www.bioversityinternational.org/e‐library/publications/	28 August 2017	keywords: community management, payment for environmental services, payment for ecosystem services, payment	16
GEF evaluation database: http://www.gefieo.org/evaluations/all?f[0]=field_ieo_grouping%3A312	31 August 2017	Go through all pubs (thematic and impact)	57
AgEcon: https://ageconsearch.umn.edu/?ln=en	15 September 2017	Keywords: payment for environmental services, payment for ecosystem services	110

## APPENDIX 5 LIST OF INCLUDED STUDIES

**List of included impact evaluations**

Alix‐Garcia, J. M., Arenson, G., Radeloff, V., Ramirez‐Reyes, C., Shapiro, E., Sims, K., & Yañez‐Pagans, P., (2015b). Impacts of Mexico's payments for ecosystem services programme.

Alix‐Garcia, J. M., Sims, K. R., & Yañez‐Pagans, P. (2015a). Only one tree from each seed? Environmental effectiveness and poverty alleviation in Mexico's Payments for Ecosystem Services Program. *American Economic Journal*, *7*(4), 1–40.

Alix‐Garcia, J. M., Shapiro, E. N., & Sims, K. R. (2012). Forest conservation and slippage: Evidence from Mexico's national payments for ecosystem services program. *Land Economics*, *88*(4), 613–638.

Arriagada, R. A., Sills, E. O., Ferraro, P. J., Pattanayak, S. K. (2015). Do payments pay off? Evidence from participation in Costa Rica's PES program. *PLOS One*, *10*(7), e0131544.

Arriagada, R.A., Sills, E.O., Ferraro, P.J, & Pattanayak, S.K. (2015). Correction: Do Payments Pay Off? Evidence from Participation in Costa Rica's PES Program. *PloS one*, *10*(8), p.e0136809.

Arriagada, R. A., Sills, E. O., & Pattanayak, S.K. (2011). *Payments for environmental services and their impact on forest transition in Costa Rica* (Working Paper).

Arriagada, R. A., Villaseñor, A., Rubiano, E., Cotacachi, D., & Morrison, J. (2018). Analysing the impacts of PES programmes beyond economic rationale: Perceptions of ecosystem services provision associated to the Mexican case. *Ecosystem Services*, *29*, 116–127.

Beauchamp, E., Clements, T., & Milner‐Gulland, E.J. (2018). Assessing medium‐term impacts of conservation interventions on local livelihoods in Northern Cambodia. *World Development*, *101*, 202–218.

Chervier, C., & Costedoat, S. (2017a). Heterogeneous impact of a collective payment for environmental services scheme on reducing deforestation in Cambodia. *World Development*, *98*, 148–159.

Chervier, C., Le Velly, G., & Ezzine‐de‐Blas, D. (2017b). When the implementation of payments for biodiversity conservation leads to motivation crowding‐out: A case study from the cardamoms forests, Cambodia. *Ecological Economics*.

Clements, T., & Milner‐Gulland, E. J. (2015). Impact of payments for environmental services and protected areas on local livelihoods and forest conservation in northern Cambodia. *Conservation Biology*, *29*(1), 78–87.

Clements, T. (2013). *Money for something? Investigating the effectiveness of biodiversity conservation interventions in the Northern Plains of Cambodia* (Doctoral dissertation). University of Cambridge.

Costedoat, S., Corbera, E., Ezzine‐de‐Blas, D., Honey‐Rosés, J., Baylis, K., & Castillo‐Santiago, M.A. (2015). How effective are biodiversity conservation payments in Mexico? *PLOS one*, *10*(3), e0119881.

Démurger, S., & Wan, H. (2012). Payments for ecological restoration and internal migration in China: The sloping land conversion program in Ningxia. *IZA Journal of Migration*, *1*(1), 10.

Duan, W., Lang, Z., & Wen, Y. (2015). The effects of the sloping land conversion program on poverty alleviation in the Wuling mountainous area of China. *Small‐scale Forestry*, *14*(3), 331–350.

Garbach, K., Lubell, M., & DeClerck, F.A. (2012). Payment for ecosystem services: The roles of positive incentives and information sharing in stimulating adoption of silvopastoral conservation practices. *Agriculture, Ecosystems and Environment*, *156*, 27–36.

Garbach, K.M. (2012). *Linking social and ecological systems to sustain ecosystem services in a tropical landscape*. University of California, Davis.

Groom, B., Grosjean, P., Kontoleon, A., Swanson, T., & Zhang, S. (2010). Relaxing rural constraints: A ‘win‐win'policy for poverty and environment in China? *Oxford Economic Papers*, *62*(1), 132–156.

Groom, B., Grosjean, P., Kontoleon, A., Swanson, T., & Zhang, S. (2006). Relaxing rural constraints: A ‘win‐win'policy for poverty and environment in China? *Oxford Economic Papers*.

Groom, B., & Palmer, C. (2012). REDD+ and rural livelihoods. *Biological Conservation*, *154*, 42–52.

Hayes, T., Murtinho, F., & Wolff, H. (2017). The impact of payments for environmental services on communal lands: an analysis of the factors driving household land‐use behavior in Ecuador. *World Development*, *93*, 427–446.

Hegde, R. & Bull, G.Q. (2011). Performance of an agro‐forestry based payments‐for‐environmental‐services project in Mozambique: A household level analysis. *Ecological Economics*, *71*, 122–130.

Hegde, R. (2010). *Payments for ecosystem services and farm household behaviour: The case of carbon in Mozambique's agroforests* (Doctoral dissertation). University of British Columbia.

Honey‐Roses, J. O. R. D. I., Baylis, K., & Ramirez, M. I. (2011). A spatially explicit estimate of avoided forest loss. *Conservation biology*, *25*(5), 1032–1043.

Baylis, K., Honey‐Rosés, J., & Ramírez, M. I. (2012, September). Conserving forests: Mandates, management or money? *2012 Annual Meeting, August* (pp. 12–14).

Jack, B. K. (2013). Private information and the allocation of land use subsidies in Malawi. *American Economic Journal*, *5*(3), 113–35.

Jack, B. K. & Santos, E. C. (2017). The leakage and livelihood impacts of PES contracts: A targeting experiment in Malawi. *Land Use Policy*, *63*, 645–658.

Jayachandran, S., De Laat, J., Lambin, E. F., Stanton, C. Y., Audy, R., & Thomas, N. E. (2017). Cash for carbon: A randomized trial of payments for ecosystem services to reduce deforestation. *Science*, *357*(6348), 267–273.

Jayachandran, S., De Laat, J., Lambin, E. F., & Stanton, C. Y. (2016). *Cash for carbon: A randomized controlled trial of payments for ecosystem services to reduce deforestation* (No. w22378). National Bureau of Economic Research.

Jindal R., Kerr J. M., Carter S. (2012). Reducing poverty through carbon forestry? Impacts of the N'hambita community carbon project in Mozambique. *World Development. 40*(10), 2123–2135.

Jindal R. *Measuring the socio‐economic impact of carbon sequestration on local communities: An assessment study with specific reference to the Nhambita pilot project in Mozambique* (Doctoral dissertation) University of Edinburgh.

Jones, K. W., Holland, M. B., Naughton‐Treves, L., Morales, M., Suarez, L., & Keenan, K. (2017). Forest conservation incentives and deforestation in the Ecuadorian Amazon. *Environmental Conservation*, *44*(1), 56–65.

Jones, K. W. & Lewis, D. J. (2015). Estimating the counterfactual impact of conservation programs on land cover outcomes: The role of matching and panel regression techniques. *PLOS One*, *10*(10), e0141380.

Kwayu, E. J., Paavola, J., & Sallu, S. M. (2017). The livelihood impacts of the equitable payments for watershed services (EPWS) program in Morogoro, Tanzania. *Environment and Development Economics*, *22*(3), 328–349.

Le Velly G, Sauquet A, Cortina‐Villar S. (2017). PES impact and leakages over several cohorts: The case of the PSA‐H in Yucatan, Mexico. Land Economics. *93*(2), 230–257.

Le Velly, G. (2015). *The effectiveness of payments for environmental services in Mexican community forests* (Doctoral dissertation). Université d'Auvergne‐Clermont‐Ferrand I.

Liang, Y., Li, S., Feldman, M. W., & Daily, G. C. (2012). Does household composition matter? The impact of the Grain for Green Program on rural livelihoods in China. *Ecological Economics*, *75*, 152–160.

Li, J., Feldman, M. W., Li, S., & Daily, G. C. (2011). Rural household income and inequality under the sloping land conversion program in western China. *Proceedings of the National Academy of Sciences*, *108*(19), 7721–7726.

Li, J., Feldman, M. W., Li, S., & Daily, G. C. (2011). Supporting information: Rural household income and inequality under the sloping land conversion program in western China. *Proceedings of the National Academy of Sciences*, *108*(19), 1–3

Lin, Y. & Yao, S. (2014). Impact of the sloping land conversion program on rural household income: An integrated estimation. *Land Use Policy*, *40*, 56–63.

Liu, C., Mullan, K., Liu, H., Zhu, W., & Rong, Q. (2014). The estimation of long term impacts of China's key priority forestry programs on rural household incomes. *Journal of Forest Economics*, *20*(3), 267–285.

Liu, C., Lü, J., & Yin, R. (2010). An estimation of the effects of China's forestry programs on farmers' income. *An Integrated Assessment of China*'*s Ecological Restoration Programs* (pp. 201–218). Dordrecht: Springer.

Liu, C., Wang, S., Liu, H., & Zhu, W. (2013). The impact of China's priority forest programs on rural households' income mobility. *Land Use Policy*, *31*, 237–248.

Liu, Y., Yao, S., & Lin, Y. (2018). Effect of key priority forestry programs on off‐farm employment: Evidence from Chinese rural households. *Forest Policy and Economics*, *88*, 24–37.

Liu, C., Wang, S., Liu, H., & Zhu, W. (2013). The impact of China's priority forest programs on rural households' income mobility. *Land Use Policy*, *31*, 237–248.

Liu, Y., Yao, S., & Lin, Y. (2018). Effect of key priority forestry programs on off‐farm employment: Evidence from Chinese rural households. *Forest Policy and Economics*, *88*, 24–37.

Liu, Z., Gong, Y., & Kontoleon, A. (2018). How do payments for environmental services affect land tenure? Theory and evidence from China. *Ecological Economics*, *144*, 195–213.

Liu, Z. & Lan, J. (2015). The sloping land conversion program in China: Effect on the livelihood diversification of rural households. *World Development*, *70*, 147–161.

Liu, Z. & Lan, J. (2014). *The Sloping Land Conversion Program in China: Effect on rural households' livelihood diversification* (No. 2014/07). University of Copenhagen, Department of Food and Resource Economics.

Lokina, R. B. & John, I. (2016). Welfare implications of the payment for environmental services: Case of Uluguru Mountain–Morogoro. *African Journal of Economic Review*, *4*(1), 61–85.

John, I. (2012). *How Successful has Payment for Environmental Services Improved Welfare: The Case of Uluguru Mountain‐Morogoro* (Masters dissertation). University of Dar es Salam.

Mohebalian, P. M. & Aguilar, F. X. (2016). Additionality and design of forest conservation programs: Insights from Ecuador's Socio Bosque program. *Forest Policy and Economics*, *71*, 103–114.

Mohebalian, P. M. & Aguilar, F. X. (2018). Beneath the canopy: Tropical forests enrolled in conservation payments reveal evidence of less degradation. *Ecological Economics*, *143*, 64–73.

Pagiola, S. & Rios, A. R. (2013). Evaluation of the impact of payments for environmental services on land use change in Quindío, Colombia.

Pagiola, S., Ramírez, E., Gobbi, J., de Haan, C., Ibrahim, M., Murgueitio, E., & Ruíz, J. P. (2007). Paying for the environmental services of silvopastoral practices in Nicaragua. *Ecological Economics*, *64*(2), 374–385.

Pagiola, S., Honey‐Rosés, J., & Freire‐González, J. (2016). Evaluation of the permanence of land use change induced by payments for environmental services in Quindío, Colombia. *PLOS One*, *11*(3), e0147829.

Robalino, J. & Pfaff, A. (2013). Ecopayments and deforestation in Costa Rica: A nationwide analysis of PSA's initial years. *Land Economics*, *89*(3), 432–448.

Pfaff, A., Robalino, J. A., & Sanchez‐Azofeifa, G. A. (2008). *Payments for environmental services: Empirical analysis for Costa Rica* (pp. 404–424). Terry Sanford Institute of Public Policy, Duke University, Durham, NC.

Robalino, J., Sandoval, C., Barton, D.N., Chacon. A., Pfaff. A. (2015). Evaluating interactions of forest conservation policies on avoided deforestation. *PLOS One*, *10*(4), e0124910.

Robalino, J., Sandoval, C., Villalobos, L., & Alpízar, F. (2014). *Local effects of payments for environmental services on poverty* (No. dp‐14‐12‐efd).

Scullion, J., Thomas, C. W., Vogt, K. A., Pérez‐Maqueo, O., & Logsdon, M. G. (2011). Evaluating the environmental impact of payments for ecosystem services in Coatepec (Mexico) using remote sensing and on‐site interviews. *Environmental Conservation*, *38*(4), 426–434.

Sharma, B. P. & Pattanayak, S. (2015). *REDD+ impacts: Evidence from Nepal*. South Asian Network for Development and Environmental Economics.

Sierra, R. & Russman, E. (2006). On the efficiency of environmental service payments: A forest conservation assessment in the Osa Peninsula, Costa Rica. *Ecological economics*, *59*(1), 131–141.

Simonet, G., Subervie, J., Ezzine‐de‐Blas, D., Cromberg, M., & Duchelle, A. (2017). *Paying smallholders not to cut down the Amazon forest: Impact evaluation of a REDD+ pilot project*, *14*.

Sims, K. R. & Alix‐Garcia, J. M. (2017). Parks versus PES: Evaluating direct and incentive‐based land conservation in Mexico. *Journal of Environmental Economics and Management*, *86*, 8–28.

Uchida, E., Rozelle, S., & Xu, J. (2009). Conservation payments, liquidity constraints, and off‐farm labor: Impact of the grain‐for‐green program on rural households in China. *American Journal of Agricultural Economics*, *91*(1), 70–86.

Uchida, E., Xu, J., Xu, Z., & Rozelle, S. (2007). Are the poor benefiting from China's land conservation program? *Environment and Development Economics*, *12*(4), 593–620.

Xu, J., Tao, R., Xu, Z., & Bennett, M. T. (2010). China's sloping land conversion program: Does expansion equal success? *Land Economics*, *86*(2), 219–244.

Yao, S., Guo, Y., & Huo, X. (2010). An empirical analysis of the effects of China's land conversion program on farmers' income growth and labor transfer. *Environmental Management*, *45*(3), 502–512.

Zhang, W. & Liu, C. (2005, July). The impact of environmental policy on household income and activity choice: Evidence from Sandstorm source control program in North China. *American Agricultural Economics Association Annual Meeting, Providence, RI* (pp. 24–27).

Zheng, H., Robinson, B. E., Liang, Y. C., Polasky, S., Ma, D. C., Wang, F. C., Ruckelshaus, M., Ouyang, Z. Y., & Daily, G. C. (2013). Benefits, costs, and livelihood implications of a regional payment for ecosystem service program. *Proceedings of the National Academy of Sciences*, *110*(41), 16681–16686.

**List of included qualitative studies, descriptive quantitative studies and process evaluations**

Alix‐Garcia, J., Janvry, A. De, Sadoulet, E., Manuel, J., & Torres, J. M. (2009). 10 lessons learned from Mexico's payment for environmental services program. In L. Lipper, T. Sakuyama, R. Stringer, & D. Zilberman (Eds.), *Payment for Environmental Services in Agricultural Landscapes* (1951, pp. 1–27). https://doi.org/10.1007/978‐0‐387‐72971‐8

Ajayi, O. C., Jack, B. K., & Leimona, B. (2012). Auction design for the private provision of public goods in developing countries: Lessons from payments for environmental services in Malawi and Indonesia. *World development*, *40*(6), 1213–1223. https://doi.org/10.1016/j.worlddev.2011.12.007

Bennett, M. T. (2008). China's sloping land conversion program: Institutional innovation or business as usual? *Ecological Economics*, *65*(4), 699–711. https://doi.org/10.1016/j.ecolecon.2007.09.017

Blackman, A., & Woodward, R. T. (2010). User financing in a national payments for environmental services program: Costa Rican hydropower. *Ecological Economics*, *69*(8), 1626–1638. https://doi.org/10.1016/j.ecolecon.2010.03.004

Branca, G., Lipper, L., Neves, B., Lopa, D., & Mwanyoka, I. (2011). Payments for watershed services supporting sustainable agricultural development in Tanzania. *The Journal of Environment and Development*, *20*(3), 278–302. http://journals.sagepub.com/doi/abs/10.1177/1070496511415645

Bremer, L. L., Farley, K. A., & Lopez‐Carr, D. (2014). What factors influence participation in payment for ecosystem services programs? An evaluation of Ecuador's SocioPáramo program. *Land Use Policy*, *36*, 122–133. https://doi.org/10.1016/j.landusepol.2013.08.002

Calle, A., Montagnini, F., & Zuluaga, A. F. (2009). Farmer's perceptions of silvopastoral system promotion in Quindío, Colombia. Bois et Forêts Des Tropiques, 300(2), 79–94. http://bft.cirad.fr/cd/BFT_300_79‐94.pdf

Cao, S., Chen, L., & Liu, Z. (2009). An investigation of Chinese attitudes toward the environment: Case study using the grain for green project. *A Journal of the Human Environment*, *38*(1), 55–64. https://doi.org/10.1579/0044‐7447‐38.1.55

Clements, T., Rainey, H., An, D., Rours, V., Tan, S., Thong, S., … Milner‐Gulland, E. J. (2013). An evaluation of the effectiveness of a direct payment for biodiversity conservation: The bird nest protection program in the northern plains of Cambodia. *Biological Conservation*, *157*, 50–59. https://doi.org/10.1016/j.biocon.2012.07.020

Collen, W., Krause, T., Mundaca, L., & Nicholas, K. A. (2016). Building local institutions for national conservation programs: Lessons for developing reducing emissions from deforestation and forest degradation (REDD+) programs. *Ecology and Society*, *21*(2), 36–49. https://doi.org/10.5751/ES‐08156‐210204

Costedoat, S., Koetse, M., Corbera, E., & Ezzine‐de‐Blas, D. (2016). Cash only? Unveiling preferences for a PES contract through a choice experiment in Chiapas, Mexico. *Land Use Policy*, *58*, 302–317. https://doi.org/10.1016/j.landusepol.2016.07.023

Edwards, S., Allison, J., Cheetham, S., & Hoeun, B. (2012). Mammal and bird diversity at a salt lick in Kulen‐Promtep Wildlife Sanctuary, Northern Cambodia. *Cambodian Journal of Natural History*, *2012*(1), 56–63.

Ezzine‐De‐Blas, D., Dutilly, C., Lara‐Pulido, J. A., Le Velly, G., & Guevara‐Sanginés, A. (2016). Payments for environmental services in a policymix: Spatial and temporal articulation in Mexico. *PLOS One*, 11(4), 1–15. https://doi.org/10.1371/journal.pone.0152514

Feng, L., & Xu, J. (2015). Farmers' willingness to participate in the next‐stage grain‐for‐green project in the three gorges reservoir area, China. *Environmental Management*, *56*(2), 505–518. https://doi.org/10.1007/s00267‐015‐0505‐1

Hayes, T. M. (2012). Payment for ecosystem services, sustained behavioural change, and adaptive management: Peasant perspectives in the Colombian Andes. *Environmental Conservation*, *39*(2), 144–153. https://doi.org/10.1017/S0376892912000045

Hayes, T., Murtinho, F., & Wolff, H. (2015). An institutional analysis of payment for environmental services on collectively managed lands in Ecuador. *Ecological Economics*, *118*, 81–89. https://doi.org/10.1016/j.ecolecon.2015.07.017

Hegde, R., Bull, G. Q., Wunder, S., & Kozak, R. A. (2015). Household participation in a payments for environmental services programme: The Nhambita Forest Carbon Project (Mozambique). *Environment and Development Economics*, *20*(5), 611–629. https://doi.org/10.1017/S1355770X14000631

Honey‐Rosés, J., López‐García, J., Rendón‐Salinas, E., Peralta‐Higuera, A., & Galindo‐Leal, C. (2009). To pay or not to pay? Monitoring performance and enforcing conditionality when paying for forest conservation in Mexico. *Environmental Conservation*, *36*(2), 120–128. https://doi.org/10.1017/S0376892909990063

Jayachandran, S. (2013). American economic association liquidity constraints and deforestation: The limitations of payments for ecosystem. *The American Economic Review*, *103*(3), 309–313. https://doi.org/10.1257/aer.l03.3.309

Krause, T., Collen, W., & Nicholas, K. A. (2013). Evaluating safeguards in a conservation incentive program: Participation, consent, and benefit sharing in indigenous communities of the Ecuadorian Amazon. *Ecology and Society*, *18*(4). https://doi.org/10.5751/ES‐05733‐180401

Krause, T., & Zambonino, H. (2013). More than just trees‐Animal species diversity and participatory forest monitoring in the Ecuadorian Amazon. *International Journal of Biodiversity Science, Ecosystem Services and Management*, *9*(3), 225–238. https://doi.org/10.1080/21513732.2013.822930

Legrand, T., Froger, G., & Le Coq, J. F. (2013). Institutional performance of payments for environmental services: An analysis of the Costa Rican program. *Forest Policy and Economics*, *37*, 115–123. https://doi.org/10.1016/j.forpol.2013.06.016

Li, Q., Amjath‐Babu, T. S., & Zander, P. (2016). Role of capitals and capabilities in ensuring economic resilience of land conservation efforts: A case study of the grain for green project in China's Loess Hills. *Ecological Indicators*, *71*, 636–644. https://doi.org/10.1016/j.ecolind.2016.07.027

Lopa, D., Mwanyoka, I., Jambiya, G., Massoud, T., Harrison, P. A. U. L., Ellis‐Jones, M., … & Burgess, N. D. (2012). Towards operational payments for water ecosystem services in Tanzania: A case study from the Uluguru Mountains. *Oryx*, *46*(1), 34–44. https://doi.org/10.1017/S0030605311001335

Master, J. E., & Studies, E. (2009). Socio‐environmental approach to drip irrigation system implementation as a climate change adaptation measure within N'hambita community carbon project area, Mozambique.

Milne, S., & Adams, B. (2012). Market masquerades: Uncovering the politics of community‐level payments for environmental services in Cambodia. *Development and Change*, *43*(1), 133–158. https://doi.org/10.1111/j.1467‐7660.2011.01748.x

Missrie, M., & Nelson, K. (2005). *Direct payments for conservation: Lessons from the Monarch butterfly conservation fund* (8).

Mudaca, J. D., Tsuchiya, T., Yamada, M., & Onwona‐Agyeman, S. (2015). Household participation in payments for ecosystem services: A case study from Mozambique. *Forest Policy and Economics*, *55*, 21–27. https://doi.org/10.1016/j.forpol.2015.03.002

Muñoz‐Piña, C., Guevara, A., Torres, J. M., & Braña, J. (2008). Paying for the hydrological services of Mexico's forests: Analysis, negotiations and results. *Ecological Economics*, *65*(4), 725–736. https://doi.org/10.1016/j.ecolecon.2007.07.031

Murtinho, F., & Hayes, T. (2017). Communal participation in payment for environmental services (PES): Unpacking the collective decision to enroll. *Environmental Management*, *59*(6), 939–955. https://doi.org/10.1007/s00267‐017‐0838‐z

Pagiola, S., Agostini, P., Gobbi, J., de Haan, C., Ibrahim, M., Murgueitio, E., … Ruíz, J. P. (2005). Paying for biodiversity conservation services. *Mountain Research and Development*, *25*(3), 206–211. https://doi.org/10.1659/0276‐4741(2005)025[0206:PFBCS]2.0.CO;2

Pagiola, S., Rios, A. R., & Arcenas, A. (2010). Poor household participation in payments for environmental services: Lessons from the silvopastoral project in Quindío, Colombia. *Environmental and Resource Economics*, *47*(3), 371–394. https://doi.org/10.1007/s10640‐010‐9383‐4

Porras, I. (2010). Fair and green?: Social impacts of payments for environmental services in Costa Rica. IIED. Chandara, P., & Sophat, S. (2015). The contribution of local ecological knowledge and practices to waterbird conservation along the Sekong and Sesan River IBAs, Cambodia. https://doi.org/10.13140/RG.2.2.11174.11848

Sharma, B. P., Shyamsundar, P., Nepal, M., Pattanayak, S. K., & Karky, B. S. (2017). Costs, cobenefits, and community responses to REDD plus: A case study from Nepal. *Ecology and Society*, *22*(2). https://doi.org/10.5751/ES‐09370‐220234

Shen, H., Zhang, W., Cao, J., Zhang, X., Xu, Q., Yang, X., … Zhao, Y. (2016). Carbon concentrations of components of trees in 10‐year‐old *Populus davidiana* stands within the desertification combating program of Northern China. *Frontiers of Earth Science*, *10*(4), 662–668. https://doi.org/10.1007/s11707‐016‐0562‐7

Shrestha, S., Karky, B. S., & Karki, S. (2014). Case study report: REDD+ pilot project in community forests in three watersheds of Nepal. *Forests*, *5*(10), 2425–2439. https://doi.org/10.3390/f5102425

Sims, K. R. E., Alix‐Garcia, J. M., Shapiro‐Garza, E., Fine, L. R., Radeloff, V. C., Aronson, G., … Yañez‐Pagans, P. (2014). Improving environmental and social targeting through adaptive management in Mexico's payments for hydrological services program. *Conservation Biology*, *28*(5), 1151–1159. https://doi.org/10.1111/cobi.12318

Spiric, J. (2009). Investigating the socio‐economic impact of REDD scheme implemented in the Nhambita community carbon project, Mozambique.

The World Bank (2008, November). *For the integrated silvopastoral approaches to ecosystem management project in Colombia, Costa Rica, and Nicaragua* (Document of The World Bank Report No: ICR0000875), Environmentally and Socially Sustainable Development Central American Depa.

Tu, Q., Mol, A. P. J., Zhang, L., & Ruben, R. (2011). How do trust and property security influence household contributions to public goods? The case of the sloping land conversion program in China. *China Economic Review*, *22*(4), 499–511. https://doi.org/10.1016/j.chieco.2011.07.011

Uprety, D. R., Luintel, H., & Bhandari, K. (2011). REDD + and conflict: A case study of the REDD + projects in Nepal, 1–34.

Woodhouse, E. (2015). Local experiences and contested meanings of the Chinese “grain for green” land conversion programme in an agro‐pastoralist Tibetan community. *Nomadic Peoples*, *19*(2), 281–302. https://doi.org/10.3197/np.2015.190208

Xu, J., Yin, R., Li, Z., & Liu, C. (2006). China's ecological rehabilitation: Unprecedented efforts, dramatic impacts, and requisite policies. *Ecological Economics*, *57*(4), 595–607. https://doi.org/10.1016/j.ecolecon.2005.05.008

Yin, R., Liu, H., Liu, C., & Lu, G. (2018). Households' decisions to participate in China's sloping land conversion program and reallocate their labour times: Is there endogeneity bias? *Ecological Economics*, *145*, 380–390. https://doi.org/10.1016/j.ecolecon.2017.11.020

Yin, R., Liu, T., Yao, S., & Zhao, M. (2013). Designing and implementing payments for ecosystem services programs: Lessons learned from China's cropland restoration experience. *Forest Policy and Economics*, *35*, 66–72. https://doi.org/10.1016/j.forpol.2013.06.010

Yuan, Y., Liu, Y., Hu, Y. N., Chen, X., & Peng, J. (2017). Identification of non‐economic influencing factors affecting farmer's participation in the paddy landto‐dry land program in Chicheng County, China. *Sustainability*, *9*(3), 366. doi:10.3390/su9030366

Zbinden, S., & Lee, D. R. (2005). Paying for environmental services: An analysis of participation in Costa Rica's PSA program. *World Development*, *33*(2 Spec. Iss.), 255–272. https://doi.org/10.1016/j.worlddev.2004.07.012

Zhang, L., Tu, Q., & Mol, A. P. J. (2008). Payment for environmental services: The sloping land conversion program in Ningxia Autonomous Region of China. *China and World Economy*, *16*(2), 66–81. https://doi.org/10.1111/j.1749‐124X.2008.00107.x

## APPENDIX 6 FULL RESULTS OF RISK OF BIAS ASSESSMENT

**Risk of bias assessment by each criterion for included impact evaluations**

**Table:**

Surname/year of first author of main paper	1: Mechanism of assignment: was the allocation or identification mechanism able to control for selection bias?	2. Group equivalence: was the method of analysis executed adequately to ensure comparability of groups throughout the study and prevent confounding?	3: Performance bias: was the process of being observed free from motivation bias?	4: Spill‐overs, cross‐overs and contamination: was the study adequately protected against spill‐overs, cross‐overs and contamination?	5: Selective outcome reporting: was the study free from selective outcome reporting?	6: Selective analysis reporting: was the study free from selective analysis reporting?	7: Other risks of bias: Is the study free from other sources of bias?	Are there any unit of analysis errors?	Final RoB Assessment
Hedge & Bull, 2011	No	No	Yes	No	Yes	No	Yes	No	Critical
Jindal, 2012	No	No	Yes	Yes	Yes	No	Yes	No	Critical
Garbach	Unclear	No	No	Unclear	Unclear	Unclear	Yes	No	Critical
Honey‐Roses (2011)	Unclear	Yes	Yes	Yes	Yes	Yes	Yes	No	Medium
Beauchamp, 2018	Unclear	Unclear	Yes	No	Yes	Yes	Unclear	No	Critical
Sharma, 2015	Unclear	Unclear	Yes	No	Yes	Yes	Yes	No	High
Arriagada, 2011	Unclear	No	Yes	Unclear	Yes	Unclear	Yes	No	High
Arriagada, 2012	Yes	Yes	Yes	Yes	Yes	Yes	Yes	No	Low
Arriagada, 2015	Yes	Yes	Yes	Unclear	Yes	Unclear	Yes	No	Medium
Robalino, 2013	No	Unclear	Yes	No	Yes	Yes	Yes	Unclear	High
Robalino, 2014	Unclear	No	Yes	Unclear	Yes	Unclear	Yes	Unclear	High
Robalino, 2015—also checked Robalino 2008	No	Unclear	Yes	No	Yes	Unclear	Unclear	Unclear	Critical
Sierra, 2006	No	No	Yes	No	Yes	Unclear	Unclear	No	Critical
Alix‐Garcia, 2015a	Yes	Yes	Yes	Unclear	Yes	Yes	Yes	No	Medium
Arriagada, 2018	Unclear	Unclear	Yes	No	Yes	Yes	Unclear	Unclear	High
Le Velley 2017—Le Velley 2015 also checked	Yes	Yes	Yes	Yes	Yes	Yes	Yes	No	Low
Scullion 2011	No	No	Yes	No	Yes	No	Yes	Unclear	Critical
Sims 2017	Unclear	Unclear	Yes	Yes	Yes	Yes	Yes	No	High
Liu, 2015	No	No	Yes	No	Yes	Unclear	Unclear	No	Critical
Liu, 2014	No	Unclear	Yes	No	Yes	Unclear	Unclear	No	Critical
Lin, 2014	No	No	Yes	No	Yes	Unclear	Unclear	No	Critical
Liu, 2018	Unclear	Unclear	Yes	No	Yes	Unclear	Unclear	No	Critical
Demurger, 2012	Unclear	No	Yes	Unclear	Yes	Unclear	Unclear	No	High
Duan, 2015	No	No	Yes	No	No	No	Unclear	No	Critical
Groom, 2010	Unclear	No	Yes	No	Yes	Yes	No	No	Critical
Liang, 2012	No	No	Yes	No	Yes	Unclear	Unclear	No	Critical
Uchida, 2009	No	No	Yes	No	No	Yes	Unclear	No	Critical
Xu, 2010	Unclear	Unclear	Yes	No	Yes	Unclear	Unclear	No	Critical
Yao, 2010	Unclear	No	Yes	No	Yes	Unclear	Unclear	No	Critical
Kwayu, 2017	No	No	Yes	No	Yes	Unclear	Unclear	Unclear	Critical
Lokina, 2016	No	No	Yes	No	Yes	Yes	Yes	Unclear	Critical
Hayes, 2017	Unclear	No	Yes	No	Yes	Yes	Yes	No	Critical
Jones, 2016	Unclear	Unclear	Yes	No	Yes	Yes	Yes	No	High
Mohebalian, 2016	Unclear	Unclear	Yes	No	Yes	Yes	Yes	No	High
Mohebalian, 2018	Unclear	Unclear	Yes	No	Yes	Yes	Yes	No	High
Jayachandran et al., 2017	Yes	Yes	Yes	Yes	Yes	Yes	Yes	No	Low
Pagiola, 2013 (Pagiola, 2016 also checked)	Unclear	Unclear	Yes	Unclear	Yes	Yes	Yes	No	High
Chervier, 2017a	Unclear	Unclear	Yes	Yes	Yes	Yes	Yes	Unclear	High
Chervier, 2017b	No	Unclear	Yes	Yes	No	Yes	Yes	Unclear	High
Zhang, 2013	Unclear	No	Yes	No	Yes	Unclear	Yes	No	Critical
Jack, 2017	Yes	Yes	Yes	Yes	Yes	Yes	Yes	No	Low
Simonet, 2017	No	Yes	Yes	Yes	Yes	Yes	Yes	No	High
Zhang, 2005	Unclear	No	Yes	No	Yes	Unclear	Unclear	No	Critical
Liu, 2014	No	Unclear	Yes	No	Yes	Unclear	Yes	No	Critical
Costedoat, 2015	Unclear	Yes	Yes	Yes	Yes	Yes	Yes	Unclear	Medium

**Full Results of qualitative critical appraisal**

**Table:**

Surname/year of first author of main paper	Critical screening criterion				1: Research design is defensible	2. Research features an appropriate sample	3: Research is rigorous in conduct	4: Research findings are credible in claim/based on data	5: Research attends to contexts	6: Research is reflexive	Final critical appraisal
	Primary data and applied methods	Research question & objective	Fit between design & question	Findings based on data							
Hedge (2015)	Y	Y	Y	Y	Defensible	Appropriate	Rigorous	Credible	n/a	n/a	High quality
Mudaca (2011)	Y	Y	Y	Y	Defensible	Functional	Critical	Credible	n/a	n/a	Moderate quality
Spiric (2009)	Y	Y	Y	Y	Arguable	Appropriate	Rigorous	Arguable	Considered	Acknowledged	Moderate quality
Barbir (2009)	Y	Y	Y	Y	Critical	Appropriate	Critical	Credible	Considered	No reflection	Low quality
W0lrd Bank (2008)	Y	Y	Y	Y	Defensible	Appropriate	Rigorous	Credible	n/a	n/a	High quality
Honey‐Roses (2009)	Y	Y	Y	Y	Defensible	Appropriate	Rigorous	Credible	n/a	n/a	High quality
Missrie (2005)	Y	Y	Y	Y	Arguable	Critical	Flawed	n/a	n/a	n/a	Critical quality
Clements (2013)	Y	Y	Y	Y	Arguable	Critical	Critical	Credible	No attention	No reflection	Low quality
Ko (2012)	N	Y	N	Y	n/a	n/a	n/a	n/a	n/a	n/a	Critical quality
Chandara (2011)	Y	Y	Y	Y	Defensible	Critical	Critical	Credible	n/a	n/a	Low quality
Shresta (2014)	Y	Y	N	N	n/a	n/a	n/a	n/a	n/a	n/a	Critical quality
Uprey (2011)	Y	Y	Y	Y	Defensible	Appropriate	Critical	Doubtful	Mentioned	No reflection	Low quality
Sharma (2017)	Y	Y	N	Y	n/a	n/a	n/a	n/a	n/a	n/a	Critical quality
Bossel (2013)	Y	Y	Y	Y	Defensible	Functional	Considerate	Credible	n/a	n/a	Moderate quality
Le Coq (2013)	N	Y	N	Y	n/a	n/a	n/a	n/a	n/a	n/a	Critical quality
Le Coq (2015)	Y	Y	Y	Y	Defensible	Appropriate	Rigorous	Credible	Considered	Acknowledged	High quality
Bosma (2012)	Y	Y	Y	Y	Defensible	Critical	Critical	Doubtful	Mentioned	Considered	Low quality
Murillo (2014)	Y	Y	Y	Y	Defensible	Critical	Critical	Credible	n/a	n/a	Low quality
Porras (2010)	N	Y	N	N	n/a	n/a	n/a	n/a	n/a	n/a	Critical quality
Blackman (2010)	Y	Y	Y	Y	Arguable	Appropriate	Rigorous	Arguable	n/a	n/a	Moderate quality
Legrand (2013)	Y	Y	Y	Y	Critical	Appropriate	Considerate	Doubtful	n/a	n/a	Low quality
Zbinden (2005)	Y	Y	Y	Y	Defensible	Appropriate	Critical	Credible	n/a	n/a	Moderate quality
Ezzine‐de‐Blas (2012)	Y	Y	Y	Y	Defensible	Functional	Considerate	Credible	n/a	n/a	Moderate quality
Sims (2014)	Y	Y	Y	Y	Defensible	Functional	Considerate	Credible	n/a	n/a	Moderate quality
Munoz‐Pinaet (2007)	N	N	N	N	n/a	n/a	n/a	n/a	n/a	n/a	Critical quality
Alix‐Garcia (2009)	Y	Y	N	N	n/a	n/a	n/a	n/a	n/a	n/a	Critical quality
Tu (2012)	Y	Y	Y	Y	Defensible	Critical	Critical	Credible	n/a	n/a	Low quality
Xu (2006)	N	Y	N	N	n/a	n/a	n/a	n/a	n/a	n/a	Critical quality
Yin (2013)	N	Y	N	N	n/a	n/a	n/a	n/a	n/a	n/a	Critical quality
Li (2016)	Y	Y	Y	Y	Defensible	Appropriate	Critical	Credible	n/a	n/a	Moderate quality
Bennett (2008)	Y	Y	N	N	n/a	n/a	n/a	n/a	n/a	n/a	Critical quality
Yin (2018)	Y	Y	Y	Y	Defensible	Appropriate	Rigorous	Credible	n/a	n/a	High quality
Cao (2009)	Y	Y	Y	Y	Arguable	Appropriate	Rigorous	Arguable	n/a	n/a	Moderate quality
Zhang (2008)	N	Y	N	N	n/a	n/a	n/a	n/a	n/a	n/a	Critical quality
Woodhouse (2015)	Y	Y	Y	Y	Defensible	Appropriate	Considerate	Doubtful	Considered	Acknowledged	Low quality
Feng (2015)	Y	Y	Y	Y	Defensible	Appropriate	Critical	Credible	n/a	n/a	Moderate quality
Branca (2011)	N	Y	N	N	n/a	n/a	n/a	n/a	n/a	n/a	Critical quality
Kwayu (2017)	N	Y	Y	N	n/a	n/a	n/a	n/a	n/a	n/a	Critical quality
Lopa (2012)	N	Y	Y	N	n/a	n/a	n/a	n/a	n/a	n/a	Critical quality
Bremer (2013)	Y	Y	Y	Y	Arguable	Functional	Flawed	n/a	n/a	n/a	Critical quality
Murtinho (2017)	Y	Y	Y	Y	Defensible	Appropriate	Critical	Doubtful	Considered	Acknowledged	Low quality
Hayes (2015)	Y	Y	Y	Y	Defensible	Appropriate	Rigorous	Credible	n/a	n/a	High quality
Krause (2013a)	Y	Y	Y	Y	Defensible	Appropriate	Critical	Doubtful	Considered	Acknowledged	Low quality
Kraus (2013b)	Y	Y	Y	Y	Defensible	Appropriate	Rigorous	Credible	Considered	Acknowledged	High quality
Collen (2016)	Y	Y	Y	Y	Defensible	Appropriate	Rigorous	Credible	Considered	Acknowledged	High quality
Jayachandran (2014)	Y	Y	Y	Y	Defensible	Appropriate	Rigorous	Credible	n/a	n/a	High quality
Calle (2009)	Y	Y	Y	Y	Defensible	Critical	Critical	Credible	Considered	No reflection	Low quality
Hayes (2012)	Y	Y	Y	Y	Defensible	Appropriate	Considerate	Arguable	Not attention	No reflection	Moderate quality
Pagiola (2005)	N	Y	Y	Y	n/a	n/a	n/a	n/a	n/a	n/a	Critical quality
Pagiola (2010)	Y	Y	Y	Y	Defensible	Appropriate	Rigorous	Credible	n/a	n/a	High quality
Milne (2012)	Y	Y	Y	Y	Critical	Critical	Flawed	Not credible	n/a	n/a	Critical quality
Yuan (2017)	Y	Y	Y	Y	Defensible	Functional	Considerate	Credible	n/a	n/a	Moderate quality
Ajayi (2012)	Y	Y	Y	Y	Critical	Appropriate	Critical	Arguable	Considered	Acknowledged	Low quality
Lopez (2017)	N	Y	Y	N	n/a	n/a	n/a	n/a	n/a	n/a	Critical quality
Chen (2016	Y	Y	Y	Y	Arguable	Appropriate	Rigorous	Credible	n/a	n/a	High quality
Costeodat (2016)	Y	Y	Y	Y	Defensible	Appropriate	Rigorous	Credible	n/a	n/a	High quality

Summary of critical appraisal category ratings across studies included in the qualitative synthesis*
*Excluding studies rated as of critical quality.

## APPENDIX 8 EFFECT SIZES NOT INCLUDED IN THE META‐ANALYSIS

**Intermediate outcome**

*Agricultural behaviour*

**Table:**

Study	Outcome	*N*	Nt	Nc	Unit of analysis	TREATeff	Effect size	Variance	Lower bound	Upper bound
Zheng2013 China PLDL	Agricultural intensification, person‐days/mu	723	394	329	Household	ATT	−0.4969	0.0057	−0.6455	−0.3483
Zheng2013 China PLDL	Phosporus application, kg/mu	723	394	329	Household	ATT	0.1611	0.0056	0.0145	0.3078
Zheng2013 China PLDL	Nitrogen application, kg/mu	723	394	329	Household	ATT	0.0836	0.0056	−0.0629	0.2300
Sharma2017 Nepal Redd++	Open grazing signs observed in the sampled forest plots	554	306	248	Plot	ATE	0.0664	0.0073	−0.1011	0.2339
Simonet2017 Brazil PAS	Cattle ranching outcome is the ratio of the value of total livestock owned to pasture in 2014; it is expressed in Reais per hectare.	181	106	75	Household	ITT	0.1362	0.0228	−0.1599	0.4323
Arriagada2015 Costa Rica PSA	Change in cattle owned between 1996 and 2005, Change in hired labor since 1996 (dummy variable: 1 indicates no hired labor in 1996 and hired labor in 2005)	80	40	40	Plot	ATT	−0.9602	0.0558	−1.4230	−0.4973
Alix‐Garcia, Sims, et al. (2015) Mexico PSAH	Number of cattle (Private properties)	228	120	108	Plot	ATE	0.0845	0.0176	−0.1755	0.3446
Alix‐Garcia2015a Mexico PSAH	Number of small animals (Private properties)	228	120	108	Plot	ATE	0.0066	0.0176	−0.2534	0.2665
Alix‐Garcia2015a Mexico PSAH	Livestock infrastructure (Private Properties)	228	120	108	Plot	ATE	0.1732	0.0177	−0.0873	0.4336
Alix‐Garcia2015a Mexico PSAH	Agricultural inputs (Private properties)	228	120	108	Plot	ATE	0.2012	0.0177	−0.0595	0.4618
Alix‐Garcia2015a Mexico PSAH	Agricultural Equipment (Private properties)	228	120	108	Plot	ATE	0.0944	0.0176	−0.1657	0.3545
Pagiola2013 Columbia Silvopastoral	PES recipient (1=yes)‐ Area changed in HA	101	72	29	Plot	ATE	0.4156	0.0492	−0.0192	0.8505
Pagiola2013 Columbia Silvopastoral	PES recipient (1=yes)‐ Proportion of Farm changed %	101	72	29	Plot	ATE	0.5187	0.0497	0.0817	0.9556
Sierra2006 Costa Rica PSA	Land use—% of land under agricultural land	60	30	30	Plot	ATE	−0.3899	0.0679	−0.9008	0.1209
Sierra2006 Costa Rica PSA	Land use—% of land under charral (scrubland)	60	30	30	Plot	ATE	0.7340	0.0712	0.2111	1.2568
Simonet2017 Brazil PAS	Crop land participants	181	106	75	Plot and Household	ITT and ATT	0.0288	0.0228	−0.2670	0.3245
Jack2017 Malawi ICRAF	Has acquired new land since 2008 (Lottery)	319	205	114	Plot	ITT	−0.1225	0.0137	−0.3517	0.1067
Jack2017 Malawi ICRAF	Has acquired new land since 2008 (Lottery)	319	205	114	Plot	ITT	−0.1854	0.0137	−0.4149	0.0440
Simonet2017 Brazil PAS	Total land (total in hectares)	181	106	75	Plot and Household	ITT and ATT	−0.0064	0.0228	−0.3022	0.2893
Hayes2017 Ecuador Socio Bosque	Household decision to stop grazing animals (cows and sheep) in the collective paramo. Specifically, asked if the household had grazed animals in the past year and if the household used the paramo for grazing in 2008 as compared to 2013 (recall)"	776	NA	NA	Plot	ATE	−0.1721	0.005	−0.313	−0.031
Alix‐Garcia2015 Mexico PSAH	Has large or small grazers	1,464	NA	NA	Plot	ATE	0.0796	0.003	−0.023	0.182
Alix‐Garcia2015 Mexico PSAH	# Large grazers (such as cattle)	1,464	NA	NA	Plot	ATE	0.1071	0.003	0.005	0.210
Alix‐Garcia2015 Mexico PSAH	Participates livestock activitites	1,464	NA	NA	Plot	ATE	0.0954	0.003	−0.007	0.198
Alix‐Garcia2015 Mexico PSAH	Quantity staples cultivated (Staples include maize and beans. Large cattle, horses, and bullocks.)	1,401	NA	NA	Plot	ATE	−0.1324	0.003	−0.237	−0.028
Alix‐Garcia2015 Mexico PSAH	Produces staples (Staples include maize and beans. Large cattle, horses, and bullocks.)	1,464	NA	NA	Plot	ATE	−0.1457	0.003	−0.248	−0.043
Alix‐Garcia2015a Mexico PSAH	Number of cattle (Common properties)	1,844	NA	NA	Plot	ATE	0.1143	0.002	0.023	0.206
Alix‐Garcia2015a Mexico PSAH	Number of small animals (Common properties)	1,844	NA	NA	Plot	ATE	−0.3185	0.002	−0.410	−0.227
Alix‐Garcia2015a Mexico PSAH	Livestock infrastructure (Common Properties)	1,844	NA	NA	Plot	ATE	0.0528	0.002	−0.039	0.144
Alix‐Garcia2015a Mexico PSAH	Agricultural inputs (Common properties)	1,844	NA	NA	Plot	ATE	−0.0130	0.002	−0.104	0.078
Alix‐Garcia2015a Mexico PSAH	Agricultural Equipment (Common properties)	1,844	NA	NA	Plot	ATE	−0.0372	0.002	−0.129	0.054

*Forest behaviour*

**Table:**

Study	Outcome	*N*	Nt	Nc	Unit of analysis	TREATeff	Effect size	Variance	Lower bound	Upper bound
Sharma2017 Nepal Redd++	Firewood collection signs observed in the sampled forest plots	554	306	248	Plot/Village	ATE	0.1551	0.0073	−0.0126	0.3229
Sharma2017 Nepal Redd++	Fodder collection signs observed in the sampled forest plots	554	306	248	Plot/Village	ATE	0.0853	0.0073	−0.0822	0.2529
Sharma2017 Nepal Redd++	Timber extraction signs observed in the sampled forest plots	554	306	248	Plot/Village	ATE	−0.1707	0.0073	−0.3384	−0.0029
Sharma2017 Nepal Redd++	Encroachment signs observed in the sampled forest plots	554	306	248	Plot/Village	ATE	−0.2133	0.0073	−0.3812	−0.0454
Sharma2017 Nepal Redd++	Forest fire signs observed in the sampled forest plots	554	306	248	Plot/Village	ATE	−0.2133	0.0073	−0.3812	−0.0454
Jayachadran2016 Uganda PES	Program impacts on tree‐planting: Took up reforestation option		564	535	Plot/Village	ITT	0.4992	0.0038	0.3791	0.6193
Jayachadran2016 Uganda PES	Program impacts on tree‐planting: Total trees planted		564	535	Plot/Village	ITT	0.5259	0.0038	0.4056	0.6462
Jack2017 Malawi ICRAF	Has cleared land in last 3 years (Lottery)	319	205	114	Plot/Village	ITT	0.2809	0.0138	0.0508	0.5109
Jack2017 Malawi ICRAF	Has cleared land in last 3 years (Auction)	319	205	114	Plot/Village	ITT	0.2929	0.0138	0.0628	0.5230
Jack2017 Malawi ICRAF	Total plots cleared in last 3 years (Lottery)	319	205	114	Plot/Village	ITT	0.2583	0.0138	0.0285	0.4882
Jack2017 Malawi ICRAF	Total plots cleared in last 3 years (Auction)	319	205	114	Plot/Village	ITT	0.2381	0.0137	0.0083	0.4678
Jack2017 Malawi ICRAF	No. of plots planted with trees (Lottery)	319	205	114	Plot/Village	ITT	0.2322	0.0137	0.0025	0.4619
Jack2017 Malawi ICRAF	No. of plots planted with trees (Auction)	319	205	114	Plot/Village	ITT	0.0677	0.0137	−0.1614	0.2967
Jack2017 Malawi ICRAF	Total trees across all plots (Lottery)	319	205	114	Plot/Village	ITT	0.1493	0.0137	−0.0800	0.3786
Jack2017 Malawi ICRAF	Total trees across all plots (Auction)	319	205	114	Plot/Village	ITT	−0.0488	0.0137	−0.2778	0.1802
Jayachadran2017 Uganda PES	Cut any trees in the past year	994	NA	NA	Plot/Village	ITT	−0.3002	0.0041	−0.4252	−0.1751
Jayachadran2017 Uganda PES	Allow others to gather firewood from own forest	957	NA	NA	Plot/Village	ITT	−0.3622	0.0042	−0.4899	−0.2344
Jayachadran2017 Uganda PES	Increased patrolling of the forest in last 2 years	965	NA	NA	Plot/Village	ITT	0.1551	0.0042	0.0287	0.2814
Jayachadran2017 Uganda PES	Has any fence around land with natural forest	998	NA	NA	Plot/Village	ITT	0.0134	0.0040	−0.1107	0.1375
Jayachadran2016 Uganda PES	Cut trees to clear land for cultivation	994	NA	NA	Plot/Village	ITT	0.1444	0.0040	0.0199	0.2689
Jayachadran2016 Uganda PES	Cut trees for timber products	994	NA	NA	Plot/Village	ITT	−0.2261	0.0040	−0.3508	−0.1014
Jayachadran2016 Uganda PES	ES_Cut trees	994	NA	NA	Plot/Village	ITT	−0.1512	0.0040	−0.2757	−0.0266
Jayachadran2016 Uganda PES	Decreased access to others who take trees from forest in last 2 years	965	NA	NA	Plot/Village	ITT	0.0849	0.0041	−0.0413	0.2112
Jayachadran2016 Uganda PES	Had dispute with neighbors regarding and in last 2 years	998	NA	NA	Plot/Village	ITT	−0.0584	0.0040	−0.1825	0.0657
Jayachadran2016 Uganda PES	Claim to ownership of forest became stronger in last 2 years	982	NA	NA	Plot/Village	ITT	0.0928	0.0041	−0.0324	0.2179
Jayachadran2016 Uganda PES	Have planted trees in the past 12 mths	998	NA	NA	Plot/Village	ITT	0.2483	0.0040	0.1237	0.3729
Alix‐Garcia2015 Mexico PSAH	Quantity of firewood collected	1,162	NA	NA	Plot/Village	ATE	0.1327	0.0034	0.0176	0.2478

*Other intermediate outcomes*

**Table:**

Study	Outcome	*N*	Nt	Nc	Unit of analysis	TREATeff	Effect size	Variance	Lower bound	Upper bound
Demurger2012 China SLCP	Rural labour migration decision—probability of migration	5,068	3,072	1,996	Plot/Village	ATE	0.3397	0.0008	0.283	0.3964
Uchida2007 China SLCP	Migration status (number of adult migrants in household)	339	253	86	Plot/Village	ATT	0.0722	0.0156	−0.172	0.3169
Arriagada2015 Costa Rica PSA	Change in absentee status since 1996, Off‐farm in 1996 ‐> On‐farm in 2005 (dummy variable: 1 indicates living off‐farm in 1996 and living on‐farm in 2005) Residence 1	80	40	40	Plot/Village	ATT	−0.261	0.0504	−0.7007	0.1796
Arriagada2015 Costa Rica PSA	Change in absentee status since 1996, Off‐farm in 1996 ‐> On‐farm in 2005 (dummy variable: 1 indicates living off‐farm in 1996 and living on‐farm in 2005) Residence 2	80	40	40	Plot/Village	ATT	−0.256	0.0504	−0.6956	0.1845
Arriagada2015 Costa Rica PSA	Change in absentee status since 1996, Off‐farm in 1996 ‐> On‐farm in 2005 (dummy variable: 1 indicates living off‐farm in 1996 and living on‐farm in 2005) Residence 3	80	40	40	Plot/Village	ATT	0.276	0.0505	−0.1643	0.7164
Arriagada2015 Costa Rica PSA	Change in absentee status since 1996, Off‐farm in 1996 ‐> On‐farm in 2005 (dummy variable: 1 indicates living off‐farm in 1996 and living on‐farm in 2005) Residence 4	80	40	40	Plot/Village	ATT	0.2214	0.0503	−0.2182	0.6611

**Socio‐economic outcomes**

*Total household income*

**Table:**

Study	Outcome	*N*	Nt	Nc	Unit of analysis	TREATeff	Effect size	Variance	Lower bound	Upper bound
Liu et al. 2014—China—SLCP	Total income R	1,458	729	729	Household		0.04	0.0027	−0.06	0.14
Lin et al. 2014—China—SLCP	Household income consists of 1) on‐farm income, 2) off‐farm income and 3) other income.	189	94.5	94.5			0.07	0.0212	−0.21	0.36
Lin et al. 2014—China—SLCP	Household income consists of 1) on‐farm income, 2) off‐farm income and 3) other income.	234	117	117			0.07	0.0171	−0.19	0.32
Lin et al. 2014—China—SLCP	Household income consists of 1) on‐farm income, 2) off‐farm income and 3) other income.	200	100	100			0.07	0.0200	−0.21	0.35
Lin et al. 2014—China—SLCP	Household income consists of 1) on‐farm income, 2) off‐farm income and 3) other income.	269	134.5	134.5			0.06	0.0149	−0.18	0.30
Liang et al. 2012—China—SLCP	"Total income (on‐farm income, wage‐labor income, rural self‐employment non‐farm income, payments from participating in theGFG, and all other income)"	442	221	221			0.05	0.0091	−0.14	0.23
Liang et al. 2012—China—SLCP	"Total income (on‐farm income, wage‐labor income, rural self‐employment non‐farm income, payments from participating in theGFG, and all other income)"	366	183	183			0.25	0.0110	0.04	0.46
Liang et al. 2012—China—SLCP	"Total income (on‐farm income, wage‐labor income, rural self‐employment non‐farm income, payments from participating in theGFG, and all other income)"	132	66	66			0.09	0.0303	−0.25	0.43
Liang et al. 2012—China—SLCP	"Total income (on‐farm income, wage‐labor income, rural self‐employment non‐farm income, payments from participating in theGFG, and all other income)"	127	63.5	63.5			0.09	0.0315	−0.26	0.44
Zhang et al. 2005—China—DCBT Programme	Household per capital income	188	94	94			0.09	0.0213	−0.20	0.37

*Household income –agricultural sources*

**Table:**

Study	Outcome	*N*	Nt	Nc	Unit of analysis	TREATeff	Effect size	Variance	Lower bound	Upper bound
Xu et al. 2010—China—SLCP	Total agricultural income with subsidy	345	264	81	Household	ATE	0.3298	0.0163	0.0796	0.5800
Xu et al. 2010—China—SLCP	Husbandry income includes both sales income and own consumption, valued at market prices.	345	264	81	Household	ATE	0.2932	0.0163	0.0433	0.5431
Xu et al. 2010—China—SLCP	Cropping with subsidy (Cropping income consists of total crop production valued at average village market price, net of materials and hired labor costs).	345	264	81	Household	ATE	0.6568	0.0168	0.4031	0.9106
Xu et al. 2010—China—SLCP	Cropping before subsidy (Cropping income consists of total crop production valued at average village market price, net of materials and hired labor costs).	345	264	81	Household	ATE	0.6620	0.0168	0.4082	0.9158
Yao et al. 2010—China—SLCP	Animal husbandry income(income from raising livestock, predominantly goats)	600	492	108	Household	ATE	−0.2834	0.0114	−0.4923	−0.0745
Uchida et al. 2007—China—SLCP	Other agricultural income per capita (yuan)	339	253	86	Household	ATT	0.4135	0.0158	0.1669	0.6601
Duan et al. 2015—China—SLCP	Forest income	375	283	92	Household	ATE	0.0659	0.0144	−0.1694	0.3012
Zhang et al. 2013—China—PLDL	Agricultural income, %	723	394	329	Household	ATT	−0.4670	0.0057	−0.6154	−0.3187
Jack & Santos 2017—Malawi—ICRAF	Total income from crop sales—auction group	319	205	114	Household	ITT	0.2079	0.0137	−0.0217	0.4374
Liu et al. 2014—China—SLCP	Land‐based income (RL)—SLCP + NFPP	1,458	729	729	Household	ATE	−0.0200	0.0030	−0.1200	0.0800
Liang et al. 2012—China—SLCP	On‐farm income (income from crops and forests (fruits from trees).	442	221	221	Household	ATE	−0.1700	0.0090	−0.3600	0.0200
Liang et al. 2012—China—SLCP	On‐farm income (income from crops and forests (fruits from trees).	366	183	183	Household	ATE	−0.2900	0.0110	−0.5000	−0.0900
Liang et al. 2012—China—SLCP	On‐farm income (income from crops and forests (fruits from trees).	127	63.5	63.5	Household	ATE	−0.0900	0.0320	−0.4400	0.2600
Liu et al. 2014—China—DCPT programme	Land‐based income (RL)	1458	729	729	Household	ATE	−0.0400	0.0030	−0.1500	0.0600

*Non‐agricultural income*

**Table:**

Study	Outcome	N	Nt	Nc	Unit of analysis	TREATeff	Effect size	Variance	Lower bound	Upper bound
Xu et al. 20120—China—SLCP	“Other income consists of aquaculture, rental and interest income, gifts, pension income, and government subsidies and transfer payments”.	345	264	81	Household		−0.0196	0.0161	−0.2685	0.2294
Yao et al. 2010—China—SLCP	Other income(income from other sources, such as family properties and government subsidies)	600	492	108	Household		0.0149	0.0113	−0.1934	0.2231
Zhang et al. 2013—China—PLDL	Migrant income	723	394	329	Household		0.2223	0.0056	0.0755	0.3692
Jack et al. 2017—Malawi—ICRAF PES experiment	Casual labor income (0/1)—lottery group	319	205	114	Household		0.2438	0.0137	0.0141	0.4736
Jack et al. 2017—Malawi—ICRAF PES experiment	Casual labor income (0/1)—auction group	319	205	114	Household		0.1518	0.0137	−0.0775	0.3811
Liu et al. 2014—China—SLCP	Off‐farm income (RO)—SLCP + NFPPP	1,458	729	729	Household		0.0508	0.0027	−0.0519	0.1534
Liang et al. 20120—China—SLCP	Local wage‐income (income from working in the villages and towns).	442	221	221	Household		0.0475	0.0091	−0.1390	0.2340
Liang et al. 20120—China—SLCP	Local wage‐income (income from working in the villages and towns).	366	183	183	Household		0.2921	0.0110	0.0861	0.4981
Liang et al. 2012—China—SLCP	Local wage‐income (income from working in the villages and towns).	127	63.5	63.5	Household		0.0882	0.0315	−0.2598	0.4362
Liu et al. 2014—China—DCBT	Off‐farm income (RO)	1,458	729	729	Household		0.0143	0.0027	−0.0884	0.1169
Sharma et al. 2014—Nepal—REDD+ Pilot	Household income from CFUG (Community Forest User Groups) initiated activities in community Rs.	614	307	307	Household		0.0134	0.0065	−0.1448	0.1716
Sharma et al. 2014—Nepal—REDD+ Pilot	Gross income from CFUGs to the household in Rs.	614	307	307	Household		0.0347	0.0065	−0.1235	0.1929
Lin et al. 2014—China—SLCP	Household income consists of 1)on‐farm income, 2)off‐farm income and 3)other income.	269	134.5	134.5	Household		0.2189	0.0150	−0.0208	0.4586
Lin et al. 2014—China—SLCP	Household income consists of 1)on‐farm income, 2)off‐farm income and 3)other income.	234	117	117	Household		0.2346	0.0172	−0.0226	0.4917
Lin et al. 2014—China—SLCP	Household income consists of 1)on‐farm income, 2)off‐farm income and 3)other income.	200	100	100	Household		0.3381	0.0203	0.0590	0.6173
Lin et al. 2014—China—SLCP	Household income consists of 1)on‐farm income, 2)off‐farm income and 3)other income.	189	94.5	94.5	Household		0.2608	0.0213	−0.0255	0.5472

*Household assets*—*asset count*

**Table:**

Study	Outcome definition	*N*	Nt	Nc	Unit of analysis	Type of effect size	Effect size	Variance	Lower bound	Upper bound
Jindal 2012—Mozambique—Nhambita Project	Asset ownership per household (number))	291	238	53	Household	ATE	0.0891	0.02308	−0.2087	0.3869
Arriagada et al. 2015—Costa Rica—PSA	Household Change in Asset Count (2005 Count—1996 Count)	80	40	40	Household	ATT	−0.1586	0.05016	−0.5975	0.2804
PES009_Uchida_China_SLCP	Value of house (yuan)	339	253	86	Household	ATT	0.3126	0.01572	0.0668	0.5584
PES009_Uchida_China_SLCP	Fixed productive assets (yuan)	339	253	86	Household	ATT	0.0996	0.01560	−0.1451	0.3444
PES009_Uchida 2007	Livestock inventories (yuan)	339	253	86	Household	ATT	0.3412	0.01575	0.0953	0.5872

*Household assets*—*Asset index*

**Table:**

Study	Outcome definition	*N*	Nt	Nc	Unit of analysis	Type of effect size	Effect size	Variance	Lower bound	Upper bound
Jack & Santos 2017—Malawi—ICRAF	Asset index (Auction)	342	228	114	Household	ITT	0.102294	0.013173	−0.12266	0.327252
Alix‐Garcia et al. 2015a—Mexico—PSAH	Durables index—The durables index includes the following assets: television, refrigerator, computer, car, stove, phone, and cell phone (Common Property only)	1,844	NA	NA	Household	ATE	0.059591	0.001085	−0.00497	0.124155

*Education*

**Table:**

Study	Outcome	*N*	Nt	Nc	Unit of analysis	TREATeff	Effect size	Variance	Lower bound	Upper bound
Jindal2012—Mozambique	7 (Number of literates per Household)	291	238	53	Household	ATT	0.0842	0.0231	−0.2136	0.3819
Zheng2013—China	Education, yuan/hh	723	394	329	Household	ATT	0.1335	0.0056	−0.0130	0.2801
Alix‐Garcia2015a—Mexico	Education Investment ages 12–22 (Private Property)	201	NA	NA	Household	ATE	0.1068	0.0100	−0.1699	0.3835
Alix‐Garcia2015a—Mexico	Education Investment ages 15–17 (Common Property)	676	NA	NA	Household	ATE	0.1319	0.0030	−0.0190	0.2828
Alix‐Garcia2015a—Mexico	Education Investment ages 18–22 (Common Property)	979	NA	NA	Household	ATE	0.0493	0.0020	−0.0760	0.1746
Alix‐Garcia2015a—Mexico	Education Investment ages 12–14 (Common Property)	597	NA	NA	Household	ATE	0.0710	0.0034	−0.0895	0.2315

*Employment*

**Table:**

Study	Outcome	*N*	Nt	Nc	Unit of analysis	TREATeff	Effect size	Variance	Lower bound	Upper bound
Jindal 2012—Mozambique—Nhambita Project	Access to a permanent job or a small business (which translates into a regular source of cash income)	291	238	53	Plot	ATE	−32.9496	1.88849	−35.6431	−30.2561
Jindal 2012—Mozambique—Nhambita Project	(Households with access to wage labor in the village (%))	291	238	53	Plot	ATE	9.821751	0.18882	8.970064	10.67344
Groom 2010—China— SLCP	Househld off‐farm labour supply [Unconstrained](194 days per household per annum)	159	48	111	Plot	ATT	−0.13083	0.029896	−0.46973	0.20806
Groom 2010—China— SLCP	Househld off‐farm labour supply [constrained](194 days per household per annum)	159	48	111	Plot	ATT	0.635623	0.031113	0.289902	0.981344
Groom 2010—China—SLCP	Househld off‐farm labour supply [Pooled](194 days per household per annum)	159	48	111	Plot	ATT	0.038468	0.029847	−0.30015	0.377084
Uchida 2009—China—SLCP	Off‐farm labor status Change(Off farm labour includes any labor that is not on a farm).We define an individual to have an off‐farm occupation if the person engages in wage‐earning activities in an off‐farm firm or in nonfarm self employment for at least seven days in a given year.	956	818	138	Plot	ATT	0.251954	0.008502	0.071229	0.432679
Uchida 2009—China—SLCP	On‐farm labor status change	956	818	138	Plot	ATT	0.214253	0.008493	0.033626	0.39488
Uchida 2009—China—SLCP	Off‐farm work (number of adults with off‐farm work in household)	339	253	86	Plot	ATT	0.201761	0.015641	−0.04336	0.446883
Liu et al. 2015—China—SLCP	Household income diversity index (HDI)—using a dimensional diversification measurement called the inversed Herfindahl–Hirschman Index.	1,226	NA	NA	Household	ATE	0.1142	0.0033	0.0021	0.2262
Liu et al. 2015—China—SLCP	Household income diversity index (HDI) —using a dimensional diversification measurement called the inversed Herfindahl–Hirschman Index.	1,226	NA	NA	Household	ATE	0.1013	0.0033	−0.0107	0.2134
Liu et al. 2015—China—SLCP	Household income diversity index (HDI)—using a dimensional diversification measurement called the inversed Herfindahl–Hirschman Index.	1,226	NA	NA	Household	ATE	0.1998	0.0033	0.0876	0.3120
Liu et al. 2015—China—SLCP	Household income diversity index (HDI)—using a dimensional diversification measurement called the inversed Herfindahl–Hirschman Index.	1,226	NA	NA	Household	ATE	0.0870	0.0033	−0.0250	0.1990
Liu et al. 2015—China—SLCP	Household income diversity index (HDI)—using a dimensional diversification measurement called the inversed Herfindahl–Hirschman Index.	1,226	NA	NA	Household	ATE	0.1337	0.0033	0.0217	0.2458
Liu et al. 2015—China—SLCP	Household income diversity index (HDI)—using a dimensional diversification measurement called the inversed Herfindahl–Hirschman Index.	1,226	NA	NA	Household	ATE	0.1159	0.0033	0.0039	0.2279
Liu et al. 2015—China—SLCP	Household income diversity index (HDI)—using a dimensional diversification measurement called the inversed Herfindahl–Hirschman Index.	1,226	NA	NA	Household	ATE	0.1539	0.0033	0.0418	0.2660
Liu et al. 2015—China—SLCP	Household income diversity index (HDI)—using a dimensional diversification measurement called the inversed Herfindahl–Hirschman Index.	1,226	NA	NA	Household	ATE	0.1764	0.0033	0.0643	0.2886
Liu et al. 2015—China—SLCP	Household income diversity index (HDI)—using a dimensional diversification measurement called the inversed Herfindahl–Hirschman Index.	1,226	NA	NA	Household	ATE	0.1305	0.0033	0.0184	0.2426
Liu et al. 2015—China—SLCP	Household income diversity index (HDI)—using a dimensional diversification measurement called the inversed Herfindahl–Hirschman Index.	1,226	NA	NA	Household	ATE	0.0700	0.0033	−0.0420	0.1820
Liu et al. 2015—China—SLCP	Household income diversity index (HDI)—using a dimensional diversification measurement called the inversed Herfindahl–Hirschman Index.	1,226	NA	NA	Household	ATE	0.0285	0.0033	−0.0834	0.1405
Liu et al. 2015—China—SLCP	Household income diversity index (HDI)—using a dimensional diversification measurement called the inversed Herfindahl–Hirschman Index.	1,226	NA	NA	Household	ATE	0.0959	0.0033	−0.0161	0.2079
PES009_Liu Y 2018 ‐China SLCP	Off‐farm labor time inputs (person‐days) Both the SLCP and the NFPP (if yes= 1; otherwise = 0)	1,158	NA	NA	Plot	ATE	−0.2187	0.0035	−0.3343	−0.1032
PES009_Liu Y 2018 China SLCP	Off‐farm labor time inputs (person‐days) The SLCP (if yes = 1; otherwise = 0)	1,158	NA	NA	Plot	ATE	0.1566	0.0035	0.0413	0.2720
PES019_Liu Y 2018—China‐ DCBT	Off‐farm labor time inputs (person‐days) The DCBT (if yes= 1; otherwise = 0)	1,158	NA	NA	Plot	ATE	0.1288	0.0035	0.0135	0.2442

*Food security*

**Table:**

Study	Outcome	*N*	Nt	Nc	Unit of analysis	TREATeff	Effect size	Variance	Lower bound	Upper bound
Alix‐Garcia et al. 2015—Mexico—PSAH	"Food index = (The food index is constructed using households' reported prices and considering the consumption of tortillas, milk, beef, pork, cheese, bread, tomatos, and beans.) (Common Property)"	1,096	590	506	Household	ATE	0.0892	0.0037	−0.0296	0.2080
Alix‐Garcia et al. 2015—Mexico—PSAH	"Food index = (The food index is constructed using households' reported prices and considering the consumption of tortillas, milk, beef, pork, cheese, bread, tomatos, and beans.) (Private Property)"	114	60	54	Household	ATE	−0.0621	0.0352	−0.4298	0.3056
Jack 2017—Malawi—ICRAF	Per capita spending on food—Lottery group	319	205	114	Household	ITT	−0.1176	0.0137	−0.3467	0.1116
Jack 2017—Malawi—ICRAF	Per capita spending on food—Auction group	319	205	114	Household	ITT	0.1720	0.0137	−0.0574	0.4014
Jack 2017—Malawi—ICRAF	Months of food shortage—Lottery group	319	205	114	Household	ITT	−0.0413	0.0137	−0.2703	0.1877
Jack 2017—Malawi—ICRAF	Months of food shortage—Auction group	319	205	114	Household	ITT	0.1126	0.0137	−0.1166	0.3418
Jayachandran et al. 2017—Uganda—PES	IHS of food expend. in past 30 days	998	NA	NA	Household	ITT	−0.0262	0.0040	−0.1503	0.0979

*Other socioeconomic outcomes*

**Table:**

Study	Outcome	*N*	Nt	Nc	Unit of analysis	TREATeff	Effect size	Variance	Lower bound	Upper bound
PES001_Jindal2012	Number of m'shambas per Household)	291	238	53	Household	ATT	0.2214	0.0232	−0.0768	0.5197
PES012_Jayachandran2017	IHS of nonfood expend. in past 30 days	998	NA	NA	Household	ITT	0.0524	0.0040	−0.0717	0.1765
PES012_Jayachandran2016	IHS of alcohol/tobacco expend. In last 30 days	998	NA	NA	Household	ITT	−0.0759	0.0040	−0.2000	0.0482
PES012_Jayachandran2016	Has outstanding loan or repaid a loan in past year	996	NA	NA	Household	ITT	−0.1349	0.0040	−0.2593	−0.0106
PES012_Jayachandran2016	Child was sick with malaria in last 30 days (age 0–15)	2,145	NA	NA	Household	ITT	−0.1563	0.0019	−0.2411	−0.0715
PES012_Jayachandran2016	Child was sick with diarrhea in last 30 days (age 0–5)	470	NA	NA	Household	ITT	−0.3293	0.0086	−0.5114	−0.1473
PES007_Sims2017	localities with a greater than 5% share in PES, and Population growth Population data is from CONAPO and is convertedi n to density measures (hundreds of people per square km).	59,535	NA	NA	Plot	ATE	−0.0170	0.0001	−0.0331	−0.0010

*Poverty*

**Table:**

Study	Outcome	*N*	Nt	Nc	Unit of analysis	TREATeff	Effect size	Variance	Lower bound	Upper bound
John 2012—Tanzania—EPWS	Welfare	189	100	89	Household	ATT	0.321921	0.02151	0.034461	0.609381
Sims & Alix‐Garcia 2017—Mexico—PSAH	Poverty—based on a weighted average of indicators including rates of literacy, primary schooling, availability of potable water, sanitation and electricity, and housing characteristics. Localities with a greater than 5% share in PES.	59,535	NA	NA	Plot	ATE	0.027078	3.36E‐05	0.011012	0.043145
Beauchamp et al. 2018—Cambodia—Bird Nest Protection Program	Economic status was calculated using the Basic Necessities Survey (BNS) methodology, which incorporates multiple aspects of poverty into a single score for each household in the sample	596	177	419	Household	ATT	0.0448	0.0080	−0.1310	0.2205

**Environmental outcomes**

*Deforestation*

**Table:**

Study	Outcome	*N*	Nt	Nc	TREATeff	Effect size	Variance	Lower bound	Upper bound
Robalino et al. 2008—Costa Rica—PSA	Deforestation (2000–2005) 5 year effect (%)—observe land cover change in this period. Thus, if any location was covered by forest in 2000 but not in 2005, it is considered to have been deforested and is assigned a value of 1.	10,944	925	10,019	ATT	−0.02	0.0012	−0.08	0.05
Robalino et al. 2013—Costa Rica—PSA	Deforestation (1997–2000)—1 if point was deforested in 1997–2000 (=0 if not)—parcel of land	10,108	5,054	5,054	ATT	−0.06	0.0004	−0.09	−0.02
Robalino et al. 2015—Costa Rica—PSA	Deforestation (2000–2005)—observe land cover change in this period. Thus, if any location was covered by forest in 2000 but not in 2005, it is considered to have been deforested and is assigned a value of 1.	6,517	330	6,187	ATT	−0.12	0.0032	−0.23	−0.01
Robalino et al. 2015—Costa Rica—PSA	"Deforestation (2000–2005)—observe land cover change in this period. Thus, if any location was covered by forest in 2000 but not in 2005, it is considered to have been deforested and is assigned a value of 1. If a location was still covered by forest in 2005, it is assigned a value of 0. From GIS.	6,517	330	6,187	ATT	−0.08	0.0032	−0.19	0.03
Robalino et al. 2015—Costa Rica—PSA	Deforestation (2000–2005)—observe land cover change in this period. Thus, if any location was covered by forest in 2000 but not in 2005, it is considered to have been deforested and is assigned a value of 1. If a location was still covered by forest in 2005, it is assigned a value of 0. From GIS.Impact of PES in a buffer zone versus buffer zone no PES	3,530	556	2,974	ATT	−0.13	0.0021	−0.22	−0.04
De Velley et al. 2017—Mexico—PSAH	Forest loss within a polygon—2005–2012. SPOT GIS data and Time in Time in PSA‐H: Nonrenewed grids	7,331	4,911	2,420	ATE	0.01	0.0006	−0.04	0.06
De Velley et al. 2017—Mexico—PSAH	Forest loss within a polygon—2005–2012. SPOT GIS data and Time in PSA‐H: Renewed grids	7,331	4,911	2,420	ATE	−0.12	0.0006	−0.17	−0.08
De Velley et al. 2017—Mexico—PSAH	Forest loss within a polygon—2005–2012. SPOT GIS data andTime in PSA‐H: Newly enrolled grids (tpsalate)	7,331	4,911	2,420	ATE	−0.10	0.0006	−0.15	−0.05

*Forest cover*

**Table:**

Study	Outcome	*N*	Nt	Nc	Effect size	Variance	Lower bound	Upper bound
Arriagada_2008—Costa Rica—PSA	Self‐reported native forest cover change (ha)—model using only statistically significant covariates from logit	169	84.5	84.5	0.1144	0.0237	−0.1874	0.4162
Arriagada_2008—Costa Rica—PSA	Self‐reported native forest cover change (ha)—model using only statistically significant covariates from logit +imputed data	197	98.5	98.5	0.0475	0.0203	−0.2319	0.3268
Arriagada et al. 2011—Costa Rica—PSA	Net deforestation 1997–2005—from satelitte data—measured at the census tract level	8188	1050	7138	0.0925	0.0011	0.0277	0.1573
Arriagada_2012‐Costa Rica‐PSA	"Change in forestcover on the farm between 1992 and 2005—farm‐level forest cover (rather than in contracted parcels of PES land)—sample for which data may be imputed (full sample)"	202	50	152	0.4919	0.0272	0.1688	0.8150
Sierra2006_Costa Rica_PSA	Land use—% of land under intervened forest cover	60	30	30	0.3950	0.0680	−0.1160	0.9060
Sierra2006_Costa Rica_PSA	Land use—% of land under primary forest	60	30	30	−0.4791	0.0686	−0.9924	0.0342
Alix‐Garcia et al. 2015a—Mexico—PSAH	Per cent forest cover change (locality data)	52,824	26,412	26,412	0.0334	0.0001	0.0164	0.0505
Alix‐Garcia et al. 2015a—Mexico—PSAH	Average dry season normalized difference vegetation index (NDVI). (NDVI measures the “greenness” of vegetation based on the reflectance signatures of leafy vegetation) NDVI OUT 2004–2011	21,796	17,307	4,489	0.0558	0.0003	0.0230	0.0886
Sims & Alix‐Garcia 2017‐Mexico‐PSAH	the net change in forest cover from 2000–2012	59,535	29,767.5	29,767.5	−0.0186	0.0001	−0.0346	−0.0025
Lokina2016_Tanzania_EPWS	Perception of the forest size	198	100	98	0.1071	0.0202	−0.1717	0.3859
Jayachandran2016_Uganda‐PES	Reforestation area	1099	564	535	0.3807	0.0037	0.2614	0.5001
Jayachandran2016_Uganda‐PES	Total trees survived	1099	564	535	0.3806	0.0037	0.2612	0.4999
Jayachandran2016_Uganda‐PES	Tree cover—spillovers/anticipation effects Treat * Distance to forest reserve	995	497.5	497.5	0.0163	0.0040	−0.1080	0.1406
Jayachandran2016_Uganda‐PES	Tree cover—spillovers/anticipation effects Treat Contiguous to forest reserve	995	497.5	497.5	−0.0632	0.0040	−0.1875	0.0611
Jayachandran2016_Uganda‐PES	Tree cover—spillovers/anticipation effects # of treatment villages within 5 km	487	243.5	243.5	0.0441	0.0082	−0.1336	0.2217
Jayachandran2016_Uganda‐PES	Tree cover—spillovers/anticipation effects Believes program likely to come to village	487	243.5	243.5	0.0917	0.0082	−0.0860	0.2694
Jayachandran2016_Uganda‐PES	Tree cover—spillovers/anticipation effects Believes program ends in 2015 or later	508	254	254	−0.0934	0.0079	−0.2674	0.0807
Jayachandran2017_Uganda‐PES	PFO‐level land circles: Change in tree cover (ha)	995	497.5	497.5	0.1596	0.0040	0.0351	0.2841

*Other environmental outcomes*

**Table:**

Study	Outcome	*N*	Nt	Nc	Unit of analysis	TREATeff	Effect size	Variance	Lower bound	Upper bound
Sharma et al. 2015—Nepal—REDD + Pilot	Total forest carbon—Weight of total forest carbon (tons per hectare) in the sample plot; measured by summing the forest soil carbon and converted value of biomass into carbon equivalent	554	306	248	Plot	ATE	0.0892	0.0073	−0.0783	0.2568
Sharma et al. 2015—Nepal—REDD + Pilot	Tree crown cover observed in the sampled forest plots	554	306	248	Plot	ATE	0.2133	0.0073	0.0454	0.3812
Sharma et al. 2015—Nepal—REDD + Pilot	Shrub cover observed in the sampled forest plots	554	306	248	Plot	ATE	−0.0569	0.0073	−0.2244	0.1106
Sharma et al. 2015—Nepal—REDD + Pilot	Grass cover observed in the sampled forest plots	554	306	248	Plot	ATE	0.1991	0.0073	0.0312	0.3670
Sharma et al. 2015—Nepal—REDD + Pilot	Soil erosion signs observed in the sampled forest plots	554	306	248	Plot	ATE	−0.1463	0.0073	−0.3140	0.0214
Sharma et al. 2015—Nepal—REDD + Pilot	Signs of wildlife observed in the sampled forest plots	554	306	248	Plot	ATE	0.1862	0.0073	0.0183	0.3540
Pagiola et al. 2013—Colombia—Silvopastoral Project	PES with technical assistance (1=yes)‐ LN Change in ESI Per HA	101	72	29	Household	ATE	0.0833	0.0484	−0.3479	0.5146
Pagiola et al. 2013—Colombia—Silvopastoral Project	PES with technical assistance (1=yes)‐ LN Change in ESI	101	72	29	Household	ATE	0.1819	0.0485	−0.2499	0.6137
Pagiola et al. 2013—Colombia—Silvopastoral Project	PES with technical assistance (1=yes)‐ Change in ESI per HA	101	72	29	Household	ATE	0.1675	0.0485	−0.2642	0.5992
Pagiola et al. 2013—Colombia—Silvopastoral Project	PES with technical assistance (1=yes)‐ Change in ESI	101	72	29	Household	ATE	0.3577	0.0490	−0.0761	0.7916
Pagiola et al. 2013—Colombia—Silvopastoral Project	PES recipient (1=yes) Ln(change in ESI)	101	72	29	Household	ATE	0.2025	0.0486	−0.2295	0.6344
Pagiola et al. 2013—Colombia—Silvopastoral Project	PES recipient (1=yes) LN Change in ESI (Environmental services index) PER HA	101	72	29	Household	ATE	0.0283	0.0484	−0.4028	0.4594
Pagiola et al. 2013—Colombia—Silvopastoral Project	PES recipient (1=yes) Change in ESI per HA (Environmental services index)	101	72	29	Household	ATE	0.1830	0.0485	−0.2489	0.6148
Pagiola et al. 2013—Colombia—Silvopastoral Project	PES recipient (1=yes) Change in ESI (Environmental services index)	101	72	29	Household	ATE	−0.1403	0.0485	−0.5718	0.2913
Mohebalian & Aguilar 2018—Ecuador—Socio Bosque	Tree species richness (Frequency)	38	19	19	Household	ATT	1.0499	0.1198	0.3716	1.7282
Mohebalian & Aguilar 2018—Ecuador—Socio Bosque	Trees species at risk of extinction (Frequency)	38	19	19	Household	ATT	0.1915	0.1057	−0.4458	0.8289
Mohebalian & Aguilar 2018—Ecuador—Socio Bosque	Tree species with commercial timber value (Frequency)	38	19	19	Household	ATT	0.4975	0.1085	−0.1481	1.1432
Pagiola et al. 2016—Colombia—Silvopastoral Project	4 year PES+ Technical assistance=1 ESI per ha 2011—follow up data from the above, post‐PES implementation (2007‐2011)	85	NA	NA	Household	ATE	0.0887	0.0471	−0.3367	0.5141
Pagiola et al. 2016—Colombia—Silvopastoral Project	2 year PES+ Technical assistance ESI per ha 2011—follow up data from the above, post‐PES implementation (2007‐2011)	85	NA	NA	Household	ATE	0.1803	0.0473	−0.2458	0.6063
Pagiola et al. 2016—Colombia—Silvopastoral Project	4 year PES=1 ESI per ha 2011—follow up data from the above, post‐PES implementation (2007‐2011)	85	NA	NA	Household	ATE	−0.0969	0.0471	−0.5223	0.3286

## References

[cl21045-bib-0001] Alix‐Garcia, J. , & Wolff, H. (2014). Payment for ecosystem services from forests. Annual Review of Resource Economics, 6, 361–380.

[cl21045-bib-0002] Alix‐Garcia, J , Aronson, G , Radeloff, V , Ramirez‐Reyes, C , Shapiro, E , Sims, K , & Yañez‐Pagans, P , (2014) *Environmental and socioeconomic impacts of Mexico's payments for ecosystem services program* (3ie Impact Evaluation Report 20). New Delhi: International Initiative for Impact Evaluation (3ie)

[cl21045-bib-0003] Agrawal, A. , & Angelsen, A. (2009). Using community forest management to achieve REDD+ goals. In S. Angelsen , A. Brockhaus , M. Kanninen , M. Sills , E. Sunderlin , & W. D. Wertz‐Kanounnikoff (Eds.), Realising REDD: National strategy and policy options (pp. 201–211). Bogor: Center for International Forestry Research (CIFOR).

[cl21045-bib-0004] Angelsen, A. (2009). Policy options to reduce deforestation. In A. Angelsen , M. Brockhaus , M. Kanninen , E. Sills , W. D. Sunderlin , & S. Wertz‐Kanounnikoff (Eds.), Realising REDD+: National strategy and policy options (pp. 125–138). Bogor: Center for International Forestry Research (CIFOR).

[cl21045-bib-0005] Arriagada, R. , & Perrings, C. (2009). Making Payments for Ecosystem Services Work, Ecosystem Services Economics. Nairobi: United Nations Environment Program.

[cl21045-bib-0006] Baird, S. , Ferreira, F. H. G. , Özler, B. , & Woolcock, M. (2013). Relative effectiveness of conditional and unconditional cash transfers for schooling outcomes in developing countries: A systematic review. Campbell Systematic Reviews, 2013(8).

[cl21045-bib-0007] BMUB (German Federal Ministry for the Environment, Nature Conservation, Building and Nuclear Safety) . (2015) Joint Statement by Germany, Norway and the United Kingdom of Great Britain and Northern Ireland. Unlocking the Potential of Forests and Land Use. Paris, COP21.

[cl21045-bib-0008] Borenstein, M. , Hedges, L. V. , Higgins, J. P. T. , & Rothstein, H. (2009). Introduction to meta analysis (statistics in practice). Chichester: John Wiley & Sons.

[cl21045-bib-0009] Börner, J. , Baylis, K. , Corbera, E. , Ezzine‐De‐Blas, D. , Honey‐Rosés, J. , Persson, U. M. , & Wunder, S. (2017). The effectiveness of payments for environmental services. World development, 96, 359–374

[cl21045-bib-0010] Climate Focus . (2015) *Progress on the New York declaration on forests—An assessment framework and initial report*. Prepared by Climate Focus, in collaboration with Environmental Defense Fund, Forest Trends, The Global Alliance for Clean Cookstoves, and The Global Canopy Program.

[cl21045-bib-0011] Critical Appraisal Skills Programme . (2006) *10 questions to help you make sense of qualitative research*. Public Health Resource Unit. Retrieved from http://www.biomedcentral.com/content/supplementary/2046‐4053‐3‐139‐S8.pdf

[cl21045-bib-0012] Démurger, D. , & Wan, H. (2012). Payments for ecological restoration and rural labor migration in China: The sloping land conversion program in Ningxia. IZA Journal of Migration, 1(10), 1–10.

[cl21045-bib-0013] Dhaliwal, I. , Duflo, E. , Glennerster, R. , & Tulloch, C. (2012). *Comparative Cost‐Effectiveness Analysis to Inform Policy in Developing Countries: A General Framework with Applications for Education*. Poverty Action Lab, MIT. Available from https://www.povertyactionlab.org/sites/default/files/publications/CEA%20in%20Education%202013.01.29_0.pdf. Accessed September 11, 2019

[cl21045-bib-0014] Dixon‐Woods, M. , Agarwal, S. , Jones, D. , Young, B. , & Sutton, A. (2005). Synthesising qualitative and quantitative evidence: a review of possible methods. Journal of Health Services Research and Policy, 10(1), 45–53. 15667704 10.1177/135581960501000110

[cl21045-bib-0015] Duval, S. , & Tweedie, R. (2000). A nonparametric “trim and fill” method of accounting for publication bias in meta‐analysis. Journal of the American Statistical Association, 95, 89–98.

[cl21045-bib-0016] Egger, M. , Smith, G. D. , Schneider, M. , & Minder, C. (1997). Bias in meta‐analysis detected by a simple, graphical test. BMJ, 315, 629–634. 9310563 10.1136/bmj.315.7109.629 PMC2127453

[cl21045-bib-0017] Ellis, P. D. (2010). The essential guide to effect sizes: Statistical power, meta‐analysis, and the interpretation of research results, New York, NY: Cambridge University Press.

[cl21045-bib-0018] Engel, S. , Pagiola, S. , & Wunder, S. (2008). Designing payments for environmental services in theory and practice: An overview of the issues. Ecological Economics, 65, 663–674.

[cl21045-bib-0019] Ezzine‐de‐Blas, D. , Wunder, S. , Ruiz‐Pérez, M. , & Moreno‐Sanchez, R. P. (2016). Global patterns in the implementation of payments for environmental services. PLOS One, 11(3), e0149847. 26938065 10.1371/journal.pone.0149847 PMC4777491

[cl21045-bib-0020] FAO and IPCC . (2017) *FAO‐IPCC Expert meeting on climate change, land use and food security* (Meeting report). Rome, Italy.

[cl21045-bib-0021] FAO . (2016a). The state of food and agriculture: Climate change, agriculture and food security. Rome: FAO.

[cl21045-bib-0022] FAO . (2016b). State of the world's forests 2016. Rome: FAO.

[cl21045-bib-0023] FAO (2016c). Global Forest Resources Assessment 2015: How are the world's forests changing? (2nd ed.). Rome: FAO.

[cl21045-bib-0024] FAO . (2013). The state of food insecurity in the world: The multiple dimensions of food security. Rome: United Nation Food and Agriculture Organization.

[cl21045-bib-0025] FAO . (2012). Forest resources assessment 2015: Terms and definitions. Rome: FAO.

[cl21045-bib-0026] FAO . (2009). Declaration of the world summit on food security. Rome: United Nation Food and Agriculture Organization.

[cl21045-bib-0027] FAO . (2005). Grasslands of the world. Rome: FAO.

[cl21045-bib-0028] Ferraro, P. (2011). The future of payments for environmental services. Conservation Biology, 25(6), 1134–1138. 22070269 10.1111/j.1523-1739.2011.01791.x

[cl21045-bib-0029] Ferraro, P. J. (2017). Are payments for ecosystem services benefiting ecosystems and people? In P. Kareiva , M. Marvier , & B. Silliman (Eds.), Effective conservation science: Data not dogma. Oxford: Oxford Scholarship.

[cl21045-bib-0030] Ferraro, P. J. , & Miranda, J. J. (2017). Panel data designs and estimators as substitutes for randomised controlled trials in the evaluation of public programs. JAERE, 4(1), 281–317.

[cl21045-bib-0031] Ferraro, P. J. , & Miranda, J. J. (2014). The performance of non‐experimental designs in the evaluation of environmental programs: A design‐replication study using a large‐scale randomized experiment as a benchmark. Journal of Economic Behavior and Organization, 107, 344–365.

[cl21045-bib-0032] Ferraro, P. J. , & Pattanayak, S. K. (2006). Money for nothing? A call for empirical evaluation of biodiversity conservation investments. PLoS Biology, 4(4), e105. 10.1371/journal.pbio.0040105 16602825 PMC1435411

[cl21045-bib-0033] Garbach, K. , Lubell, M. , & DeClerck, F. A. J. (2012). Payment for ecosystem services: the roles of positive incentives and information sharing in stimulating adoption of silvopastoral conservation practices. Agriculture, and Ecosystems and Environment, 156, 27–36.

[cl21045-bib-0034] Gleser, L. J. , & Olkin, I. (2007). *Stochastically dependent effect sizes* (Technical report No. 2007‐2). Stanford, California: Stanford University.

[cl21045-bib-0035] Global Environment Facility . (2014) *GEF Investments on Payment for Ecosystem Services Schemes*. GEF

[cl21045-bib-0036] Hammerstrøm, K. , Wade, A. , & Jørgensen, A.‐M.K. (2010). Searching for studies: A guide to information retrieval for Campbell Systematic Reviews. *Campbell Systematic Reviews 2010*, Suppl 1. Retrieved from www.campbellcollaboration.org/lib/download/969/ 10.1002/cl2.1433 PMC11386270 39258215

[cl21045-bib-0037] Hansen, M.C. , et al. (2013). High‐resolution global maps of 21st‐century forest cover change. Science, 342(6160), 850–853. 10.1126/science.1244693 24233722

[cl21045-bib-0038] Hegde, R. , & Bull, G. Q. (2011). Performance of an agro‐forestry based payments‐for‐environmental‐services project in Mozambique: A household level analysis. Ecological Economics, 71, 122–130.

[cl21045-bib-0039] Hedges, L. V. , Tipton, E. , & Johnson, M. C. (2010). Robust variance estimation in meta‐regression with dependent effect size estimates. Research Synthesis Methods, 1, 39–65. 26056092 10.1002/jrsm.5

[cl21045-bib-0040] Higgins, J. , & Green, S. (2011). *Cochrane handbook for systematic reviews of interventions*. (version 5.0.2). The Cochrane Collaboration.

[cl21045-bib-0041] HM Treasury . (2011). *Magenta book*. Retrieved from http://www.hm‐treasury.gov.uk/data_magentabook_index.htm

[cl21045-bib-0042] Hombrados, G. J. , & Waddington, H. (2012). *Internal validity in social experiments and quasi experiments: An assessment tool for reviewers* (Unpublished working document). International Initiative for Impact Evaluation (3ie).

[cl21045-bib-0043] IPCC . (2014). Climate change 2014: Synthesis report. Contribution of working groups I, ii and iii to the fifth assessment report of the intergovernmental panel on climate change. Geneva: IPCC.

[cl21045-bib-0044] Jayachandran, S. , de Laat, j , Lambin, E. F. , & Stanton, C. Y. (2016). *Cash for carbon: A randomized controlled trial of payments for ecosystem services to reduce deforestation* (NBER Working Paper No. 22378.). Cambridge, MA: NBER. 10.1126/science.aan0568 28729505

[cl21045-bib-0045] Le Velly, G. , & Dutilly, C. (2016). Evaluating payments for environmental services: Methodological challenges. PLOS One, 11(2):e0149374. 26910850 10.1371/journal.pone.0149374 PMC4766196

[cl21045-bib-0046] Larson, A. M. , Brockhaus, M. , Sunderlin, W. D. , Duchelle, A. , Babon, A. , Dokken, T. , … Huynh, T. B. (2013). Land tenure and REDD+: the good, the bad and the ugly. Global Environmental Change, 23, 678–689.

[cl21045-bib-0047] Lawlor, K. , Madeira, E. , Blockhus, J. , & Ganz, D. (2013). Community participation and benefits in REDD+: A review of initial outcomes and lessons. Forests, 4, 296–318.

[cl21045-bib-0048] Lipsey, M. , & Wilson, D. (2001). Practical Meta‐analysis. Thousand Oaks, CA: Sage publications Ltd.

[cl21045-bib-0049] McEwan, P. J. (2012). Cost‐effectiveness analysis of education and health interventions in developing countries. Journal of Development Effectiveness, 4, 189–213.

[cl21045-bib-0050] Millennium Ecosystem Assessment (MEA) . (2005). Ecosystems and human well‐being: Policy Responses: Findings of the Responses Working Group of the Millennium Ecosystem Assessment. Washington, DC: Island Press.

[cl21045-bib-0051] Muradian, R. , Corbera, E. , Pascual, U. , Kosoy, N. , & May, P. H. (2010). Reconciling theory and practice: An alternative conceptual framework for understanding payments for environmental services. Ecological Economics, 69(6), 1202–1208.

[cl21045-bib-0052] Mutabazi, K. D. , George, C. K. , Dos Santos, A. S. , & Felister, M. M. (2014). Livelihood implications of REDD+ and costs‐benefits of agricultural intensification in REDD+ pilot area of kilosa, Tanzania. Journal of Ecosystems and Ecography, 4(2), 144.

[cl21045-bib-0053] O'Mara‐Eves, A. , Thomas, J. , McNaught, J. , Miwa, M. , Ananiadou, S. , O'Mara‐Eves, A. , … Ananiadou, S. (2015). Erratum to: Using text mining for study identification in systematic reviews: A systematic review of current approaches. Systematic Reviews, 4(5), 1–22. 25588314 10.1186/2046-4053-4-5 PMC4320539

[cl21045-bib-0054] Pattanayak, S. K. , Wunder, S. , & Ferraro, P. J. (2010). Show me the money: Do payments supply environmental services in developing countries? Review of Environmental Economics and Policy, 4(2), 254–274. summer 2010.

[cl21045-bib-0055] Pirard, R. (2012). Market‐based instruments for biodiversity and ecosystem services: a lexicon. Environmental Science and Policy, 19–20(2012), 59–68.

[cl21045-bib-0056] Pluye, P. , Robert, E. , Cargo, M. , Bartlett, G. , O'Cathain, A. , Griffiths, F. , … Rousseau, M.C. (2011). *Proposal: A mixed methods appraisal tool for systematic mixed studies reviews*. Retrieved from http://mixedmethodsappraisaltoolpublic.pbworks.com. Archived by WebCite® at http://www.webcitation.org/5tTRTc9yJ

[cl21045-bib-0057] Puri, J. , Nath, M. , Bhatia, R. , & Glew, L. (2016). *Examining the evidence base for forest conservation interventions* (Evidence Gap Map Report 4). New Delhi: International Initiative for Impact Evaluation (3ie).

[cl21045-bib-0058] R Development Core Team . (2008). R: A language and environment for statistical computing. Vienna, Austria: R Foundation for Statistical Computing. ISBN 3‐900051‐07‐0.

[cl21045-bib-0059] Robinson, B. E. , Masuda, Y. J. , Kelly, A. , Holland, M. B. , Bedford, C. , Childress, M. , … Veit, P. (2017). Incorporating land tenure security into conservation. Conservation Letters.

[cl21045-bib-0060] Rubenstein, L. V. , Williams, J. , Danz, M. , & Shekelle, P. (2009). Determining key features of effective depression interventions. Los Angeles, CA: Greater Los Angeles Veterans Affairs Healthcare System/Southern California/RAND Evidence‐based Practice Centre. 21155205

[cl21045-bib-0061] Samii, C. , Lisiecki, M. , Kulkarni, P. , Paler, L. , Chavis, L. , Snilstveit, B. , … Gallagher, E. (2014). Effects of payment for environmental services (PES) on deforestation and poverty in low and middle income countries: A systematic review. Campbell Systematic Reviews, 10, 1–95.

[cl21045-bib-0062] Schomers, S. , & Matzdorf, B. (2013). Payments for ecosystem services: A review and comparison of developing and industrialized countries. Ecosystem Services, 6, 16–30.

[cl21045-bib-0063] Shadish, W. , & Myers, D. (2004). *Research design policy brief*. Oslo: Campbell Collaboration. Retrieved from http://www.campbellcollaboration.org/artman2/uploads/1/C2_Research_Design_Policy_Brief‐2.pdf

[cl21045-bib-0064] Sharma, B. P. , & Pattanayak, S. (2015). *REDD+ impacts: Evidence from Nepal*. Kathmandu: South Asian Network for Development and Environmental Economics (SANDEE). Retrieved from http://www.sandeeonline.org/uploads/documents/publication/1064_PUB_Working_Paper_95_Bishnu_et_al.pdf

[cl21045-bib-0065] Shemilt, I. , Khan, A. , Park, S. , & Thomas, J. (2016). Use of cost‐effectiveness analysis to compare the efficiency of study identification methods in systematic reviews. Systematic Reviews, 5(140), 1–13. 27535658 10.1186/s13643-016-0315-4 PMC4989498

[cl21045-bib-0066] Shemilt, I. , Mugford, M. , Byford, S. , Drummond, M. , Eisenstein, E. , Knap, M. , & Walker, D. (2008). *The Campbell Collaboration Economics Methods Policy Brief*. Oslo: Campbell Collaboration. Retrieved from http://www.campbellcollaboration.org/artman2/uploads/1/Economic_Methods_Policy_Brief.pdf

[cl21045-bib-0067] Shemilt, I. , Valentine, J. C. , Pössel, P. , Mugford, M. , & Wooldridge, D. T. (2012). Costing program implementation using systematic reviews: Interventions for the prevention of adolescent depression. Research Synthesis Methods, 3, 191–201. 26062162 10.1002/jrsm.1041

[cl21045-bib-0068] Sills, E. , Arriagada, R. A. , Ferraro, P. J. , Pattanayak, S. K. , Carrasco, L. E. , Ortiz, E. , … Andam, K. (2008). *Private provision of public goods: Evaluating payments for ecosystem services in Costa Rica* (Working Paper). Raleigh: North Carolina State University.

[cl21045-bib-0069] Snilstveit, B. , Stevenson, J. , Langer, L. , Polanin, J. , Shemilt, I. , Eyers, J. , & Ferraro, P. J. (2018). Protocol: Incentives for climate mitigation in the land use sector: A mixed‐methods systematic review of the effectiveness of payment for environment services (PES) on environmental and socio‐economic outcomes in low‐ and middle‐income countries. Campbell Systematic Reviews, 14, 1–77. 10.1002/CL2.209 PMC8427996 37131365

[cl21045-bib-0070] Snilstveit, B. , Stevenson, J. , Villar, P.F. , Eyers, J. , Harvey, C. , Panfil, S. , … McKinnon, M.C. (2016). *Land‐use change and forestry programmes: evidence on the effects on greenhouse gas emissions and food security* (Evidence Gap Map Report 3). London: International Initiative for Impact Evaluation (3ie).

[cl21045-bib-0071] Snilstveit, B. , Stevenson, J. , Phillips, D. , Vojtkova, M. , Gallagher, E. , Schmidt, T. , … Eyers, J. (2015). Interventions for improving learning outcomes and access to education in low‐ and middle‐ income countries: a systematic review. 3ie Systematic Review 24. London: International Initiative for Impact Evaluation (3ie).

[cl21045-bib-0072] Snilstveit, B. (2012). Systematic reviews: From ‘bare bones’ reviews to policy relevance. Journal of Development Effectiveness, 4(3), 388–408.

[cl21045-bib-0073] Stickler, C. M. , Nepstad, D. C. , Coe, M. T. , Mcgrath, D. G. , Rodrigues, H. O. , Walker, W. S. , … DAVIDSON, E. A. (2009). The potential ecological costs and cobenefits of REDD: A critical review and case study from the Amazon region. Global Change Biology, 15, 2803–2824.

[cl21045-bib-0074] Tanner‐Smith, E. E. , & Tipton, E. (2014). Robust variance estimation with dependent effect sizes: Practical considerations including a software tutorial in stata and SPSS. Research Synthesis Methods, 5, 13–30. 26054023 10.1002/jrsm.1091

[cl21045-bib-0075] The Steering Group of the Campbell Collaboration . (2016). *Campbell systematic reviews: Policies and guidelines* (Campbell P). The Campbell Collaboration.

[cl21045-bib-0076] Thomas, J. , & Harden, A. (2008). Methods for the thematic synthesis of qualitative research in systematic reviews. BMC Medical Research Methodology, 8(45), 1–10. 18616818 10.1186/1471-2288-8-45 PMC2478656

[cl21045-bib-0077] UNFCCC. (2005). *Reducing emissions from deforestation in developing countries: approaches to stimulate action ‐ Submissions from Parties*. Conference of the Parties Eleventh Session Montreal, 28 November to 9 December. Available from https://unfccc.int/resource/docs/2005/cop11/eng/misc01.pdf. Accessed September 11, 2019.

[cl21045-bib-0078] UNFCCC (2009). Investment and Financial Flows to Address Climate Change: An Update. Bonn: United Nations Framework Convention on Climate Change.

[cl21045-bib-0079] UNFCCC . (2015). *Adoption of the Paris agreement, Decision ‐/CP.21. Bonn, Germany, United Nations Framework Convention on Climate Change (UNFCCC)*. Retrieved from http://unfccc.int/files/meetings/paris_nov_2015/application/pdf/cop_auv_template_4b_new__1.pdf

[cl21045-bib-0080] UNFCCC . (2010). *The Cancun agreements: Outcome of the work of the ad hoc working group on long‐term cooperative action under the convention, Decision 1/CP.16*.

[cl21045-bib-0081] UN‐REDD . (2016). Key achievements of the UN‐REDD programme, 2008‐2016. Geneva: UN‐REDD Programme Secretariat.

[cl21045-bib-0082] Viechtbauer, W. (2010). Conducting meta‐analyses in R with the metafor package. Journal of Statistical Software, 36(3), 1–48.

[cl21045-bib-0083] Waddington, H. , White, H. , Snilstveit, B. , Hombrados, J. G. , Vojtkova, M. , Davies, P. , … Tugwell, P. (2012). How to do a good systematic review of effects in international development: A tool kit. Journal of Development Effectiveness, 4(3), 359–387.

[cl21045-bib-0084] White, H. (2009). *Theory‐based impact evaluation: Principles and practice* (Working paper series No. 3). New Delhi: 3ie.

[cl21045-bib-0085] Wilson, D. B. , Weisburd, D. , & McClure, D. (2011). Use of DNA testing in police investigative work for increasing offender identification, arrest, conviction and case clearance. Campbell Systematic Reviews, 7(1), 1–53.

[cl21045-bib-0086] Wunder, S. (2015). Revisiting the concept of payments for environmental services. Ecological Economics, 117, 234–243.

